# Highly active ruthenium metathesis catalysts enabling ring-opening metathesis polymerization of cyclopentadiene at low temperatures

**DOI:** 10.1038/s41467-019-11806-5

**Published:** 2019-08-27

**Authors:** Kitaek Song, Kunsoon Kim, Daeun Hong, Jungwon Kim, Chae Eun Heo, Hugh I. Kim, Soon Hyeok Hong

**Affiliations:** 1 Department of Chemistry, Korea Advanced Institute of Science and Technology (KAIST), Daejeon, 34141 Republic of Korea; 2 Department of Chemistry, College of Natural Sciences, Seoul National University, Seoul, 08826 Republic of Korea; 3 Department of Chemistry, Korea University, Seoul, 02841 Republic of Korea

**Keywords:** Catalysis, Organic chemistry, Polymer chemistry

## Abstract

Development of versatile ruthenium olefin-metathesis catalysts with high activity, stability, and selectivity is a continuous challenge. Here we report highly controllable ruthenium catalysts using readily accessible and versatile *N*-vinylsulfonamides as carbene precursors. Catalyst initiation rates were controlled in a straightforward manner, from latent to fast initiating, through the facile modulation of the *N*-vinylsulfonamide ligands. Trifluoromethanesulfonamide-based catalysts initiated ultrarapidly even at temperatures as low as −60 °C and continuously propagated rapidly, enabling the enthalpically and entropically less-favored ring-opening metathesis polymerizations of low-strained functionalized cyclopentene derivatives, some of which are not accessible with previous olefin-metathesis catalysts. To our surprise, the developed catalysts facilitated the polymerization of cyclopentadiene (CPD), a feedstock that is easily and commonly obtainable through the steam cracking of naphtha, which has, to the best of our knowledge, not been previously achieved due to its low ring strain and facile dimerization even at low temperatures (below 0 °C).

## Abstract (web summary)

Due to the low ring strain and facile dimerization, ring-opening metathesis polymerization of cyclopentadiene, readily obtainable from petroleum feedstock, has not been realized. Here the authors show an ultrarapidly initiating trifluoromethanesulfonamide-based ruthenium catalyst enables it at low temperatures.

## Introduction

**Fig. 1:**
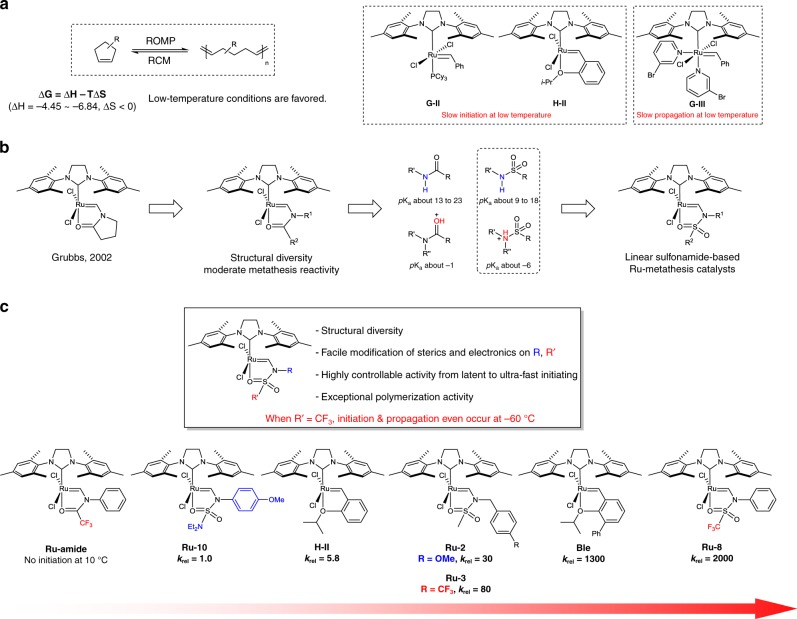
Development of *N*-vinylsulfonamide-based olefin-metathesis catalysts. **a** Representative Ru-based olefin-metathesis catalysts have limited initiation or propagation properties in ROMP at low temperatures. **b** The development of *N*-vinylsulfonamide-based metathesis catalysts. **c** Comparison of the initiation rates of Ru-based olefin-metathesis catalysts. The *k*_init_ values were determined by reacting a catalyst with 30 equiv. of butyl vinyl ether (BVE) at 10 °C in toluene (0.1 mM)

Olefin metathesis is a key C–C double-bond forming reaction that is widely applied in the chemical synthesis^1,2^, pharmaceuticals^3,4^, agrochemicals^5^, and polymer industries^5,6^. Polymerization, especially ring-opening metathesis polymerization (ROMP), has been studied for several decades, as it is a highly efficient way of obtaining a variety of functionalized linear and controlled polymers^6^. The basic driving force for ROMP is the release of the strain energies of constrained monomers, such as norbornene and its derivatives (>20 kcal/mol)^6–8^. Cyclopentenes, however, have significantly lower ring strains (4.5–6.8 kcal/mol) than those of norbornene derivatives; consequently, fewer examples of ROMP involving cyclopentenes have been reported^9–15^. Notably, the ROMP of cyclopentadiene (CPD), which is easily obtained by the cracking of naphtha and rapidly dimerized to dicyclopentadiene (DCPD) even below 0 °C, has, to the best of our knowledge, never been achieved. Polymers derived from cyclopentenes are attractive and potentially highly useful materials because of their natural rubber-like properties and their recyclability through the ring-closing metathesis (RCM) of the polypentenamer^12,16^. In addition, functionalized linear polymers obtained by the ROMP of functionalized cyclopentenes, such as hydroxyl^17^-, siloxy^18^-, or ester^19,20^-functionalized polypentenamers, are of great industrial significance due to their distinct mechanical and thermal properties. However, the low ring strains of cyclopentene derivatives are intrinsic obstacles to polymerization; hence, controlling the thermal equilibrium is the key to achieving polymerization. As polymerization is an entropically disfavored reaction, low-temperature conditions are preferred, as they thermodynamically drive the reaction toward polymerization^9,12^. Unfortunately, low-temperature conditions are kinetically unfavorable for precatalyst initiation, which usually requires ligand dissociation. Therefore, a rapidly initiating catalyst that can be activated at low temperatures would be ideal for facilitating the ROMP of challenging low-strained cyclopentene derivatives (Fig. 1a).

Ruthenium-based olefin-metathesis catalysts have widened the scope of olefin-metathesis reactions due to their ease of accessibility, functional group compatibilities, and stabilities^21–24^. One of the major issues in catalyst development is control of the precatalyst initiation rate^25–36^. Fast-initiating catalysts are especially useful for selective metathesis^25,30^, controlled living ROMP^26,35^, and ROMP of low-strained monomers^9,34^, whereas slow-initiating catalysts are beneficial for industrial molding process involving the ROMP of highly constrained monomers such as DCPD and orthogonal metathesis reactions^31–33,36^.

In 2002, Louie and Grubbs^37^ reported that electron-rich carbene-based Ru complexes can slowly initiate the catalysis of various metathesis reactions at relatively high temperatures. This study revealed that an *N*-vinylpyrrolidinone-based carbene can exist in a chelate complex through the dissociation of one phosphine ligand and the intramolecular coordination of its carbonyl group (Fig. 1b). However, the *N*-vinylpyrrolidinone-based Ru complex was not active in olefin-metathesis reactions under mild reaction conditions. Inspired by these reported complexes and the structural diversity of amides, we envisioned versatile, highly controllable metathesis catalysts based on a less-alkaline amide scaffold that can facilitate initiation. Sulfonamides, which are readily available and structurally diverse, were identified as the less-alkaline amides for realizing this hypothesis. The acidity (p*K*_a_) of the N‒H bond in an amide is in the 13–23 range, whereas that of a sulfonamide is 9–18, which indicates that electron density on the sulfonamide nitrogen atom is usually lower than that of an amide. Notably, the carbonyl oxygen is the preferred protonation position in an amide, whereas the nitrogen atom is generally protonated in a sulfonamide^38,39^. The p*K*_a_ values of the conjugate acids of amides and sulfonamides are around −1 and −6, respectively (Fig. 1b). The data also indicate that the oxygen atoms of sulfonamides are much less basic than those of amides. Therefore, we envisaged that *N*-vinylsulfonamide-based ruthenium complexes are more reactive in olefin-metathesis reactions as a result of weaker oxygen chelation compared with *N*-vinylamide derivatives.

Herein, ruthenium olefin-metathesis catalysts that exhibit controllable initiation rates by the simple modification of readily accessible and structurally diverse *N*-vinylsulfonamide ligands are presented. Among the developed catalysts, trifluoromethanesulfonamide-based catalysts exhibited exceptional activities in metathesis reactions, especially for the polymerization of low-strained cyclopentene derivatives (Fig. 1c).

## Results

### Preparation of Ru complexes

**Fig. 2:**
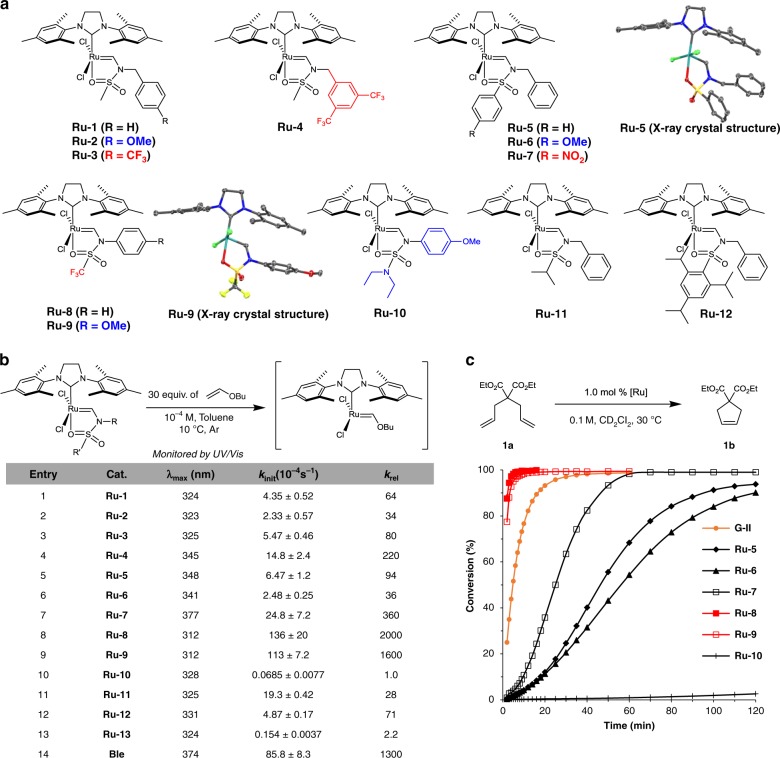
*N*-vinylsulfonamide-based metathesis catalysts and their catalytic activities. **a** Electronically and sterically diversified *N*-vinylsulfonamide-based metathesis catalysts. **b** Initiation rate constant for each complex determined by adding excess butyl vinyl ether to a solution of catalyst in toluene at 10 °C. **c** Standard RCM kinetics using electronically modified catalysts. The averages of three independent experiments are displayed

A variety of *N*-vinylsulfonamide ligands were readily obtained by the simple vinylation of versatile and synthetically or commercially readily available sulfonamides (Supplementary Figs. 1 and 2). Electronically and structurally diverse *N*-vinylsulfonamides were prepared and the corresponding catalysts were synthesized by ligand exchange reactions with **G-II** in the presence of CuCl as the phosphine scavenger (Supplementary Fig. 3). The complexes were firmly characterized by ^1^H, ^13^C, and ^19^F NMR spectroscopies, elemental analysis, and X-ray crystallography (Fig. 2a). Bench-top stability of the representative precatalysts, **Ru-1**, **Ru-5**, and **Ru-8**, were checked. After 1 month as a solid form in air, the colors of three complexes became darker, but their ^1^H NMR spectra were intact without observation of any other peak. However, when the catalysts were solubilized in dichloromethane and the solutions were exposed to air, the carbene peaks disappeared in an hour. With the developed complexes in hand, the initiation rate of each complex was measured by adding excess butyl vinyl ether (30 equiv.) at 10 °C and following the decay of the *λ*_max_ peak by UV/Vis spectroscopy at regular intervals (Fig. 2b and Supplementary Table 1)^27^.

### Examination of initiation rates

**Fig. 3:**
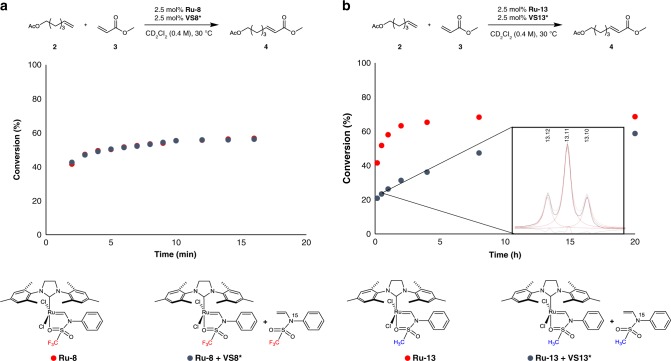
Effect of the external *N*-vinylsulfonamide ligand. **a** No difference in reactivity of **Ru-8** was observed regardless of the presence of ^15^N-labeled external VS8*. **b** The catalyst with the electron-donating group on the sulfonyl moiety exhibited a diminished reaction rate in the presence of ^15^N-labeled external VS13*. The carbene’s proton peak of **Ru-13** in ^1^H NMR spectra indicated the occurrence of ligand exchange (54:46 ratio of **Ru-13** (13.11 ppm, singlet) to Ru-13* (doublet)) from the initial stage of the reaction

At first, the initiation rate of the 2,2,2-trifluoro-*N*-phenyl-*N*-vinylacetamide-based ruthenium complex (**Ru-amide**), bearing a highly electron-withdrawing trifluoromethyl group was examined; however, no initiation was observed at 10 °C, which demonstrates that the amide-based catalyst is difficult to activate at low temperature (Fig. 1b). Confirming our initial hypothesis, the sulfonamide-based catalysts exhibited much improved initiation rates compared with the amide-based catalyst (Fig. 2b). Within the set of prepared sulfonamide-based catalysts, mesyl-based catalysts (**Ru-1** to **4**) with electronically modified benzyl groups were synthesized, to examine the effect of the electronic nature of the nitrogen atom. Among the mesyl-based catalysts, **Ru-2** bearing a methoxy group exhibited the lowest initiation rate constant (2.33 × 10^−4^ s^−1^, 10 °C), whereas **Ru-4** bearing a bis(trifluoromethyl) group exhibited the highest initiation rate constant (14.8 × 10^−4^ s^−1^) (Fig. 2b, entries 2 and 4). To determine the effect of the electronic nature of the sulfur atom, arenesulfonamide-based catalysts bearing arene groups with different electronic properties were next investigated (**Ru-5** to **7**). **Ru-7** with the *p*-nitro group exhibited a ten-times higher initiation rate constant (24.8 × 10^−^^4^ s^−1^) than that of the *p*-methoxy-substituted **Ru-6** (2.48 × 10^−4^ s^−1^) (Fig. 2b, entries 6 and 7). As the initiation rate constants changed more distinctively by varying the electronics of the sulfur atom rather than the nitrogen atom, varying the electronics of the functional group attached to the sulfur atom was chosen as the strategy for controlling the initiation rate. To our delight, the trifluoromethanesulfonamide-based catalysts **Ru-8** (136 × 10^−4^ s^−1^) and **Ru-9** (113 × 10^−4^ s^−1^) initiated very fast, with rate constants that even exceeded that of Blechert’s catalyst (**Ble**) (85.8 × 10^−4^ s^−1^), which is one of the fastest chelating-type catalysts reported to date (Fig. 3b, entries 8, 9, 13)^27,40^. On the other hand, complex **Ru-10** bearing the diethylamino group initiated slowly, with a 2000-times lower initiation rate constant (0.0685 × 10^−4^ s^−1^) compared with that of **Ru-8** (Fig. 2b, entry 10). Increasing the steric hindrance on the sulfur atom was observed to decrease the rate of initiation (**Ru-11**, 1.93 × 10^−4^ s^−1^; **Ru-12**, 4.87 × 10^−4^ s^−1^) (Fig. 2b, entries 11 and 12).

### Ring-closing metathesis

The developed catalysts were further examined in the RCM of diethyl diallylmalonate (**1a**) under the standard conditions reported by Grubbs and colleagues^41^ (Fig. 2c); these catalysts exhibited dramatically improved reactivities compared with those previously reported for Fischer-type carbene-based ruthenium complexes (Supplementary Figs. 4, 5, and 6)^37^. Complexes **Ru-8** and **Ru-9** exhibited the fastest initiation rates among the developed catalysts and showed even higher activities compared with that of **G-II**. **Ru-10** gave only 2% of the desired product at 30 °C after 2 h. When the temperature was elevated to 80 °C (C_6_D_6_), the RCM of **1a** was completed by **Ru-10** within 10 min (Supplementary Fig. 7). Other catalysts also exhibited predictable reactivities based on the electronic characteristics of their ligands.

A variety of standard olefin-metathesis reactions^41^ were compared and evaluated using complex **Ru-9**, which has similar RCM reactivity to that of **Ru-8** but is expected to be more stable for challenging metathesis reactions (Supplementary Table 2). First, the RCM activity of complex **Ru-9** was further investigated with a variety of dienes; it was faster in the chosen RCM reaction than **G-II**, which is the most popular catalyst for a variety of metathesis reactions. Even the sterically hindered trisubstituted olefin **S1** reacted very fast to give a 95% conversion in 10 min and a 97% final yield after 1 h. Under the same conditions, **G-III** provided a conversion of <50% in 10 min and an 80% yield after 70 min (Supplementary Table 2, entry 1). Further RCM reactivity was monitored using **G-III**, to compare initial conversions with those obtained using **Ru-9**. Based on the results obtained, **Ru-9** performed faster and more efficiently than **G-III** in the RCM of *N*,*N*-diallyl-4-methylbenzenesulfonamide **S2** (>99% vs. >85%) (Supplementary Table 2, entry 2). In the case of **G-III**, the reaction was observed to gradually slow down due to the instability of the methylidene species in the presence of pyridine, consistent with previous reports^42^.

### Mechanistic investigation

To evaluate the performance of **Ru-9**, the cross-metathesis (CM) of allylbenzene (**S7**) and 1,4-diacetoxy-2-butene (**S8**) was also examined. **Ru-9** promoted a consistently faster initial conversion than **G-II**. The *cis*/*trans*-isomerization equilibrium of the CM product was quickly achieved, even within 1 min (*E*/*Z* = ~9) (Supplementary Table 3). **Ru-9** produced the hetero-coupled product **4** (72%) more rapidly in the CM of 5-hexenyl acetate (**2**) with methyl acrylate (**3**), a standard chemoselective metathesis reaction used to check the catalytic performance with electron-deficient olefins, than **G-II** (66%) and **H-II** (66%) after 1 h (Supplementary Fig. 8)^43^.

We next investigated the mechanism of this reaction in detail, to determine whether or not the developed chelating catalysts operate through a boomerang mechanism. The release–return or boomerang mechanism is a long-standing issue in ruthenium-chelating metathesis catalysis^44–46^, which posits the reuptake of a released ligand by the active catalyst during the metathesis reactions to regenerate the precatalyst. The Hoveyda–Grubbs catalyst (**H-II**) with the chelating isopropoxystyrenyl ligand was reported by Grela and colleagues^45^ and by Fogg and colleagues^44^ to operate by the release–return mechanism. Based on the previous mechanistic studies, the CMs of **2** and **3** were catalyzed by the electronically modified sulfonamide-based catalysts **Ru-8** and **Ru-13** in the presence of additional ^15^N-labeled 1,1,1-trifluoro-*N*-phenyl-*N*-vinylmethanesulfonamide (VS8*) and *N*-phenyl-*N*-vinylmethanesulfonamide (VS13*), respectively (Fig. 3a, b). The reaction rate constant of **Ru-8** was not affected when an equimolar amount of VS8* was added, indicating a lack of reuptake (Fig. 3a)^47^. Negligible ligand exchange between **VS8** and VS8* was observed, and only a trace amount of carbene peak was observed in the ^1^H NMR spectrum. On the other hand, the reaction of **Ru-13** was clearly retarded in the presence of VS13*. In addition, ligand exchange between **VS13** and VS13* was detected by ^1^H NMR spectroscopy, which implies that the release–return mechanism operates in this case. Further background ligand exchanges in the absence of a substrate were negligible in both cases, which indicate that ligand-reuptake only occurs via the active methylidene species. These data indicate that the release–return mechanism is highly dependent on the electronic nature of the sulfonamide ligand.

### Ring-opening metathesis polymerization

**Fig. 4:**
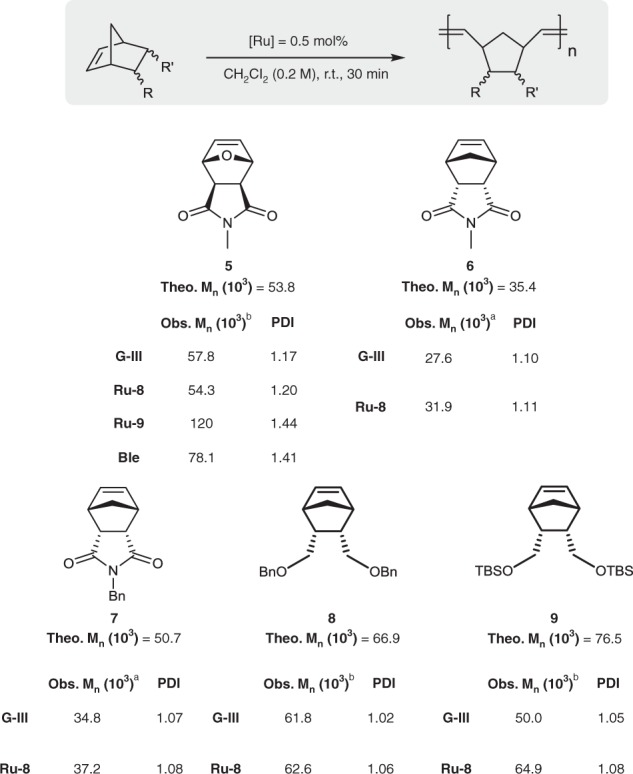
ROMPs of strained monomers. After quenching with ethyl vinyl ether, the polymer was isolated by precipitation in methanol. ^a^Determined by GPC in tetrahydrofuran (THF) relative to a polystyrene standard. ^b^Determined by GPC in *N*,*N*-dimethylformamide (DMF) relative to a polystyrene standard. ^c^Theoretical *M*_n_ was calculated by assuming quantitative conversion. ^d^Bn = Benzyl; TBS = *tert*-Butyldimethylsilyl

The application of the fastest-initiating **Ru-8** for typical ROMPs of strained monomers was next examined. It is well known that fast precatalyst initiation is important for controlling the living polymerization of strained monomers^26,35^. For the ROMPs of *endo*-norbornene and *exo-*7-oxonorbornene derivatives, **Ru-8** afforded products with similar narrow polydispersity indexes (PDIs) to those obtained with **G-III**, including *exo-*7-oxonorbornene derivative (**5**), which indicates that the initiation of **Ru-8** is sufficiently fast for use in controlled living polymerizations (Fig. 4). In the ROMP of **5**, both **Ru-9** and **Ble** gave the desired polymer with broad PDIs, which is likely due to insufficient initiation, resulting in a much higher molecular weight than that of the polymer obtained using initiator **Ru-8** or **G-III**. All the *endo*-norbornene derivatives also exhibited narrow PDIs.

**Fig. 5:**
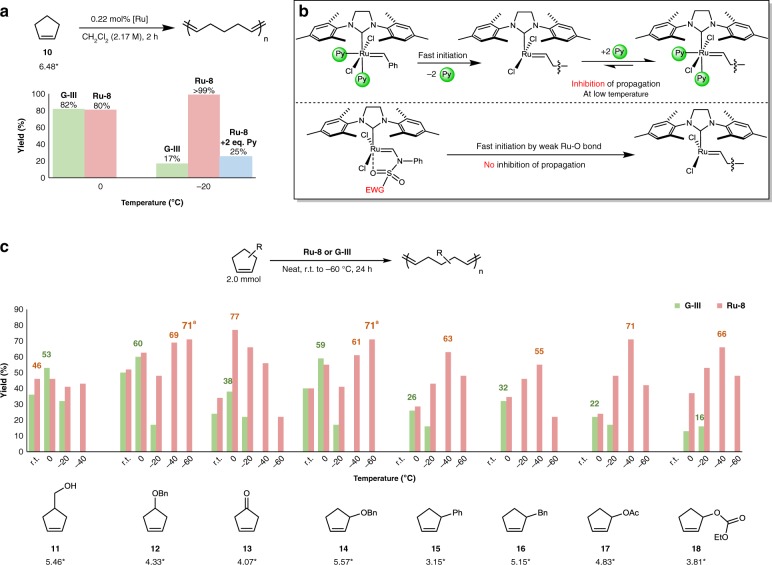
ROMP of cyclopentene derivatives at low temperatures. **a** Comparing **G-III** and **Ru-8** in the ROMP of cyclopentene at various temperatures. **b** Outlining pyridine interruption during propagation at low temperature. **c** ROMPs of functionalized cyclopentenes. After quenching with ethyl vinyl ether, the polymer was isolated by precipitation in methanol or hexane. ^a^In dichloromethane (2.0 M). ^*^Ring strain (kcal/mol) calculated at the G3//RB3LYP/6–31 + + G(2df,2p) level of theory

For unique application of highly active **Ru-8**, we turned our attention to the polymerization of challenging low-strained monomers. To investigate whether or not **Ru-8** is active at low temperatures, the ROMP of cyclopentene (**10**) was performed at various temperatures (Fig. 5a and Supplementary Table 9). The equilibrium conversion of monomer to polymer through ROMP of **10** has been reported to increase with decreasing temperature on the basis of thermodynamic considerations^12^. At 0 °C, both **G-III** and **Ru-8** catalyzed only moderate yields (>80%) (Fig. 5a). However, only **Ru-8** maintained its reactivity at −20 °C (>99% yield) and below, whereas the ROMP activity of **G-III** significantly decreased (17% yield) below −20 °C. Inspired by this observation, the deleterious effect of pyridine on the propagating species at low temperature was examined (Fig. 5b); when pyridine was used as an additive with **Ru-8**, a lower yield was obtained (25%). Stabilization of the propagating Ru carbene intermediates during ROMP by coordinating an additional ligand such as pyridine, phosphine, and an olefin has been reported with observation of the arrested intermediates^48,49^.

**Ru-8** was then applied to the ROMP of functionalized cyclopentenes, which have even lower strain energies than cyclopentene, some of which have never been used in ROMP (Supplementary Tables 10–17). Recently, the Moore group reported the reversible polymerization of 4-substituted cyclopentenes for the interconversion of a polymer and a neat monomer through control of the reaction temperature^50^. Although the ROMPs of 4-substituted cyclopentenes have been reported with **G-II** and **G-III**, 3-substituted cyclopentenes, which have even lower ring strains than the 4-substituted derivatives, are still challenging with the previously reported catalysts^9,17,20^. Expectedly, the polymerizations of 4-substituted monomers **11** and **12** with **G-III** and **Ru-8** exhibited similar moderate yields (36% and 46% for **11**, and 50% and 52% for **12**, respectively, Fig. 5c) at room temperature, and higher yields were not observed at low temperatures. This observation is probably due to physical limitations, such as initiator solubility or rapid gelation due to moderate conversions of the reaction mixtures, even at room temperature, resulting in entropic factors that further hinder these polymerization reactions.

We were clearly able to confirm the temperature effect during the ROMP of cyclopent-3-en-1-one (**13**). A dramatic increase in yield was observed when the temperature was decreased to 0 °C (from 33% at room temperature to 77% at 0 °C). However, lower yields were observed when the temperature was further decreased, which is probably due to the lower solubility of the produced polymer. In the case of the (cyclopen-2-en-1-yloxy)methylbenzene monomer (**14**), the obtained polymer produced under neat conditions formed a soft gel after a few minutes, with loss of solubility. Meanwhile, other than **14**, which showed a slightly higher ring strain compared with the other monomers, the challenging 3-substituted monomers (**15**–**18**) showed no conversion at room temperature with either **G-III** or **Ru-8**. When the temperature was decreased to 0 °C, similar but low yields were observed using both catalysts. The yield of the desired polymer formed by **G-III** was further reduced, as the temperature was further decreased to −20 °C. These data are in good agreement with the ROMP results of cyclopentene, namely the inhibiting effect of free 3-bromopyridine. To our delight, higher yields of polymer were obtained in all examples catalyzed by **Ru-8** at the same temperature (−20 °C). **G-III** exhibited no activity when the reaction temperature was further decreased to −40 °C, whereas **Ru-8** showed the highest yield in all examples (Fig. 5c). However, due to solubility limitations or the low freezing points of some monomers, the overall yields of the desired polymers at −60 °C were somewhat lower compared with those obtained at −40 °C. Among the obtained polymers, **poly-18** has good regio-regularity as only two peaks were observed at 133.7 and 128.3 ppm in ^13^C NMR spectra. Further two-dimensional NMR experiments revealed that head-to-tail and head-to-head isomers were present at a ratio of 86:14, respectively, as dominant *trans*-configuration (Supplementary Fig. 10). It has been reported that ROMP of allylic-substituted cyclopentene monomers with high steric bulk can drive highly regular microstructure of major *trans*-olefins with head-to-tail insertion^51^. It is noteworthy that the challenging monomers with low ring strains successfully underwent ROMP with the very fast **Ru-8** initiator. To the best of our knowledge, no ROMP example of monomers **14**–**18** has been previously reported.

### ROMP of cyclopentadiene

**Fig. 6:**
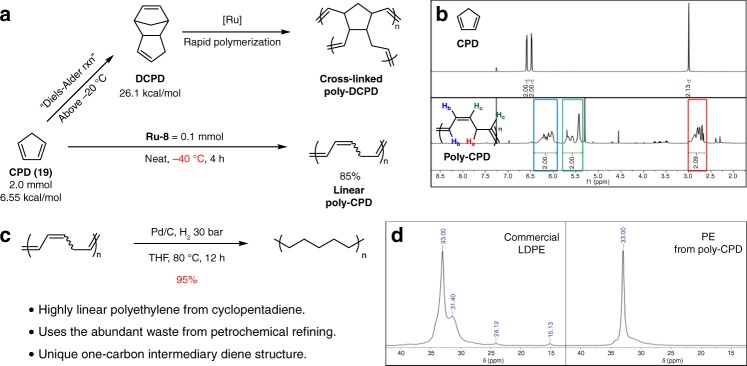
ROMP of cyclopentadiene. **a** Dimerization of CPD at ambient temperature. **b** ROMP of CPD at −40 °C with **Ru-8**. Conversion was calculated from the ^1^H NMR spectrum. No reaction was observed with **G-II** or **G-III** under same conditions. **c** Post-treatment of **poly-CPD**. Without purification, the crude **poly-CPD** was directly dissolved in THF and hydrogenated in quantitative yields. **d** Comparing polyethylene samples by solid-state ^13^ C NMR spectroscopy; commercially available low-density polyethylene (left), polyethylene via hydrogenation of **poly-CPD** (right)

For further application of **Ru-8**, the ROMP of CPD (**19**) was investigated. CPD is a useful monomer and precursor for a variety of reactions, including anionic or thermal polymerizations, but is not a suitable monomer for ROMP due to its low ring strain (6.55 kcal/mol) and fast dimerization at ambient temperature (Fig. 6a). Even worse, DCPD from the dimerization of CPD has exceptionally high ring strain (26.1 kcal/mol), which results in the rapid formation of a cross-linked polymer network. Due to these characteristics, the ROMP of CPD has, to the best of our knowledge, not been demonstrated. To prevent the formation of DCPD, the reaction temperature needs to be sufficiently low, because the dimerization occurs slowly even at −20 °C^52^. Therefore, to obtain a linear polymer from CPD by ROMP, the reaction needs to be conducted at −40 °C (Fig. 6a). Among the reactions attempted, only **Ru-8** demonstrated activity for the polymerization of CPD (up to 85% yield over 4 h) (Fig. 6a and Supplementary Table 18). The obtained polymer was carefully characterized by ^1^H and ^1^H-^1^H COSY NMR spectroscopy, which exhibited data that are well matched to those of the expected polymer structure (Fig. 6b and Supplementary Fig. 11). Atmospheric pressure chemical ionization mass spectroscopy of CPD oligomers, synthesized from a lowered equivalent of monomer ([CPD]:[**Ru-8**] = 5:1), also confirmed the structure with clear observation of the C_5_H_6_ repeating unit (66 Da, Supplementary Fig. 12). The crude reaction mixture was then dissolved in THF and directly hydrogenated (30 bar of H_2_ pressure) to afford polyethylene (81% over two steps), the linearity of which was determined by solid-state ^13^C NMR spectroscopy (Fig. 6c)^52,53^. The NMR spectrum exhibited a single major peak at ~33 ppm (Fig. 6d and Supplementary Fig. 13), indicating the formation of highly linear polyethylene, which also verifies that DCPD was not majorly involved in the polymerization process^54^.

## Discussion

*N*-vinylsulfonamide ligand-based ruthenium olefin-metathesis catalysts were developed. On the basis of this versatile scaffold, more than ten catalysts were prepared. These catalysts exhibited initiation rates that were easily controlled in a straightforward manner by simply modifying their steric and electronic characteristics. In particular, ultrafast-initiating catalysts were obtained by introducing the electron-withdrawing trifluoromethyl group on the sulfonyl moiety. The weakened Ru–O bond enables rapid initiation along with fast propagation, even at low temperatures, without the intervention of a bound-back ligand, such as pyridine. Using the trifluoromethanesulfonamide-based catalysts, the enthalpically challenging ROMP of low-strained monomers was successfully demonstrated by driving the thermodynamics toward the polymer by decreasing the reaction temperature and minimizing the effect of entropy. To our surprise, these catalysts enabled the ROMP of CPD, an easily obtainable common feedstock from the steam cracking of naphtha, which is challenging due to its low ring strain and fast dimerization to DCPD even at 0 °C. The synthesized polymer exhibited an extraordinary linear diene structure, which is difficult to produce using other monomers and polymerization techniques. The one-carbon intermediary diene structure can be further functionalized to generate advanced materials. The developed sulfonamide-based catalysts that operate with a wide range of initiation rates are expected to be applicable to a variety of industrial applications of involving olefin-metathesis reactions that require controlled initiation rates at a specific temperature.

## Methods

### Preparation of complex Ru-8

The Grubbs second-generation catalyst (**G-II)** (425 mg, 0.5 mmol), CuCl (99.0 mg, 1.0 mmol), the appropriate *N*-vinylsulfonamide ligand (1.0 mmol), and dichloromethane (5 mL) were added under Ar to a flame-dried 25 mL Schlenk flask equipped with a Teflon-coated magnetic stirrer bar. The reaction mixture was stirred at room temperature for 30 min, during which time the color changed from dark red to dark orange. The volatiles were removed in vacuo to give a brown residue. The crude product was dissolved in excess diethyl ether (15 mL) to instantly afford CuCl∙PCy_3_ as a white precipitate. The suspension was filtered through a pad of cotton in a glass pipette to remove the CuCl∙PCy_3_ adduct. The filtrate was then diluted with pentane (15 mL) in a flame-dried 50 mL vial. The mixture was stored in a −20 °C fridge in a glovebox for 1 day. The desired orange crystalline solid was easily isolated by washing with excess pentane.

## Supplementary information

Supplementary Information

## Data Availability

Crystallographic data for the structures reported in this article (Supplementary Figs. 79–82) have been deposited at the Cambridge Crystallographic Data Center, under deposition nos. CCDC 1526992 (**Ru-5**), CCDC 1526991 (**Ru-6**), CCDC 1526994 (**Ru-7**), and CCDC 1526990 (**Ru-9**). Copies of the data can be obtained free of charge from www.ccdc.cam.ac.uk/structure/. For calculation procedures for the ring strains of monomers **11**–**19** and **DCPD**, see Supplementary Tables 4–8. For confirmation of regio-regularity of **poly-17** and **poly-18**, see Supplementary Figs. 9 and 10. For NMR analysis of the compounds in this article, see Supplementary Figs. 14–78. All other relevant data of this study are available within the manuscript and its Supplementary Information, and/or from the corresponding authors upon reasonable request.
