# Supplementary material for: Highly active ruthenium metathesis catalysts enabling ring-opening metathesis polymerization of cyclopentadiene at low temperatures
Source: Nat Commun. 2019 Aug 27;10:3860. doi: 10.1038/s41467-019-11806-5 (PMC6712042; doi:10.1038/s41467-019-11806-5)
Supplement: Supplementary file 1 — Supplementary Information [file 41467_2019_11806_MOESM1_ESM.pdf]

Highly Active Ruthenium Metathesis Catalysts Enabling Ring-Opening Metathesis Polymerization of Cyclopentadiene at Low Temperatures

Kitaek Song[1,2], Kunsoon Kim[1,2], Daeun Hong[2], Jungwon Kim[1,2], Chae Eun Heo[3], Hugh I. Kim[3] and Soon Hyeok Hong[1,2]*

[1]Department of Chemistry, Korea Advanced Institute of Science and Technology (KAIST), Daejeon 34141, Republic of Korea

[2]Department of Chemistry, College of Natural Sciences, Seoul National University, Seoul 08826, Republic of Korea

[3]Department of Chemistry, Korea University, Seoul 02841, Republic of Korea

Supplementary Methods

General considerations: Unless otherwise noted, all reactions were carried out using standard Schlenk techniques or in an argon-filled glove box. Dichloromethane, pentane and benzene were dried using a Pure Solv solvent purification system. Deuterated solvents were purchased from Cambridge Isotope Laboratories or Sigma-Aldrich. NMR spectra were recorded in $CDCl_3$, CD_2Cl_2 or C_6D_6 on a Bruker DPX300, Varian 400, or 500 MHz spectrometer. NMR sepctra were processed with MestReNova software. Chemical shifts are reported in ppm and coupling constant in Hz. High resolution mass spectrometry (HRMS) was performed by the Sogang Center for Research Facilities. Gas chromatography (GC) analysis was performed with an Agilent Technologies 7980A GC system equipped with a DB-Wax polyethylene glycol capillary column and an FID detector. Elemental analyses were conducted at the National Center for Inter-University Research Facilities of Seoul National University (NCIRF) and in the Sogang Center for Research Facilities using Thermo Scientific Flash 2000 elemental analyzer and a Thermo Finnigan Flash EA 1112, respectively. Single crystal X-ray crystallography was conducted at the Center for Research Facilities in the Research Institute of Pharmaceutical Sciences of Seoul National University, using an Agilent SuperNova X-ray diffractometer. Advanced Polymer Chromatography (APC) for polymer molecular weight analysis was carried out with Waters System (Isocratic Solvent Manger, Sample Manager w/ FTN, Refractive Index detector) and a set of three ACQUITY APC^{TM} XT (45, 200, 450) columns eluted with Tetrahydrofuran (HPLC grade, J. T. Baker). The flow rate was 1.0 mL/min, and the temperature of the column was maintained at 35 ^{o}C. Samples were diluted in 0.001-0.003 wt% by THF and filtered through a 0.20 μm PTFE filter before injection into APC. The data were analyzed using Empower 3 software. High-resolution mass spectrometry (HRMS) was performed in the Sogang Center for Research Facilities and the Korea Basic Science Institute (KBSI) using ESI, EI and FAB methods. The ^{13}C NMR solid-state, CPMAS NMR measurements of the PE samples were conducted at the National Center for Inter-University Research Facilities of Seoul National University (NCIRF) using Bruker Advance III HD Spectrometers operating 11.8 T (125.8 MHz). The samples were packed in 2.5 mm rotors and rotated at MAS rates of 20 kHz. MS experiments for analyzing CPD Oligomer were performed using a Bruker Compact quadrupole time-of-flight (q-TOF) mass spectrometer with an atmosphere pressure chemical ionization (APCI) source. The Grubbs second-generation catalyst was purchased from Sigma-Aldrich. CD_2Cl_2 was distilled from CaH_2 and freeze/pump/thawed three times. Other chemicals were purchased from commercial suppliers and filtered through pads of silica prior to use.

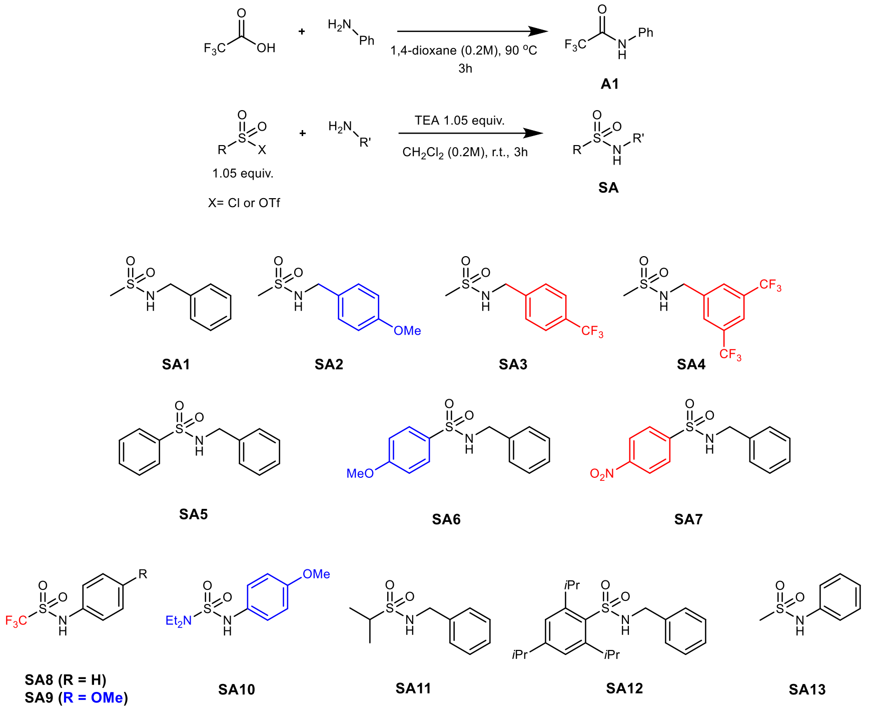

Supplementary Figure 1. Preparation of the amide and sulfonamides

Preparation of the amide and sulfonamides: Amide **A1** was synthesized in quantitative yield following a literature procedure.[1] Sulfonamides **SA1** to **SA7** and **SA11** to **SA13**,[2] **SA8** and **SA9**,[3] and **SA10**[4,5] were synthesized following literature procedures in good-to-excellent yields. The synthesized amide and sulfonamides were directly vinylated following the procedure shown in **Supplementary Figure 2**.

2,2,2-Trifluoro-*N*-phenylacetamide (A1)

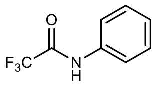

White solid; ^{1}H NMR (300 MHz, $CDCl_3$): δ = 7.87 (brs, 1H), 7.55 (dd, J = 7.5, 1.2 Hz, 2H), 7.38 (dd, J = 11.0, 4.9 Hz, 2H), 7.24 (ddd, J = 7.5, 3.8, 1.8 Hz, 1H). The compound was identified by spectral comparison with literature data.[1]

N-benzylmethanesulfonamide (SA1)

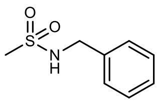

White solid; ^{1}H NMR (300 MHz, $CDCl_3$): δ = 7.40-7.32 (m, 5H), 4.51 (s, 1H), 4.31 (d, J = 6.1 Hz, 2H), 2.88 (s, 3H). The compound was identified by spectral comparison with literature data.[6]

N-(4-methoxybenzyl)methanesulfonamide (SA2)

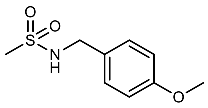

White solid; ^{1}H NMR (400 MHz, $CDCl_3$): δ = 7.23 (s, 2H), 6.85 (s, 2H), 5.07 (s, 1H), 4.19 (s, 2H), 3.77 (s, 3H), 2.78 (s, 3H). The compound was identified by spectral comparison with literature data.[7]

N-[4-(trifluoromethyl)benzyl]methanesulfonamide (SA3)

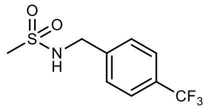

White solid; ^{1}H NMR (400 MHz, $CDCl_3$): δ = 7.61 (d, J = 7.3 Hz, 2H), 7.47 (d, J = 6.9 Hz, 2H), 5.20 (s, 1H), 4.35 (s, 2H), 2.88 (s, 3H); ^{13}C NMR (100 MHz, $CDCl_3$): δ = 141.0, 130.4 (q, J = 32.3 Hz), 128.2, 125.9 (q, J = 3.7 Hz), 124.0 (q, J = 272.5 Hz), 46.6, 41.2; ^{19}F NMR (376 MHz, $CDCl_3$) δ = -62.64; HRMS-ESI (m/z) $[M + Na]^+$ calcd. for $C_9H_{10}F_3NNaO_2S$, 276.0277; found, 276.0279.

N-[3,5-bis(trifluoromethyl)benzyl]methanesulfonamide (SA4)

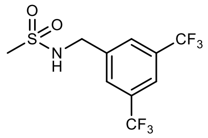

White solid; ^{1}H NMR (500 MHz, $CDCl_3$): δ = 7.83 (s, 3H), 4.99 (brs, 1H), 4.46 (d, J = 6.3 Hz, 2H), 2.98 (s, 3H); ^{13}C NMR (75 MHz, $CDCl_3$): δ = 139.8, 132.3 (q, J = 33.6 Hz), 128.0, 123.2 (q, J = 271.8 Hz), 122.1 (dt, J =7.7, 3.9 Hz), 46.2, 41.2; ^{19}F NMR (376 MHz, $CDCl_3$) δ = -62.99; HRMS-ESI (m/z) $[M + Na]^+$ calcd. for $C_{10}H_9F_6NNaO_2S$, 344.0150; found, 344.0151.

N-benzylbenzenesulfonamide (SA5)

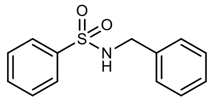

White solid; ^{1}H NMR (500 MHz, $CDCl_3$): δ = 7.89-7.83 (m, 2H), 7.58 (t, J = 7.4 Hz, 1H), 7.50 (t, J = 7.7 Hz, 2H), 7.29-7.22 (m, 3H), 7.20-7.15 (m, 2H), 4.94 (s, 1H), 4.14 (s, 2H). The compound was identified by spectral comparison with literature data.[6]

N-benzyl-4-methoxybenzenesulfonamide (SA6)

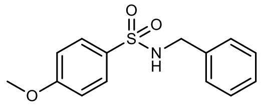

White solid; ^{1}H NMR (500 MHz, $CDCl_3$): δ = 7.85-7.78 (m, 2H), 7.30-7.23 (m, 3H), 7.22-7.17 (m, 2H), 7.00-6.94 (m, 2H), 4.68 (s, 1H), 4.11 (s, 2H), 3.88 (s, 3H). The compound was identified by spectral comparison with literature data.[6]

N-benzyl-4-nitrobenzenesulfonamide (SA7)

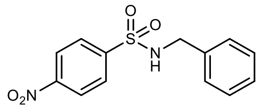

White solid; ^{1}H NMR (500 MHz, $CDCl_3$): δ = 8.32-8.26 (m, 2H), 8.01-7.94 (m, 2H), 7.25 (dd, J = 6.5, 4.0 Hz), 7.16 (dd, J = 6.5, 2.7 Hz, 2H), 5.14 (t, J = 5.9 Hz, 1H), 4.22 (d, J = 6.1 Hz, 2H). The compound was identified by spectral comparison with literature data.[6]

1,1,1-Trifluoro-N-phenylmethanesulfonamide (SA8)

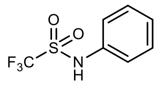

White solid; ^{1}H NMR (500 MHz, $CDCl_3$): δ = 7.60-7.20 (m, 6H). The compound was identified by spectral comparison with literature data.[8]

1,1,1-Trifluoro-N-(4-methoxyphenyl)methanesulfonamide (SA9)

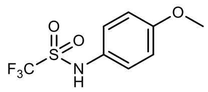

White solid; ^{1}H NMR (400 MHz, $CDCl_3$): δ = 7.21 (dd, J = 14.3, 11.1 Hz, 2H), 6.89 (dd, J = 16.5, 5.9 Hz, 2H), 3.78 (s, 3H). The compound was identified by spectral comparison with literature data.[8]

***N*,*N*-diethyl-*N*'-(4-methoxyphenyl)sulfonamide** (**SA10**)

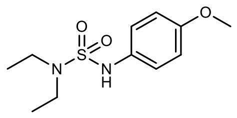

Red solid; ^{1}H NMR (300 MHz, $CDCl_3$): δ = 7.16-7.11 (m, 2H), 6.85-6.80 (m, 3H), 3.77 (s, 3H), 3.23 (q, J = 7.1 Hz, 4H), 1.04 (t, J = 7.1 Hz, 6H); ^{13}C NMR (75 MHz, $CDCl_3$): δ = 157.3, 130.1, 124.0, 114.5, 55.6, 42.4, 13.9; HRMS-EI (m/z) $[M]^+$ calcd. for $C_{11}H_{18}N_2O_3S$, 258.1038; found, 258.1040.

***N*-benzylpropane-2-sulfonamide** (**SA11**)

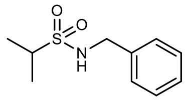

White solid; ^{1}H NMR (300 MHz, $CDCl_3$): δ = 7.26-7.15 (m, 5H), 4.85 (s, 1H), 4.19 (d, J = 5.9 Hz, 2H), 2.95 (hept, J = 6.8 Hz, 1H), 1.22 (d, J = 6.8 Hz, 6H). The compound was identified by spectral comparison with literature data.[9]

***N*-benzyl-2,4,6-triisopropylbenzenesulfonamide** (**SA12**)

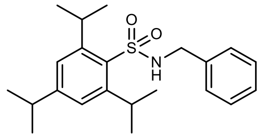

White solid; ^{1}H NMR (500 MHz, $CDCl_3$): δ = 7.31-7.15 (m, 7H), 4.51 (s, 1H), 4.24-4.08 (m, 4H), 2.92 (dp, J = 13.6, 6.8 Hz, 1H), 1.26 (t, J = 6.9 Hz, 18H). The compound was identified by spectral comparison with literature data.[10]

***N*-phenylmethanesulfonamide** (**SA13**)

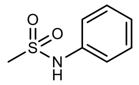

White solid; ^{1}H NMR (300 MHz, $CDCl_3$): δ = 7.32-7.27 (m, 2H), 7.19-7.13 (m, 3H), 6.44 (s, 1H), 2.94 (s, 3H). The compound was identified by spectral comparison with literature data.[7]

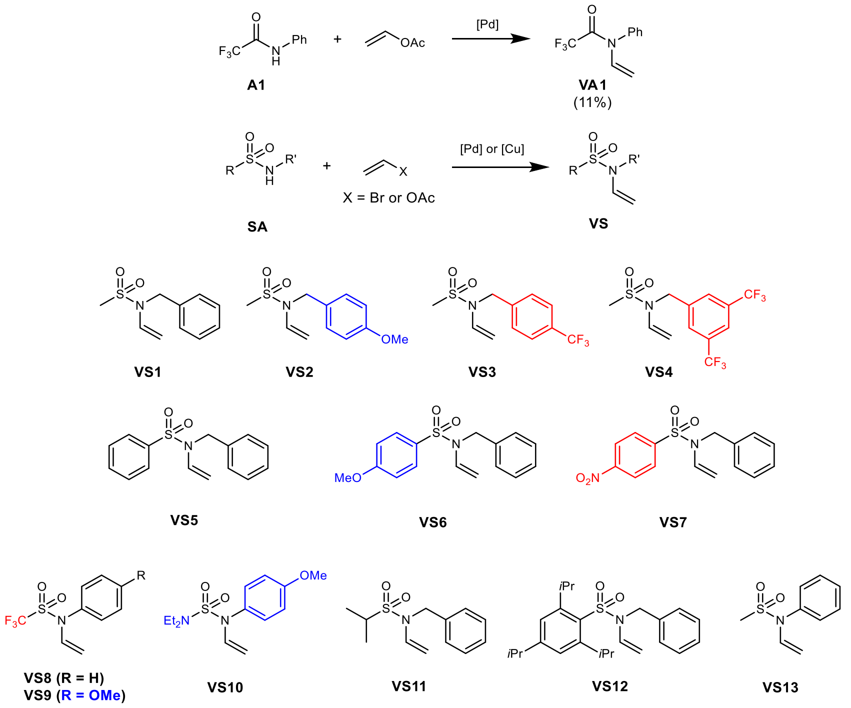

Supplementary Figure 2. Preparation of the *N*-vinylamide and sulfonamides

Preparation of the *N*-vinylsulfonamides: *N*-vinylsulfonamides **VS1–7**, **VS11**, **and VS13** were synthesized following the procedure of Buchwald,[11] while **VA1**, **VS8**, **VS9**, **VS10**, **and VS13** were prepared by the method of Guo.[12] The synthesized *N*-vinylsulfonamides were directly metallated following the procedure shown in **Supplementary Figure 3**.

2,2,2-Trifluoro-*N*-phenyl-*N*-vinylacetamide (VA1)

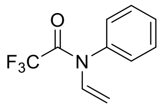

Yellow liquid, 11%; ^{1}H NMR (300 MHz, $CDCl_3$) δ = 7.73-7.56 (m, 1H), 7.49 (m, 3H), 7.26 (m, 2H), 4.71 (d, J = 8.2 Hz, 1H), 4.14 (d, J = 15.7 Hz, 1H); ^{13}C NMR (75 MHz, $CDCl_3$) δ =154.0 (q, J = 36.4 Hz), 132.6, 128.7, 128.5, 128.4, 115.1 (q, J = 287.5 Hz), 100.6, 28.7; ^{19}F NMR (376 MHz, $CDCl_3$) δ = -67.82; HRMS-EI (m/z) $[M]^+$ calcd. for $C_{10}H_8F_3NO$, 215.0558; found, 215.0558.

N-benzyl-*N*-vinylmethanesulfonamide (VS1)

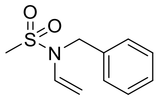

White solid, 74%; ^{1}H NMR (300 MHz, $CDCl_3$) δ = 7.27-7.17 (m, 5H), 6.74 (dd, J = 9.3, 15.6 Hz, 1H), 4.63, (s, 2H), 4.26 (dd, J = 1.5, 9.3 Hz, 1H), 4.24 (dd, J = 1.5, 15.6 Hz, 1H), 2.81 (s, 3H) ; ^{13}C NMR (75 MHz, $CDCl_3$) δ = 135.6, 131.9, 128.8, 127.7, 127.1, 94.4, 48.7, 39.7 ; HRMS−ESI (m/z) $[M + Na]^+$ calcd. for $C_{10}H_{13}NNaO_2S$, 234.0559; found, 234.0559.

N-(4-methoxybenzyl)-N-vinylmethanesulfonamide (**VS2**)

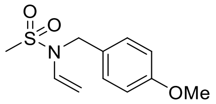

White solid, 87%; ¹H NMR (300 MHz, $CDCl_3$) δ = 7.18 (d, J = 8.6 Hz, 2H), 6.77 (d, J = 8.6 Hz, 2H), 6.69 (dd, J = 9.5, 15.4 Hz, 1H), 4.54 (s, 2H), 4.26 (dd, J = 1.4, 15.6 Hz, 1H), 4.25 (dd, J = 1.5, 9.4 Hz, 1H), 3.68 (s, 3H), 2.76 (s, 3H); ^{13}C NMR (75 MHz, $CDCl_3$) δ = 159.1, 131.9, 128.5, 127.5, 114.1, 94.2, 55.2, 48.1, 39.5; HRMS−ESI (m/z) $[M + Na]^+$ calcd. for $C_{11}H_{15}NNaO_3S$, 264.0665; found, 264.0668.

N-[4-(trifluoromethyl)benzyl]-N-vinylmethanesulfonamide (**VS3**)

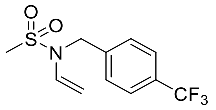

Colorless oil, 68%; ¹H NMR (300 MHz, $CDCl_3$) δ = 7.60 (d, J = 8.1 Hz, 2H), 7.45 (d, J = 8.1 Hz, 2H), 6.82 (dd, J = 9.5, 15.8 Hz, 1H), 4.75 (s, 2H), 4.36 (dd, J = 1.7, 9.3 Hz, 1H), 4.23 (dd, J = 1.7, 15.7 Hz, 1H), 2.95 (s, 3H); ^{13}C NMR (75 MHz, $CDCl_3$) δ = 139.8, 131.6, 130.0 (q, J = 32.4 Hz), 127.2, 125.8 (q, J = 3.8 Hz), 124.1 (q, J = 272.5 Hz), 94.8, 48.3, 39.5; ^{19}F NMR (376 MHz, $CDCl_3$) δ = -62.57; HRMS−ESI (m/z) $[M + Na]^+$ calcd. for $C_{11}H_{12}F_3NNaO_2S$, 302.0433; found, 302.0435.

N-[3,5-bis(trifluoromethyl)benzyl]-N-vinylmethanesulfonamide (**VS4**)

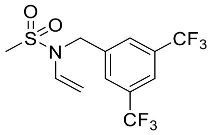

Yelow oil, 54%; ¹H NMR (400 MHz, $CDCl_3$) δ = 7.79 (s, 2H), 7.76 (s, 1H), 6.82 (dd, J = 9.3, 15.7 Hz, 1H), 4.80 (s, 2H), 4.33 (dd, J = 1.7, 9.2 Hz, 1H), 4.19 (dd, J = 1.7, 15.7 Hz, 1H), 2.96 (s, 3H); ^{13}C NMR (100 MHz, $CDCl_3$) δ = 138.9, 131.9 (q, J = 33.9 Hz), 131.4, 127.1 (q, J = 2.8 Hz), 123.2 (q, J = 272.7 Hz), 121.6 (m), 94.8, 47.7, 38.9; ^{19}F NMR (376 MHz, $CDCl_3$) δ = -62.96; HRMS−ESI (m/z) $[M + Na]^+$ calcd. for $C_{12}H_{11}F_6NNaO_2S$, 370.0433; found, 370.0305.

N-benzyl-N-vinylbenzenesulfonamide (**VS5**)

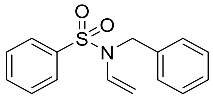

White solid, 53%; ¹H NMR (400 MHz, $CDCl_3$) δ = 7.79 (d, J = 7.5 Hz, 2H), 7.58 (t, J = 7.5 Hz, 1H), 7.50 (t, J = 7.5 Hz, 2H), 7.30-7.20 (m, 5H), 6.96 (dd, J = 9.6, 15.8 Hz, 1H), 4.54 (s, 2H), 4.27 (dd, J = 1.4, 9.3 Hz, 1H), 4.15 (dd, J = 1.2, 15.7 Hz, 1H); ^{13}C NMR (100 MHz, $CDCl_3$) δ = 139.2, 135.4, 133.1, 132.2, 129.4, 128.7, 127.6, 127.0, 127.0, 95.0, 48.9; HRMS−ESI (m/z) $[M + Na]^+$ calcd. for $C_{15}H_{15}NNaO_2S$, 296.0716; found, 296.0716.

N-benzyl-4-methoxy-N-vinylbenzenesulfonamide (**VS6**)

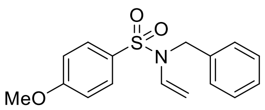

White solid, 37%; ¹H NMR (300 MHz, $CDCl_3$) δ = 7.67-7.62 (m, 2H), 7.23-7.08 (m, 5H), 6.92-6.83 (m, 3H), 4.43 (s, 2H), 4.16 (dd, J = 1.4, 9.3 Hz, 1H), 4.05 (dd, J = 1.5, 15.7 Hz, 1H), 3.76 (s, 3H); ^{13}C NMR (75 MHz, $CDCl_3$) δ = 163.2, 135.6, 132.3, 130.7, 129.1, 128.6, 127.4, 126.9, 114.5, 94.6, 55.7, 48.8; HRMS−ESI (m/z) $[M + Na]^+$ calcd. for $C_{16}H_{17}NNaO_3S$, 326.0821; found, 326.0822.

N-benzyl-4-nitro-N-vinylbenzenesulfonamide (**VS7**)

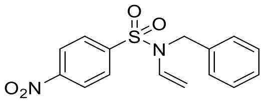

White solid, 44%; ^{1}H NMR (500 MHz, $CDCl_3$) δ = 8.34-8.32 (m, 2H), 7.95-7.93 (m, 2H), 7.31-7.23 (m, 5H), 4.51 (s, 2H), 4.43 (dd, J = 1.8, 9.2 Hz, 1H), 4.33 (dd, J = 1.7, 15.7 Hz, 1H). The compound was identified by spectral comparison with literature data.[13]

_N_-vinyl-_N_-phenyltrifluoromethanesulfonamide (VS8)

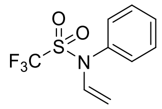

Colorless oil, >99%; ^{1}H NMR (500 MHz, $CDCl_3$) δ = 7.53-7.47 (m, 3H), 7.36-7.31 (m, 2H), 7.00 (dd, J = 15.3, 8.8 Hz, 1H), 4.57 (dd, J = 8.8, 1.7 Hz, 1H), 4.13 (dd, J = 15.4, 1.6 Hz, 1H); ^{13}C NMR (125 MHz, $CDCl_3$) δ = 134.0, 133.6, 130.1, 130.0, 129.9, 120.1 (q, J= 325.0 Hz), 98.7; ^{19}F NMR (376 MHz, $CDCl_3$) δ = -73.82; HRMS−ESI (m/z) [M + Na]$^+$ calcd. for $C_9H_8F_3NNaO_3S$, 274.0120; found, 274.0123.

_N_-vinyl-_N_-(4-methoxyphenyl)trifluoromethanesulfonamide (VS9)

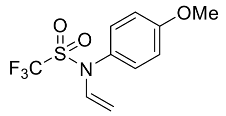

White solid, >99%; ^{1}H NMR (500 MHz, $CDCl_3$) δ = 7.20 (d, J = 8.8 Hz, 2H), 7.05-6.95 (m, 3H), 4.56 (d, J = 8.3 Hz, 1H), 4.17 (d, J = 15.2 Hz, 1H), 3.82 (s, 3H); ^{13}C NMR (125 MHz, $CDCl_3$) δ = 160.6, 133.8, 131.1, 126.1, 120.1 (q, J = 324.1 Hz), 115.1, 98.4, 55.4; ^{19}F NMR (376 MHz, $CDCl_3$) δ = -73.72; HRMS−ESI (m/z) [M + Na]$^+$ calcd. for $C_{10}H_{10}F_3NNaO_3S$, 304.0226; found, 304.0227.

_N,N_-diethyl-_N_'-(4-methoxyphenyl)-_N_'-vinylsulfamide (VS10)

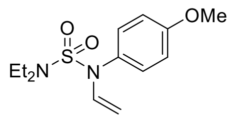

Yellow oil, 82%; ^{1}H NMR (300 MHz, C_6D_6) δ = 7.37 (dd, J = 8.9, 15.4 Hz, 1H), 7.28 (d, J = 8.2 Hz, 2H), 6.88 (d, J = 8.2 Hz, 2H), 4.14 (d, J = 9.0 Hz, 1H), 3.88 (d, J = 15.4 Hz, 1H), 3.51 (s, 3H), 3.00 (q, J = 7.2 Hz, 4H), 0.94 (t, J = 7.1 Hz, 6H); ^{13}C NMR (75 MHz, C_6D_6) δ = 160.2, 137.5, 131.8, 129.3, 115.0, 90.7, 55.2, 43.4, 14.4; HRMS−FAB (m/z) [M + H]$^+$ calcd. for $C_{13}H_{20}N_2O_3S$, 285.1267; found, 285.1270.

_N_-benzyl-_N_-vinyl-isopropanesulfonamide (VS11)

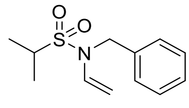

Brown solid, 41%; ^{1}H NMR (300 MHz, $CDCl_3$) δ = 7.25-7.11 (m, 5H), 6.68 (dd, J = 9.3, 15.5 Hz, 1H), 4.61 (s, 2H), 4.11 (s, 1H), 4.07 (d, J = 7.3 Hz, 1H), 3.14 (m, 1H), 1.25 (d, J = 6.9 Hz, 6H); ^{13}C NMR (75 MHz, $CDCl_3$) δ = 135.8, 132.6, 128.5, 127.4, 126.8, 92.7, 54.7, 49.2, 16.5; HRMS-ESI (m/z) [M + H]$^+$ calcd. for $C_{12}H_{18}NO_2S$, 240.1503; found 240.1506.

_N_-benzyl-_N_-vinyl-2,4,6-triisopropylbenzenesulfonamide (VS12)

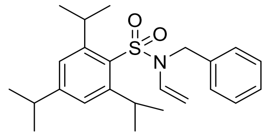

White solid, 34%; ^{1}H NMR (300 MHz, C_6D_6) δ = 7.39 (d, J = 7.7 Hz, 2H), 7.33 (s, 2H), 7.19-7.27 (m, 3H), 7.09 (t, J = 7.3 Hz, 1H), 4.62 (s, 2H), 4.51 (m, 2H), 4.11 (d, J = 4.1 Hz, 1H), 4.07 (s, 1H), 2.75 (m, 1H), 1.38 (d, J = 6.8 Hz, 12H), 1.21 (d, J = 6.9 Hz, 6H); ^{13}C NMR (75 MHz, C_6D_6) δ = 153.7, 151.9, 136.0, 132.7, 131.5, 128.8, 127.5, 127.4, 124.5, 92.3, 48.3, 34.4, 29.8, 25.1, 23.6; HRMS-ESI (m/z) [M + Na]$^+$ calcd. for $C_{24}H_{33}NNaO_2S$, 422.2124; found 422.2123.

N-phenyl-N-vinylmethanesulfonamide (VS13)

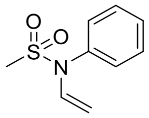

White solid, 80%; ^{1}H NMR (400 MHz, $CDCl_3$) δ = 7.53-7.38 (m, 3H), 7.30 (d, J = 7.30 Hz, 2H), 7.04 (dd, J = 15.4, 8.9 Hz, 1H), 4.27 (d, J = 8.8 Hz, 1H), 3.90 (d, J = 15.5 Hz, 1H), 2.98 (s, 3H); ^{13}C NMR (100 MHz, $CDCl_3$) δ = 135.7, 134.2, 130.0, 129.9, 129.3, 94.1, 39.1; HRMS−ESI (m/z) $[M + Na]^+$ calcd. for $C_9H_{11}NNaO_2S$, 220.0403; found, 220.0405.

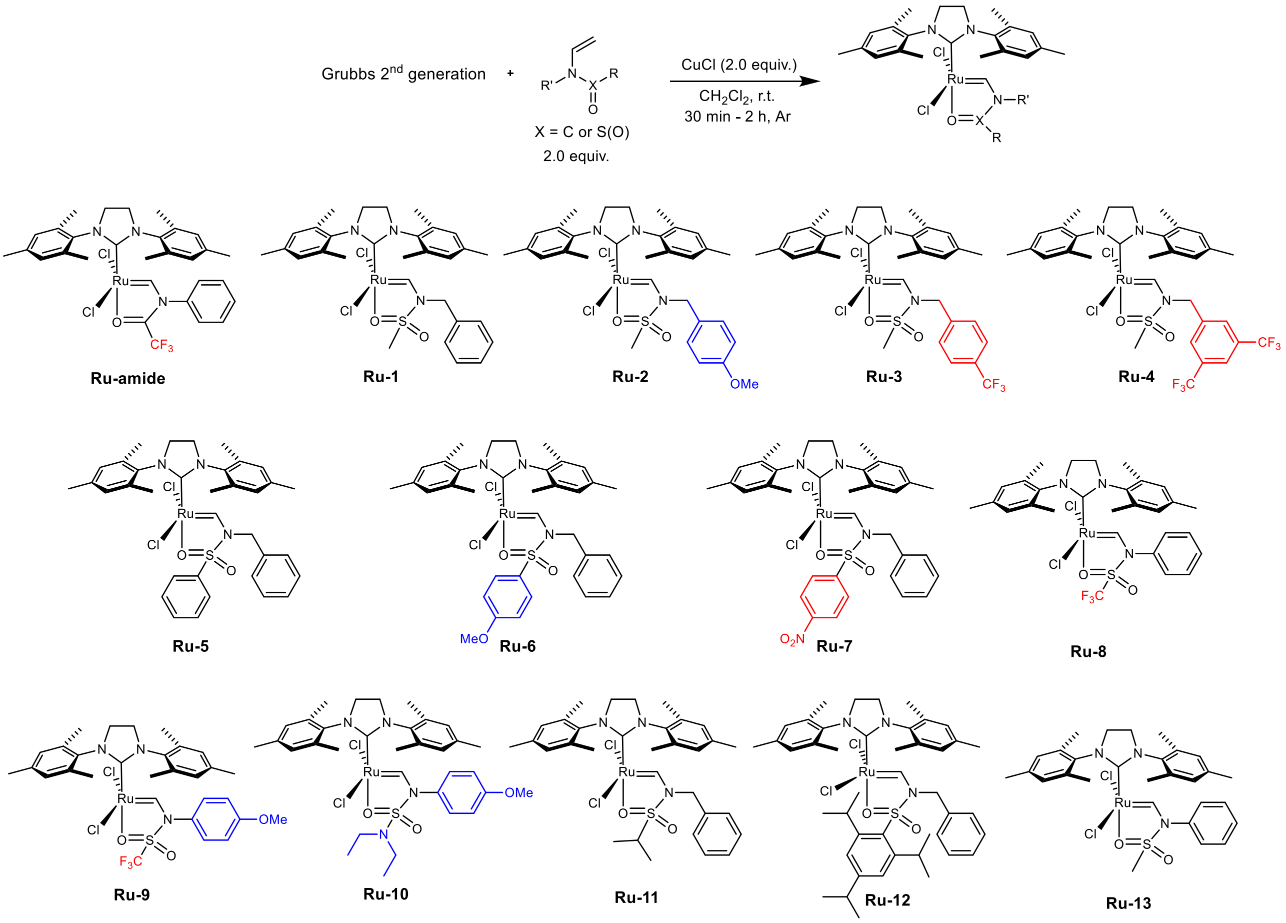

Supplementary Figure 3. Preparation of the *N*-vinylamide- and sulfonamide-based catalysts

General catalyst-preparation precedure: The required vinylated ligand (2.0 equiv., 0.2 mmol), CuCl (0.2 mmol), Grubbs second-generation catalyst (**G-II**) (0.1 mmol), and dichloromethane (4 mL) were added to a 25 mL Schlenk tube in an Ar-filled glove box and the reaction mixture was stirred at room temperature for 30–120 min, after which the solution turned orange.

[1,3-Bis(2,4,6-trimethylphenyl)-2-imidazolidinylidene]dichloro[(2,2,2-trifluoro-*N*-phenylacetamido-

κ*O*)methylene-κ*C*]ruthenium (**Ru-amide**)

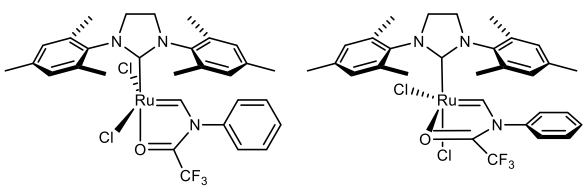

In C_6D_6 *Trans* (1.0) *Cis* (0.18)

In CD_2Cl_2 *Trans* (1.0) *Cis* (0.32)

After following the general procedure, the mixture was purified by silica column chromatography under inert conditions with 7:3 hexane/acetone as the eluent. The brown band was isolated and concentrated in vacuo, and the resulting material was redissolved in benzene and lyophilized to give the desired product.

Dark blue solid, 37%; ^{1}H NMR (400 MHz, C_6D_6) δ = 13.64 (s, 0.16 H, *cis*), 12.19 (s, 1H, *trans*), 7.28 (s, 0.13H, *cis*), 7.03 (s, 0.17H, *cis*), 6.97 (s, 0.17H, *cis*), 6.91 (s, 0.16H, *cis*), 6.87-6.46 (m, 6.23H, *cis+trans*), 6.40 (d, J = 7.8 Hz, 2H, *trans*), 3.36 (s, 4.21H, *cis+trans*), 2.52 (m, 12H, *cis+trans*), 2.18-1.48 (m, 7.8H, *cis+trans*); ^{13}C NMR (100 MHz, CD_2Cl_2) δ = 265.2, 209.4, 139.1, 138.0 (q, J = 82.1 Hz), 130.6, 130.3, 129.5, 129.4, 129.2, 126.7, 126.2, 115.4 (q, J = 282.2 Hz), 51.5, 20.6, 18.8; ^{19}F NMR (376 MHz, CD_2Cl_2) δ = -66.46 (0.32, *cis*), -67.46 (1.0, *trans*); analysis (calcd., found for $C_{30}H_{32}Cl_2F_3N_3ORu$): C (53.02, 52.91), H (4.75, 4.86), N (6.18, 6.09).

[(*N*-benzylmethylsulfonamido-κ*O*)methylene-κ*C*][1,3-bis(2,4,6-trimethylphenyl)-2-imidazolidinylidene]-dichlororuthenium (**Ru-1**)

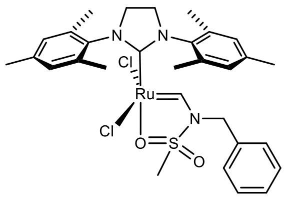

After following the general procedure, the mixture was purified by silica column chromatography under inert and gradient-eluent conditions (dichloromethane only to 50:1 dichloromethane/methanol). The orange band was isolated and concentrated in vacuo, and the resulting material was redissolved in benzene and lyophilized to give the desired product.

Orange solid, >99%; ^{1}H NMR (400 MHz, CD_2Cl_2) δ = 13.12 (s, 1H), 7.39-7.35 (m, 3H), 7.01-6.99 (m, 2H), 6.85 (bs, 4H), 4.70 (d, J = 16.8 Hz, 1H), 4.54 (d, J = 16.8 Hz, 1H), 4.08 (s, 4H), 3.03 (s, 3H), 2.60-2.01 (m, 18H); ^{13}C NMR (100 MHz, CD_2Cl_2) δ = 271.1 (d, J = 6.4 Hz), 211.7, 139.5, 134.7, 130.1, 130.0, 129.9, 129.4, 128.7, 128.4, 126.7, 57.8, 52.1, 44.9, 21.3, 19.6; analysis (calcd., found for $C_{30}H_{37}Cl_2N_3O_2RuS$): C (53.33, 53.75), H (5.52, 5.60), N (6.22, 6.11), S (4.74, 4.65).

[1,3-Bis(2,4,6-trimethylphenyl)-2-imidazolidinylidene]dichloro{[*N*-(4-methoxybenzyl)methylsulfonamido-κ*O*]methylene-κ*C*}ruthenium (**Ru-2**)

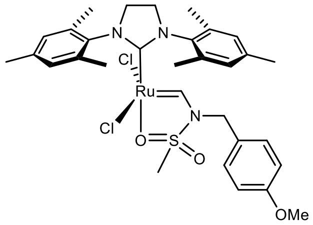

After following the general procedure, the mixture was purified by silica column chromatography under inert and gradient-eluent conditions (dichloromethane only to 50:1 dichloromethane/methanol). The orange band was isolated and concentrated in vacuo, and the resulting material was redissolved in benzene and lyophilized to give the desired product.

Orange solid, >99%; ^{1}H NMR (400 MHz, CD_2Cl_2) δ = 13.14 (s, 1H), 7.06-6.76 (m, 9H), 4.63 (d, J = 16.8 Hz, 1H), 4.44 (d, J = 16.8 Hz, 1H), 4.08 (s, 4H), 3.8. (s, 3H), 2.95 (s, 3H), 2.42 (s, 12H), 2.22 (s, 6H); ^{13}C NMR (100 MHz, CD_2Cl_2) δ = 271.2 (d, J = 9.6 Hz), 211.8, 160.2, 139.4, 139.2, 130.1, 130.0, 128.3, 126.4, 114.7, 58.0, 55.9, 45.0, 21.3, 19.5; analysis (calcd., found for $C_{31}H_{39}N_3O_3RuS$): C (52.76, 53.09), H (5.57, 5.52), N (5.95, 5.91), S (4.54, 4.63).

[1,3-Bis(2,4,6-trimethylphenyl)-2-imidazolidinylidene]dichloro{[*N*-(4-trifluoromethylbenzyl)methyl sulfonamido-κ*O*]methylene-κ*C*}ruthenium (Ru-3)

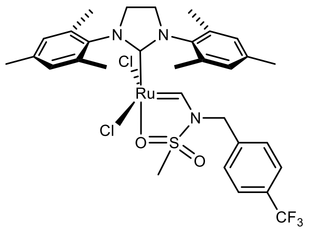

After following the general procedure, the mixture was purified by silica column chromatography under inert and gradient-eluent conditions (dichloromethane only to 50:1 dichloromethane/methanol). The orange band was isolated and concentrated in vacuo, and the resulting material was redissolved in benzene and lyophilized to give the desired product.

Orange solid, 83%; ^{1}H NMR (300 MHz, CD_2Cl_2) δ = 13.02 (s, 1H), 7.57 (d, J = 7.8, 2H), 7.07 (d, J = 7.8, 2H), 6.72 (bs, 4H), 4.58 (s, 2H), 3.98 (s, 4H), 3.05 (s, 3H), 2.28 (bs, 12H) 2.09 (bs, 6H); ^{13}C NMR (125 MHz, CD_2Cl_2) δ = 270.5 (d, J = 10.5 Hz), 210.9, 139.4, 139.2, 131.2 (q, J = 32.5 Hz), 130.2, 130.1, 129.9, 128.7, 127.0, 126.3, 124.7 (q, J = 270.3 Hz), 56.5, 52.1, 44.6, 21.3, 19.5; ^{19}F NMR (376 MHz, CD_2Cl_2) δ = -62.94; analysis (calcd., found for $C_{31}H_{36}Cl_2F_3N_3O_2RuS$): C (50.07, 49.62), H (4.88, 4.96), N (5.65, 5.85), S (4.31, 4.12).

{[*N*-(3,5-bistrifluoromethylbenzyl)methylsulfonamido-κ*O*]methylene-κ*C*}[1,3-bis(2,4,6-trimethylphenyl)-2-imidazolidinylidene] dichlororuthenium (Ru-4)

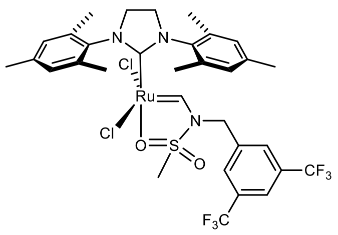

After following the general procedure, the mixture was purified by silica column chromatography under inert and gradient-eluent conditions (dichloromethane only to 50:1 dichloromethane/methanol). The orange band was isolated and concentrated in vacuo, and the resulting material was redissolved in benzene and lyophilized to give the desired product.

Orange solid, 78%; ^{1}H NMR (400 MHz, CD_2Cl_2) δ = 13.05 (s, 1H), 7.93 (s, 1H), 7.56 (s, 2H), 7.14-6.40 (m, 4H), 4.74 (d, J = 16.9 Hz, 1H), 4.64 (d, J = 16.9 Hz, 1H), 4.09 (s, 4H), 3.18 (s, 3H), 2.64-1.99 (m, 18H); ^{13}C NMR (125 MHz, CD_2Cl_2) δ = 269.5 (d, J = 9.4 Hz), 210.6, 139.5, 138.2, 132.7 (q, J = 34.1 Hz), 130.0, 129.9, 126.9, 123.7, (q, J = 273.6 Hz), 122.9, 56.2, 52.1, 44.5, 21.2, 19.4 ; ^{19}F NMR (376 MHz, CD_2Cl_2) δ = -62.85; analysis (calcd., found for $C_{32}H_{35}Cl_2F_6N_3O_2RuS$): C (47.35, 47.56), H (4.35, 4.70), N (5.18, 5.03), S (3.95, 3.73).

[*N*-benzylphenylsulfonamido-κ*O*)methylene-κ*C*][1,3-bis(2,4,6-trimethylphenyl)-2-imidazolidinylidene]dichlororuthenium (Ru-5)

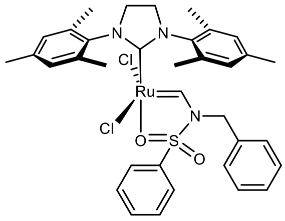

After following the general procedure, the mixture was purified by silica column chromatography under inert and gradient-eluent conditions (dichloromethane only to 50:1 dichloromethane/methanol). The orange band was isolated and concentrated in vacuo, and the resulting material was redissolved in benzene and lyophilized to give the desired product.

Orange solid, 92%; ^{1}H NMR (400 MHz, CD_2Cl_2) δ = 13.05 (s, 1H), 8.16 (d, J =7.5 Hz, 2 H), 7.57 (t, J = 7.5 Hz, 1H), 7.43 (t, J = 7.5 Hz, 2H), 7.27 (s, 3H), 6.87 (m, 2H), 6.84 (bs, 4H), 4.49 (d, J = 17.1 Hz, 1H), 4.28 (d, J = 17.1 Hz, 1H), 4.09 (bs, 4H), 2.78-1.94 (m, 18H); ^{13}C NMR (100 MHz, CD_2Cl_2) δ = 270.0 (d, J = 9.5 Hz), 212.0, 139.3, 135.2, 134.7, 134.4, 130.8, 129.9, 129.8, 128.9, 128.1, 126.2, 55.9, 54.4, 52.2, 21.3, 19.6; analysis (calcd., found for $C_{35}H_{39}Cl_2N_3O_2RuS$): C (56.98, 56.88), H (5.33, 5.32), N (5.70, 5.73), S (4.35, 4.41).

[*N*-benzyl-4-methoxyphenylsulfonamido-κ*O*)methylene-κ*C*][1,3-bis(2,4,6-trimethylphenyl)-2-imidazolidinylidene]dichlororuthenium (Ru-6)

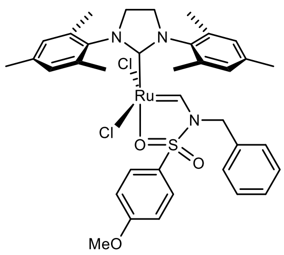

After following the general procedure, the mixture was purified by silica column chromatography under inert and gradient-eluent conditions (dichloromethane only to 50:1 dichloromethane/methanol). The orange band was isolated and concentrated in vacuo, and the resulting material was redissolved in benzene and lyophilized to give the desired product.

Orange solid, 96%; ^{1}H NMR (500 MHz, CD_2Cl_2) δ =13.01 (s, 1H), 8.09 (d, J = 8.9 Hz, 2H), 7.26-7.25 (m, 3H), 7.02-6.63 (m, 8H), 4.46 (d, J = 16.9 Hz, 1H), 4.28 (d, J = 16.9 Hz, 1H), 4.08 (s, 4H), 3.80 (s, 3H), 2.52 (bs, 6H), 2.33-2.26 (m, 9H), 2.01 (bs, 3H); ^{13}C NMR (125 MHz, CD_2Cl_2) δ = 270.0, 212.6, 165.1, 139.2, 134.9, 133.2, 129.9, 128.8, 128.0, 126.2, 125.1, 115.0, 56.3, 55.9, 55.4, 52.1, 21.3, 19.6; analysis (calcd., found for $C_{36}H_{41}Cl_2N_3O_3RuS$): C (56.32, 56.77), H (5.38, 5.32), N (5.47, 5.45), S (4.18, 4.12).

[*N*-benzyl-4-nitrophenylsulfonamido-κ*O*)methylene-κ*C*][1,3-bis(2,4,6-trimethylphenyl)-2-imidazolidinylidene]dichlororuthenium (Ru-7)

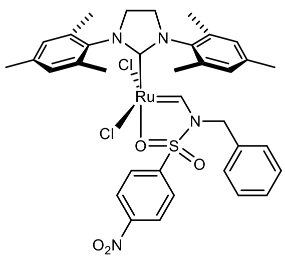

After following the general procedure, the mixture was purified by silica column chromatography under inert and gradient-eluent conditions (dichloromethane only to 50:1 dichloromethane/methanol). The orange band was isolated and concentrated in vacuo, and the resulting material was redissolved in benzene and lyophilized to give the desired product.

Orange solid, 88%; ^{1}H NMR (400 MHz, CD_2Cl_2) δ = 13.09 (s, 1H), 8.36 (d J = 8.6 Hz, 2H), 8.15 (d J = 8.6 Hz, 2H), 7.23 (s, 2H), 7.21 (s, 1H), 6.82 (d, J = 6.3 Hz, 2H), 6.83 (bs, 4H), 4.49 (d, J = 17.1 Hz, 1H), 4.35 (d, J = 17.1 Hz, 1H), 4.10 (s, 4H), 2.60-1.96 (m, 18H); ^{13}C NMR (125 MHz, CD_2Cl_2) δ = 269.7 (d, J = 9.4 Hz), 210.5, 151.4, 140.3, 139.5, 134.0, 132.4, 130.0, 129.0, 128.4, 126.2, 124.7, 56.4, 52.2, 21.3, 19.6; analysis (calcd., found for $C_{35}H_{38}Cl_2N_4O_4RuS$): C (53.71, 54.00), H (4.89, 4.95), N (7.16, 7.13), S (4.10, 4.06).

[1,3-Bis(2,4,6-trimethylphenyl)-2-imidazolidinylidene]dichloro[1,1,1-trifluoro-*N*-phenylmethylesulfonamido-κ*O*)methylene-κ*C*]ruthenium (Ru-8)

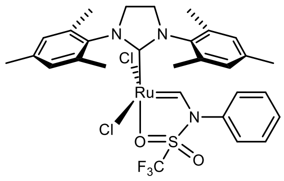

After following the method described in the manuscript, the reaction mixture was concentrated in vacuo, and the residue was washed with diethylether (15 mL) and filtered to remove the CuCl·PCy_3 adduct. Then, the resulting ether solution was diluted with pentane (15 mL) and stored in fridge for 1 day. The desired crystalline solid was washed with excess pentane.

Orange solid, 53%; ^{1}H NMR (400 MHz, CD_2Cl_2) δ = 12.96 (s, 1H), 7.54 (t, J = 7.5 Hz, 1H), 7.45 (t, J = 7.7 Hz, 2H), 7.07 (d, J = 8.0 Hz, 2H), 6.90 (brs, 4H), 4.09 (s, 4H), 2.40 (brs, 12H), 2.10 (brs, 6H); ^{13}C NMR (75 MHz, CD_2Cl_2) δ = 268.7, 206.4, 139.3, 138.4, 137.5, 130.7, 129.9, 129.4, 127.5, 117.9 (q, J = 324.1 Hz), 34.1, 22.3, 20.7, 18.8, 13.8; ^{19}F NMR (376 MHz, CD_2Cl_2) δ = -71.36; analysis (calcd., found for $C_{29}H_{32}Cl_2F_3N_3O_2RuS$): C (48.67, 49.19), H

(4.51, 4.98), N (5.87, 5.84), S (4.48, 4.02).

[1,3-Bis(2,4,6-trimethylphenyl)-2-imidazolidinylidene]dichloro[1,1,1-trifluoro-*N*-4-methoxyphenylmethyl-sulfonamido-κ*O*)methylene-κ*C*]ruthenium (Ru-9)

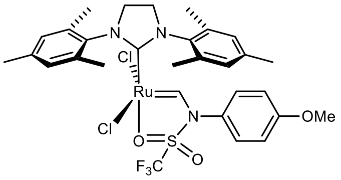

After following the general procedure, the mixture was purified by silica column chromatography under inert conditions with 7:3 hexane/acetone as the eluent. The brown band was isolated and concentrated in vacuo, and the resulting material was redissolved in benzene and lyophilized to give the desired product.

Orange solid, 40%; ^{1}H NMR (500 MHz, CD_2Cl_2) δ = 12.92 (s, 1H), 7.10-6.80 (m, 8H), 4.11 (s, 4H), 3.88 (s, 3H), 2.41 (brs, 12H), 2.14 (brs, 6H); ^{13}C NMR (75 MHz, CD_2Cl_2) δ = 270.4, 207.2, 161.8, 139.8, 138.8, 1306, 130.5, 130.0, 129.7, 118.5 (q, J = 325.3 Hz), 115.4, 56.3, 52.2, 21.3, 19.5; ^{19}F NMR (376 MHz, CD_2Cl_2) δ = -71.38; analysis (calcd., found for $C_{30}H_{34}Cl_2F_3N_3O_3RuS$): C (48.32, 48.66), H (4.60, 4.67), N (5.64, 5.54), S (4.30, 4.21).

[1,3-Bis(2,4,6-trimethylphenyl)-2-imidazolidinylidene]dichloro[*N*,*N*-diethyl-*N'*-4-methoxyphenylsulfamido-κ*O*)methylene-κ*C*]ruthenium (Ru-10)

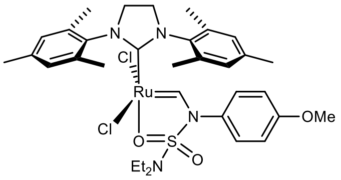

After following the general procedure, the mixture was dissolved in minimal amount of dichloromethane and poured into diethylether (4 mL). Undissolved solids were filtered off and pentane was added to the filtrate. The solution was cooled to −20 °C to grow the crystal. The resulting solids were collected and dried in vacuo to afford the desired product.

Orange solid, 67%; ^{1}H NMR (400 MHz, CD_2Cl_2) δ = 12.81 (s, 1H), 6.92 (d, J = 8.8 Hz, 2H), 6.86 (d, J = 8.8 Hz, 2H), 6.84 (bs, 4H), 4.06 (s, 4H), 3.84 (s, 3H), 3.31 (bs, 2H), 2.95 (bs, 2H), 2.45 (bs, 12H), 2.01 (bs, 6H), 0.84 (t, J = 7.0 Hz, 6H); ^{13}C NMR (100 MHz, CD_2Cl_2) δ = 271.7, 213.8, 160.8, 139.3, 133.5, 129.8, 128.7, 114.8, 56.2, 52.1, 43.5, 21.2, 19.6, 14.1; analysis (calcd., found for $C_{33}H_{44}Cl_2N_4O_3RuS$): C (52.94, 52.87), H (5.92, 6.01), N (7.48, 7.54), S (4.28, 4.28).

[*N*-benzylpropan-2-ylsulfonamido-κ*O*)methylene-κ*C*][1,3-bis(2,4,6-trimethylphenyl)-2-imidazolidinylidene]-dichlororuthenium (Ru-11)

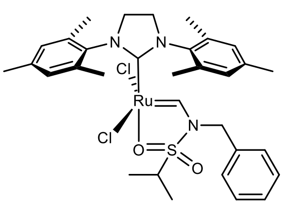

After following the general procedure, the mixture was purified by silica column chromatography under inert and gradient-eluent conditions (dichloromethane only to 50:1 dichloromethane/methanol). The orange band was isolated and concentrated in vacuo, and the resulting material was redissolved in benzene and lyophilized to give the desired product.

Orange solid, 87%; ^{1}H NMR (400 MHz, CD_2Cl_2) δ = 13.16 (s, 1H), 7.37-7.35 (m, 3H), 7.01-6.99 (m, 2H), 7.23-6.15 (m, 4H), 4.77 (d, J = 17.0 Hz, 1H), 4.48 (d, J = 16.9 Hz, 1H), 4.03 (s, 4H), 3.89 (m, 1H), 2.76-1.95 (m, 18H), 1.29 (d, J = 7.1 Hz, 3H), 1.24 (d, J = 6.9 Hz, 3H); ^{13}C NMR (100 MHz, CD_2Cl_2) δ = 271.0, 211.8, 139.2, 135.7, 130.0, 129.2, 128.2, 126.3, 59.2, 57.6, 52.2, 21.4, 19.7, 16.7, 16.4; analysis (calcd., found for $C_{32}H_{41}Cl_2N_3O_2RuS$): C (54.62,

54.64), H (5.87, 5.88), N (5.97, 5.60), S (4.56, 4.43).

[*N*-benzyl-2,4,6-triisopropylphenylsulfonamido-κ*O*)methylene-κ*C*][1,3-bis(2,4,6-trimethylphenyl)-2-imidazolidinylidene]dichlororuthenium (Ru-12)

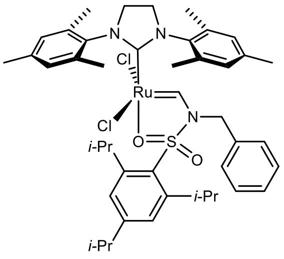

After following the general procedure, the mixture was purified by silica column chromatography under inert and gradient-eluent conditions (dichloromethane only to 50:1 dichloromethane/methanol). The orange band was isolated and concentrated in vacuo, and the resulting material was redissolved in benzene and lyophilized to give the desired product.

Orange solid, 94%; ^{1}H NMR (500 MHz, CD_2Cl_2) δ = 13.04 (s, 1H), 7.32-7.30 (m, 3H), 7.18 (s, 2H), 7.05-6.85 (m, 6H), 4.37 (d, J = 17.4 Hz, 1H), 4.12 (d, J = 17.7 Hz, 1H), 4.08 (s, 4H), 3.78 (m, 2H), 2.90 (m, 1H), 2.60-2.00 (m, 18H), 1.24 (d, J = 6.9 Hz, 6H), 1.02 (d, J = 6.0 Hz, 6H), 0.98 (d, J = 6.0 Hz, 6H); ^{13}C NMR (125 MHz, CD_2Cl_2) δ = 267.7, 211.8, 155.9, 154.3, 135.4, 130.1, 129.9, 129.7, 128.8, 128.0, 126.5, 125.4, 55.8, 52.2, 34.8, 29.9, 25.5, 24.3, 23.7, 21.3, 19.5; analysis (calcd., found for $C_{44}H_{57}Cl_2N_3O_2RuS$): C (61.17, 61.08), H (6.65, 6.62), N (4.86, 4.81), S (3.71, 3.32).

[1,3-Bis(2,4,6-trimethylphenyl)-2-imidazolidinylidene]dichloro[*N*-phenylmethylsulfonamido-κ*O*)methylene-κ*C*]ruthenium (Ru-13)

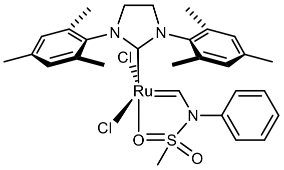

After following the general procedure, the mixture was purified by silica column chromatography under inert conditions with 7:3 hexane/acetone as the eluent. The orange band was isolated and concentrated in vacuo, and the resulting material was redissolved in benzene and lyophilized to give the desired product.

Orange solid, 82%; ^{1}H NMR (500 MHz, CD_2Cl_2) δ = 13.09 (s, 1H), 7.49 (t, J = 7.2 Hz, 1H), 7.41 (t, J = 7.4 Hz, 2H), 7.01 (d, J = 7.4 Hz, 2H), 6.95-6.75 (m, 4H), 4.09 (s, 4H), 3.19 (s, 3H), 2.44 (brs, 12H), 2.44 (brs, 6H); ^{13}C NMR (125 MHz, CD_2Cl_2) δ = 271.5, 211.3, 139.9, 139.6, 139.1, 130.3, 130.2, 130.1, 129.9, 127.3, 52.1, 42.9, 21.2, 19.5; analysis (calcd., found for $C_{29}H_{35}Cl_2N_3O_2RuS$): C (52.64, 52.53), H (5.33, 5.41), N (6.35, 6.38) S (4.85, 4.73).

UV/Vis Initiation Kinetics: A septum equipped screw-capped quartz cuvette for UV/Vis spectroscopy was used to maintain an inert atmosphere. All catalyst solutions were prepared and handled in a glovebox. Cuvettes were tightly sealed, removed from the glovebox, and wrapped with additional Parafilm to secure the cap. UV/Vis-kinetics experiments were performed on a Jasco V-650 UV/Vis spectrophotometer fitted with a temperature controller and a magnetic stirring plate. The solution was allowed to equilibrate to the desired temperature (10 °C) for at least 10 min, prior to commencing the experiment.

General Procedure for UV/Vis-Initiation Kinetics Experiments with BVE: 1.0 mM stock solution of the appropriate catalyst (0.010 mmol) in toluene (10.0 mL solution volume) was prepared in a glovebox. A 0.30 mL aliquot of the 1.0 mM stock solution was dispensed into a UV/Vis cuvette, and additional toluene (2.7 mL) was added to produce 3.0 mL of a 0.10 mM solution of the catalyst in toluene. Separately, a 0.50 M stock solution of butyl vinyl ether (100.2 mg, 1.00 mmol) in toluene (2.0 mL solution volume) was prepared in a septum-topped vial; toluene was added in the glovebox, and butyl vinyl ether was added by syringe outside of the glovebox. The cuvette was placed in the UV/Vis spectrophotometer outside of the glovebox, and allowed to equilibrate to the desired temperature (10 °C) for at least 10 min prior to commencement of the experiment. A spectrum was acquired to determine λ_{max} for the catalyst. An aliquot of the butyl vinyl ether stock solution (18 µL, 9.0 µmol, 30 equiv., 3.0 mM in the reaction solution) was added, and data collection was initialized. Spectra were acquired at regular intervals for at least three half-lives. The absorbance value at λ_{max} was plotted against time, and the data were fit to a first-order exponential decay function, from which k_{obs} (k_{init} for the purpose of this paper) was determined. Other wavelengths (λ_{max} ± 4 nm) gave very similar k_{obs} values. Each experiment was conducted in triplicate for each catalyst. The results are summarized in **Supplementary Table 1**.

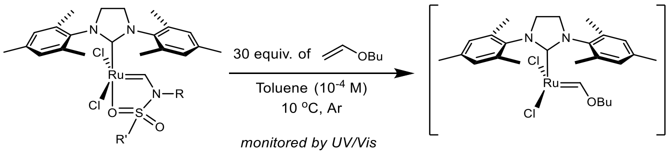

Supplementary Table 1. Summary of the UV/Vis initiation kinetics data

		k_{init} (10^{-4} s^{-1})					
**Ru Catalyst**	**λ_{max}**	**Trial 1**	**Trial 2**	**Trial 3**	**Average**	**Std. Dev.**	**k_{rel}**
**Ru-1**	324	4.62	4.31	4.11	4.35	0.52	64
**Ru-2**	323	2.21	2.66	2.13	2.33	0.57	34
**Ru-3**	325	5.62	5.59	5.20	5.47	0.46	80
**Ru-4**	345	14.0	14.3	16.2	14.8	2.4	220
**Ru-5**	348	6.46	7.51	6.46	6.81	1.2	94
**Ru-6**	341	2.44	2.37	2.61	2.48	0.25	36
**Ru-7**	377	22.9	29.0	22.6	24.8	7.2	360
**Ru-8**	312	125	141	143	136	20	2000
**Ru-9**	312	115	115	109	113	7.2	1600

**Ru-10**	328	0.0642	0.0698	0.0716	0.0685	0.0077	1.0
**Ru-11**	325	2.34	2.09	1.93	2.12	0.42	28
**Ru-12**	331	4.91	4.77	4.92	4.87	0.17	71
**Ru-13**	324	0.154	0.158	0.151	0.154	0.0037	2.2
**Ble**	374	84.6	90.4	82.4	85.8	8.3	1300

Preparation of the stock solution for Ring-Closing Metathesis (RCM): A stock solution was prepared for all RCM reactions. A volumetric flask was charged with catalyst (0.010 mmol) in an Ar-filled glovebox, and CD_2Cl_2 was added to prepare a 1.0 mL stock solution (0.010 M).

Standard RCM kinetics study of diethyl diallyl malonate: An NMR tube with a screw-cap septum top was charged inside a glovebox with the catalyst stock solution (0.010 M, 50 µL 0.50 µmol, 1.0 mol%) and CD_2Cl_2 (0.45 mL). The sample was equilibrated at 30 °C in the NMR probe before **1a** (12.1 µL, 0.050 mmol, 0.1 M) was added using a syringe. Data points were collected over an appropriate period of time using the Varian array function. The conversion to **1b** was determined by integrating the signals for the methylene protons in the starting material, δ 2.61 (dt), with those in the product, δ 2.98 (s).

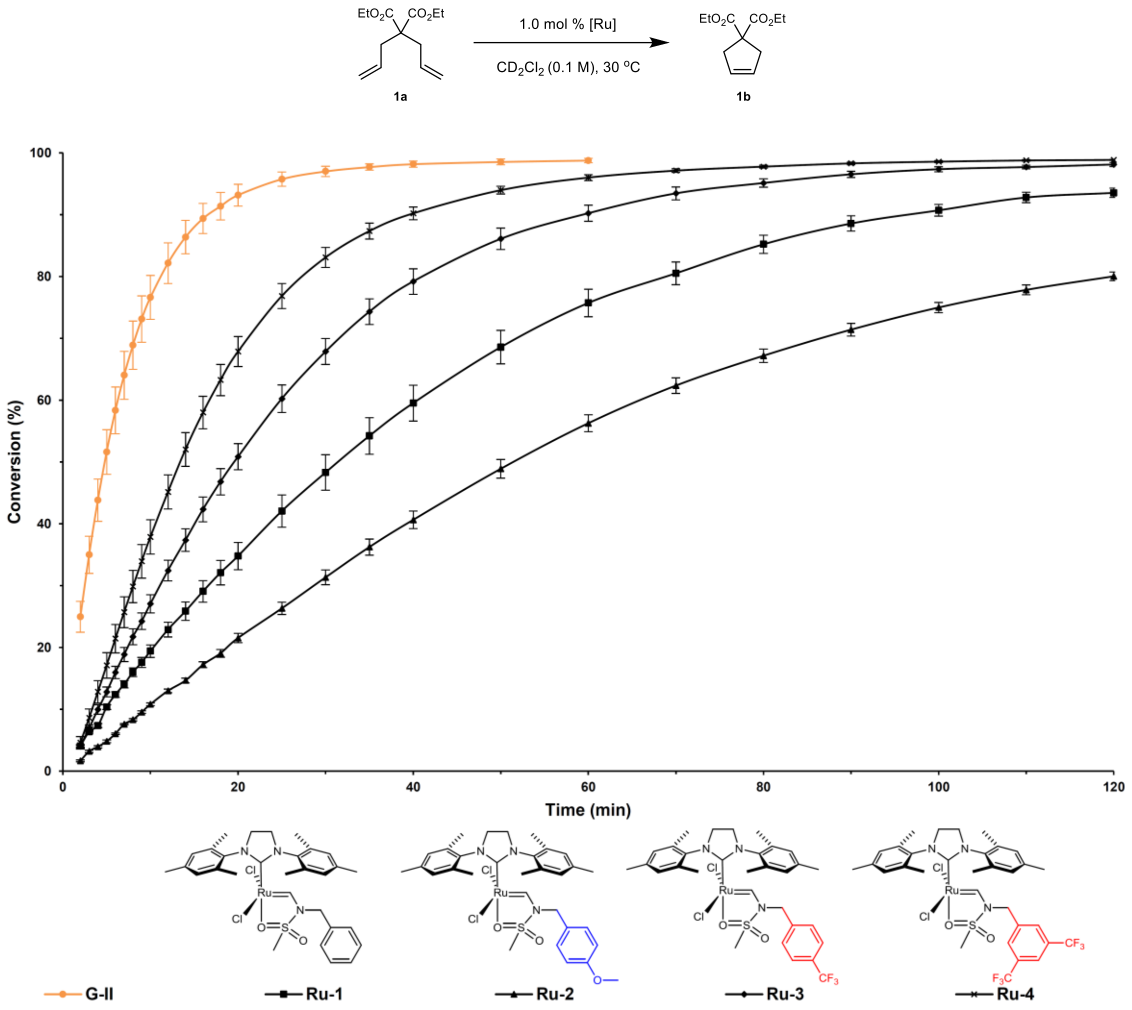

Supplementary Figure 4. ^{1}H NMR kinetic data using **G-II** and **Ru-1–4**. Each data point represents the average of three independent experiments. Error bar represents standard error of the triplicate experiments

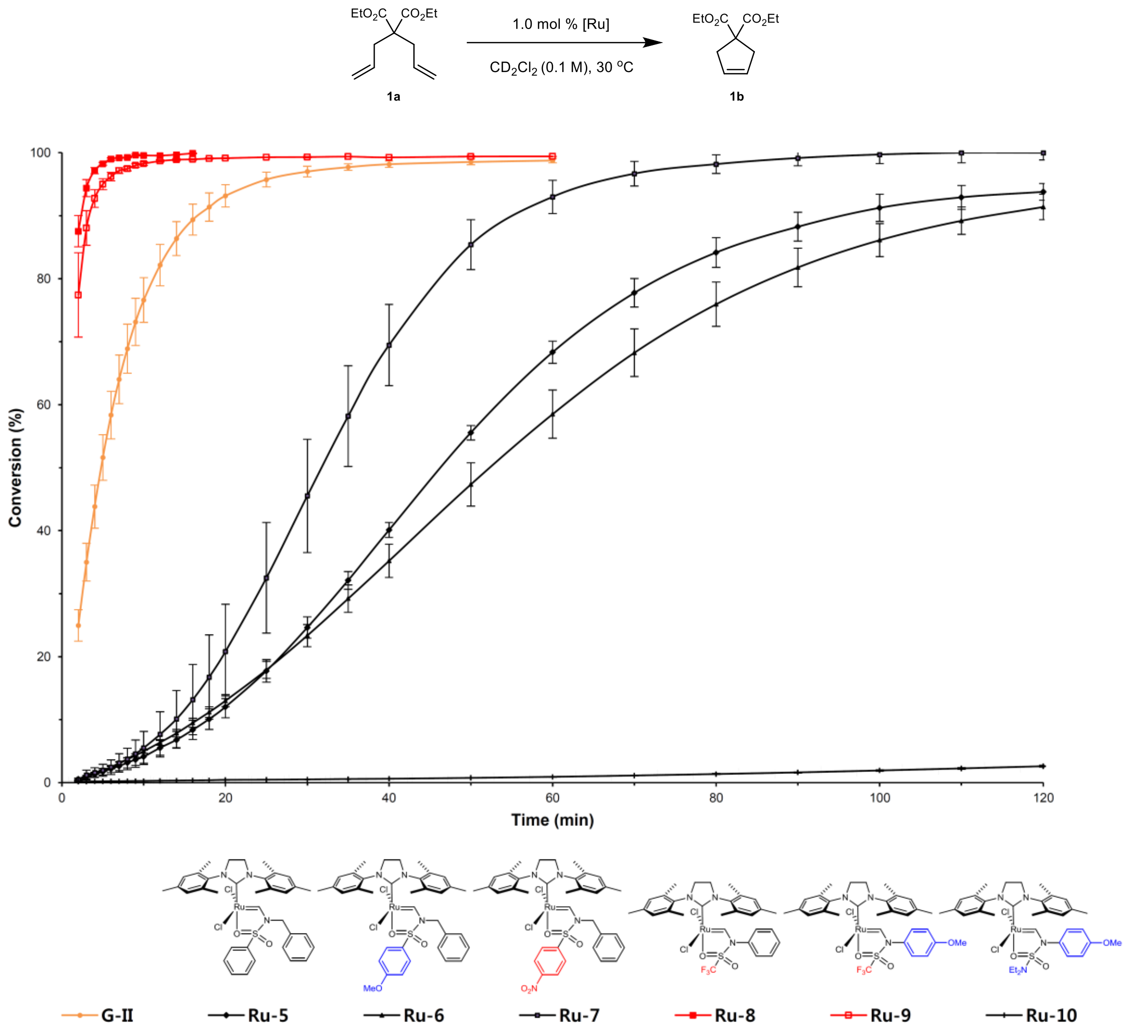

Supplementary Figure 5. ^{1}H NMR kinetic data using **G-II** and **Ru-5–10**. Each data point represents the average of three independent experiments. Error bar represents standard error of the triplicate experiments

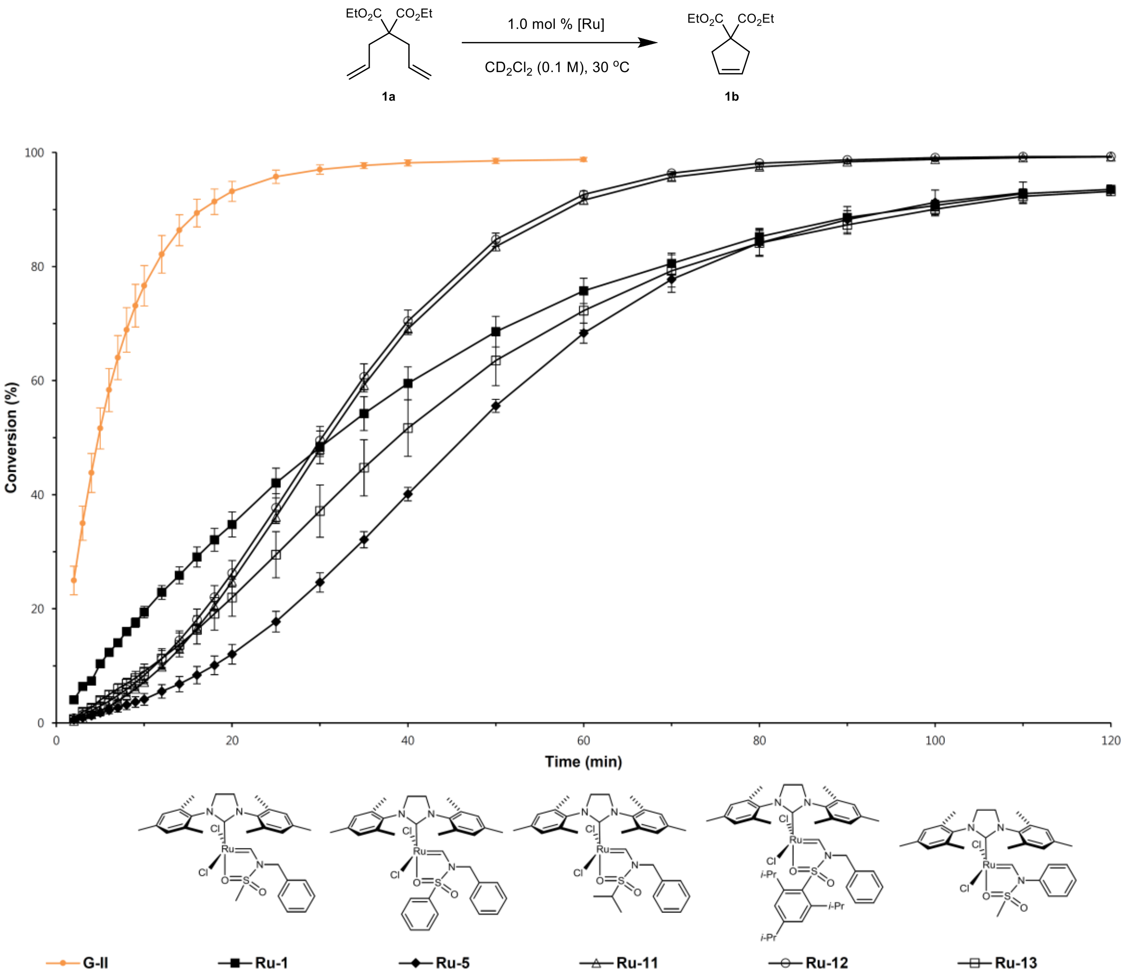

Supplementary Figure 6. ^{1}H NMR kinetic data using **G-II** and **Ru-1**, **Ru-5**, **Ru-11**, **Ru-12** and **Ru-13**. Each data point represents the average of three independent experiments. Error bar represents standard error of the triplicate experiments

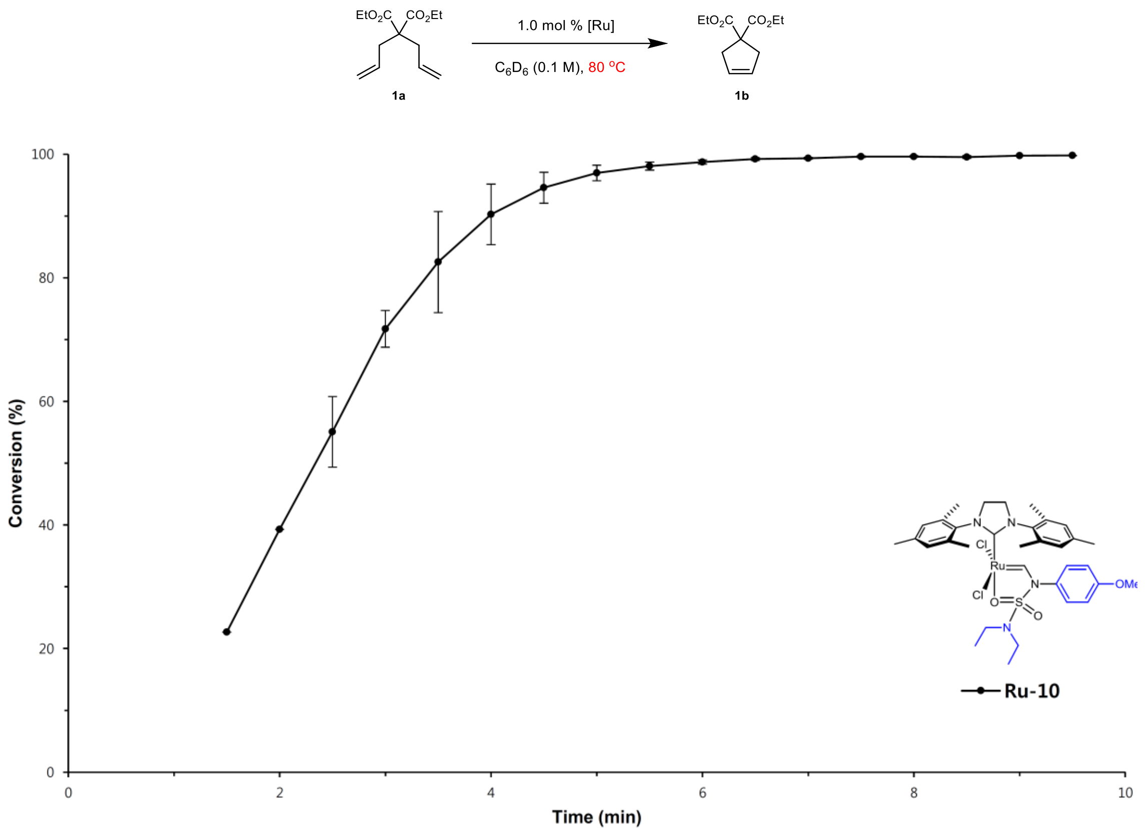

Supplementary Figure 7. ^{1}H NMR kinetic data using **Ru-10** at 80 ºC. Each data point represents the average of three independent experiments. Error bar represents standard error of the triplicate experiments

Preparation of diene substrates: Diene substrates, **S1**, **S2**, **S4**, **S5** and **S6**, were synthesized following literature procedures.[14] **S3** was purchased from Sigma-Aldrich and filtered through a pad of silica prior to use.

General procedure for RCM of diene substrates: An NMR tube with a screw-cap septum top was charged with catalyst stock solution (0.010 M, 50 µL 0.50 µmol, 1.0 mol%) and CD_2Cl_2 (0.45 mL) in a glovebox. The sample was equilibrated at 30 °C in the NMR probe before a diene was added via syringe. Data points were collected over an appropriate period of time using the array function of the Varian NMR instrument. The conversion to the corresponding product was determined by comparing the methylene-proton integrations of the starting diene with those of the product.

Supplementary Table 2. RCM data for various dienes catalyzed by **G-II**, **G-III**, and **Ru-9**

Entry	Product	Catalyst	Time	Conv. (%)
1	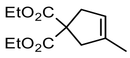 **S1**	**G-II**	1 h (10 min)	98 (60)
		**G-III**	1 h (10 min)	78 (48)
		**Ru-9**	1 h (10 min)	97 (95)
2	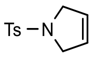 **S2**	**G-II**	20 min (5 min)	>99 (70)
		**G-III**	30 min (5 min)	98 (85)
		**Ru-9**	<5 min	>99
3	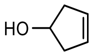 **S3**	**G-II**	45 min	70
		**Ru-9**	<1 min	70
4	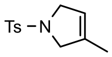 **S4**	**G-II**	30 min (10 min)	92 (67)
		**Ru-9**	30 min (10 min)	>99 (95)
5[c]	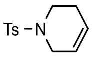 **S5**	**G-II**	40 min	90
		**Ru-9**	<1 min	90
6	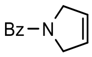 **S6**	**G-II**	10 min	>99
		**G-III**	<1 min	>99
		**Ru-9**	<1 min	>99

[a]Reaction conditions: 0.1 M in CD_2Cl_2, 30 °C. [b]Conversion determined by ^1H NMR. [c]5 mol% of catalyst loading. [d]Bz, Benzamide; Ts, *p*-Toluenesulfonyl.

Cross-Metathesis (CM) of allylbenzene (S7) with *cis*-1,4-diacetoxy-2-butene (S8): Allylbenzene (1.00 mL, 7.55 mmol) and tridecane (0.920 mL, 3.77 mmol) were combined and stirred in a 4 mL vial under inert conditions. The reaction was carried out with a 51 µL aliquot of this solution in lieu of the separate addition of allylbenzene and tridecane.

A 5.0 µmol sample of the catalyst and 1.0 mL of anhydrous dichloromethane were added to a 4 mL vial. *cis*-1,4-Diacetoxy-2-butene (64 µL, 0.40 mmol) and the prepared allylbenzene/tridecane mixture (51 µL; 0.20 mmol allylbenzene + 0.10 mmol tridecane) were then added using a syringe. The reaction mixture was allowed to stir at 23 °C, during which time aliquots were taken at specified time periods.

Samples for GC were prepared by adding a ~30 µL reaction aliquot to 500 µL of a 3 M solution of ethyl vinyl ether in dichloromethane. The sample was shaken, allowed to stand for 5 min, and then analyzed by GC. All reactions were performed in duplicate to confirm reproducibility.

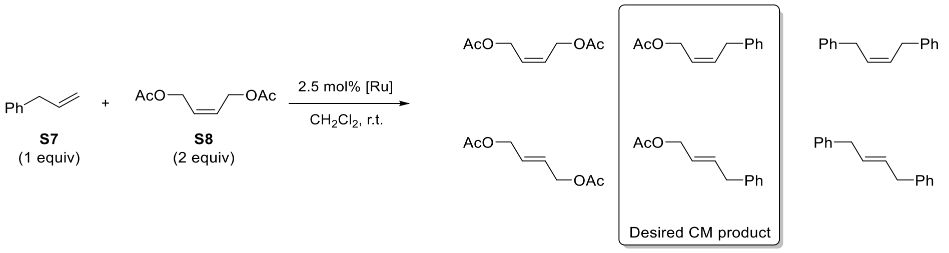

Supplementary Table 3. Kinetic comparison with **G-II** and **Ru-9**

G-II

time (min)	4	7	10	15	20	30
% conv. to CM Product	26.6	65.3	80.6	86.7	87.1	86.6
E:*Z* of CM Product	2.8	4.1	5.6	7.3	8.5	9.3

Ru-9

time (min)	1	4	7	10	15	20
% conv. to CM Product	78.4	83.7	83.9	82.3	82.3	84.1
E:*Z* of CM Product	9.6	9.4	9.5	9.6	9.7	9.7

CM of 5-hexenyl acetate (2) and methyl acrylate (3): 5-Hexenyl acetate (96.63 µL, 0.6 mmol) and methyl acrylate (54.03 µL, 0.6 mmol) were added to a solution of anthracene (15 mg) in 1.5 mL CD_2Cl_2 in a 5 mL long neck Schlenk tube fitted with an Ar-filled balloon. An appropriate aliquot was taken from the solution and diluted with CD_2Cl_2 in an NMR tube for the t = 0 time point. The catalyst (0.015 mmol, 2.5 mol%) was then added and aliquots were taken from the reaction solution at the desired times, diluted with CD_2Cl_2 and stored at –78 °C until the NMR spectrum was acquired. Each experiment was repeated twice to confirm reproducibility.

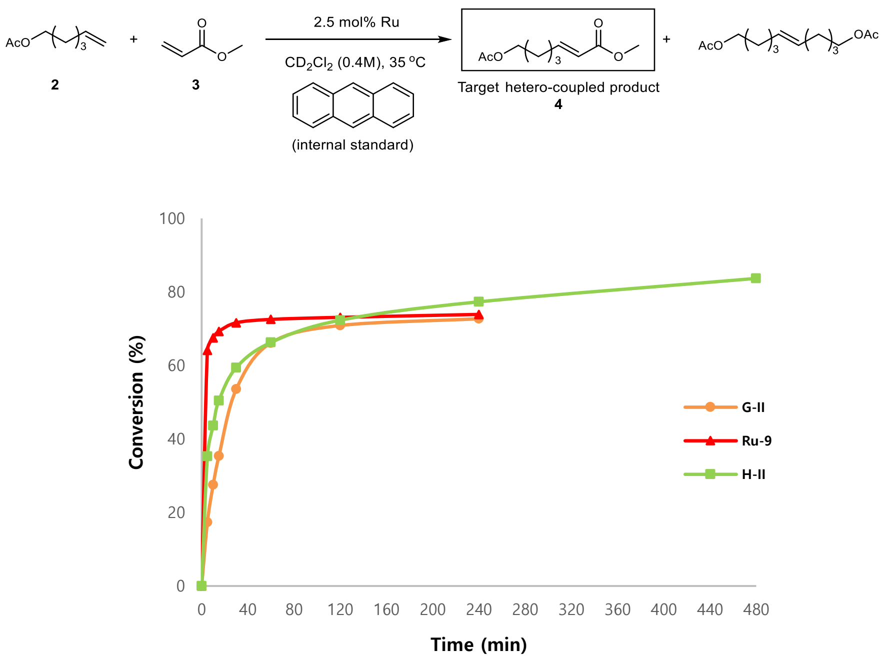

Supplementary Figure 8. ^{1}H NMR kinetic data using catalysts **G-II**, **H-II**, and **Ru-9**

General procedure for the CM of 5-hexenyl acetate (2) and methyl acrylate (3) for boomerang mechanism study: The catalyst (0.020 mmol) was added to 1.0 mL of CD_2Cl_2 in an Ar-filled dry box. The ^{15}N labeled ligand (0.10 mmol) was added to 1.0 mL of CD_2Cl_2, after which 250 µL of the catalyst stock solution and 50 µL of the ligand stock solution were combined in a screw-capped NMR tube and tightly sealed. 5-Hexenyl acetate (161 µL, 1.0 mmol) and methyl acrylate (90 µL, 1.0 mmol) were added to a solution of anthracene (10 mg) in 1.0 mL of CD_2Cl_2. An appropriate aliquot was taken from the solution and diluted with CD_2Cl_2 in an NMR tube for the t = 0 time point. A 200-µL aliquot of the substrate stock solution was added to the NMR tube and time-resolved NMR spectroscopy was conducted at room temperature. Each experiment was repeated twice to confirm reproducibility.

Background CM: The catalyst (0.020 mmol) was added to 1 mL of CD_2Cl_2 in an Ar-filled dry box. The ^{15}N labeled ligand (0.10 mmol) was added to 1.0 mL of CD_2Cl_2, after which 250 µL of the catalyst stock solution and 50 µL of the ligand stock solution were combined in a screw-capped NMR tube, an additional 200 µL of CD_2Cl_2 was added and tightly sealed. Time resolved NMR spectroscopy was performed at room temperature for 2 d and no exchange was detected. Each reaction was repeated twice to confirm reproducibility.

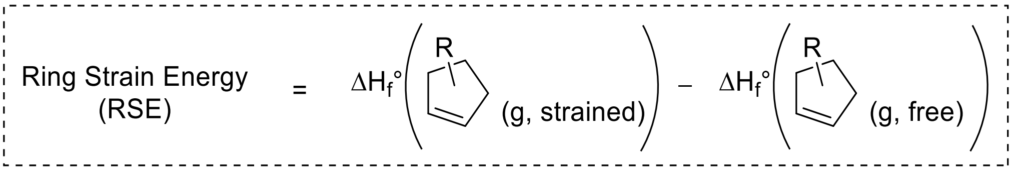

Calculation of the ring strain: The ring-strain energies (RSEs) of a series of substituted cyclopentenes were calculated using the conventional definition of RSE; i.e., the difference between the standard heat of formation ($\Delta_f H°$) of the strained molecule and the $\Delta H_f°$ of the strain-free molecule. The $\Delta_f H°$ of the strained molecule ($\Delta_f H°$ (g, strained,

298 K)) was calculated using Gaussian 09W,[15] and the $\Delta_f H°$ of the strain-free molecule ($\Delta_f H°$ (g, free, 298 K)) was calculated using Benson's group increment theory,[16] which does not consider any destabilization derived from the ring structure.

Computation of the $\Delta_f H°$ of the strained molecule ($\Delta_f H°$ (g, strained, 298 K)): The geometry of each molecule was optimization and frequencies calculated in the gas phase at the RB3LYP/6-31++G(2df,2p) level of theory. All structures were confirmed to be local minima by the absence of imaginary frequencies. The lowest conformer was used in further calculations. Single-point energy calculations were conducted on the optimized geometries using the G3 compound method.[17]

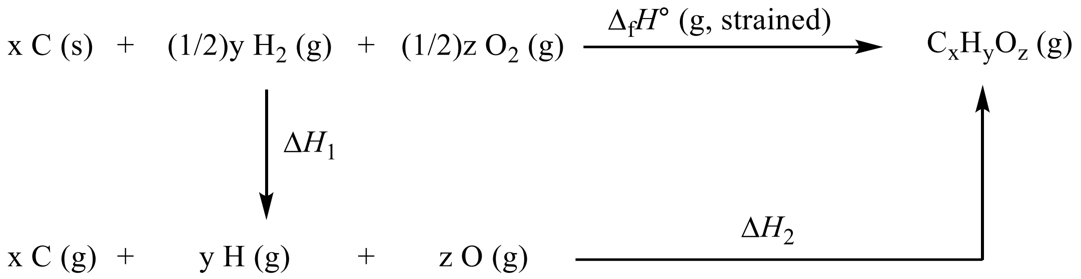

Specifically, the Hess cycle shown above was applied to computationally determine $\Delta_f H°$ (g, strained). The enthalpy of formation of the isolated atoms (ΔH_1) was calculated using the reported enthalpy of atomization of each element (**Supplementary Table 4**). The atomization energy ($-\Delta H_2$) was calculated using the G3 energy (0 K) for each atom and the target molecule. $\Delta_f H°$ (g, strained) at 0 K was obtained by adding ΔH_1 and ΔH_2.

Supplementary Table 4. Reported thermodynamics data and calculated G3 energies for atomic C, H, and O[18]

Atom	$\Delta_f H°$ (expt) [kJ/mol]	G3 energy (0 K) [Hartree]	Thermal correction (0-298K) [kcal/mol]
C (triplet)	711.2	−37.827717	0.25
H	216.035	−0.501003	1.01
O (doublet)	246.8	−75.030991	1.04

Supplementary Table 5. Calculated G3 energy and enthalpy of each monomer

Monomer	Chemical formula	G3 energy (0 K) [Hartree]	ΔH_1 [kJ/mol]	ΔH_2 [kJ/mol]	$\Delta_f H°$ (g, strained, 0 K) [kJ/mol]
**10**	C_5H_8	−195.135809	5284.28	−5222.64460000	61.63540000
**11**	$C_6H_{10}O$	−309.59261	6674.35	−6787.67101850	−113.32101850
**12**	$C_{12}H_{14}O$	−540.46045	11805.69	−11769.62553150	36.06446850
**13**	C_5H_6O	−269.139753	5099.01	−5156.89945450	−57.88945450
**14**	$C_{12}H_{14}O$	−540.459004	11805.69	−11765.82905850	39.86094150
**15**	$C_{11}H_{12}$	−426.017324	10415.62	−10240.50282550	175.11717450
**16**	$C_{12}H_{14}$	−465.288829	11558.89	−11400.40146650	158.48853350
**17**	$C_7H_{10}O_2$	−422.89113	7632.35	−7942.39742450	−310.04742450
**18**	$C_8H_{12}O_3$	−537.354073	9022.42	−9523.54966400	−501.12966400
**19**	C_5H_6	−193.933767	4852.21	−4697.45008200	154.75991800
**DCPD**	$C_{10}H_{12}$	−387.903204	9704.42	−9488.55174900	215.86825100

The $\Delta_f H°$ (g, strained) at 298 K was obtained applying an enthalpy correction term calculated by G3 (for the target molecule) and the experimentally reported enthalpy correction terms (for the constituent atoms), using the equation shown below.

$$\Delta_f H° \left(C_x H_y O_z, \text{strained}, 298\ \text{K}\right) = \Delta_f H° \left(C_x H_y O_z, \text{strained}, 0\ \text{K}\right) + \left[H° \left(C_x H_y O_z, \quad 298\ \text{K}\right) - H° \left(C_x H_y O_z, \quad 0\ \text{K}\right)\right] - x[H° (\text{C}, \quad 298\ \text{K}) - H° (\text{C}, \quad 0\ \text{K})]_{\text{st}} - y[H° (\text{H}, \quad 298\ \text{K}) - H° (\text{H}, \quad 0\ \text{K})]_{\text{st}} - z[H° (\text{O}, \quad 298\ \text{K}) - H° (\text{O}, \quad 0\ \text{K})]_{\text{st}}$$

Supplementary Table 6. Calculated thermal correction factor and $\Delta_f H°$ (g, strained, 298 K) for each monomer

Monomer	Chemical formula	$\Delta_f H°$ (g, strained, 0 K) [kJ/mol]	$H°$ (298 K) − $H°$ (0 K) [hartree]	$\Delta_f H°$ (g, strained, 298 K) [kJ/mol]
**10**	C_5H_8	61.63540000	0.005712	37.63285600
**11**	$C_6H_{10}O$	−113.32101850	0.008156	−144.74264050
**12**	$C_{12}H_{14}O$	36.06446850	0.01254	−7.00416150
**13**	C_5H_6O	−57.88945450	0.006252	−76.37782850
**14**	$C_{12}H_{14}O$	39.86094150	0.012772	−2.59857250
**15**	$C_{11}H_{12}$	175.11717450	0.010211	139.76955500
**16**	$C_{12}H_{14}$	158.48853350	0.011545	117.15473100
**17**	$C_7H_{10}O_2$	−310.04742450	0.010117	−341.71264100
**18**	$C_8H_{12}O_3$	−501.12966400	0.012281	−540.94909850
**19**	C_5H_6	154.75991800	0.005183	137.81208450
**DCPD**	$C_{10}H_{12}$	215.86825100	0.008222	176.34351200

Calculation of $\Delta_f H°$ for a strain-free molecule ($\Delta_f H°$ (g, free)): The $\Delta_f H°$ (g, free) was independently calculated using the group additivity values reported by Aubry.[19] A simple determination using the linear combination of known group additivity values was applied in order to obtain terms that are not reported, as described below.

Supplementary Table 7. Calculated values of unknown group additivities

Unknown group additivity value	Method 1	Method 2	Average value [kJ/mol]
C–(C$_d$)(C$_B$)(C)(H)	[C–(C$_d$)(C$_B$)(H)$_2$] + [C–(C$_d$)(C)(H)$_2$] – [C–(C$_d$)(H)$_3$] = 3.9 kJ/mol	[C–(C$_d$)(C$_B$)(H)$_2$] + [C–(C$_B$)(C)(H)$_2$] – [C–(C$_B$)(H)$_3$] = 4.0 kJ/mol	4.0
C–(C$_d$)(O)(C)(H)	[C–(C$_d$)$_2$(O)(H)] + [C–(C)(O)(H)$_2$] – [C–(C$_d$)(O)(H)$_2$] = –25 kJ/mol	[C–(C)$_2$(O)(H)] + [C–(C$_d$)(O)(H)$_2$] – [C–(C)(O)(H)$_2$] = –22 kJ/mol	–23.5

The calculation procedure and results for each monomer are provided below.

(1) Monomer **10**

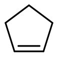

$\Delta_f H°$ (**10**, g, free, 298 K) = 2[C_d–(C)(H)] + 2[C–(C_d)(C)($H)_2$] + [C–$(C)_2(H)_2$] = 2(35.8) + 2(−20.1) + (−20.9) = 10.5 kJ/mol

(2) Monomer **11**

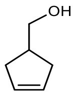

$\Delta_f H°$ (**11**, g, free, 298 K) = 2[C_d–(C)(H)] + 2[C–(C_d)(C)$(H)_2$] + [C–$(C)_3$(H)] + [C–$(H)_2$(C)(O)] + [O–(H)(C)]
= 2(35.8) + 2(−20.1) + (−7) + (−33) + (−159) = −167.6 kJ/mol

(3) Monomer **12**

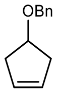

$\Delta_f H°$ (**12**, g, free, 298 K) = 2[C_d–(C)(H)] + 2[C–(C_d)(C)$(H)_2$] + [C–(O)(C_d)(C)(H)] + [O–$(C)_2$] + [C–(C_B)(O)$(H)_2$] + [C_B–(C)] + 5[C_B–(H)]
= 2(35.8) + 2(−20.1) + (−23.5) + (−99) + (−26) + (23) + 5(13.8)
= −25.1 kJ/mol

(4) Monomer **13**

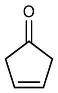

$\Delta_f H°$ (**13**, g, free, 298 K) = 2[C_d–(C)(H)] + 2[C–(CO)(C_d)$(H)_2$] + [CO–$(C)_2$] = 2(35.8) + 2(−16) + (−133) = −93.4 kJ/mol

(5) Monomer **14**

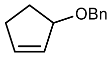

$\Delta_f H°$ (**14**, g, free, 298 K) = 2[C_d–(C)(H)] + [C–(C_d)(C)$(H)_2$] + [C–$(C)_2(H)_2$] + [C–(O)(C_d)(C)(H)] + [O–$(C)_2$] + [C–(C_B)(O)$(H)_2$] + [C_B–(C)] + 5[C_B–(H)]
= 2(35.8) + (−20.1) + (−20.9) + (−23.5) + (−99) + (−26) + (23) + 5(13.8) = −25.9 kJ/mol

(6) Monomer **15**

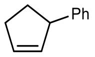

$\Delta_f H°$ (**15**, g, free, 298 K) = 2[C_d–(C)(H)] + [C–(C_d)(C)$(H)_2$] + [C–$(C)_2(H)_2$] + [C–(C_d)(C_B)(C)(H)] + [C_B–(C)] + 5[C_B–(H)]
= 2(35.8) + (−20.1) + (−20.9) + (4.0) + (23) + 5(13.8) = −126.6 kJ/mol

(7) Monomer **16**

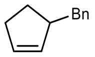

$\Delta_f H°$ (**16**, g, free, 298 K) = 2[C_d–(C)(H)] + [C–(C_d)(C)$(H)_2$] + [C–$(C)_2(H)_2$] + [C–(C_d)(H)$(C)_2$] + [C–(C)(C_B)$(H)_2$] + [C_B–(C)] + 5[C_B–(H)]
= 2(35.8) + (−20.1) + (−20.9) + (−7.0) + (−20) + (23) + 5(13.8) = 95.6 kJ/mol

(8) Monomer **17**

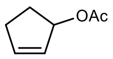

$\Delta_f H°$ (**17**, g, free, 298 K) = $2[C_d\text{–}(C)(H)] + [C\text{–}(C_d)(C)(H)_2] + [C\text{–}(C)_2(H)_2] + [O\text{–}(C)(CO)] + [CO\text{–}(O)(C)] + [C\text{–}(CO)(H)_3]$
$= 2(35.8) + (-20.1) + (-20.9) + (-23.5) + (-180) + (-147) + (-42) = -361.9$ kJ/mol

(9) Monomer **18**

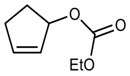

$\Delta_f H°$ (**18**, g, free, 298 K) = $2[C_d\text{–}(C)(H)] + [C\text{–}(C_d)(C)(H)_2] + [C\text{–}(C)_2(H)_2] + [C\text{–}(O)(C_d)(C)(H)] + 2[O\text{–}(CO)(C)] + [CO\text{–}(O)_2] + [C\text{–}(O)(C)(H)_2] + [C\text{–}(C)(H)_3]$
$= 2(35.8) + (-20.1) + (-20.9) + (-23.5) + 2(-180) + (-129) + (-33) + (-42) = -556.9$ kJ/mol

(10) Monomer **19**

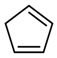

$\Delta_f H°$ (**19**, g, free, 298 K) = $2[C_d\text{–}(C)(H)] + 2[C_d\text{–}(C_d)(H)] + [C\text{–}(C_d)_2(H)_2] = 2(35.8) + 2(28.4) + (-18) = 110.4$ kJ/mol

(11) Dicyclopentadiene (**DCPD**)

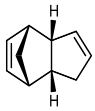

$\Delta_f H°$ (**DCPD**, g, free, 298 K) = $3[C_d\text{–}(C)(H)] + 2[C\text{–}(C_d)(C)_2(H)] + [C\text{–}(C_d)_2(C)(H)] + 2[C\text{–}(C)_2(H)_2] + [C_d\text{–}(C)_2] + [C\text{–}(C_d)(C)(H)_2] = 3(35.8) + 2(-7) + (-7) + 2(-20.9) + (42.6) + (-20.1) = 67.1$ kJ/mol

Combined data for each monomer and calculated ring-strain energies are listed in **Supplementary Table 8**.

Supplementary Table 8. Calculated ring-strain energies for monomers **10–19**

Monomer	$\Delta_f H°$ (g, strained, 298 K) [kJ/mol]	$\Delta_f H°$ (g, free, 298 K) [kJ/mol]	Ring strain energy [kJ/mol]	Ring strain energy [kcal/mol]
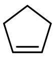 **10**	37.63	10.5	27.13	**6.48**
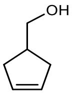 **11**	−144.74	−167.6	22.86	**5.46**
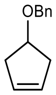 **12**	−7.00	−25.1	18.1	**4.33**
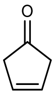 **13**	−76.38	−93.4	17.02	**4.07**
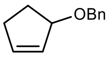 **14**	−2.60	−25.9	23.3	**5.57**
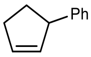 **15**	139.77	126.6	13.17	**3.15**
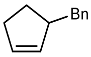 **16**	117.15	95.6	21.55	**5.15**
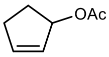 **17**	−341.71	−361.9	20.19	**4.83**
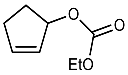 **18**	−540.95	−556.9	15.95	**3.81**
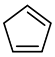 **19**	137.81	110.4	27.41	**6.55**
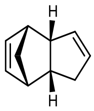 **DCPD**	176.34	67.1	109.24	**26.13**

Cartesian coordinates of the optimized geometry for each monomers are described below.

Monomer **10**

C	−0.000004	−1.228535	0.130872
C	−1.236618	−0.317795	−0.099711
C	−0.667391	1.076139	0.045180
C	0.667399	1.076134	0.045185
C	1.236614	−0.317805	−0.099714
H	−0.000008	−2.110957	−0.512835
H	−2.045440	−0.524196	0.608952
H	−1.664071	−0.454918	−1.102566
H	−1.290979	1.961281	0.101582
H	1.290995	1.961271	0.101595
H	2.045440	−0.524214	0.608942
H	1.664060	−0.454926	−1.102573
H	−0.000006	−1.582166	1.166028

Monomer **11**

C	0.251901	−0.258179	0.573134
C	−0.327323	1.176422	0.390898
C	−1.638120	0.950345	−0.323881
C	−1.969207	−0.340706	−0.379818
C	−0.937795	−1.222822	0.284263
H	0.636294	−0.396335	1.586838
H	0.360560	1.821463	−0.164937
H	−0.490646	1.671033	1.356616
H	−2.236663	1.763733	−0.718821
H	−2.876511	−0.735761	−0.822817
H	−0.638079	−2.066147	−0.350795
H	−1.333944	−1.666574	1.206278
C	1.403325	−0.533023	−0.390498
H	1.718141	−1.581701	−0.295461
H	1.066723	−0.371172	−1.425545
O	2.488120	0.339391	−0.072685
H	3.192472	0.214108	−0.714463

Monomer **12**

C	−1.510716	0.062887	−0.270169
C	−2.464216	−1.163244	−0.381840
C	−3.845742	−0.558383	−0.312952
C	−3.815198	0.773591	−0.373193
C	−2.408120	1.305694	−0.488097
H	−0.703630	0.014416	−1.008568
H	−2.278038	−1.880798	0.424282
H	−2.313584	−1.705125	−1.324444
H	−4.743722	−1.160585	−0.235572
H	−4.684771	1.420311	−0.355596
H	−2.173559	2.068233	0.260937
H	−2.230631	1.759151	−1.471085
O	−0.938443	0.174610	1.035429
C	0.152679	−0.697600	1.290708
H	0.310542	−0.638424	2.371984
H	−0.108765	−1.738825	1.056326
C	1.427456	−0.316483	0.559749
C	2.162110	−1.271013	−0.147798
C	1.904482	0.999233	0.610443
C	3.353804	−0.926089	−0.788827
H	1.800251	−2.293461	−0.199378
C	3.089617	1.348814	−0.032195
H	1.336291	1.747876	1.151638
C	3.819819	0.385838	−0.732903
H	3.911190	−1.679481	−1.334546
H	3.447167	2.371675	0.015001
H	4.742951	0.658688	−1.231972

Monomer **13**

C	0.857245	0.000000	0.000145
C	−0.042521	−1.245663	0.000200
C	−1.434105	−0.668664	−0.000159
C	−1.434105	0.668664	−0.000133
C	−0.042521	1.245664	0.000187
H	0.180516	−1.864953	0.877368
H	0.180870	−1.865418	−0.876534
H	−2.323567	−1.287007	−0.000419
H	−2.323566	1.287007	−0.000382
H	0.180521	1.864951	0.877356
H	0.180865	1.865422	−0.876547
O	2.062550	0.000000	−0.000285

Monomer **14**

C	3.008854	0.719333	1.000163
C	2.093934	−0.470595	0.636494
C	2.740986	−1.041639	−0.610639
C	3.676042	−0.224584	−1.099622
C	3.859309	1.016254	−0.261284
H	3.661101	0.432309	1.828672
H	2.030496	−1.206412	1.451635
H	2.440049	−1.982107	−1.056048
H	4.241292	−0.401781	−2.008281
H	3.510149	1.904036	−0.802863
H	4.910713	1.198879	−0.016293
H	2.408316	1.568789	1.329065
O	0.770736	0.027435	0.396452
C	−0.204315	−0.976302	0.215431
H	0.020421	−1.575706	−0.680592
H	−0.194050	−1.668466	1.073568
C	−1.581604	−0.368149	0.071648

Monomer **15**

C	−1.739594	−0.635075	−1.017440
C	−0.948302	−0.569321	0.327302
C	−1.764506	0.433212	1.124389
C	−2.959983	0.664592	0.578638
C	−3.168103	−0.134075	−0.687981
H	−1.722649	−1.634269	−1.458220
H	−1.009111	−1.544315	0.831156
H	−1.404003	0.857205	2.054683
H	−3.723768	1.307308	1.001803
H	−3.605637	0.454446	−1.500979
H	−3.858700	−0.968563	−0.504209
H	−1.270566	0.046074	−1.732981
C	0.522846	−0.238511	0.166295
C	1.500216	−1.223124	0.345965
C	0.940299	1.051665	−0.188047
C	2.854467	−0.935436	0.171108
H	1.197263	−2.227175	0.627265

C	−2.701153	−1.207792	0.100103
C	−1.770129	1.003507	−0.108991
C	−3.984755	−0.689637	−0.053557
H	−2.567807	−2.276032	0.245669
H	−3.056967	1.524136	−0.260333
H	−0.908338	1.657653	−0.126799
C	−4.167282	0.682554	−0.234725
H	−4.841106	−1.354579	−0.027644
H	−3.189218	2.591904	−0.397876
H	−5.165432	1.089513	−0.351955

C	2.291359	1.345080	−0.361817
H	0.200049	1.833494	−0.324049
C	3.255254	0.351184	−0.184649
H	3.593918	−1.715691	0.315333
H	2.592782	2.351031	−0.633443
H	4.306700	0.579306	−0.318929

Monomer **16**

C	−1.679352	0.969243	−0.474251
C	−1.428781	−0.519055	−0.103738
C	−2.801790	−0.979686	0.341769
C	−3.760768	−0.110005	0.018466
C	−3.207086	1.093785	−0.710026
H	−1.086724	1.297693	−1.329995
H	−1.141279	−1.083286	−1.003620
H	−2.961883	−1.933976	0.832543
H	−4.819608	−0.249531	0.205900
H	−3.613620	2.041793	−0.342889
H	−3.459444	1.043971	−1.778128
H	−1.395185	1.598214	0.375960
C	−0.321424	−0.744430	0.950626
H	−0.321776	−1.804321	1.231607
H	−0.577385	−0.179652	1.853771
C	1.062045	−0.355289	0.477469
C	1.667170	0.834229	0.898000
C	1.769380	−1.177358	−0.410403
C	2.934258	1.199135	0.440970
H	1.142996	1.480577	1.594742
C	3.035152	−0.818874	−0.870043
H	1.326263	−2.112400	−0.739928
C	3.622913	0.374149	−0.446767
H	3.383196	2.125634	0.782169
H	3.565296	−1.473216	−1.553606
H	4.608853	0.653428	−0.800961

Monomer **17**

C	−1.592687	−0.976199	0.739634
C	−2.607191	−0.580126	−0.363813
C	−2.191990	0.825801	−0.722550
C	−1.034353	1.180639	−0.160438
C	−0.452223	0.056917	0.659863
H	−1.209055	−1.992028	0.632702
H	−3.641373	−0.630703	−0.008423
H	−2.550005	−1.236427	−1.241003
H	−2.777675	1.454856	−1.383753
H	−0.522344	2.125915	−0.281919
H	−0.080314	0.376471	1.633977
H	−2.065458	−0.903139	1.722180
O	0.677514	−0.566126	−0.036502
C	1.866993	0.061127	0.039957
O	2.045872	1.096774	0.637814
C	2.930045	−0.697656	−0.718025
H	2.978428	−1.732146	−0.370137
H	2.676292	−0.722037	−1.781210
H	3.892862	−0.208964	−0.580670

Monomer **18**

C	2.495502	−0.849339	−0.883248
C	1.434183	0.239517	−0.634639
C	1.975050	1.019645	0.536340
C	3.019417	0.402605	1.093075
C	3.383649	−0.880337	0.387624
H	3.095857	−0.564744	−1.750757
H	1.223141	0.854602	−1.510140
H	1.516784	1.940433	0.871840
H	3.551773	0.756000	1.969225
H	3.171750	−1.746768	1.026027
H	4.449385	−0.931305	0.142511
H	2.024331	−1.807392	−1.107252
O	0.179243	−0.432819	−0.281779
C	−0.918887	0.326041	−0.290975
O	−0.974088	1.502849	−0.565412
O	−1.958467	−0.443744	0.053324
C	−3.242482	0.218601	0.093449
H	−3.184162	1.054592	0.795242
H	−3.457931	0.626357	−0.897651
C	−4.271923	−0.809846	0.520003
H	−5.259620	−0.342271	0.560963
H	−4.313171	−1.640842	−0.188519
H	−4.038695	−1.210273	1.509677

Monomer **19**

C	−1.180218	0.281692	0.000002
C	−0.000289	1.216590	−0.000011
C	1.180081	0.282254	0.000014
C	0.734562	−0.991144	−0.000009
C	−0.734087	−0.991498	0.000009
H	−0.000455	1.879891	0.876258
H	−0.000447	1.879885	−0.876284
H	2.210181	0.611047	0.000019
H	1.346775	−1.883767	−0.000017

DCPD

C	1.057391	−0.674519	1.278643
C	1.096429	−1.117666	−0.174632
C	1.026417	1.142462	−0.150983
C	1.018640	0.664346	1.294188
C	1.915351	0.045006	−0.791119
H	1.899476	0.055409	−1.885898
H	2.948213	0.073137	−0.436538
C	−0.314856	−0.800822	−0.812560
C	−0.360743	0.771361	−0.790940

H	−2.210475	0.610000	0.000020
H	−1.345873	−1.884422	−0.000021

H	1.449415	−2.132084	−0.360532
H	1.324391	2.179191	−0.310598
H	0.969313	−1.339858	2.127677
H	0.900884	1.303021	2.160005
C	−1.545189	−1.212870	−0.051576
H	−1.808308	−2.251345	0.119651
C	−1.646007	1.159566	−0.012401
H	−1.432683	1.779069	0.867579
H	−2.336432	1.742525	−0.633578
C	−2.253059	−0.167556	0.382655
H	−3.167715	−0.236174	0.962268
H	−0.391622	1.174190	−1.805683
H	−0.321176	−1.202930	−1.832003

Ring-Opening Metathesis Polymerization (ROMP) of norbornene derivatives: All experiments were performed under the same conditions, including vial type, and stirrer shape and size. A 4 mL, screw-cap reaction vial was charged with 2.0 mmol of the norbornene derivative in CH_2Cl_2 in a glove box, after which it was added via syringe to a vial charged with the catalyst under inert conditions at room temperature. After 30 min, excess ethyl vinyl ether was added to terminate the reaction, and the reaction mixture was poured into methanol to precipitate the polymer.

Monomer **5**,[20] **6**,[21] **7**,[22] **8**,[23] and **9**[24] were synthesized following literature procedures.

***N*-methyl-7-oxabicyclo[2.2.1]hept-5-ene-2-*exo*,3-*exo*-dicarboximide** (**5**)

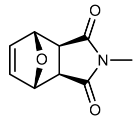

Crystalline white solid; ^{1}H NMR (500 MHz, $CDCl_3$): δ = 6.50 (s, 2H), 5.26 (s, 2H), 2.97 (s, 3H), 2.85 (s, 2H). The compound was identified by spectral comparison with literature data.[20]

***N*-methylbicyclo[2.2.1]hept-5-ene-2-*endo*,3-*endo*-dicarboximide** (**6**)

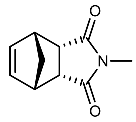

Crystalline white solid; ^{1}H NMR (500 MHz, $CDCl_3$): δ = 6.09 (s, 2H), 3.38 (s, 2H), 3.26 (s, 2H), 2.82 (s, 3H), 1.73 (d, J = 8.8 Hz, 1H), 1.54 (d, J = 8.8 Hz, 1H). The compound was identified by spectral comparison with literature data.[21]

***N*-benzylbicyclo[2.2.1]hept-5-ene-2-*endo*,3-*endo*-dicarboximide** (**7**)

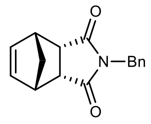

White solid; ^{1}H NMR (500 MHz, $CDCl_3$): δ = 7.28 (dt, J = 16.3, 8.0 Hz, 5H), 5.89 (s, 2H), 4.48 (s, 2H), 3.36 (s, 2H), 3.25 (s, 2H), 1.68 (d, J = 8.8 Hz, 1H), 1.50 (d, J = 8.8 Hz, 1H). The compound was identified by spectral comparison with literature data.[22]

***endo*-5,6-Bis(benzyloxymethyl)bicyclo[2.2.1]hept-2-ene** (**8**)

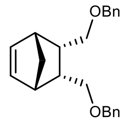

Colorless liquid; ^{1}H NMR (500 MHz, $CDCl_3$): δ = 7.40-7.23 (m, 10H), 6.04 (s, 2H), 4.43 (dd, J = 31.8, 12.0 Hz, 4H), 3.30 (dd, J = 9.0, 5.8 Hz, 2H), 3.04 (t, J = 8.9 Hz, 2H), 2.96 (s, 2H), 2.52 (s, 2H), 1.47 (d, J = 8.3 Hz, 1H), 1.32 (d, J = 8.3 Hz, 1H). The compound was identified by spectral comparison with literature data.[23]

***endo*-5,6-Bis(tert-butyldimethyloxymethyl)bicyclo[2.2.1]hept-2-ene** (**9**)

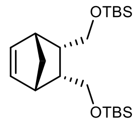

Colorless liquid; ^{1}H NMR (400 MHz, $CDCl_3$): δ = 6.09 (s, 2H), 3.46 (dd, J = 9.7, 5.3 Hz, 2H), 3.16 (t, J = 9.1 Hz, 2H), 2.89 (s, 2H), 2.32 (s, 2H), 1.42 (d, J = 7.9 Hz, 1H), 1.27 (d, J = 8.1 Hz, 1H), 0.87 (s, 18H), 0.00 (s, 12H); ^{13}C NMR (100 MHz, $CDCl_3$) δ = 135.3, 63.0, 48.9, 45.4, 44.3, 25.9, 18.2, -5.4; HRMS-EI (m/z) $[M]^+$ calcd for $C_{21}H_{42}O_2Si_2$, 382.2723; found, 382.2726.

ROMP of cyclopentene: All experiments were performed under the same conditions, including vial type, stirrer shape and size, and concentration ranges, at 0, –20, –40 °C. A 4 mL, screw-cap reaction vial was charged with 3.5 mg (0.00495 mmol) of the catalyst and 0.80 mL of purified dichloromethane in a glovebox. The vial was removed from the glovebox and cooled to the target temperature with continuous stirring (800 rpm), after which 0.20 mL (2.17 mmol) of cyclopentene was added using a 1.0 mL Hamilton glass syringe under an inert atmosphere. After 2 h, the reaction mixture was quenched with 0.10 mL (1.0 mmol) ethyl vinyl ether. The solution was stirred for additional 15 min then precipitated in methanol. The obtained rubber-like residue was washed with methanol and evaporated. The obtained polymer was dissolved in THF to obtain a 1.0 mg/mL polymer solution for GPC.

Supplementary Table 9. ROMP of cyclopentene catalyzed by **G-III** and **Ru-8** at various temperatures

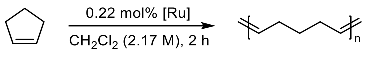

10

Entry	T (°C)	Catalyst	Time (h)	M_n (10^3)	PDI	Yield (%)
1	0	**G-III**	2	21.8	2.01	82
2	0	**Ru-8**	2	24.2	2.08	81
3	–20	**G-III**	2	6.0	1.17	17
4	–20	**G-III**	24	38.4	2.09	>99
5	–20	**Ru-8**	2	59.2	1.60	>99
6	–20	**Ru-8** + 2.0 equiv. Py	2	21.3	1.75	25
7	–40	**G-III**	2	-	-	N. R.
8	–40	**Ru-8**	2	28.8	2.10	>99

Polypentenamer (**poly-10**)

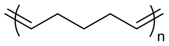

Colorless sticky gel; ^{1}H NMR (400 MHz, $CDCl_3$) δ = 5.30 (brs, 2H), 1.91 (brs, 4H), 1.44-1.22 (m, 2H). The compound was identified by spectral comparison with literature data.[25]

Monomers **11**,[26] **12**,[27] **15**,[28] **16**,[29] **17**,[30] and **18**[31] were synthesized by literature procedures.

Cyclopent-3-en-1-ylmethanol (**11**)

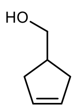

Colorless liquid; ^{1}H NMR (500 MHz, $CDCl_3$): δ = 5.66 (s, 2H), 3.57-3.54 (m, 2H), 2.56-2.37 (m, 3H), 2.11 (d, J = 8.8 Hz, 2H). The compound was identified by spectral comparison with literature data.[26]

(Cyclopent-3-en-1-yloxy)methylbenzene (**12**)

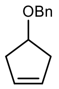

Colorless liquid; ^{1}H NMR (300 MHz, $CDCl_3$): δ = 7.54-7.25 (m, 5H), 5.72 (s, 2H), 4.53 (s, 2H), 4.32 (ddd, J = 10.3, 6.9, 3.5 Hz, 1H), 2.67-2.43 (m, 4H). The compound was identified by spectral comparison with

literature data.[27]

Cyclopent-2-en-1-ylbenzene (**15**)

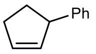

Colorless liquid; ^{1}H NMR (500 MHz, $CDCl_3$): δ = 7.40 (dd, J = 12.9, 6.3 Hz, 2H), 7.31 (dt, J = 27.3, 13.7 Hz, 3H), 6.06 (s, 1H), 5.91 (s, 1H), 4.01 (brs, 1H), 2.68-2.58 (m, 1H), 2.57-2.44 (m, 2H), 1.92-1.79 (m, 1H). The compound was identified by spectral comparison with literature data.[32]

Cyclopent-2-en-1-ylmethylbenzene (**16**)

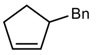

Colorless liquid; ^{1}H NMR (500 MHz, $CDCl_3$): δ = 7.34-7.24 (m, 2H), 7.19 (dt, J = 3.3, 2.6 Hz, 3H), 5.77-5.72 (m, 1H), 5.70-5.61 (m, 1H), 3.06-2.90 (m, 1H), 2.64 (ddd, J = 37.7, 13.4, 7.6 Hz, 2H), 2.41-2.22 (m, 2H), 2.06-1.93 (m, 1H), 1.57-1.47 (m, 1H). The compound was identified by spectral comparison with literature data.[29]

Cyclopent-2-en-1-ylacetate (**17**)

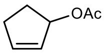

Colorless liquid; ^{1}H NMR (500 MHz, $CDCl_3$): δ = 6.16-6.06 (m, 1H), 5.87-5.78 (m, 1H), 5.74-5.65 (m, 1H), 2.58-2.44 (m, 1H), 2.36-2.21 (m, 2H), 2.03 (s, 3H), 1.85-1.75 (m, 1H). The compound was identified by spectral comparison with literature data.[30]

Cyclopent-2-en-1-ylethylcarbonate (**18**)

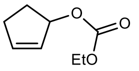

Colorless liquid; ^{1}H NMR (400 MHz, $CDCl_3$): δ = 6.12 (s, 1H), 5.85 (s, 1H), 5.60 (s, 1H), 4.21-4.13 (m, 2H), 2.62-2.42 (m, 1H), 2.40-2.18 (m, 2H), 1.95-1.82 (m, 1H), 1.30-1.26 (m, 3H). The compound was identified by spectral comparison with literature data.[31]

Monomers **13**[33] and **14**[34] were synthesized by modified literature procedures, as detailed below.

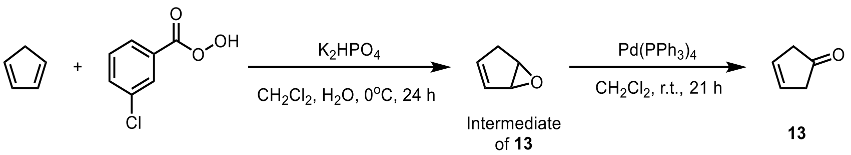

Monomer **13**: Cyclopentadiene (3.5 mL, 41.3 mmol), *m*-CPBA (70%, 10.2 g, 41.3 mmol) and K_2HPO_4 (7.2 g, 41.4 mmol) were stirred in CH_2Cl_2 (300 mL) and water (2 mL) for 18 h. The solution was then successively washed with water and brine, and dried over $MgSO_4$. The solvent was removed by distillation at atmospheric pressure to give 6-oxabicyclo[3.1.0]hex-2-ene (Intermediate of **13**) (1.7 g, 50%), which was subsequently reacted as described in the literature procedure.[33]

Cyclopent-3-en-1-one (**13**)

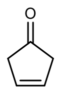

Colorless liquid; ^{1}H NMR (500 MHz, $CDCl_3$): δ = 6.07 (s, 2H), 2.86 (s, 4H). The compound was identified by spectral comparison with literature data.[33]

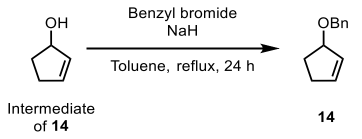

Monomer **14**: Sodium hydride (1.2 g, 28 mmol, 60% suspension in mineral oil) and 30 mL of dry toluene were stirred in a three-necked flask under the exclusion of moisture with cooling. After extensive flushing with nitrogen, cyclopent-3-en-1-ol[31] (1.8 g, 21 mmol) was added with a dropping funnel over 1 h. As gas ceased to evolve, a solution of benzyl chloride (2.9 mL, 25 mmol) in toluene was added dropwise, after which the reaction mixture was refluxed for 24 h, resulting in a brownish reaction mixture. Excess sodium hydride was inactivated by the careful addition of methanol in toluene. The mixture was filtered, and the red organic phase was washed with water, dried over sodium sulfate, and the solvent evaporated *in vacuo*. Final vacuum distillation afforded a colorless liquid (1.3 g, 35%).

(Cyclopent-2-en-1-yloxy)methylbenzene (**14**)

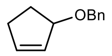

Colorless liquid; ^{1}H NMR (500 MHz, $CDCl_3$): δ = 7.45-7.20 (m, 5H), 6.08-6.00 (m, 1H), 5.94-5.85 (m, 1H), 4.68 (brs, 1H), 4.61-4.45 (m, 2H), 2.57-2.46 (m, 1H), 2.38-2.24 (m, 1H), 2.20-2.07 (m, 1H), 1.86 (qd, J = 8.6, 4.2 Hz, 1H). The compound was identified by spectral comparison with literature data.[34]

ROMP of low-strained monomers: All experiments were performed under the same conditions including vial type, stirrer shape and size, and concentration ranges, at 0, –20, –40, and –60 °C. A 20 mL screw-cap reaction tube was charged with 7.2 mg (0.010 mmol) of one of the seven catalyst and 2.0 mmol of the low-strained monomer in a glovebox. The reaction mixture was removed from the glovebox and cooled to the target temperature with continuous stirring (800 rpm). After 24 h, the reaction mixture was quenched with 1 mL of a 0.10 M solution of ethyl vinyl ether (1.0 mmol) in dichloromethane. The solution was stirred for an additional 1 h, and then precipitated in methanol. The obtained rubber-like residue was washed with methanol and evaporated. The obtained polymer was dissolved in THF to prepare a 1.0 mg/mL polymer solution for GPC.

Poly(cyclopent-3-en-1-ylmethanol) (**poly-11**)

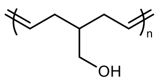

Colorless sticky gel; ^{1}H NMR (500 MHz, DMSO-d_6) δ = 5.34 (brs, 2H), 4.33 (brs, 1H), 3.24 (brs, 2H), 1.92 (d, J = 28.2 Hz, 4H), 1.42 (s, 1H); ^{13}C NMR (125 MHz, DMSO-d_6) δ = 130.0, 63.4, 41.3, 33.7.

Poly[(cyclopent-3-en-1-yloxy)methylbenzene] (**poly-12**)

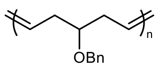

Colorless sticky gel; ^{1}H NMR (300 MHz, $CDCl_3$) δ = 7.67-7.17 (m, 5H), 5.53 (brs, 2H), 4.53 (brs, 2H), 3.45 (brs, 1H), 2.28 (brs, 4H); ^{13}C NMR (125 MHz, $CDCl_3$) δ = 138.9, 128.8, 128.2, 127.6, 127.4, 78.9, 70.9, 37.0.

Poly(cyclopent-3-en-1-one) (**poly-13**)

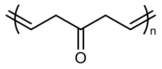

Insoluble solid; ^{13}C NMR (125MHz, CPTOSS 5 kHz) δ = 209. 5, 128.5, 45.2, 41.2. The compound was identified by spectral comparison with literature data.[35]

Poly[(cyclopent-2-en-1-yloxy)methylbenzene] (**poly-14**)

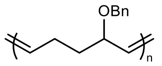

Colorless sticky gel; ^{1}H NMR (300 MHz, $CDCl_3$) δ = 7.26 (brs, 5H), 5.53 (brs, 1H), 5.28 (brs, 1H), 4.49 (brs, 1H), 4.27 (brs, 1H), 3.69 (d, J = 37.8 Hz, 1H), 2.08 (brs, 2H), 1.61 (brs, 2H); ^{13}C NMR (125 MHz, $CDCl_3$) δ = 138.9, 133.9, 130.8, 128.3, 127.8, 127.4, 79.4, 69.7, 35.5, 28.3.

Poly(cyclopent-2-en-1-ylbenzene) (**poly-15**)

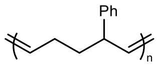

Colorless sticky gel; ^{1}H NMR (400 MHz, $CDCl_3$) δ = 7.48-6.83 (m, 5H), 5.76-4.98 (m, 2H), 3.17 (brs, 1H), 1.91 (brs, 2H), 1.68 (brs, 2H); ^{13}C NMR (100 MHz, $CDCl_3$) δ = 145.2, 130.2, 129.7, 128.3, 127.5, 125.9, 48.1 (d, J = 25.2 Hz), 35.6, 30.5.

Poly(cyclopent-2-en-1-ylmethylbenzene) (**poly-16**)

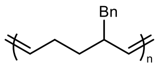

Colorless sticky gel; ^{1}H NMR (500 MHz, $CDCl_3$) δ = 7.62-6.71 (m, 5H), 5.40-4.89 (m, 2H), 2.49 (brs, 2H), 2.31-1.56 (m, 3H), 1.51-0.99 (m, 2H); ^{13}C NMR (125 MHz, $CDCl_3$) δ = 140.8, 133.7, 130.7, 129.3, 127.9, 125.6, 43.8, 42.3, 34.5, 30.2.

Poly(cyclopent-2-en-1-ylacetate) (**poly-17**)

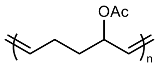

Colorless sticky gel; ^{1}H NMR (500 MHz, $CDCl_3$) δ = 5.77-5.53 (m, 1H), 5.50-5.30 (m, 1H), 5.25-5.06 (m, 1H), 2.33-2.16 (m, 1H), 2.11-1.86 (m, 4H), 1.79-1.43 (m, 2H); ^{13}C NMR (125 MHz, $CDCl_3$) δ = 170.2, 133.0, 128.7, 74.0, 33.7, 27.9, 21.3.

Poly(cyclopent-2-en-1-ylethylcarbonate) (**poly-18**)

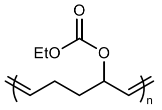

Colorless sticky gel; ^{1}H NMR (500 MHz, $CDCl_3$) δ = 5.82-5.64 (m, 1H), 5.40 (dd, J = 15.3, 7.5 Hz, 1H), 4.16 (q, 2H), 2.07 (brs, 2H), 1.75 (d, J = 7.0 Hz, 1H), 1.63 (dd, J = 14.3, 7.6 Hz, 1H), 1.29 (t, J = 7.1 Hz, 3H); ^{13}C NMR (125 MHz, $CDCl_3$) δ = 154.4, 133.7, 128.3, 78.0, 63.7, 33.6, 27.7, 14.2.

Supplementary Table 10. ROMP of **11** at various reaction temperatures

Monomer	Cat.	T (°C)	M_n (10^3)	PDI	Yield (%)*
		r.t.	Insol. Gel	-	59
	**G-II**	0	Insol. Gel	-	47
		–20	Insol. Gel	-	7
		r.t.	Insol. Gel	-	36
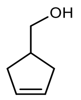 **11**	**G-III**	0	Insol. Gel	-	53
		–20	Insol. Gel	-	32
		–40	-	-	N. R.
		r.t.	Insol. Gel	-	46
	**Ru-8**	0	Insol. Gel	-	46
		–20	Insol. Gel	-	41
		–40	Insol. Gel	-	43

* The reaction mixture was quenched with 1 mL of 0.10 M solution of ethyl vinyl ether (1.0 mmol) in methanol. The solution was stirred for additional 1 h and then precipitated in dichloromethane.

**The obtained polymer rapidly became a soft gel and lost its solubility.

Supplementary Table 11. ROMP of **12** at various reaction temperatures

Monomer	Cat.	T (°C)	M_n (10^3)	PDI	Yield (%)
		r.t.	3.9	1.4	42
	**G-II**	0	5.0	1.5	34
		–20	5.0	2.3	17
		r.t.	4.7	1.8	50
		0	5.7	2.1	60
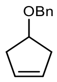 **12**	**G-III**	–20	4.5	1.8	17
		–40	-	-	N. R.
		–60	-	-	N. R.
		r.t.	4.6	1.6	52
		0	5.6	2.2	64
	**Ru-8**	–20	5.3	2.9	48
		–40	9.2	2.8	69
		–60	5.6	1.6	71*

***Solvent condition:** Dichloromethane (2.0 M)

Supplementary Table 12. ROMP of **13** at various reaction temperatures

Monomer	Cat.	T (°C)	M_n (10^3)	PDI	Yield (%)
		r.t.	-	-	N. R.
	**G-II**	0	-	-	N. R
		–20	-	-	N. R
		r.t.	Insol. Solid	-	24
		0	Insol. Solid	-	38
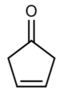	**G-III**	–20	Insol. Solid	-	22
		–40	-	-	N. R.
**13**		–60	-	-	N. R.
		r.t.	Insol. Solid	-	34
		0	Insol. Solid	-	77
	**Ru-8**	–20	Insol. Solid	-	66
		–40	Insol. Solid	-	56
		–60	Insol. Solid	-	22

Supplementary Table 13. ROMP of **14** at various reaction temperatures

Monomer	Cat.	T (°C)	M_n (10^3)	PDI	Yield (%)
		r.t.	Insol. Gel	-	7
	**G-II**	0	Insol. Gel	-	Gel.
		–20	Insol. Gel	-	12
		r. t.	Insol. Gel	-	40
		0	Insol. Gel	-	59
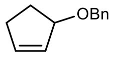	**G-III**	–20	Insol. Gel	-	17
		–40	-	-	N. R.
**14**		–60	-	-	N. R.
		r. t.	Insol. Gel	-	40
		0	Insol. Gel	-	55
	**Ru-8**	–20	Insol. Gel	-	41
		–40	Insol. Gel	-	61
		–60	109.4	1.4	71*

*Solvent condition: Dichloromethane (2.0 M)

Supplementary Table 14. ROMP of **15** at various reaction temperatures

Monomer	Cat.	T (°C)	M_n (10^3)	PDI	Yield (%)
		r.t.	-	-	N. R.
	**G-II**	0	-	-	N. R.
		–20	-	-	N. R.
		r.t.	-	-	N. R.
		0	17.5	2.26	26
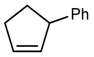 **15**	**G-III**	–20	N. D.	N. D.	16
		–40	-	-	N. R.
		–60	-	-	N. R.
		r.t.	-	-	N. R.
		0	10.0	2.23	36
	**Ru-8**	–20	8.02	2.41	43
		–40	17.9	2.58	63
		–60	12.2	3.00	48

*N.D., not detected due to the small molecular weight.

Supplementary Table 15. ROMP of **16** at various reaction temperatures

Monomer	Cat.	T (℃)	M_n (10^3)	PDI	Yield (%)
		r.t.	-	-	N. R.
	**G-II**	0	-	-	Trace
		–20	-	-	N. R.
		r.t.	-	-	N. R.
		0	15.9	2.66	32
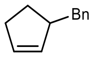 **16**	**G-III**	–20	-	-	Trace
		–40	-	-	N. R.
		–60	-	-	N. R.
		r.t.	-	-	N. R.
		0	18.8	2.26	36
	**Ru-8**	–20	34.0	2.38	46
		–40	62.8	1.64	55
		–60	13.2	2.58	22

Supplementary Table 16. ROMP of **17** at various reaction temperatures

Monomer	Cat.	T (℃)	M_n (10^3)	PDI	Yield (%)
	**G-II**	r.t.	-	-	N. R.
		0	-	-	N. R.
		–20	-	-	N. R.
	**G-III**	r.t.	-	-	N. R.
		0	13.4	1.83	22
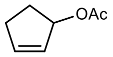 **17**		–20	13.3	1.46	17
		–40	-	-	N. R.
		–60	-	-	N. R.
	**Ru-8**	r.t.	-	-	N. R.
		0	23.0	1.76	24
		–20	38.5	1.36	48
		–40	70.0	1.38	71
		–60	-	-	Trace

*The obtained polymer was precipitated in hexane.

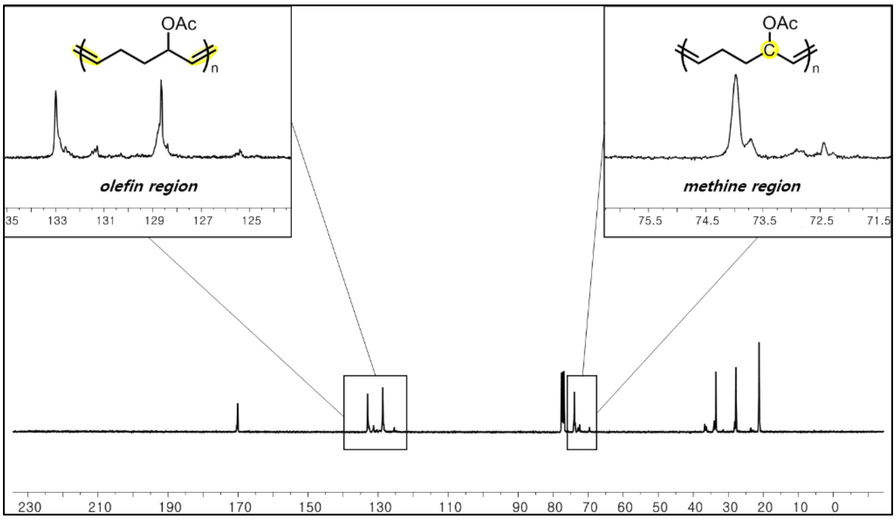

Supplementary Figure 9. ^{13}C NMR spectra of **poly-17**

Supplementary Table 17. ROMP of **18** at various reaction temperatures

Monomer	Cat.	T (°C)	M_n (10^3)	PDI	Yield (%)
		r.t.	-	-	N. R.
	**G-II**	0	-	-	N. R.
		–20	-	-	N. R.
		r.t.	-	-	N. R.
		0	N. D.	N. D.	13
	**G-III**	–20	10.4	1.3	16
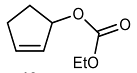 **18**		–40	-	-	N. R.
		–60	-	-	N. R.
		r.t.	-	-	N. R.
		0	26.0	1.6	37
	**Ru-8**	–20	39.0	1.8	53
		–40	54.2	1.7	66
		–60	68.6	1.7	48

*N.D., not detected due to the low molecular weight.

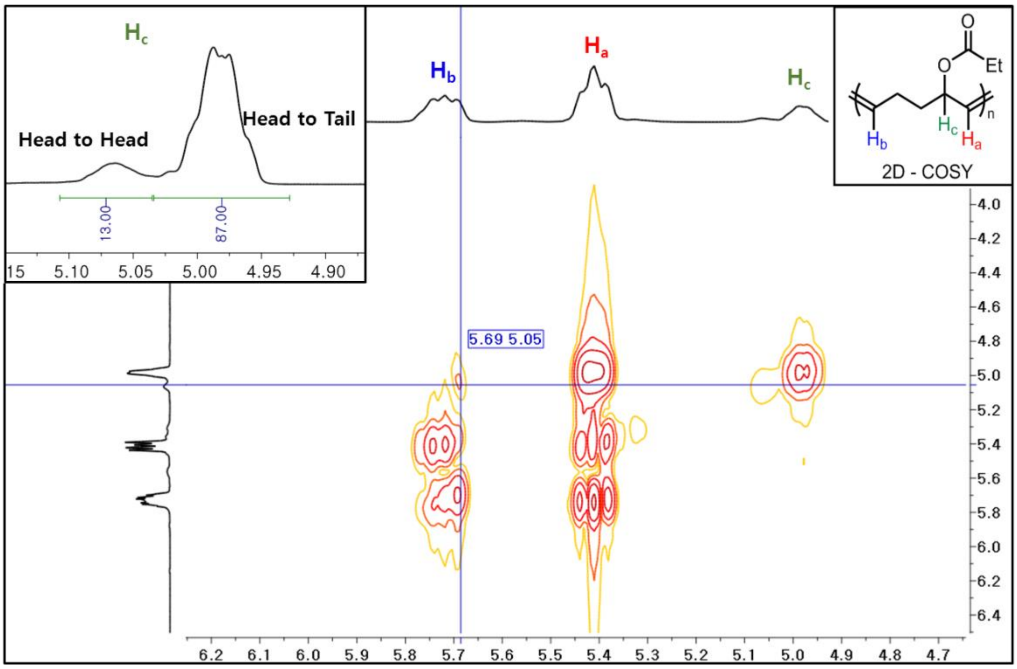

Supplementary Figure 10. ^{1}H–^{1}H COSY NMR spectra and regioregularity of **poly-18**

ROMP of cyclopentadiene: A 25 mL long-neck Schlenk reaction tube was charged with 7.1 mg (0.010 mmol) of the **Ru-8** catalyst in a glovebox. The reaction tube was removed from the glovebox and cooled to the target temperature (–40 °C) with continuous stirring (800 rpm). A 170 µL (2.0 mmol) sample of cyclopentadiene prepared by thermolysis was rapidly added by a 250-µL Hamilton glass syringe under an inert atmosphere in a glovebox. After 4 h, the reaction mixture was quenched with 0.10 mL (1.0 mmol) ethyl vinyl ether, after which 5 mL of $CDCl_3$ was added. The solution was stirred for an additional few minutes to ensure the dissolution of the obtained polymer. An appropriate aliquot was removed from the solution and further diluted with $CDCl_3$ in an NMR tube to determine the conversion, or the obtained polymer was dissolved in THF to produce a 1.0 mg/mL polymer solution for GPC. The poly(cyclopentadiene) (**poly-CPD**) rapidly becomes entangled and loses its solubility when dried or precipitated in a polar solvent.

Poly-CPD

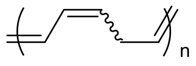

Red color in crude mixture (entangled after drying); ^{1}H NMR (500 MHz, $CDCl_3$) δ = 6.65-5.82 (m, 2H), 5.79-5.10 (m, 2H), 3.14-2.51 (m, 2H); ^{13}C NMR (125 MHz, $CDCl_3$) δ = 131.1, 130.7, 129.2, 35.5.

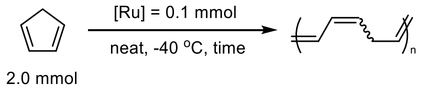

Supplementary Table 18. ROMP of cyclopentadiene catalyzed by **G-II**, **G-III** and **Ru-8**

Cat.	Time	M_n (10^3)	PDI	Conv. (%)
**Ru-8**	6	8.0	1.49	85
**G-II**	6	-	-	N. R.
**G-III**	6	-	-	N. R.
**G-II**	24	-	-	N. R.
**G-III**	24	-	-	N. R.

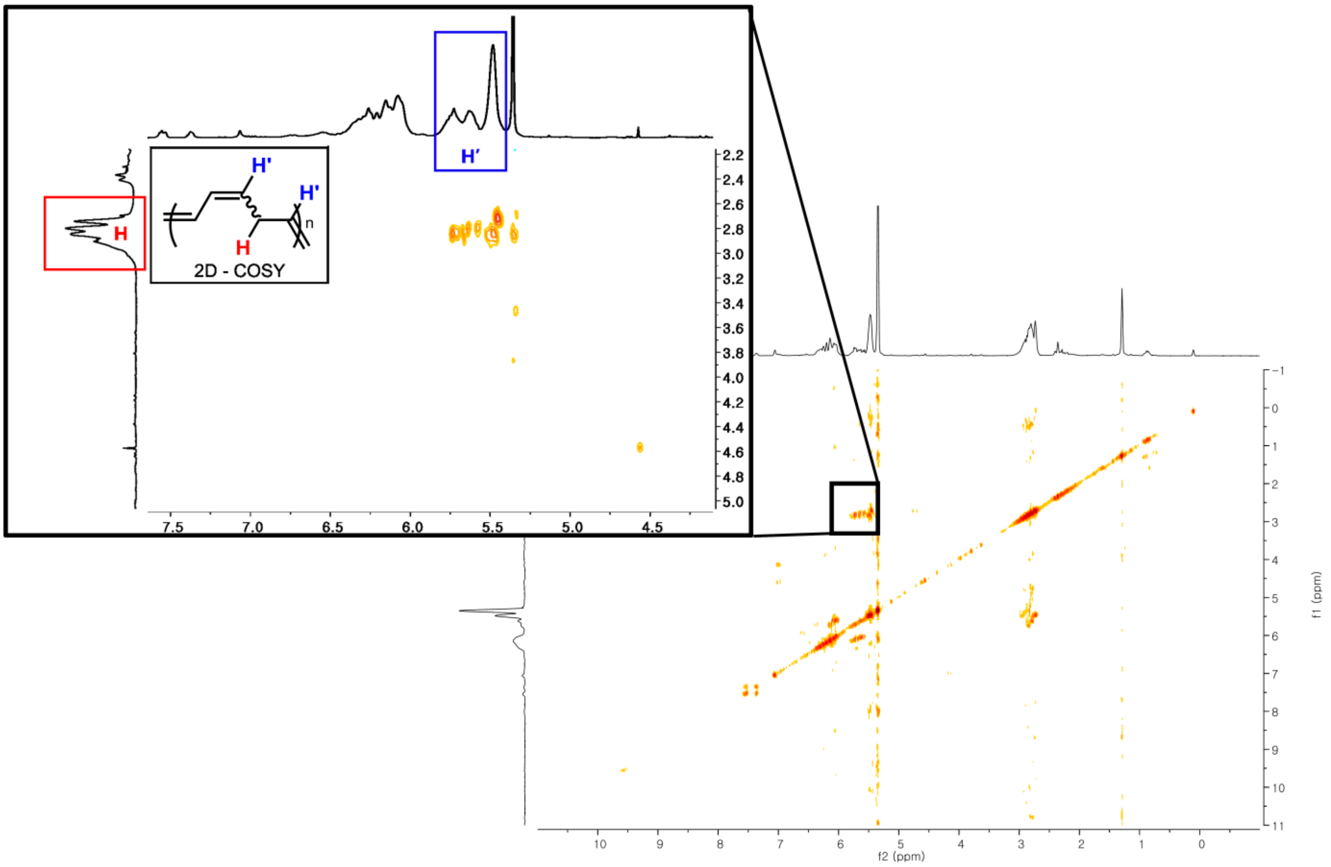

Supplementary Figure 11. ^1H–^1H COSY NMR spectra of **poly-CPD**

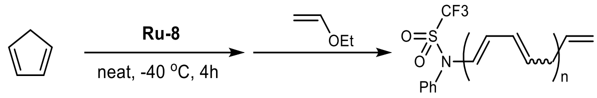

Monomer (cyclopentadiene) to Initiator (Ru-8) ratio = 5:1

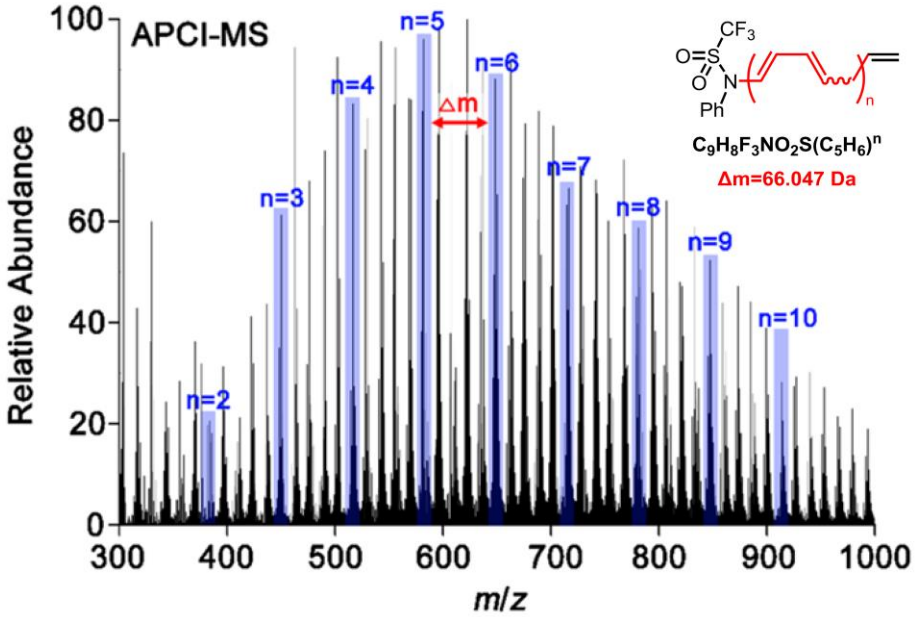

n	Calculated *m/z* of $[M+H]^+$ ion	Observed *m/z* of $[M+H]^+$ ion
1	318.078	318.0929
2	384.125	384.1474
3	450.172	450.1969
4	516.219	516.2457
5	582.266	582.2930
6	648.313	648.3425
7	714.36	714.3917
8	780.407	780.4410
9	846.454	846.4900
10	912.501	912.5370

Supplementary Figure 12. APCI-MS spectrum of CPD Oligomers. The major ion distribution comprises singly charged CPD ions separated by 66 Da, which corresponds to C_5H_6 repeating units. Other distributions anticipated to be from fragmentations of the oligomers are also shown. These fragments include the products corresponding to the loss of carbon (-12 Da), carbene (-14 Da), or methane (-16 Da)

Hydrogenation of poly-CPD: The crude **poly-CPD** was dissolved in 4 mL of THF without any isolation. The mixture was transferred to an autoclave and 10 wt% of Pd/C (13.2 mg) was added. Hydrogenation was achieved at 80 °C at a H_2-pressure of 30 bar and 80 °C for 12 h. At the completion of the reaction, the polymer was separated by filtration and dried under vacuum to give 107.6 mg of polyethylene (95% yield).

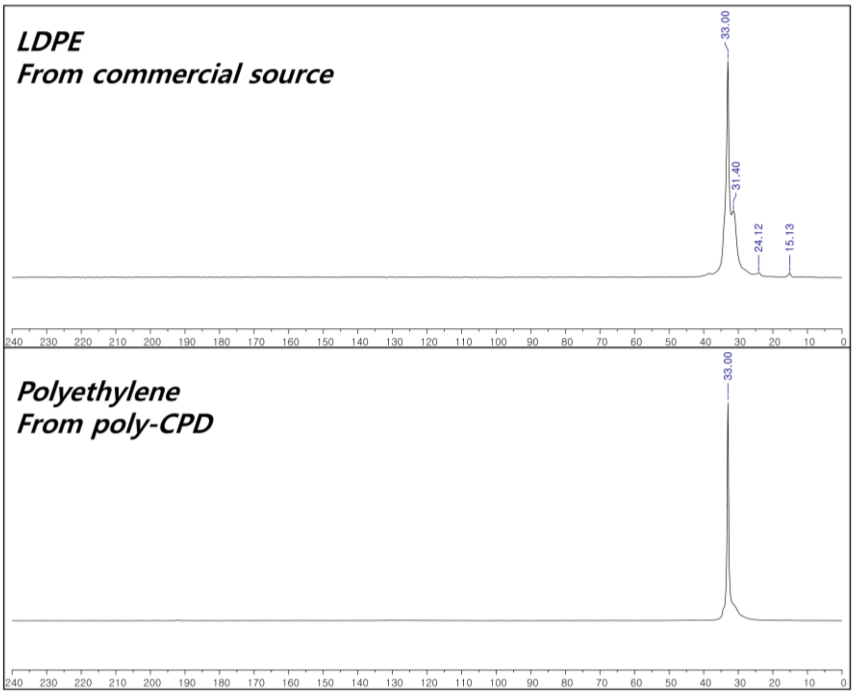

Supplementary Figure 13. Comparison of solid state ^{13}C NMR of polyethylene

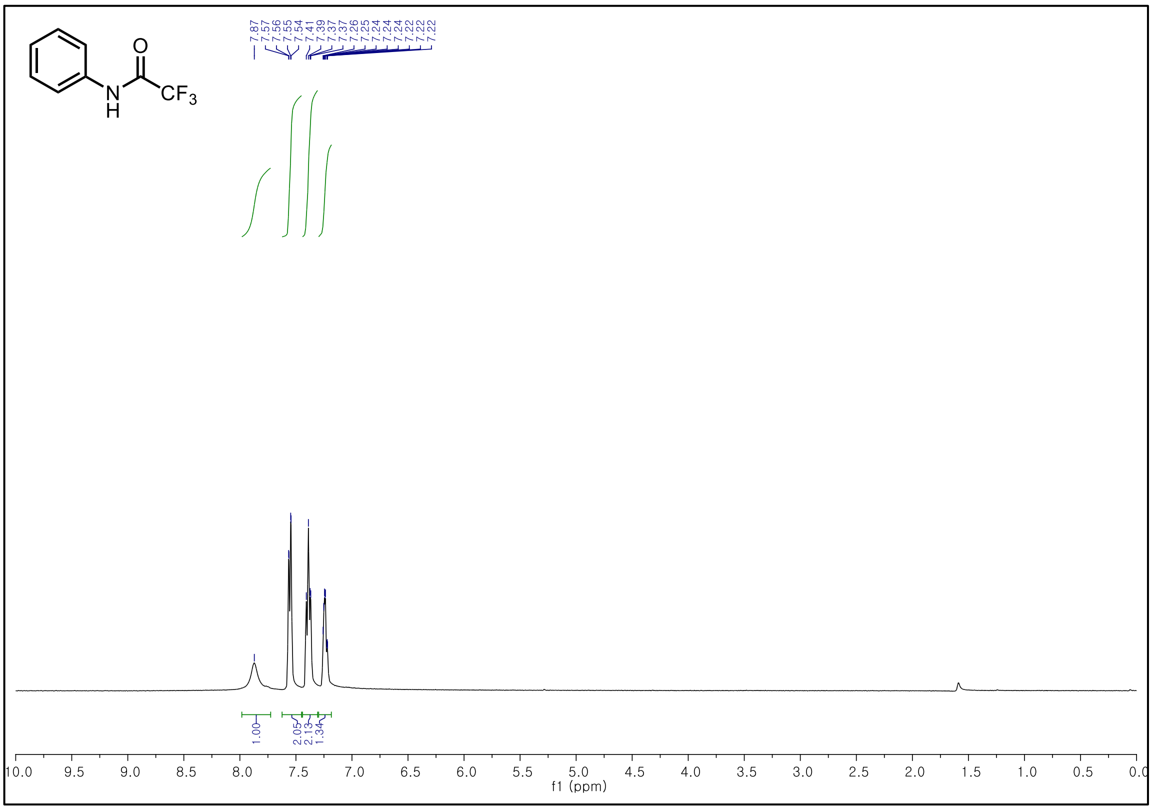

Supplementary Figure 14. ^{1}H NMR (**A1**) ($CDCl_3$, 300 MHz)

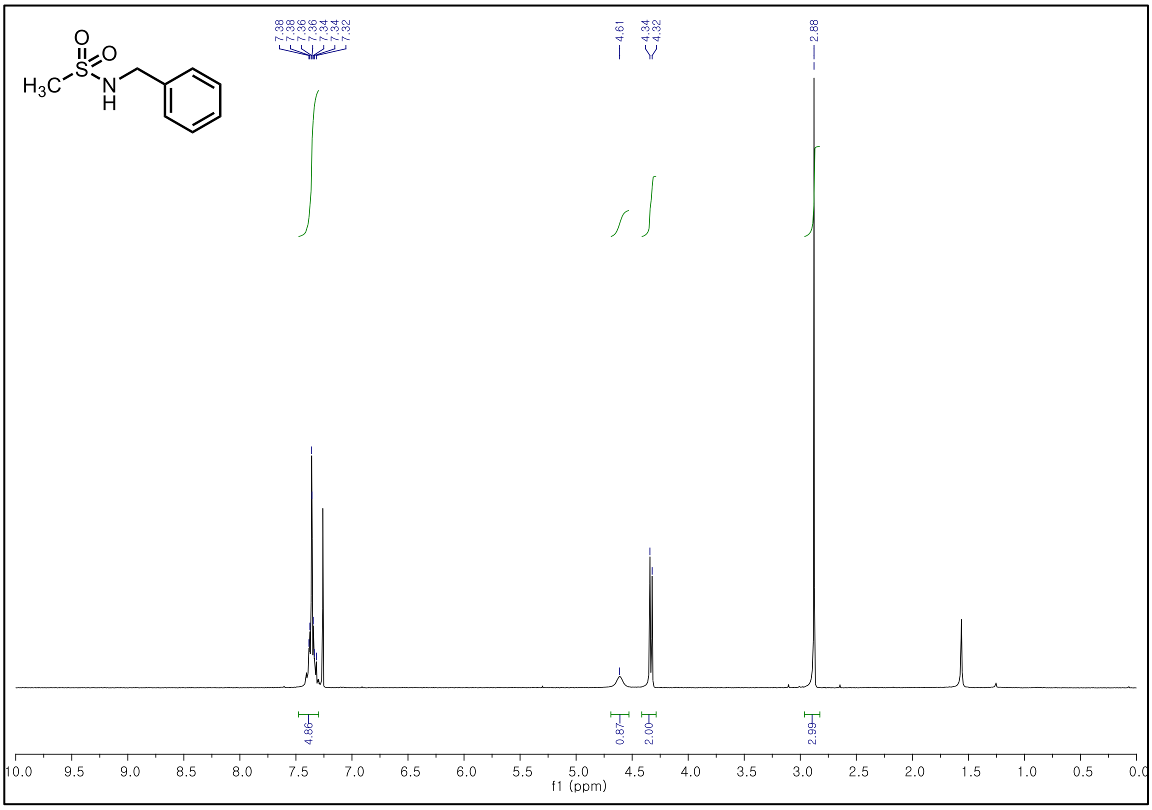

Supplementary Figure 15. ^{1}H NMR (**SA1**) ($CDCl_3$, 300 MHz)

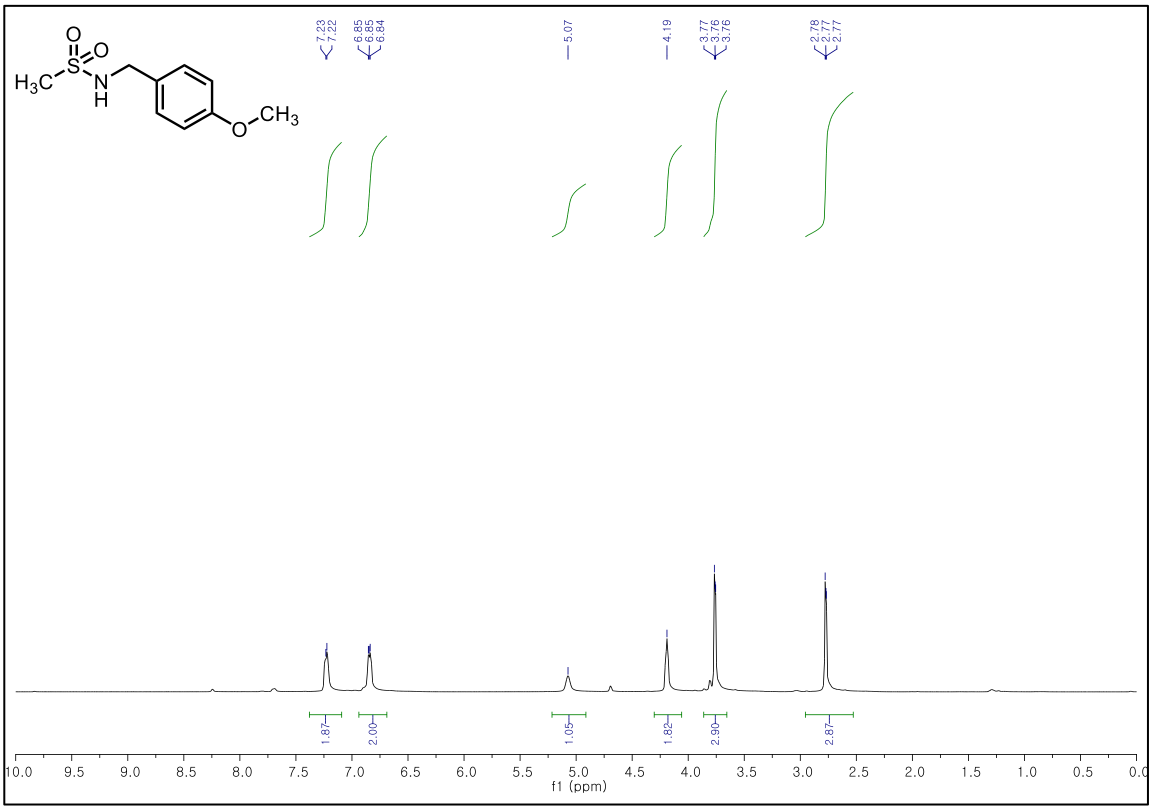

Supplementary Figure 16. ^{1}H NMR (**SA2**) ($CDCl_3$, 400 MHz)

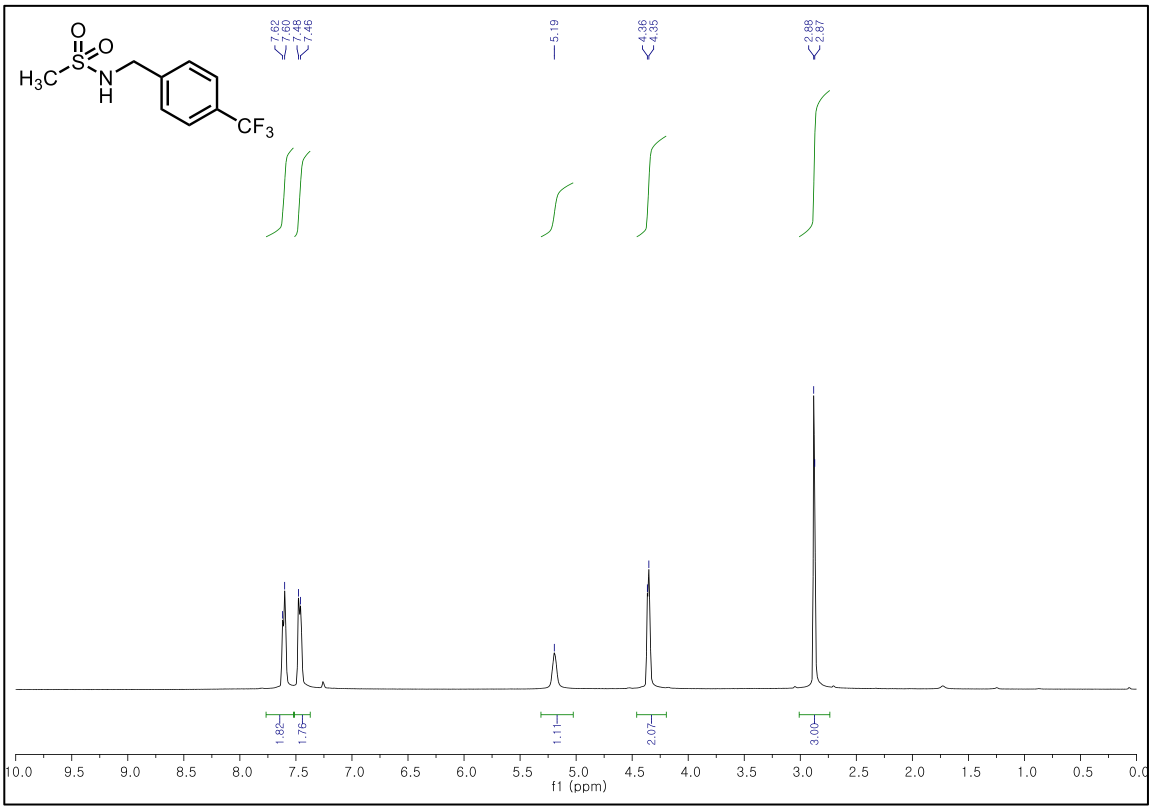

O
S
O
H_3C
N
H
CF_3
7.62
7.60
7.48
7.46
5.19
4.36
4.35
2.88
2.87
1.82
1.76
1.11
2.07
3.00
10.0 9.5 9.0 8.5 8.0 7.5 7.0 6.5 6.0 5.5 5.0 4.5 4.0 3.5 3.0 2.5 2.0 1.5 1.0 0.5 0.0
f1 (ppm)

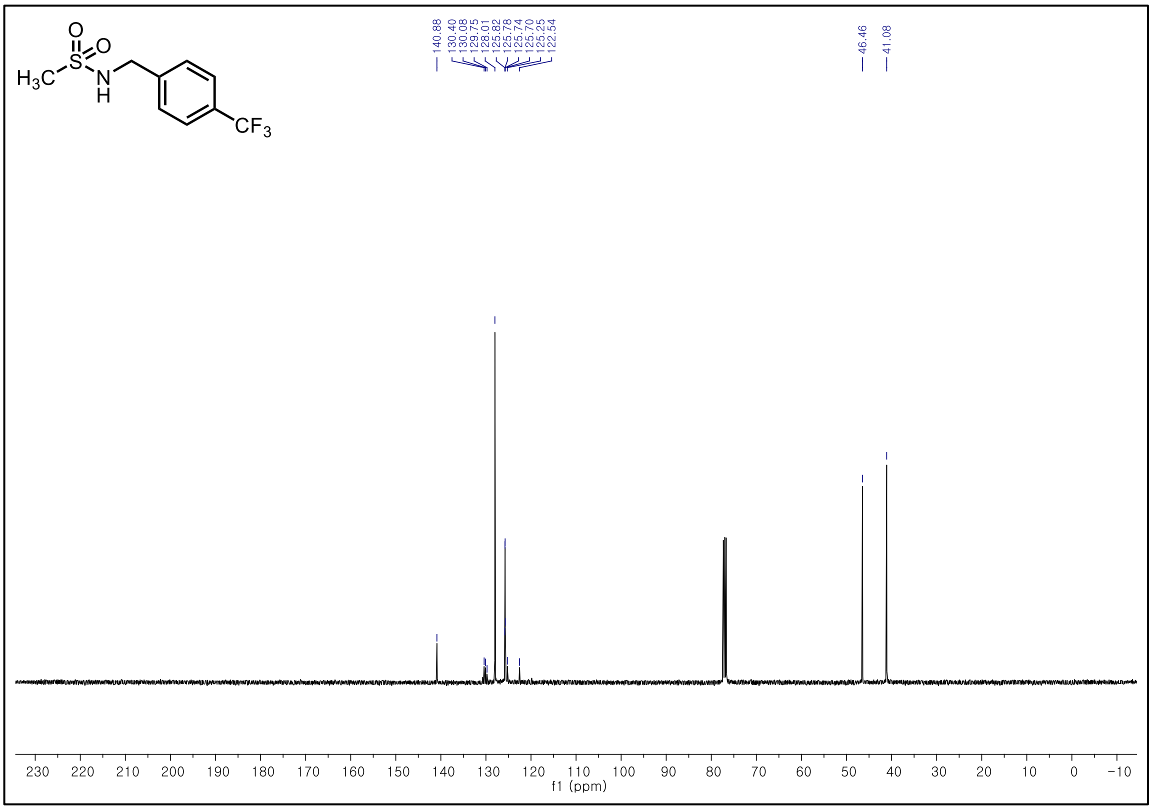

O
S
O
H_3C
N
H
CF_3
140.88
130.40
130.08
129.75
128.01
125.82
125.78
125.74
125.70
125.25
122.54
46.46
41.08
230 220 210 200 190 180 170 160 150 140 130 120 110 100 90 80 70 60 50 40 30 20 10 0 −10
f1 (ppm)

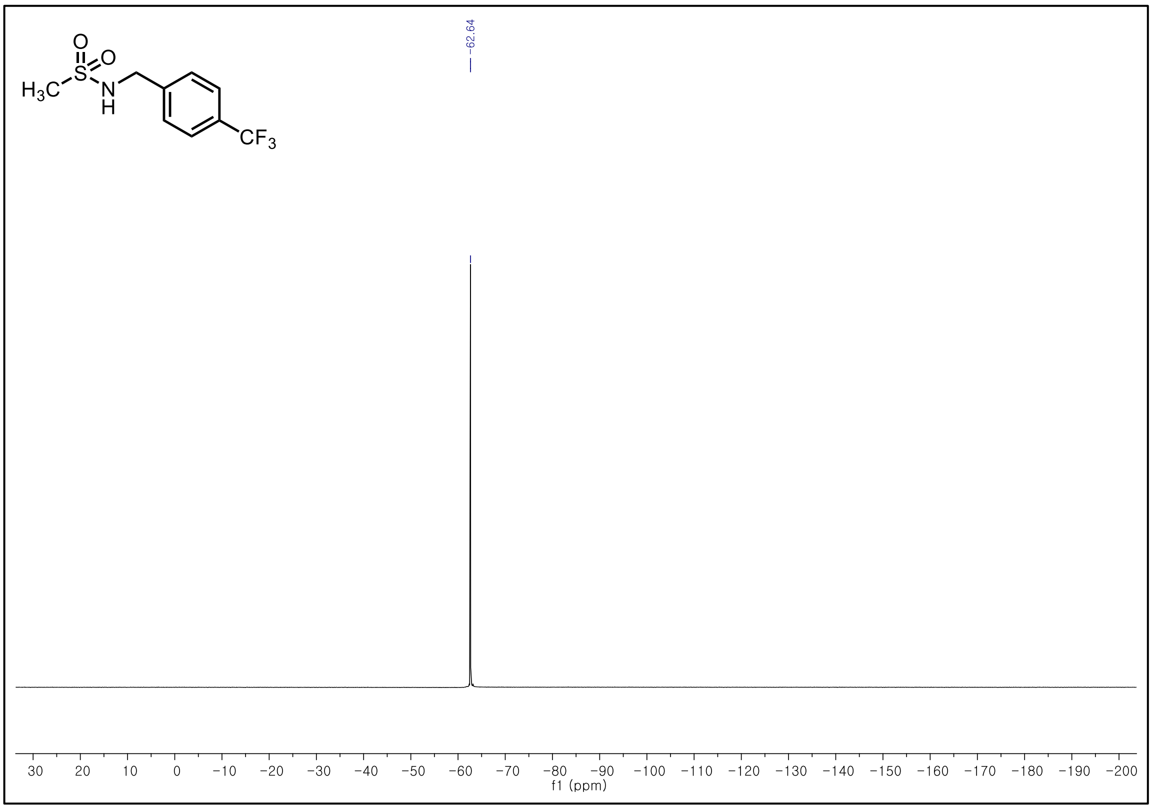

Supplementary Figure 17. ^{1}H NMR (**SA3**) ($CDCl_3$, 400 MHz), ^{13}C NMR (**SA3**) ($CDCl_3$, 100 MHz), and ^{19}F NMR (**SA3**) ($CDCl_3$, 376 MHz)

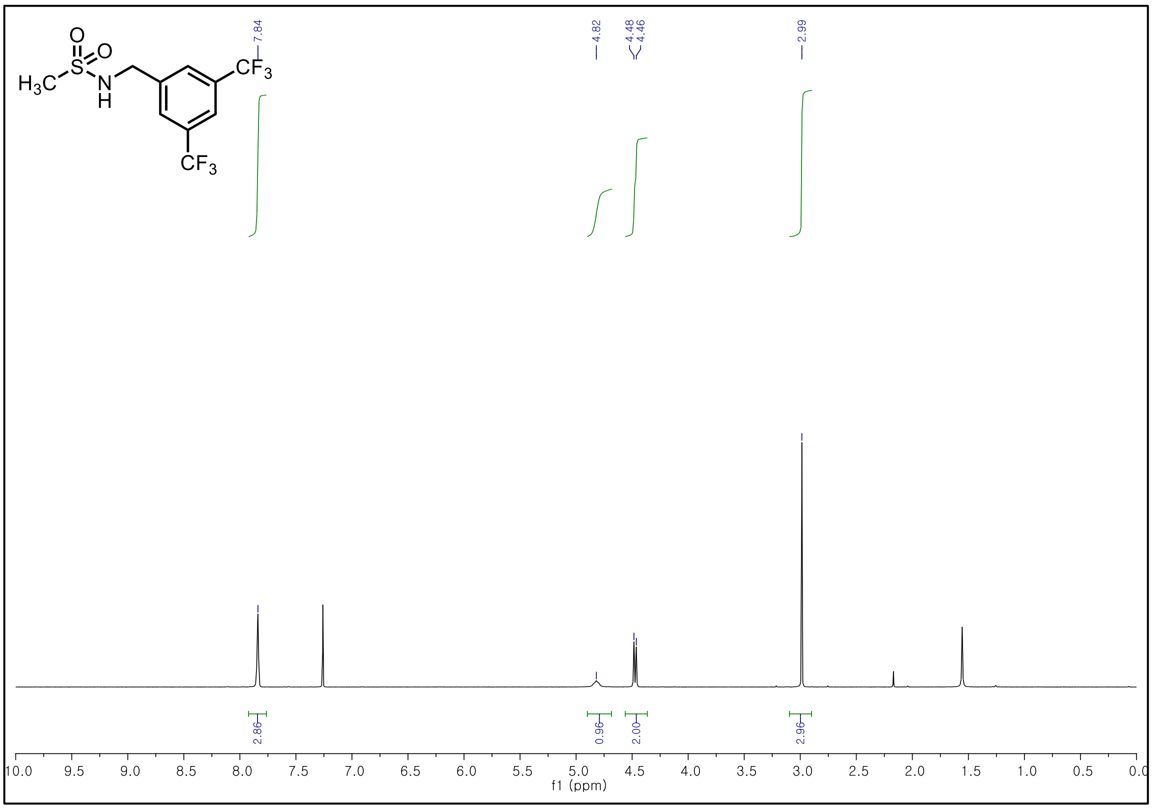

7.84
4.82
4.48
4.46
2.99
2.86
0.96
2.00
2.96
10.0 9.5 9.0 8.5 8.0 7.5 7.0 6.5 6.0 5.5 5.0 4.5 4.0 3.5 3.0 2.5 2.0 1.5 1.0 0.5 0.0
f1 (ppm)

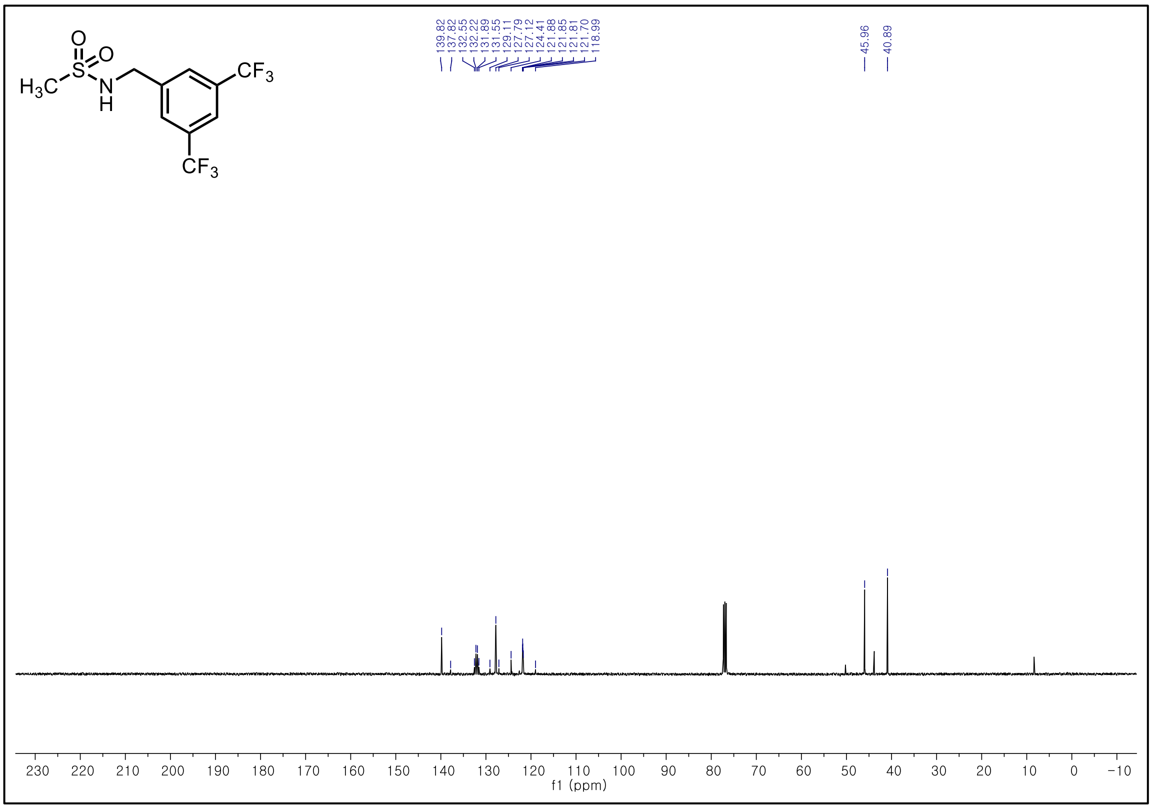

139.82
137.82
132.55
132.22
131.89
131.55
129.11
127.79
127.12
124.41
121.88
121.85
121.81
121.70
118.99
45.96
40.89
230 220 210 200 190 180 170 160 150 140 130 120 110 100 90 80 70 60 50 40 30 20 10 0 -10
f1 (ppm)

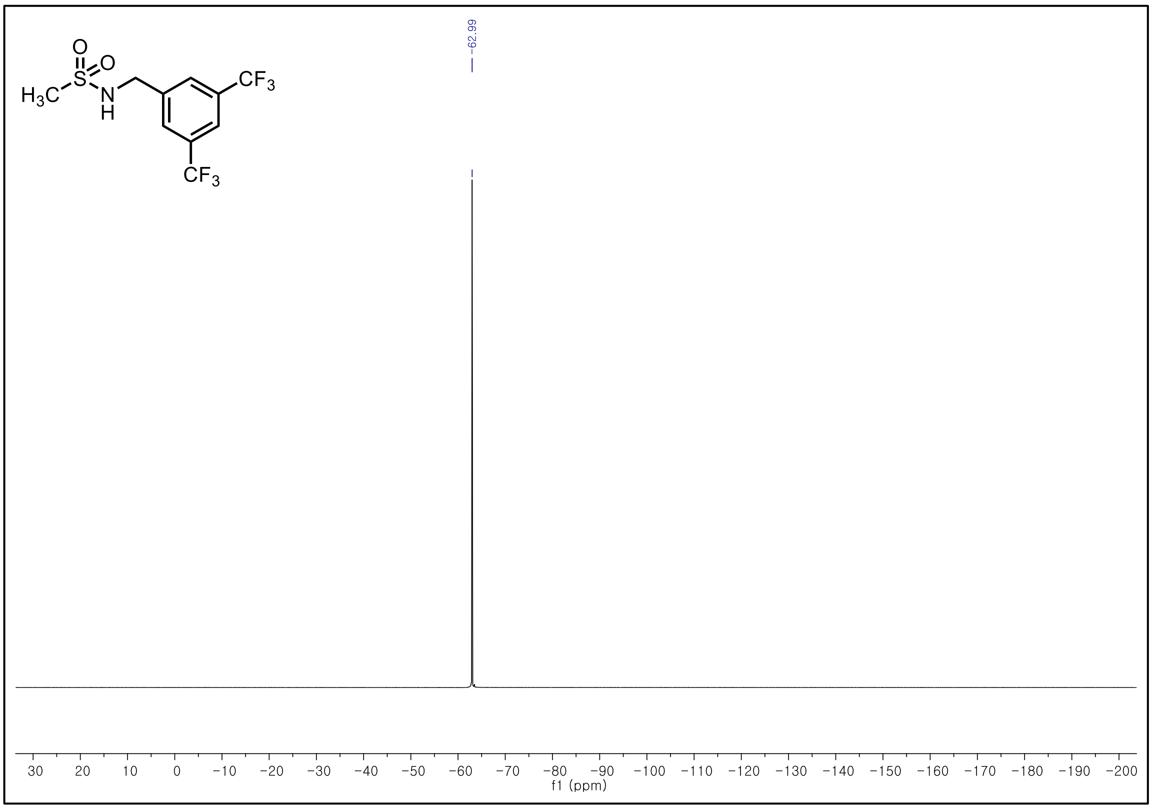

Supplementary Figure 18. ^{1}H NMR (**SA4**) ($CDCl_3$, 500 MHz), ^{13}C NMR (**SA4**) ($CDCl_3$, 75 MHz), and ^{19}F NMR (**SA4**) ($CDCl_3$, 376 MHz)

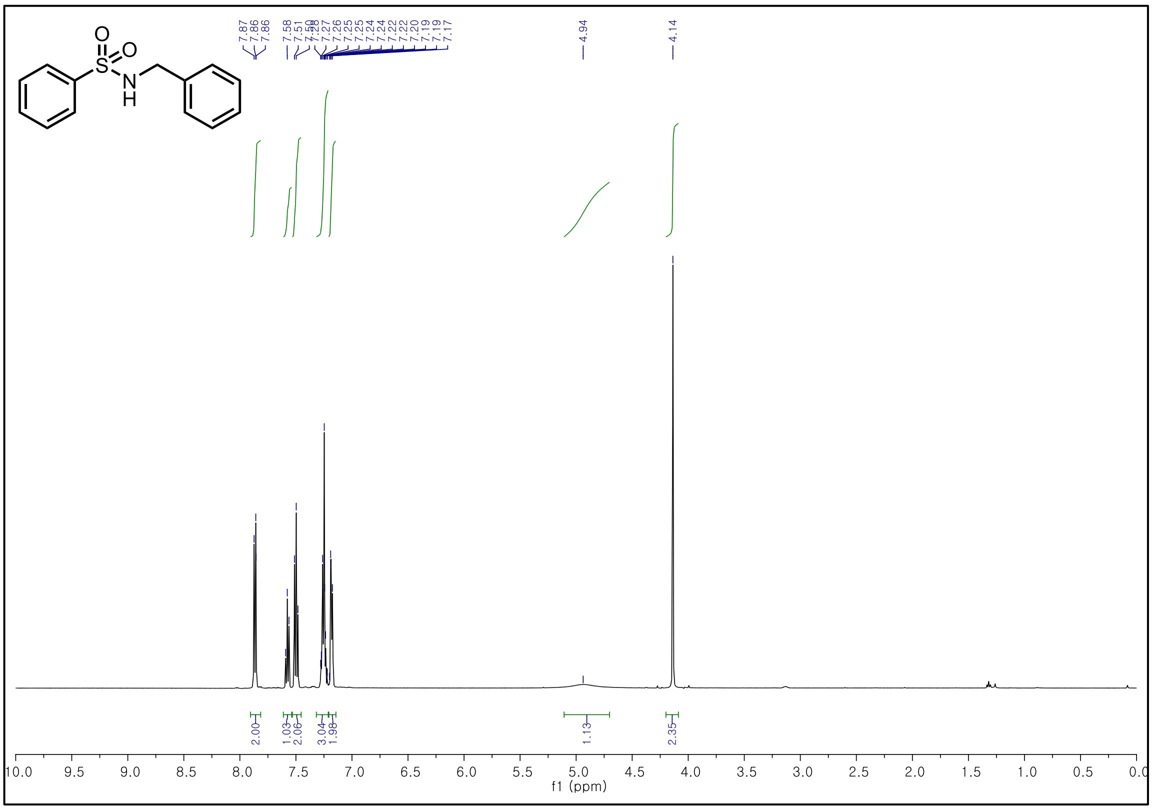

Supplementary Figure 19. ^{1}H NMR (**SA5**) ($CDCl_3$, 500 MHz)

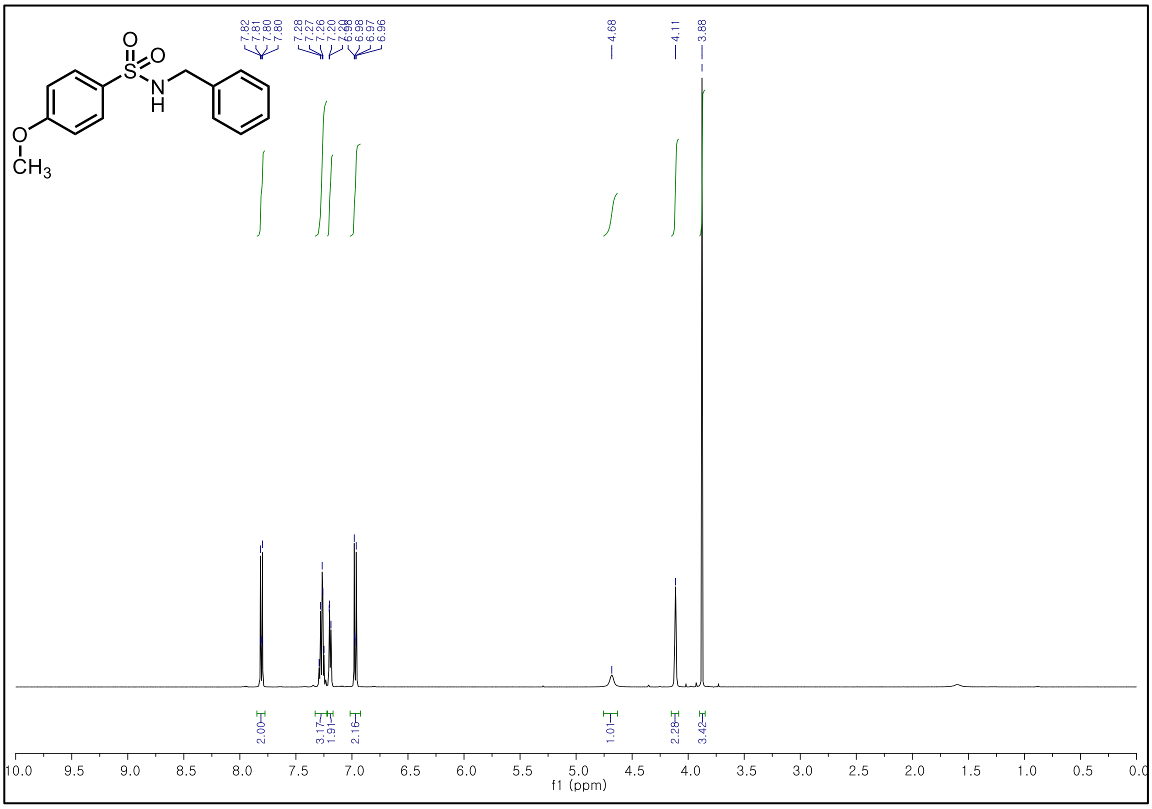

Supplementary Figure 20. ^{1}H NMR (**SA6**) ($CDCl_3$, 500 MHz)

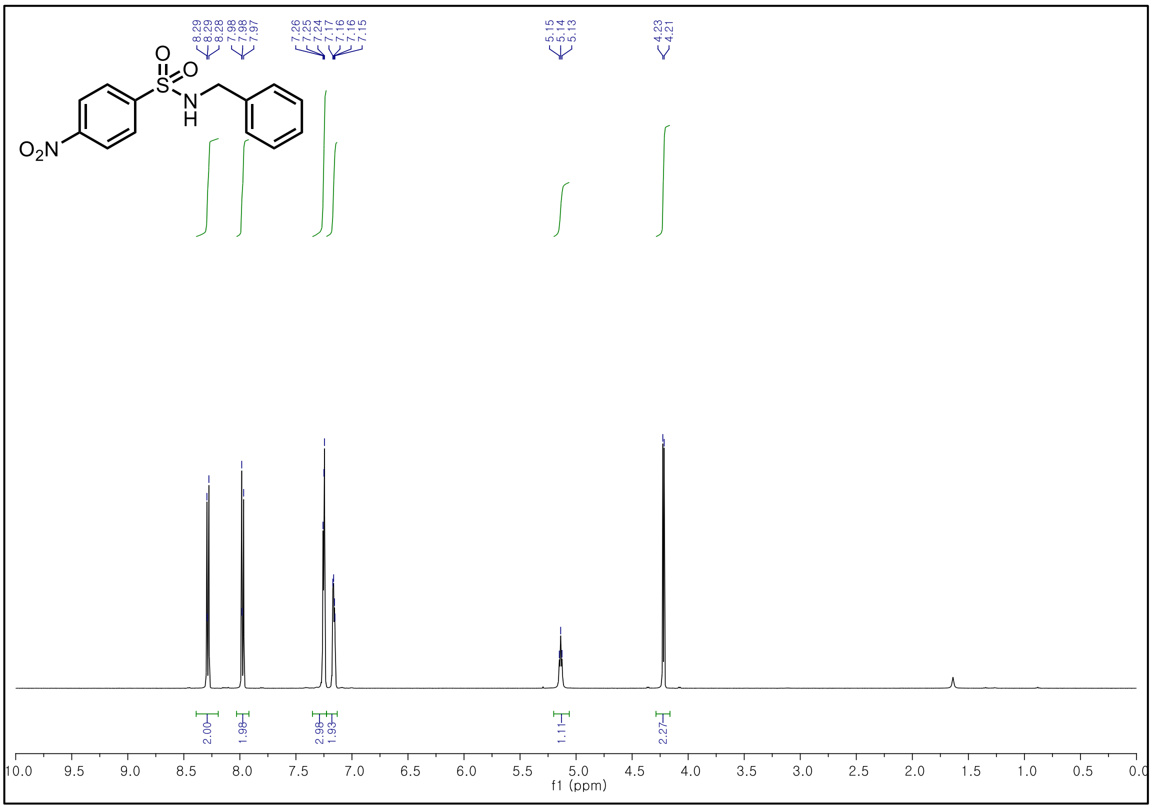

Supplementary Figure 21. ^{1}H NMR (**SA7**) ($CDCl_3$, 500 MHz)

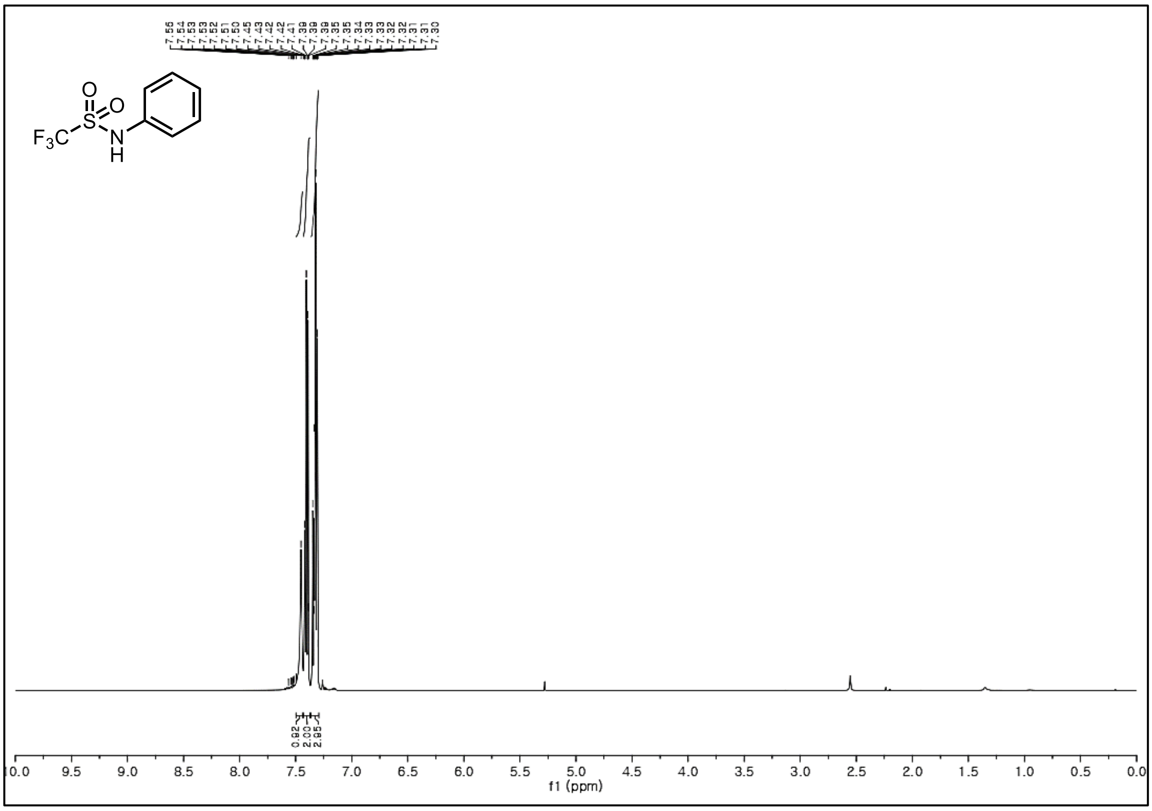

Supplementary Figure 22. ^{1}H NMR (**SA8**) ($CDCl_3$, 500 MHz)

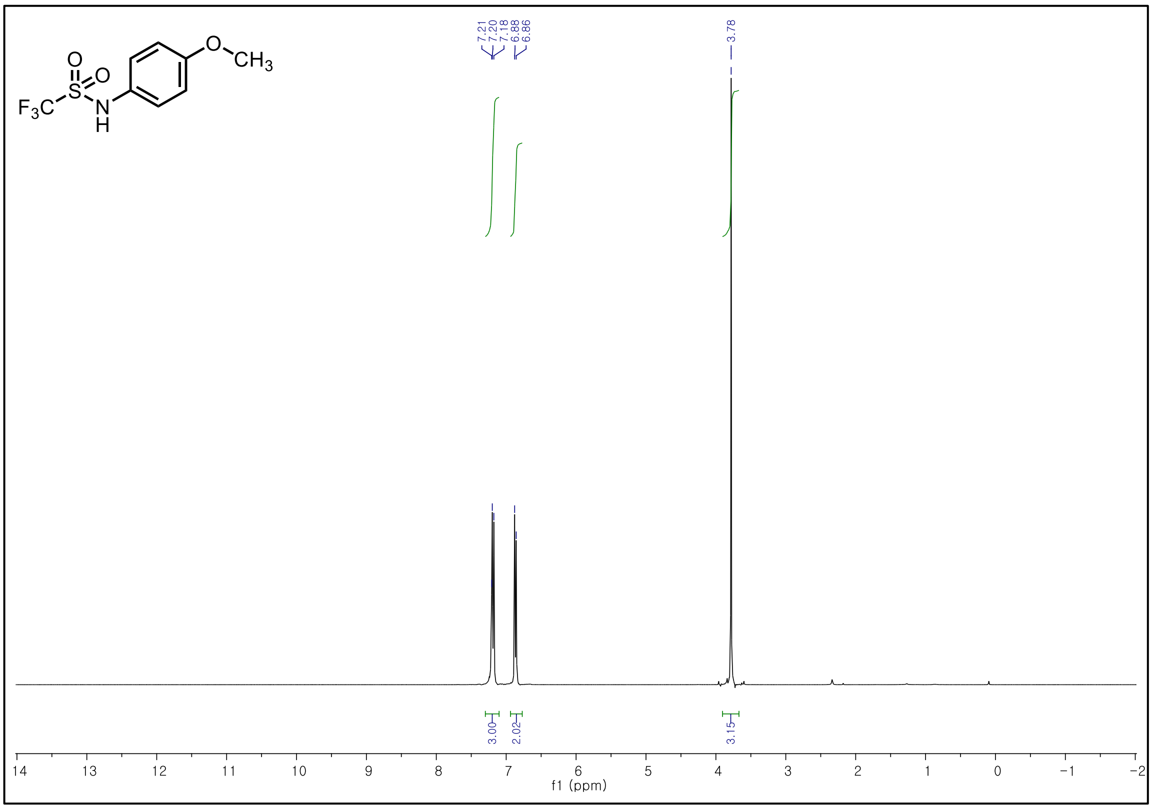

Supplementary Figure 23. ^{1}H NMR (**SA9**) ($CDCl_3$, 400 MHz)

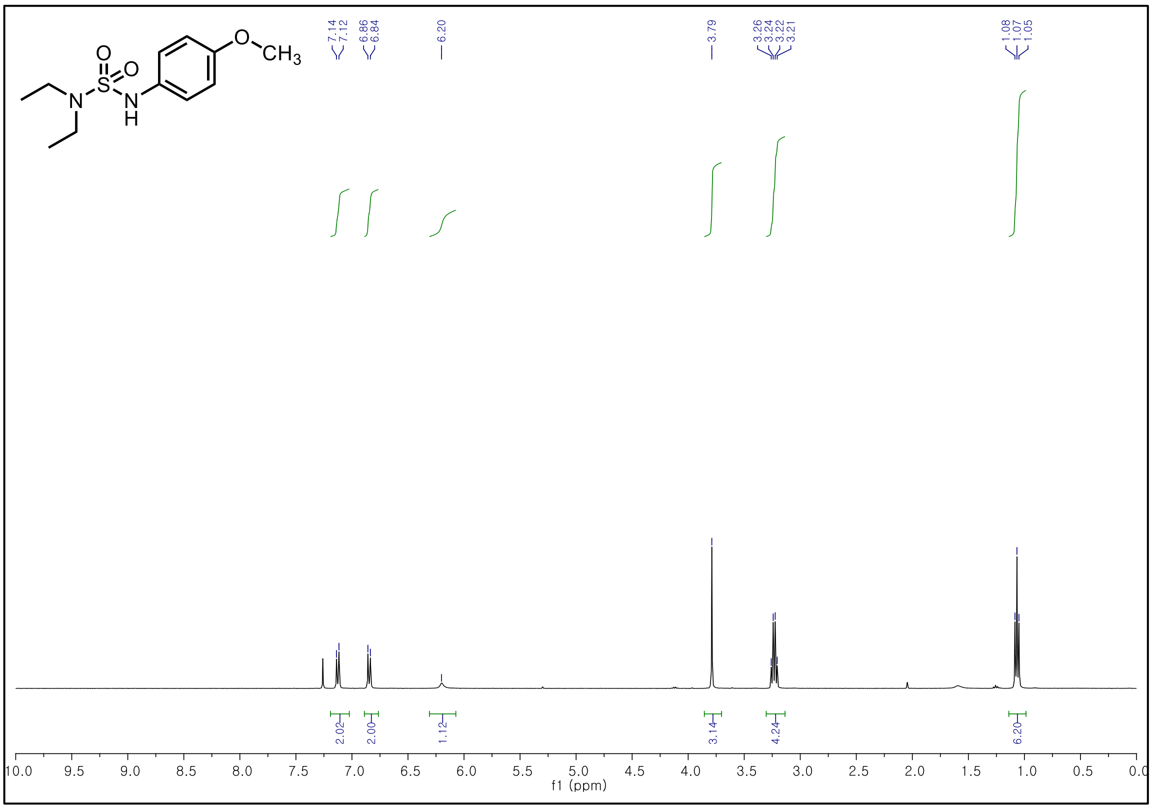

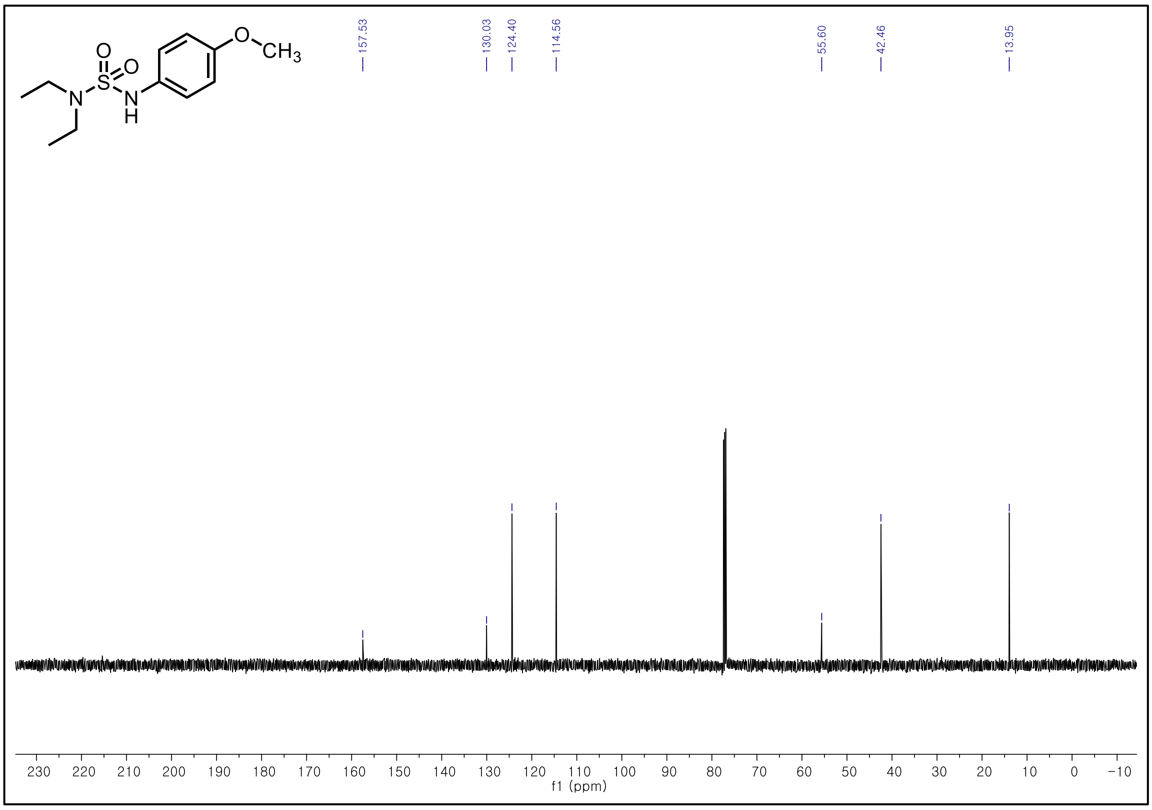

Supplementary Figure 24. ^{1}H NMR (**SA10**) ($CDCl_3$, 300 MHz) and ^{13}C NMR (**SA10**) ($CDCl_3$, 125 MHz)

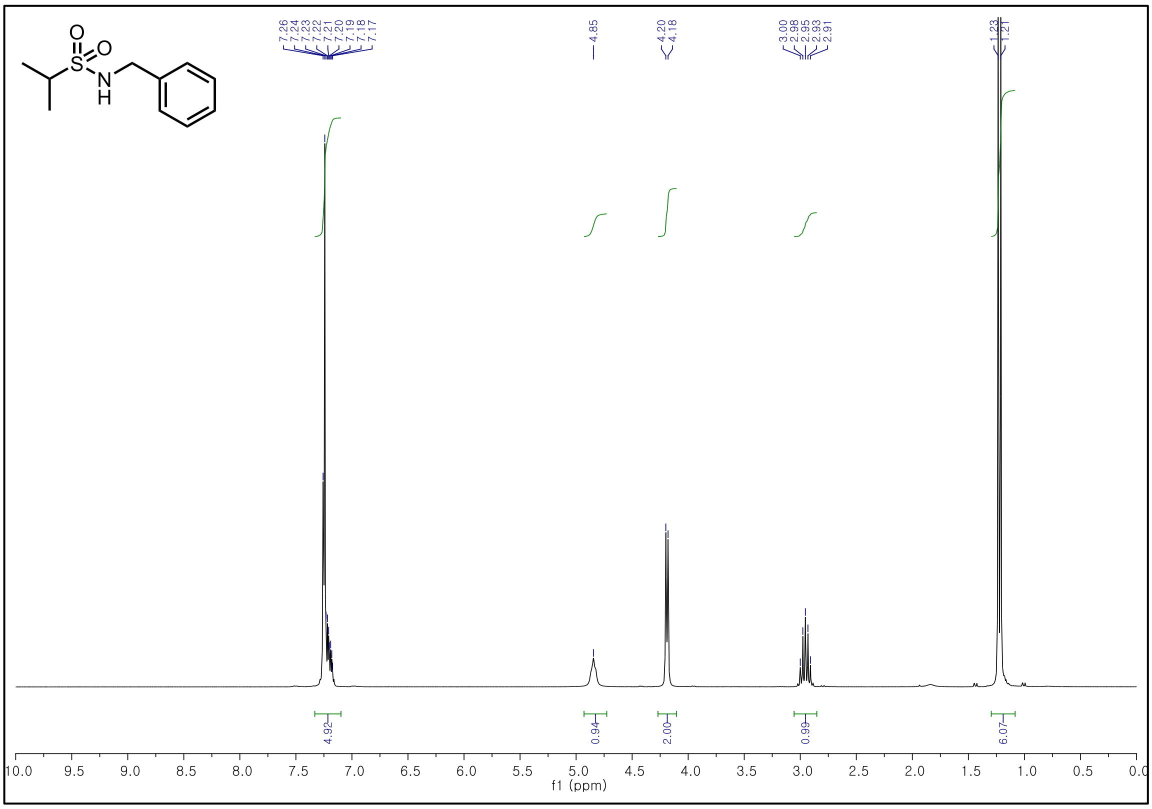

Supplementary Figure 25. ^{1}H NMR (**SA11**) ($CDCl_3$, 300 MHz)

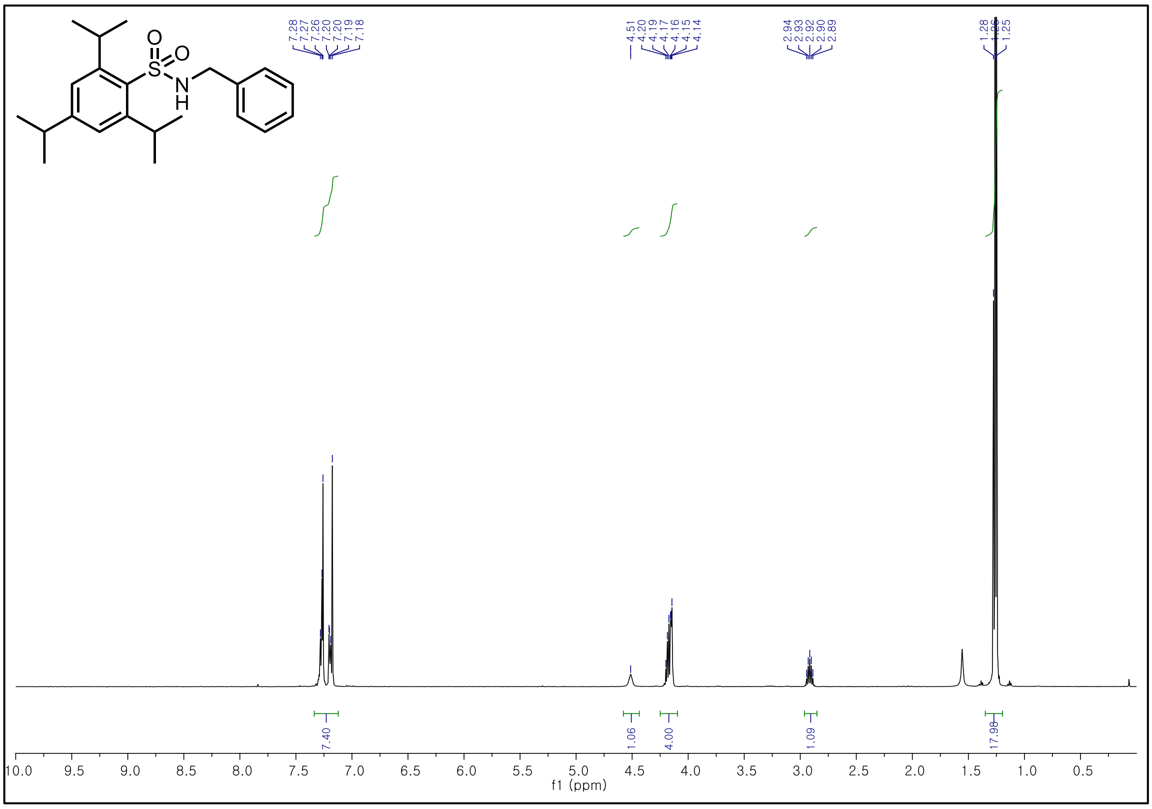

Supplementary Figure 26. ^{1}H NMR (**SA12**) ($CDCl_3$, 500 MHz)

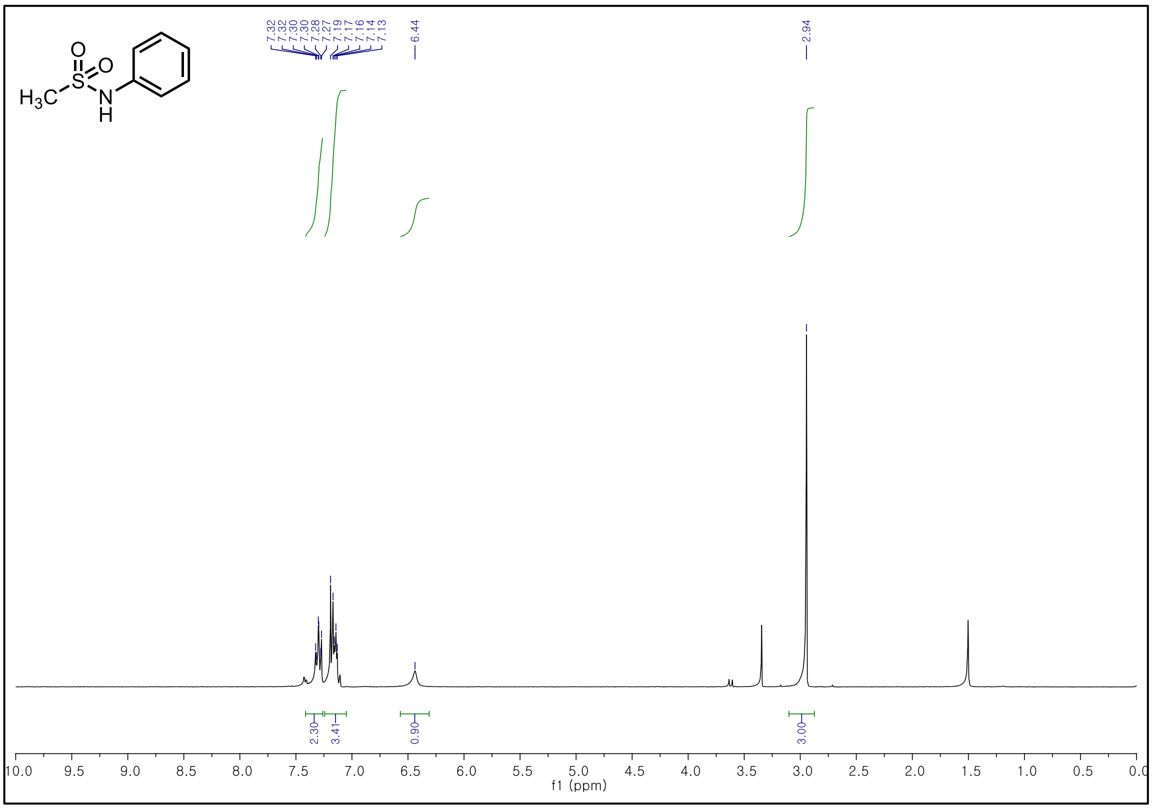

Supplementary Figure 27. ^{1}H NMR (**SA13**) ($CDCl_3$, 300 MHz)

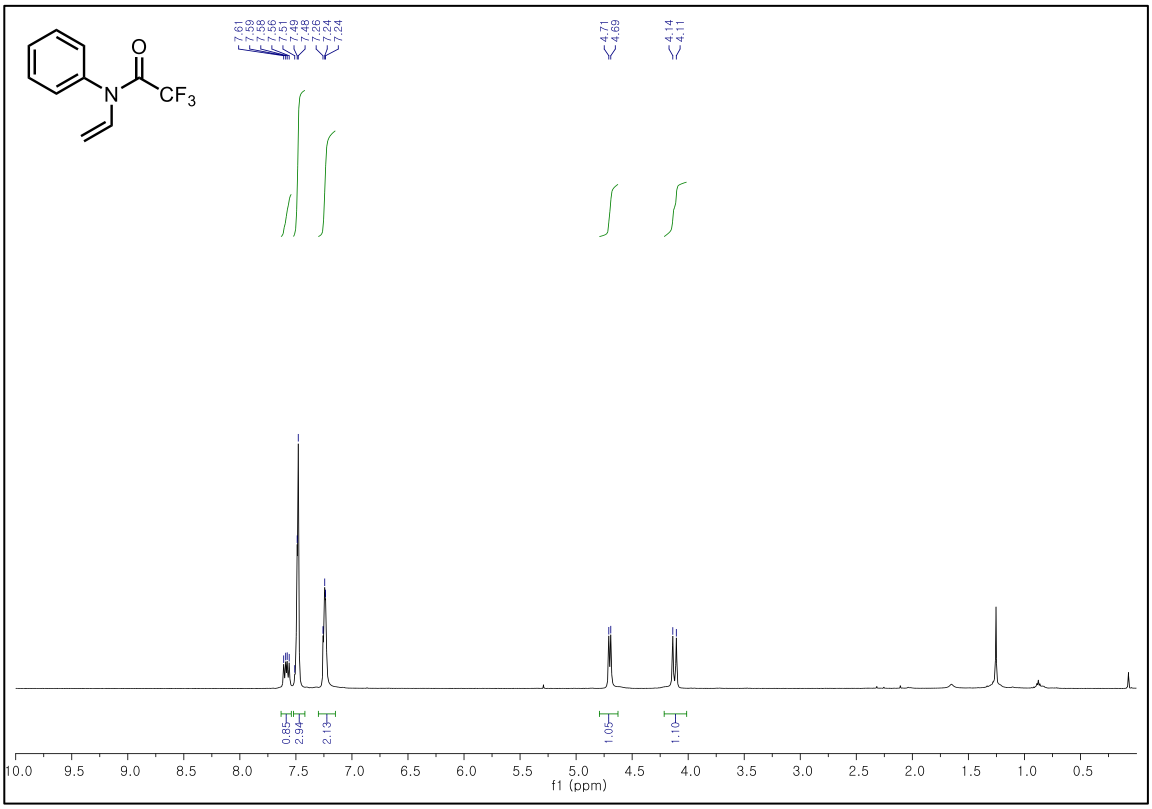
O
N
CF3
7.61
7.59
7.58
7.56
7.51
7.49
7.48
7.26
7.24
7.24
4.71
4.69
4.14
4.11
0.85
2.94
2.13
1.05
1.10
10.0 9.5 9.0 8.5 8.0 7.5 7.0 6.5 6.0 5.5 5.0 4.5 4.0 3.5 3.0 2.5 2.0 1.5 1.0 0.5
f1 (ppm)

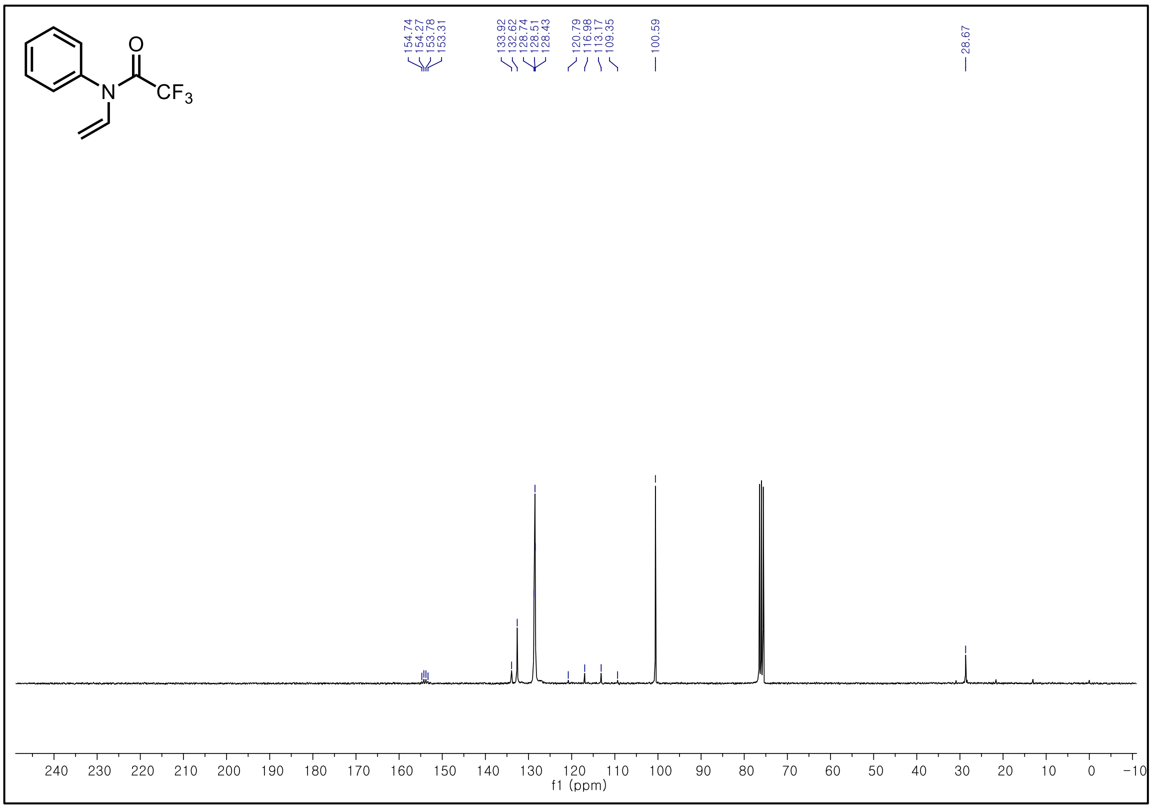
O
N
CF3
154.74
154.27
153.78
153.31
133.92
132.62
128.74
128.51
128.43
120.79
116.98
113.17
109.35
100.59
28.67
240 230 220 210 200 190 180 170 160 150 140 130 120 110 100 90 80 70 60 50 40 30 20 10 0 −10
f1 (ppm)

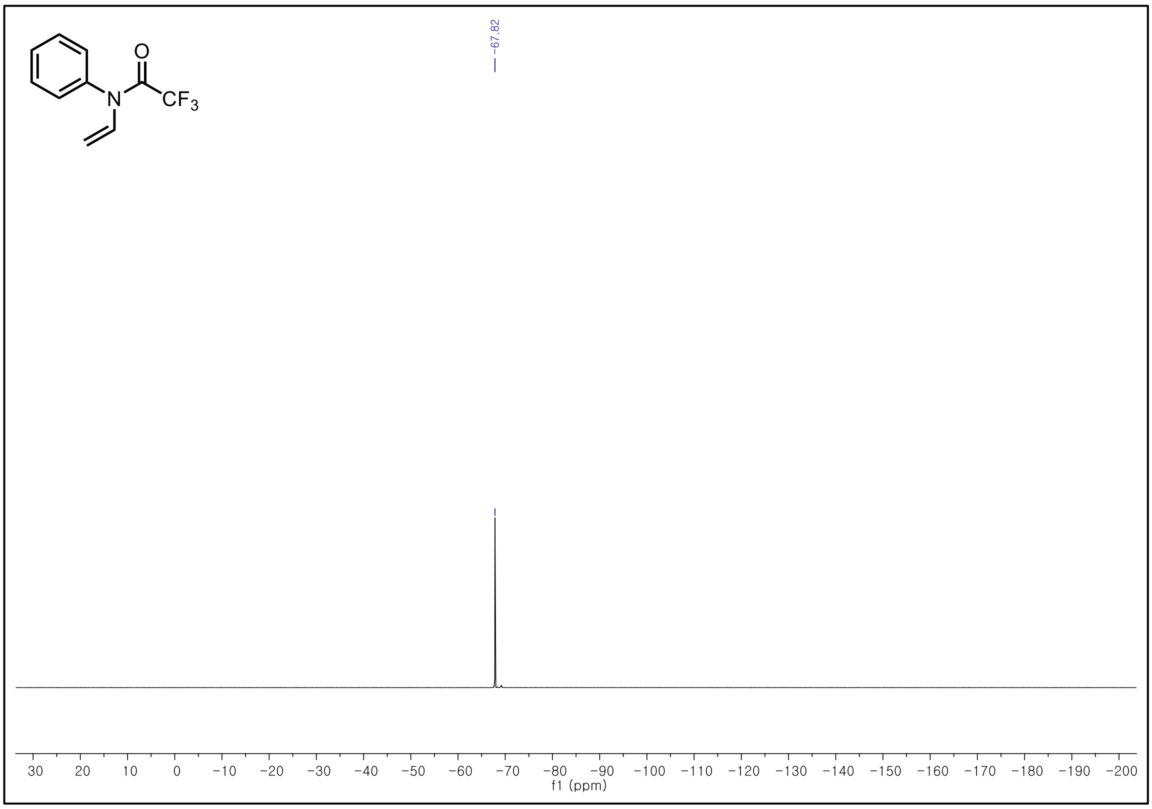

Supplementary Figure 28. ^{1}H NMR (**VA1**) ($CDCl_3$, 300 MHz), ^{13}C NMR (**VA1**) ($CDCl_3$, 75 MHz), and ^{19}F NMR (**VA1**) ($CDCl_3$, 376 MHz)

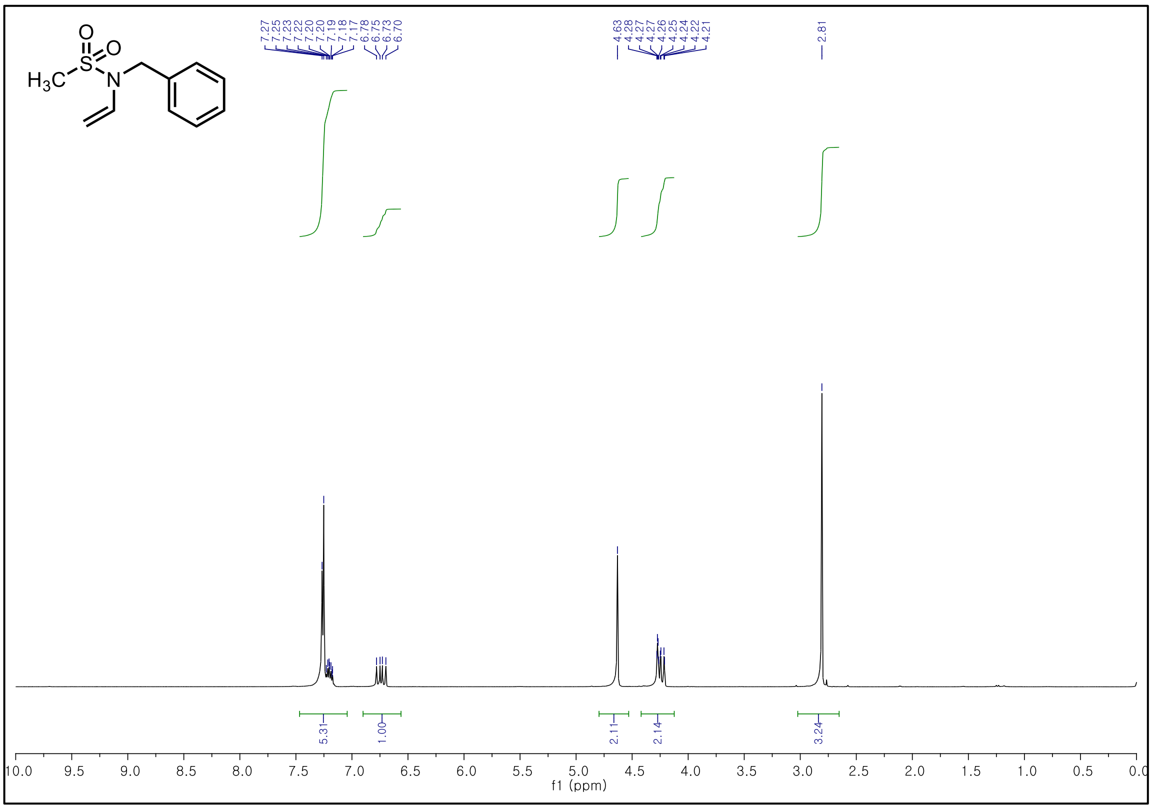

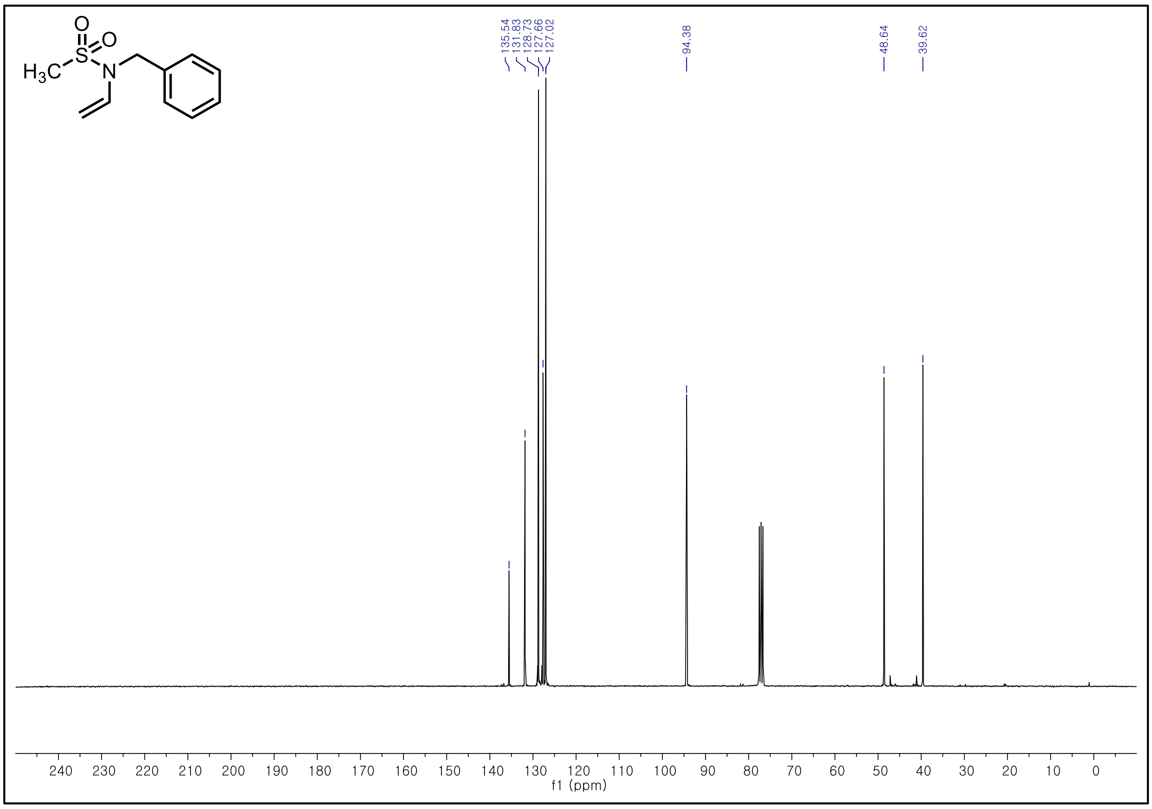

Supplementary Figure 29. ^{1}H NMR (**VS1**) ($CDCl_3$, 300 MHz), and ^{13}C NMR (**VS1**) ($CDCl_3$, 75 MHz)

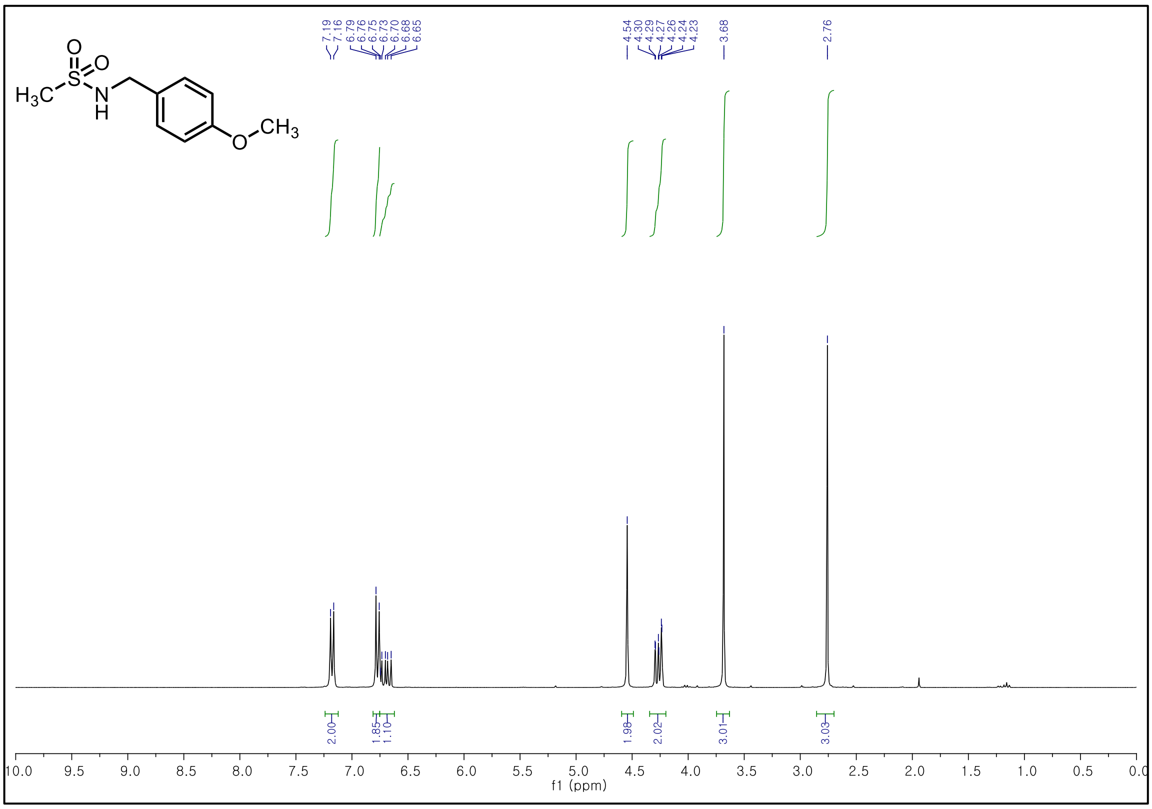

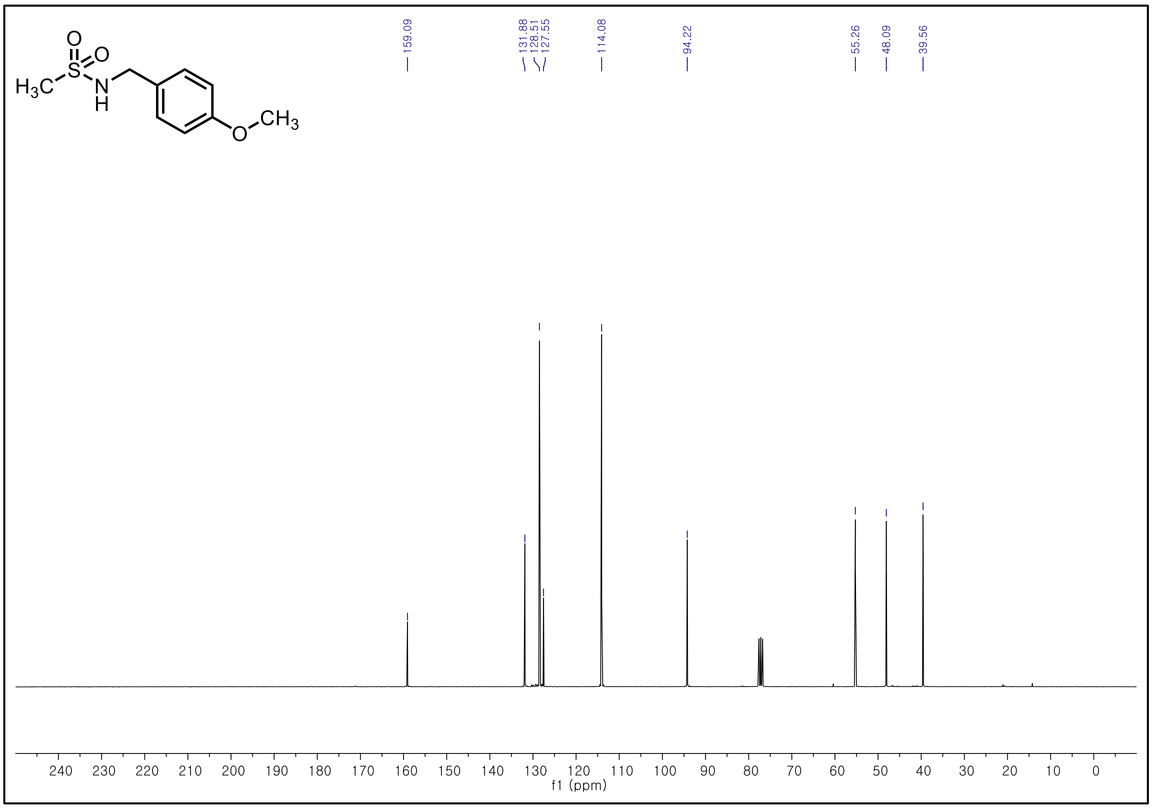

Supplementary Figure 30. ^{1}H NMR (**VS2**) ($CDCl_3$, 300 MHz), and ^{13}C NMR (**VS2**) ($CDCl_3$, 75 MHz)

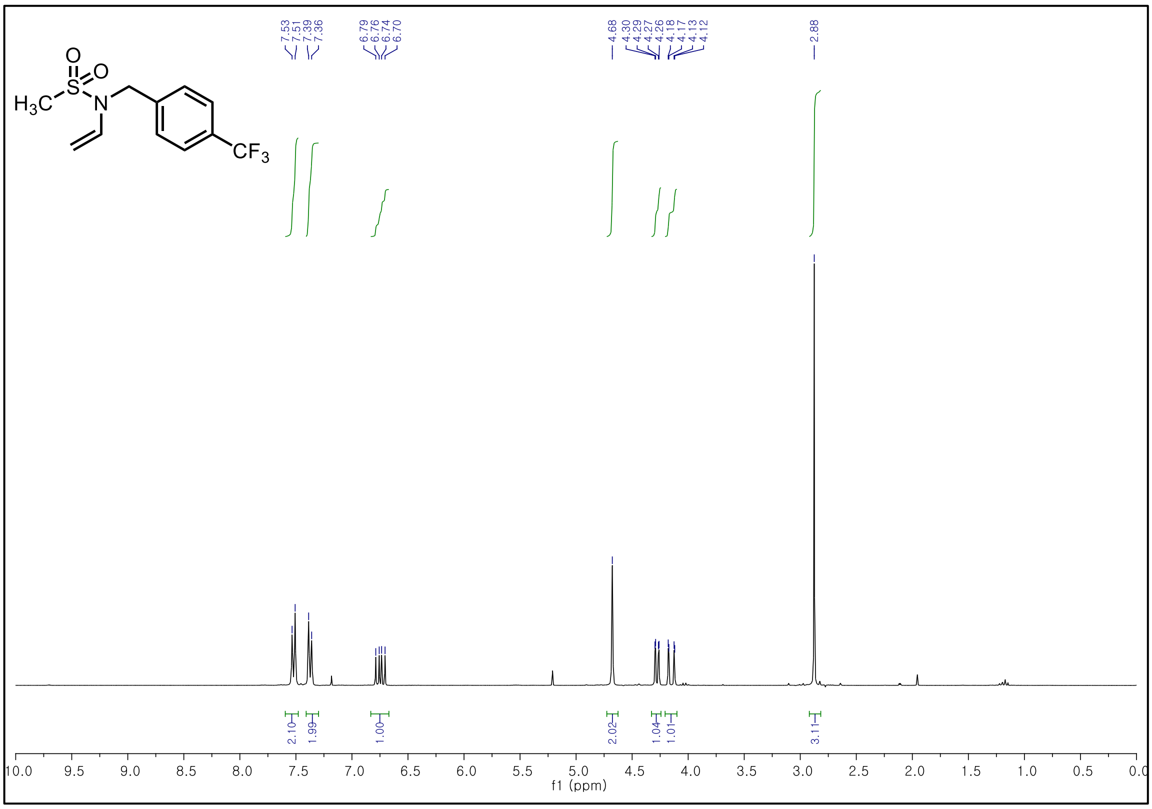
7.53
7.51
7.39
7.36
6.79
6.76
6.74
6.70
4.68
4.30
4.29
4.27
4.26
4.18
4.17
4.13
4.12
2.88
H3C
S
O
O
N
CF3
2.10
1.99
1.00
2.02
1.04
1.01
3.11
f1 (ppm)

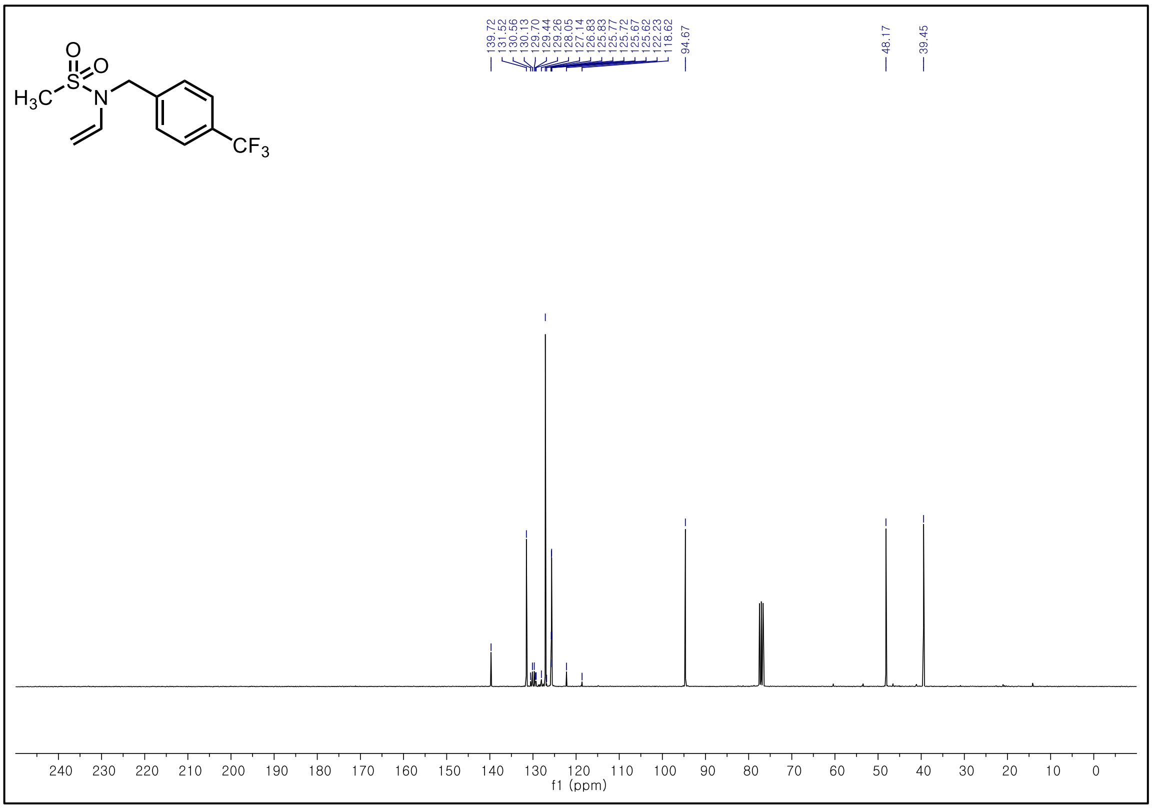
139.72
131.52
130.56
130.13
129.70
129.44
129.26
128.05
127.14
126.83
125.83
125.77
125.72
125.67
125.62
122.23
118.62
94.67
48.17
39.45
H3C
S
O
O
N
CF3
f1 (ppm)

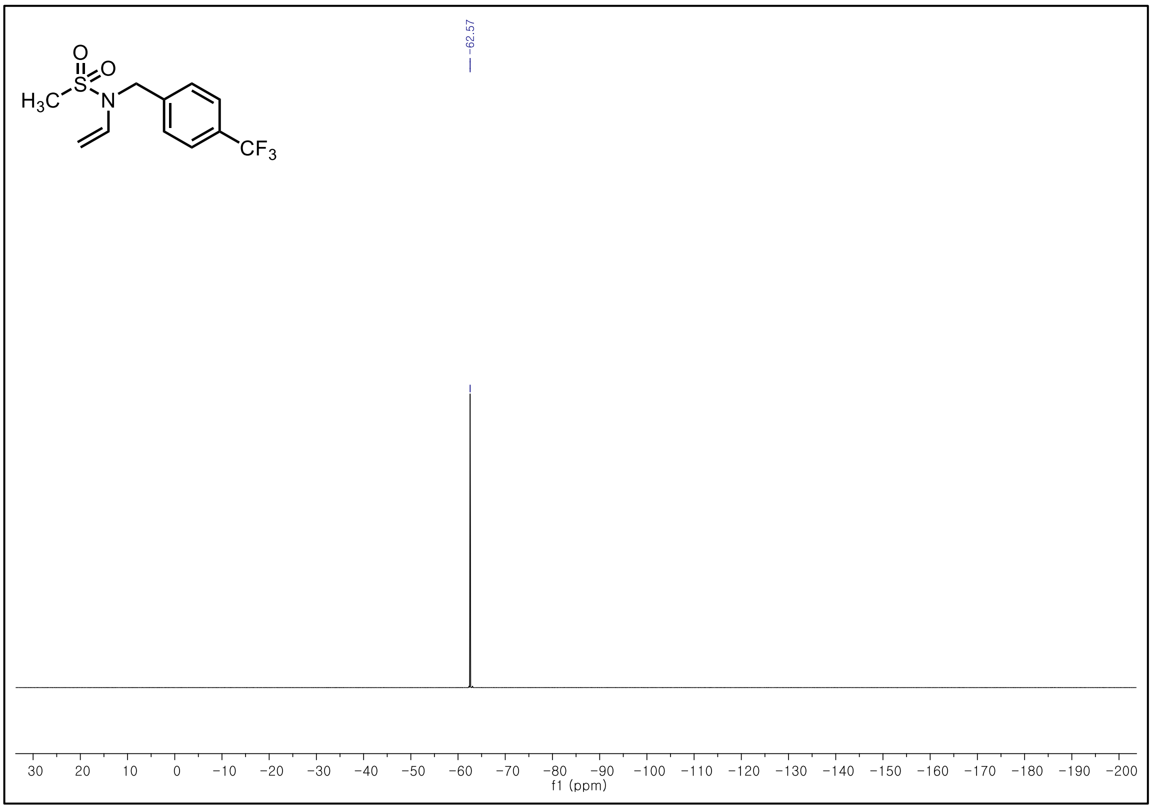

Supplementary Figure 31. ^{1}H NMR (**VS3**) ($CDCl_3$, 300 MHz), ^{13}C NMR (**VS3**) ($CDCl_3$, 75 MHz), and ^{19}F NMR (**VS3**) ($CDCl_3$, 376 MHz)

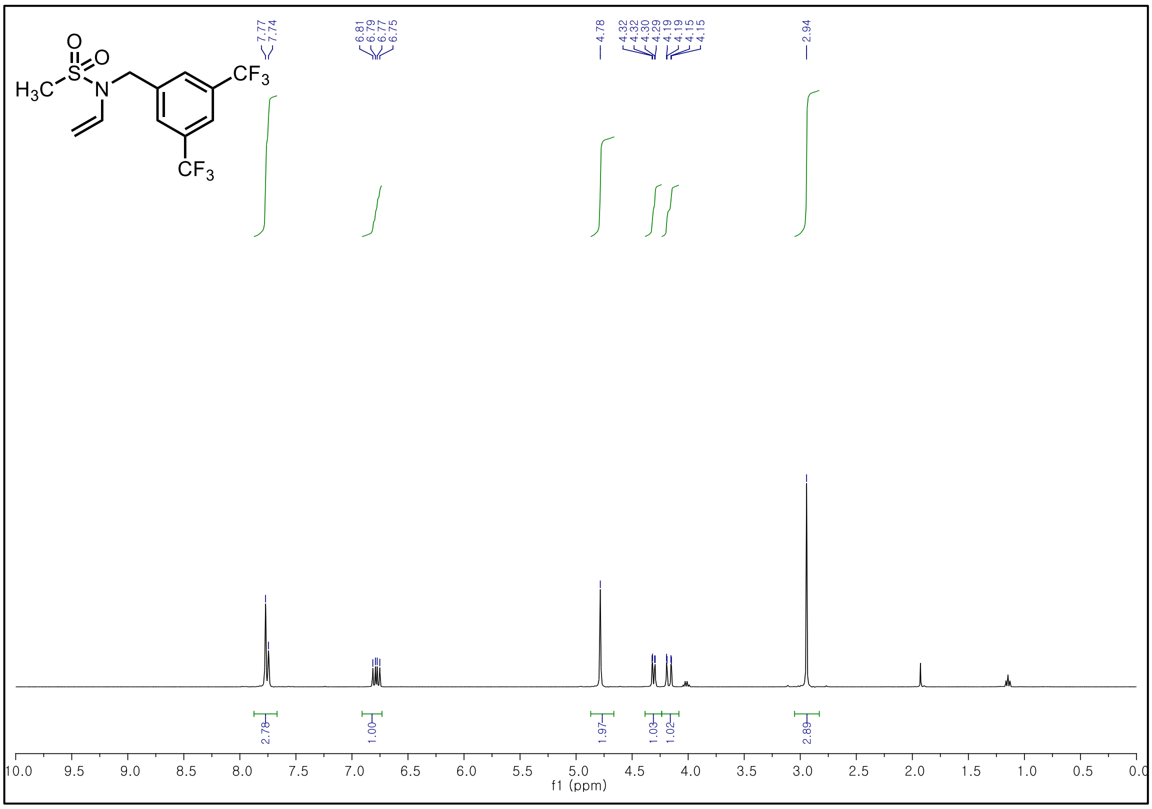
7.77
7.74
6.81
6.79
6.77
6.75
4.78
4.32
4.32
4.30
4.29
4.19
4.19
4.15
4.15
2.94
2.78
1.00
1.97
1.03
1.02
2.89
f1 (ppm)

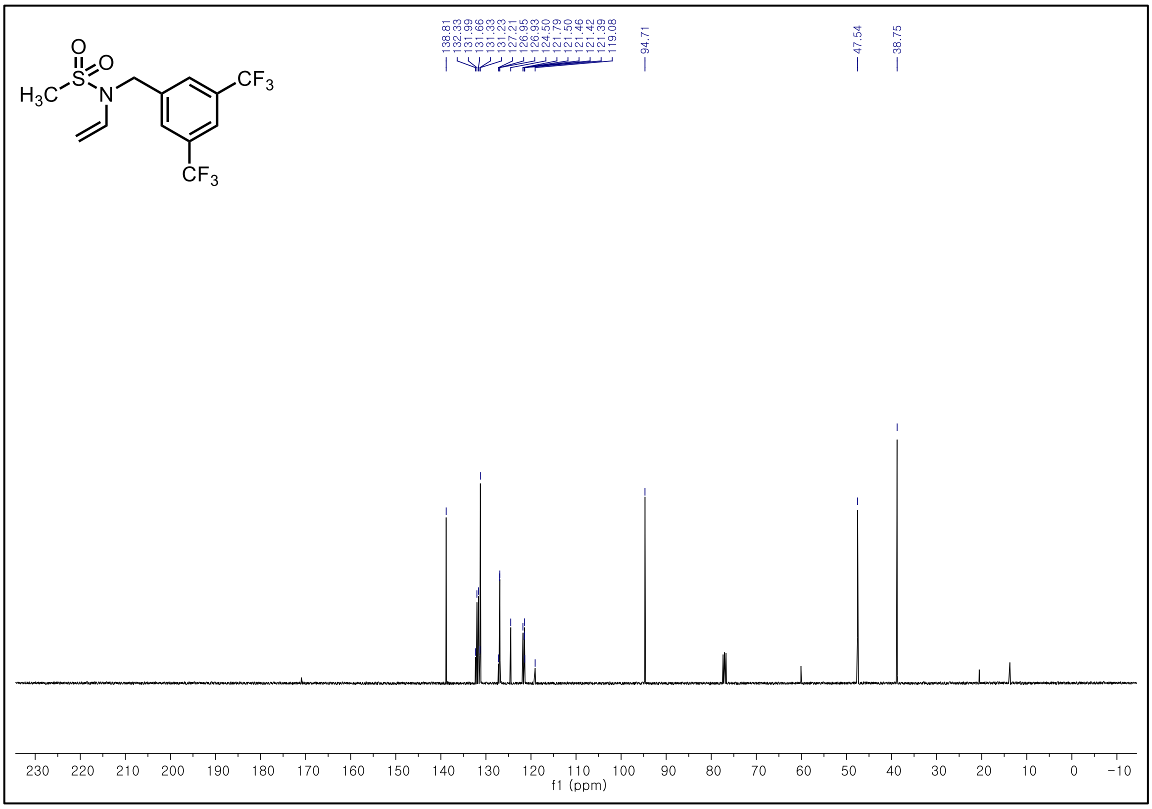
138.81
132.33
131.99
131.66
131.33
131.23
127.21
126.95
126.93
124.50
121.79
121.50
121.46
121.42
121.39
119.08
94.71
47.54
38.75
f1 (ppm)

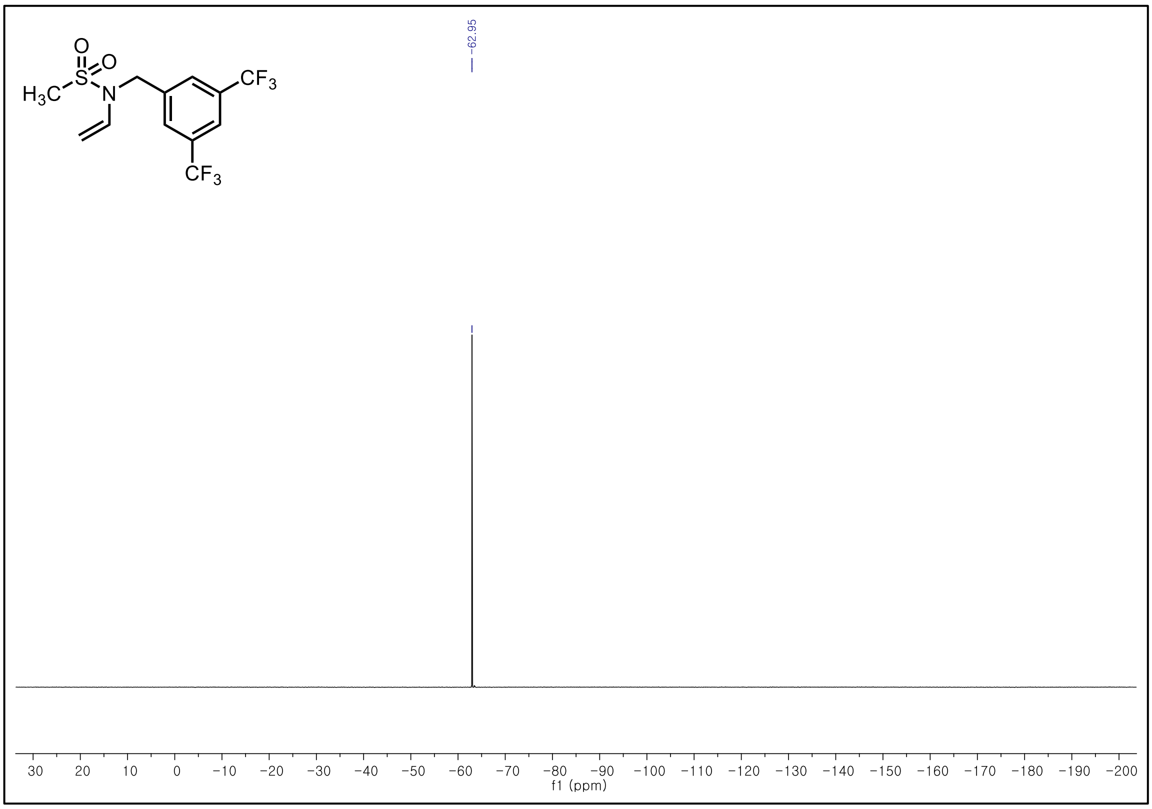

Supplementary Figure 32. ^{1}H NMR (**VS4**) ($CDCl_3$, 400 MHz), ^{13}C NMR (**VS4**) ($CDCl_3$, 100 MHz), and ^{19}F NMR (**VS4**) ($CDCl_3$, 376 MHz)

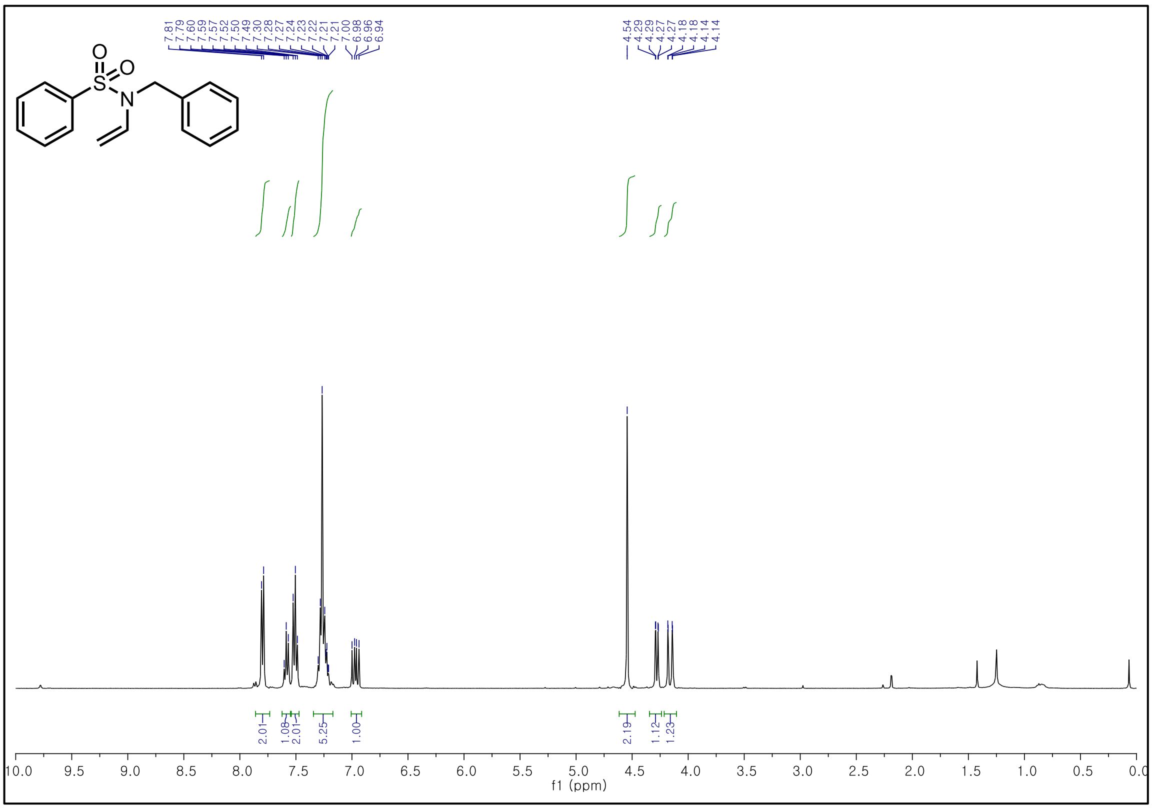

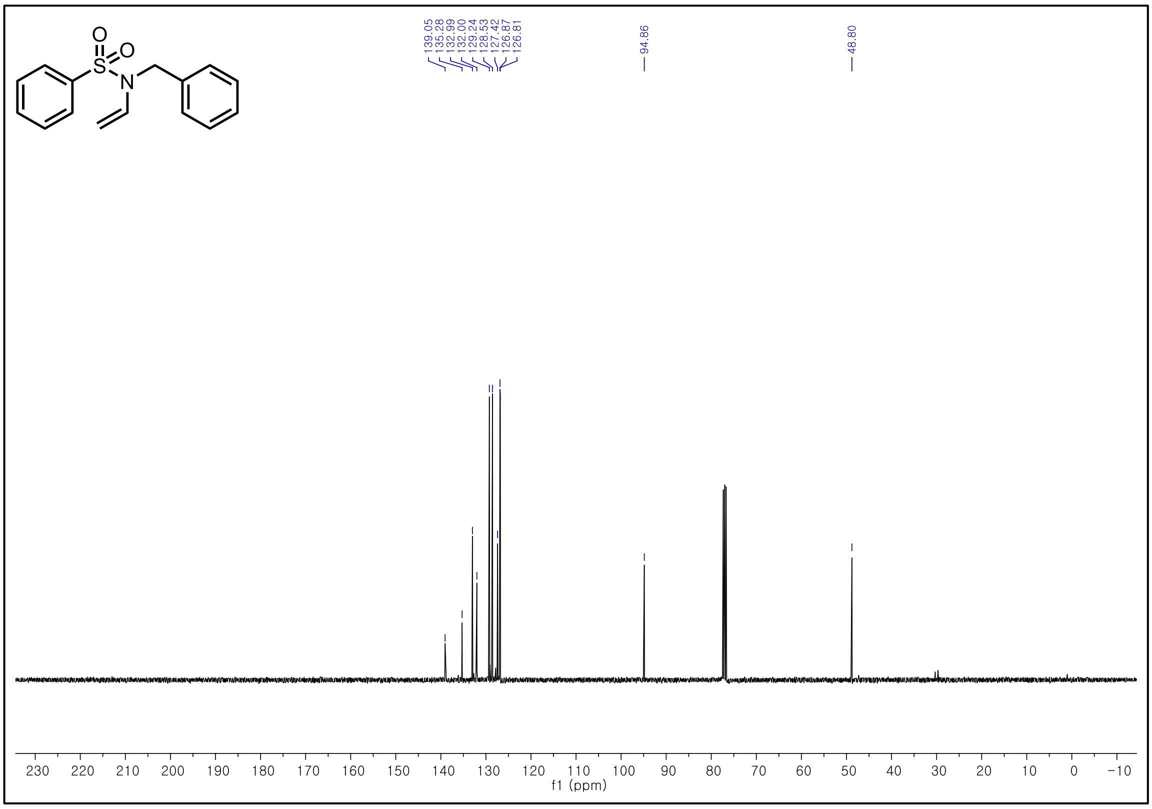

Supplementary Figure 33. ^{1}H NMR (**VS5**) ($CDCl_3$, 400 MHz), and ^{13}C NMR (**VS5**) ($CDCl_3$, 100 MHz)

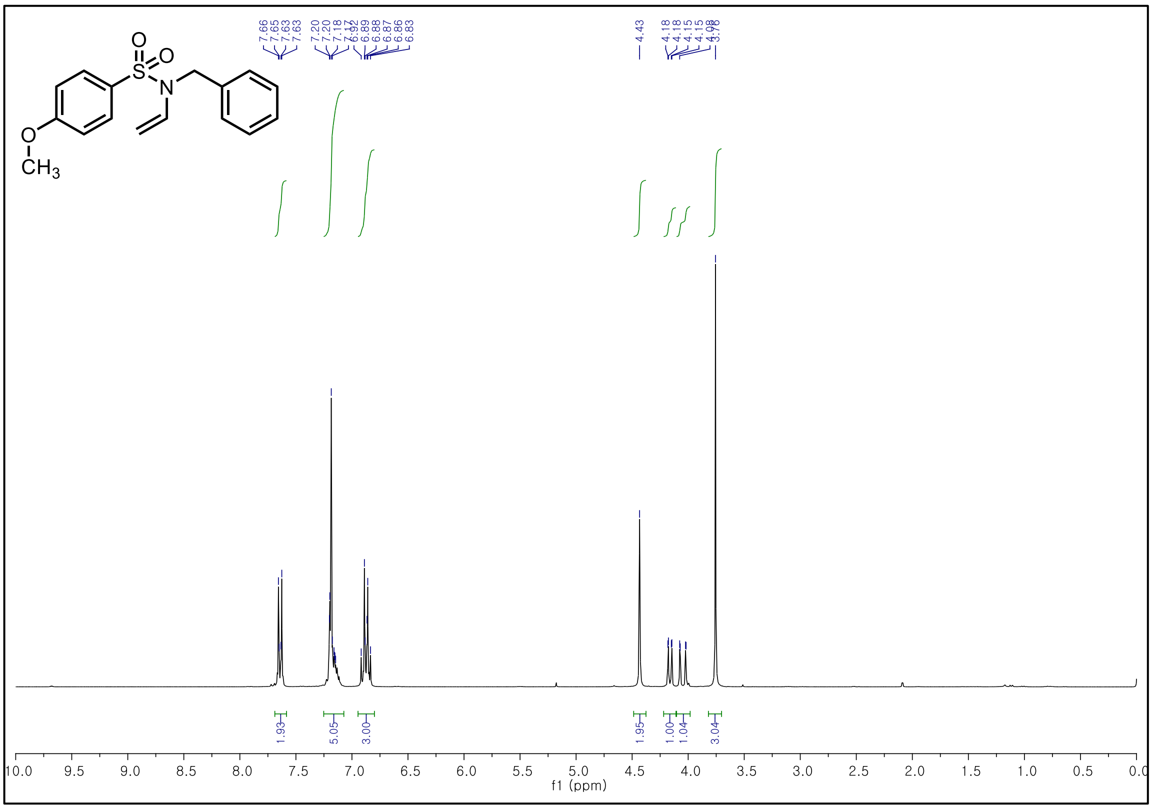

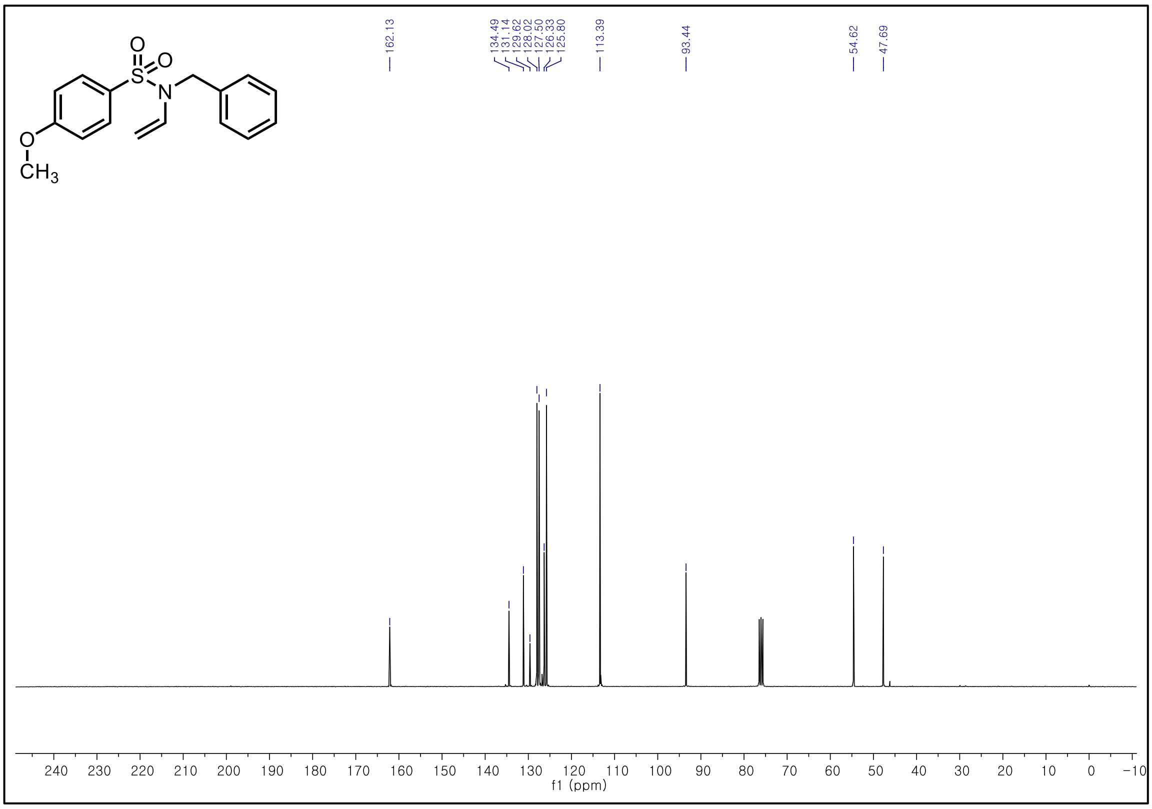

Supplementary Figure 34. ^{1}H NMR (**VS6**) ($CDCl_3$, 300 MHz), and ^{13}C NMR (**VS6**) ($CDCl_3$, 75 MHz)

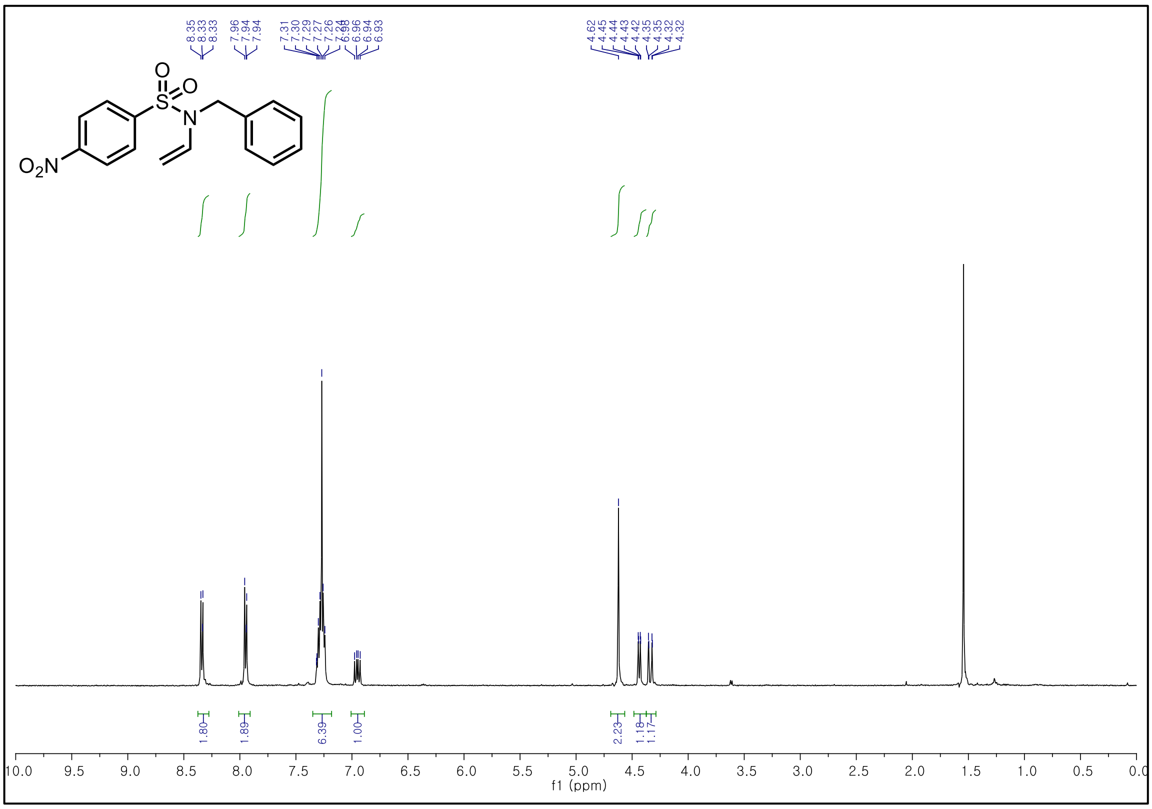

Supplementary Figure 35. ^{1}H NMR (**VS7**) ($CDCl_3$, 500 MHz)

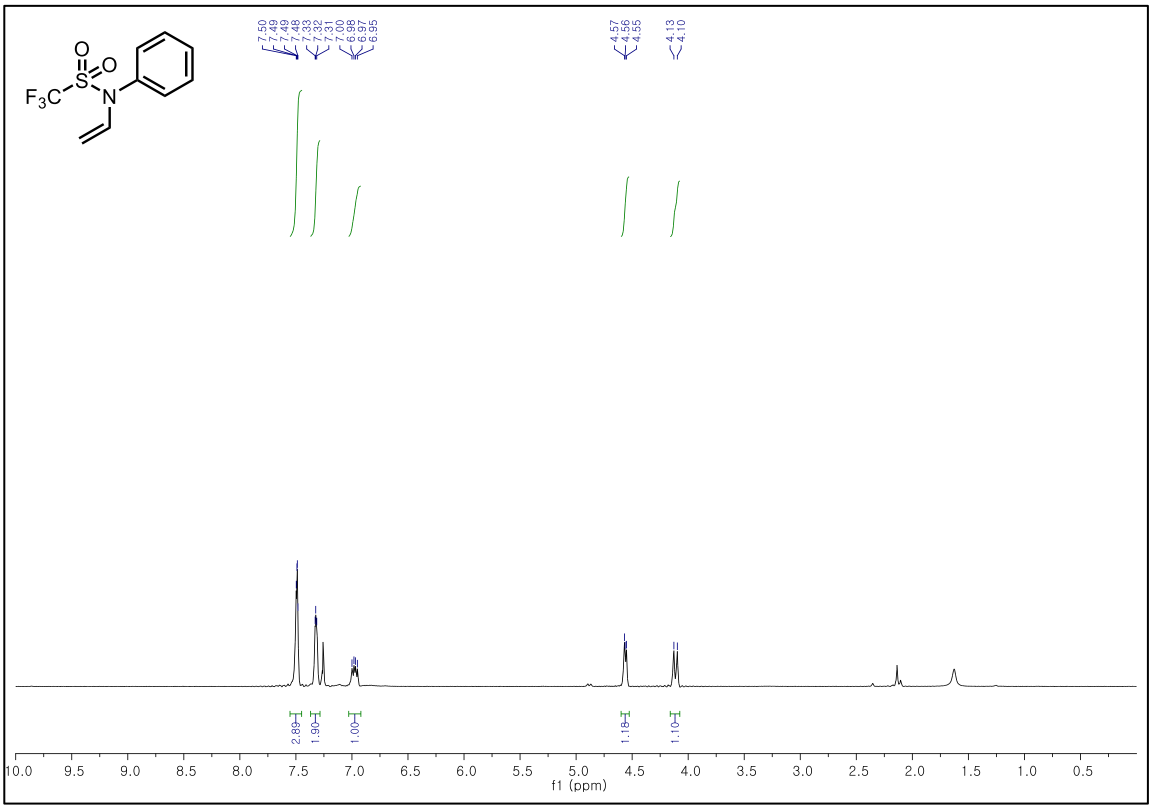
7.50
7.49
7.49
7.48
7.33
7.32
7.31
7.00
6.98
6.97
6.95
4.57
4.56
4.55
4.13
4.10
2.89
1.90
1.00
1.18
1.10
10.0 9.5 9.0 8.5 8.0 7.5 7.0 6.5 6.0 5.5 5.0 4.5 4.0 3.5 3.0 2.5 2.0 1.5 1.0 0.5
f1 (ppm)

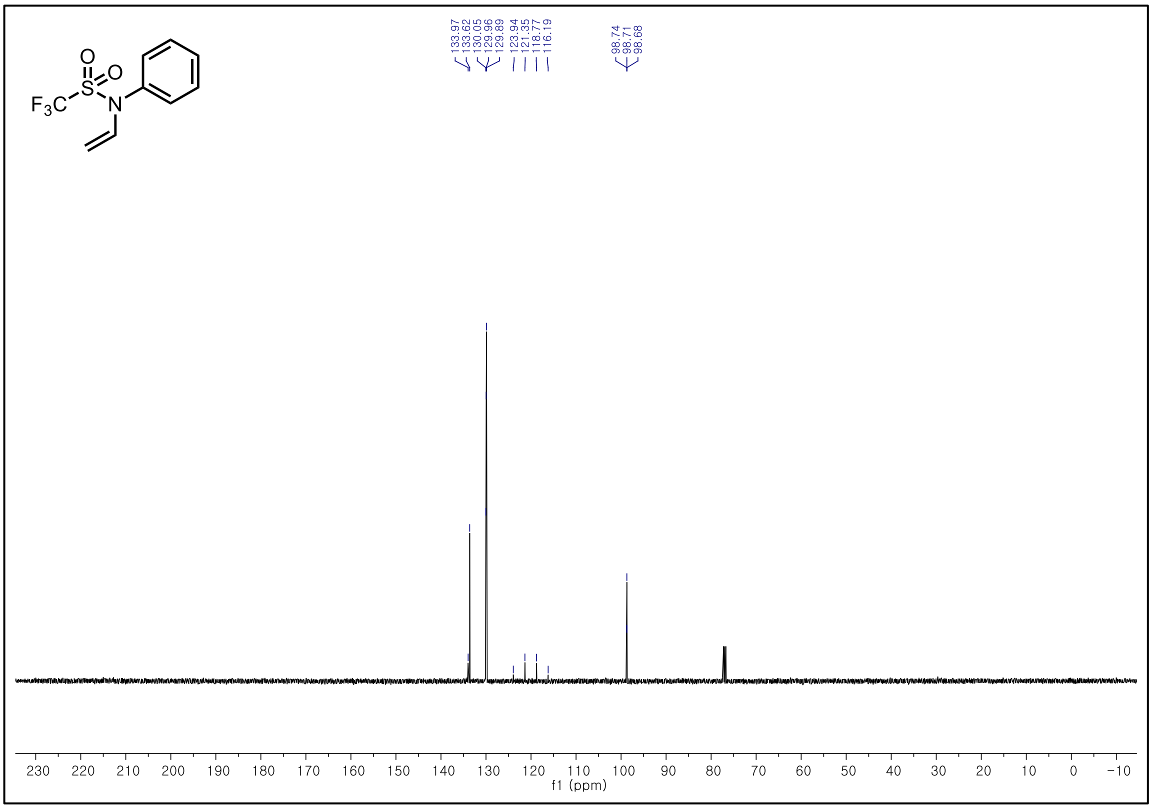
133.97
133.62
130.05
129.96
129.89
123.94
121.35
118.77
116.19
98.74
98.71
98.68
230 220 210 200 190 180 170 160 150 140 130 120 110 100 90 80 70 60 50 40 30 20 10 0 −10
f1 (ppm)

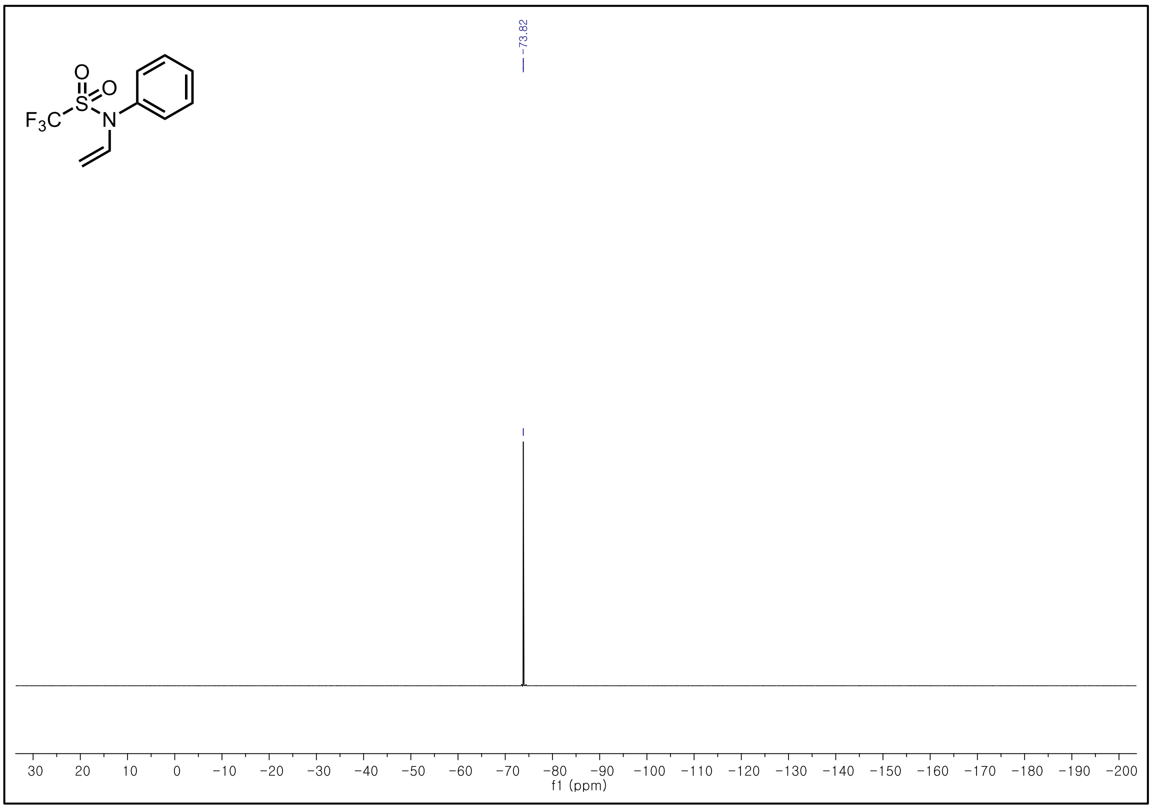

Supplementary Figure 36. ^{1}H NMR (**VS8**) ($CDCl_3$, 500 MHz), ^{13}C NMR (**VS8**) ($CDCl_3$, 125 MHz), and ^{19}F NMR (**VS8**) ($CDCl_3$, 376 MHz)

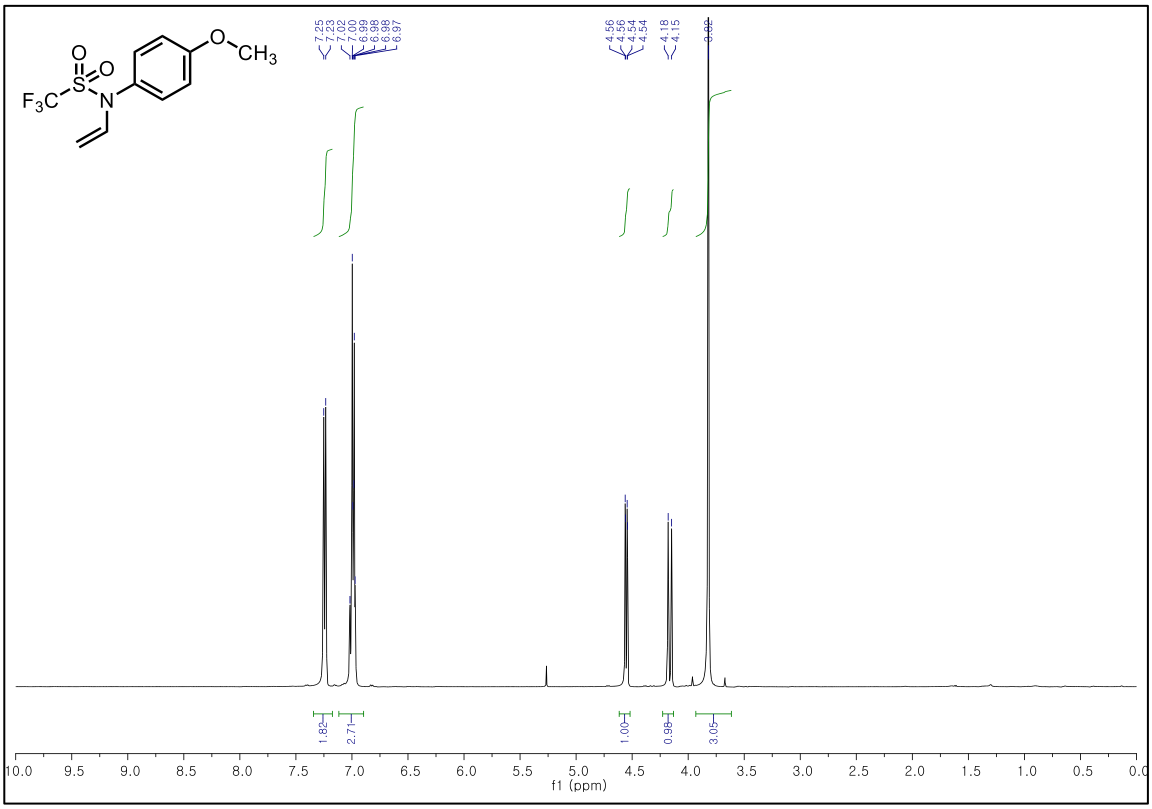

7.25
7.23
7.02
7.00
6.99
6.98
6.98
6.97
4.56
4.56
4.54
4.54
4.18
4.15
3.82
1.82
2.71
1.00
0.98
3.05
f1 (ppm)

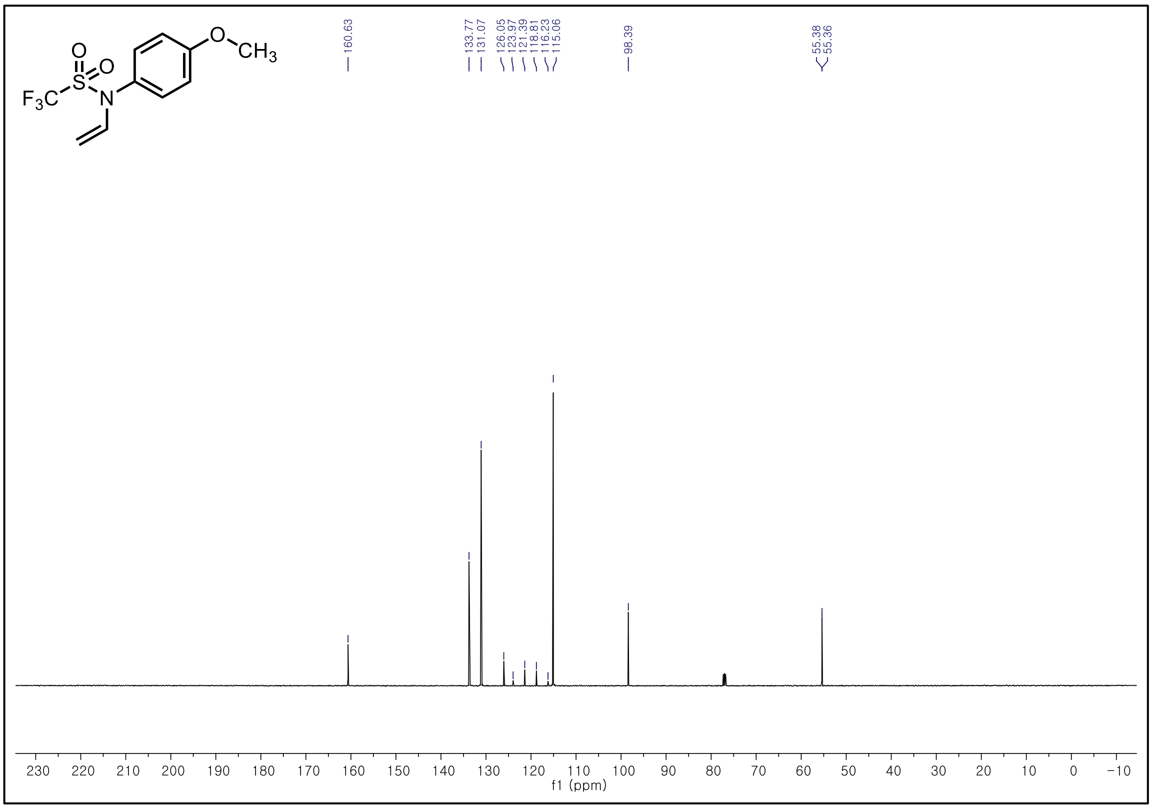

160.63
133.77
131.07
126.05
123.97
121.39
118.81
116.23
115.06
98.39
55.38
55.36
f1 (ppm)

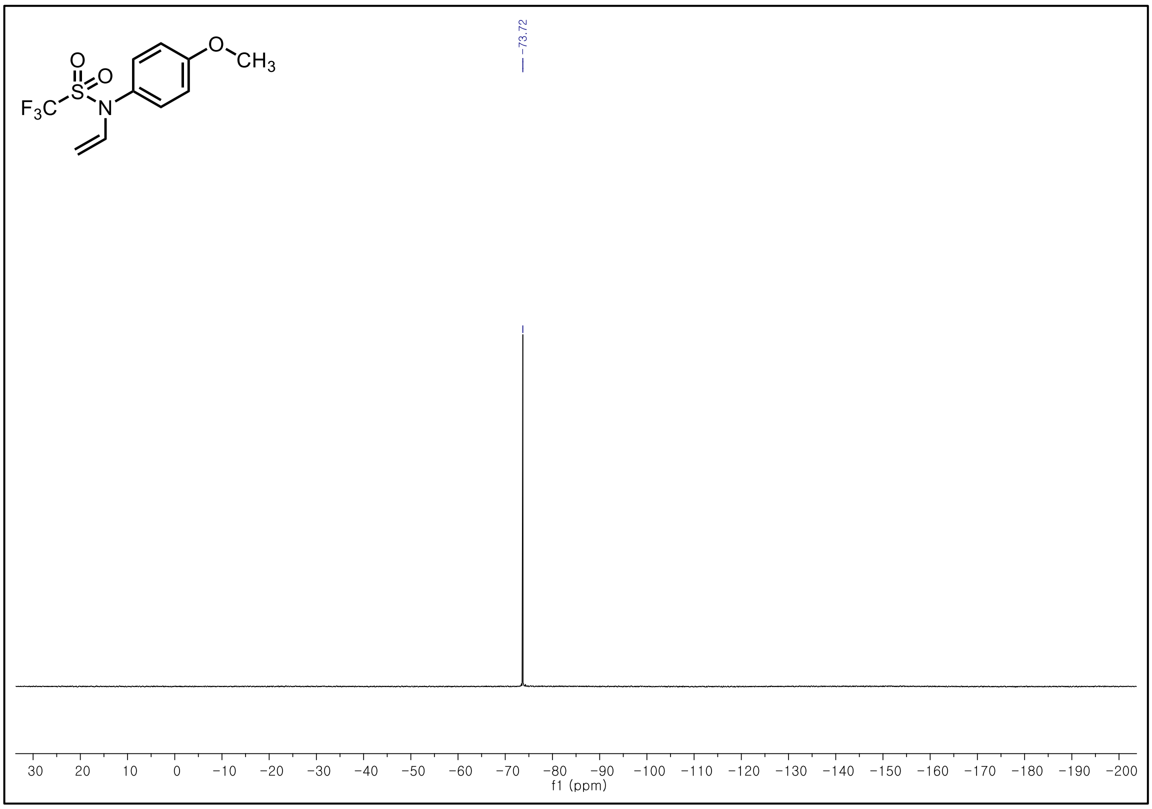

Supplementary Figure 37. ^{1}H NMR (**VS9**) ($CDCl_3$, 500 MHz), ^{13}C NMR (**VS9**) ($CDCl_3$, 125 MHz), and ^{19}F NMR (**VS9**) ($CDCl_3$, 376 MHz)

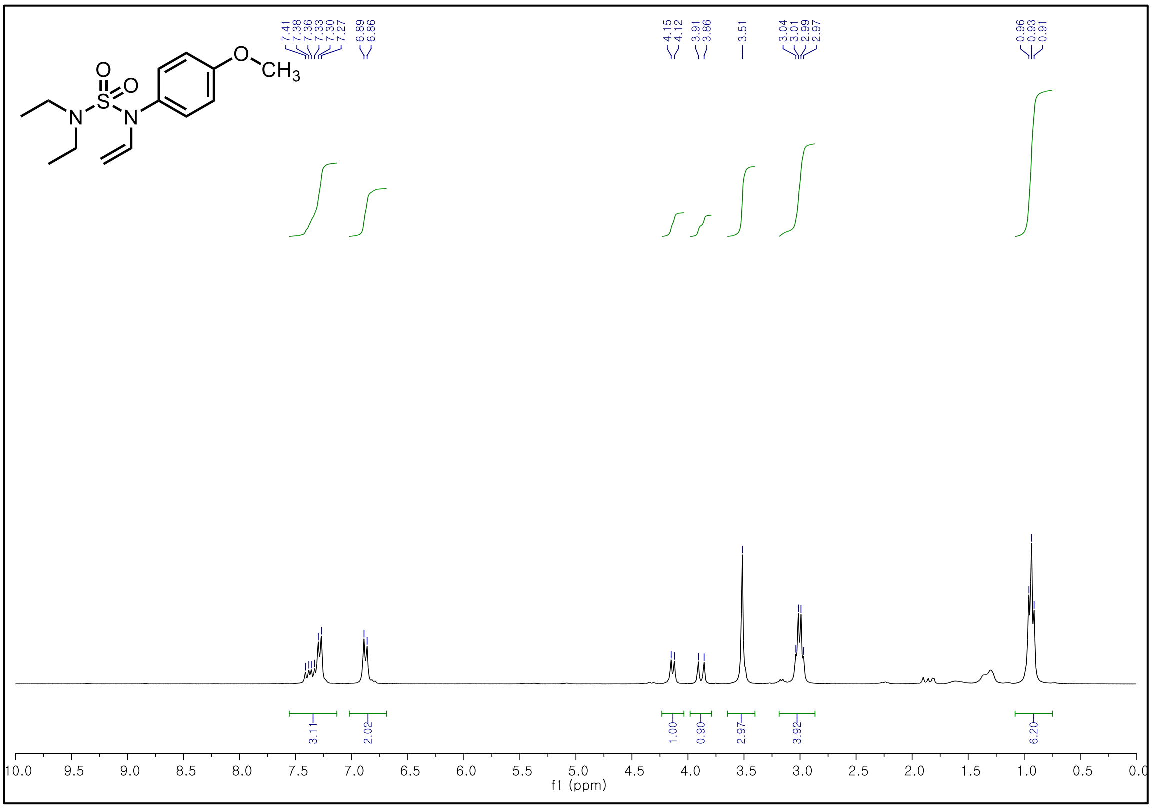

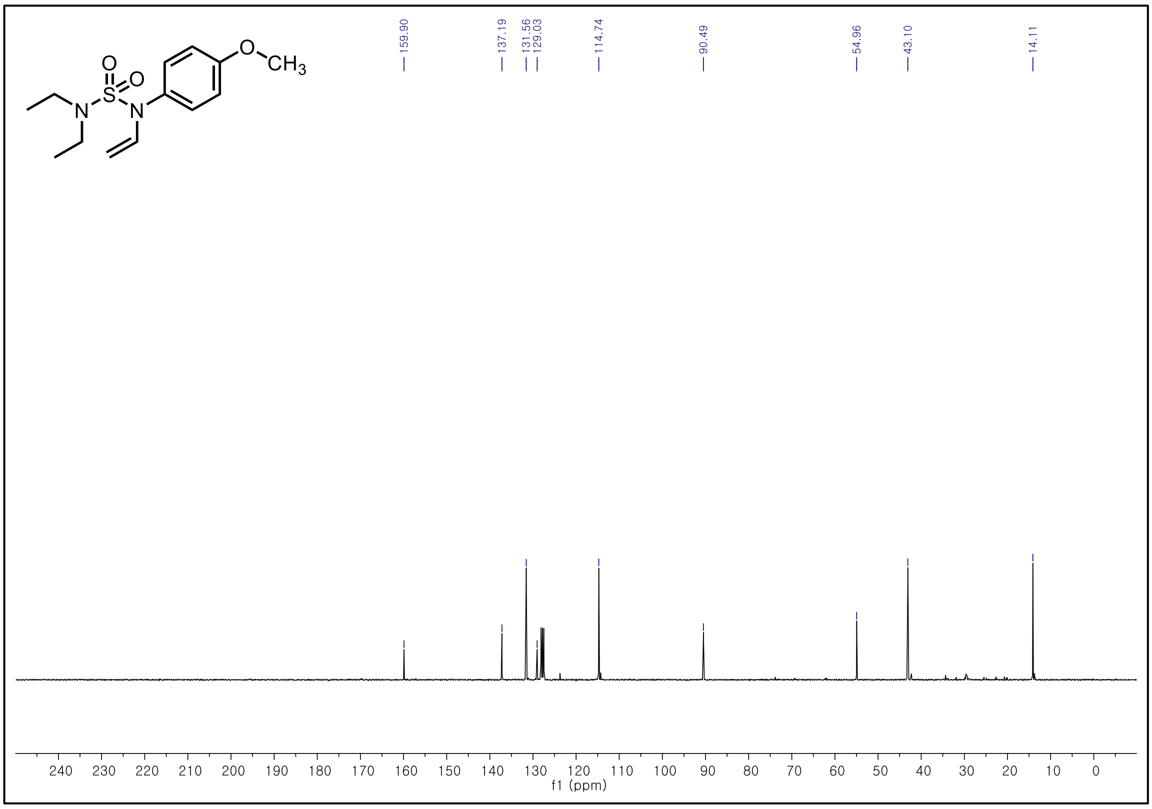

Supplementary Figure 38. ^{1}H NMR (**VS10**) (C_6D_6, 300 MHz), and ^{13}C NMR (**VS10**) (C_6D_6, 75 MHz)

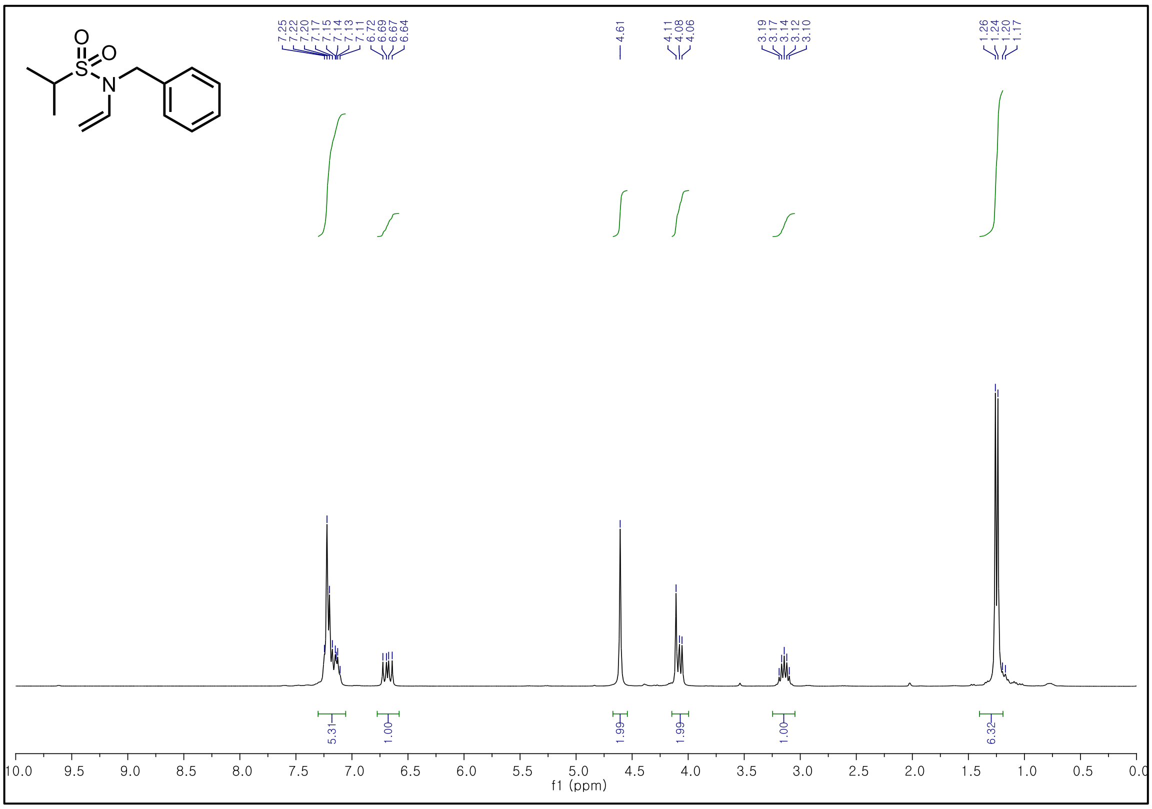

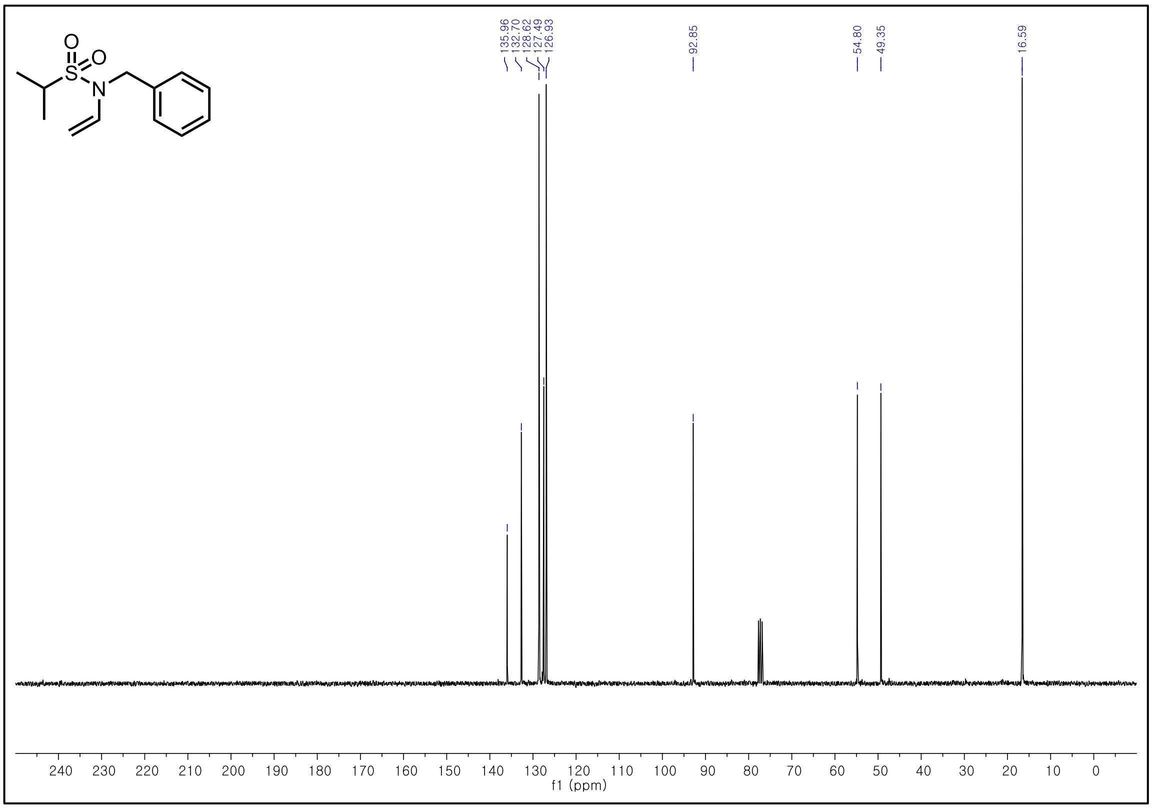

Supplementary Figure 39. ^{1}H NMR (**VS11**) ($CDCl_3$, 300 MHz), and ^{13}C NMR (**VS11**) ($CDCl_3$, 75 MHz)

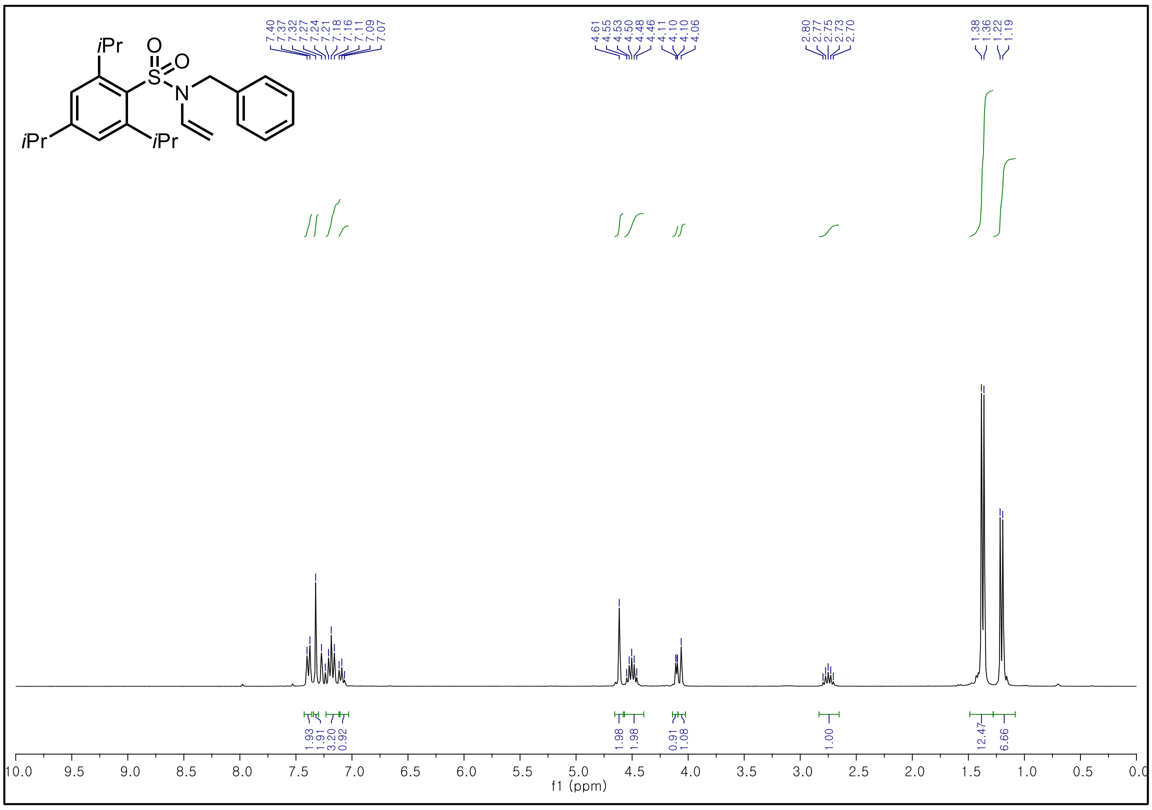

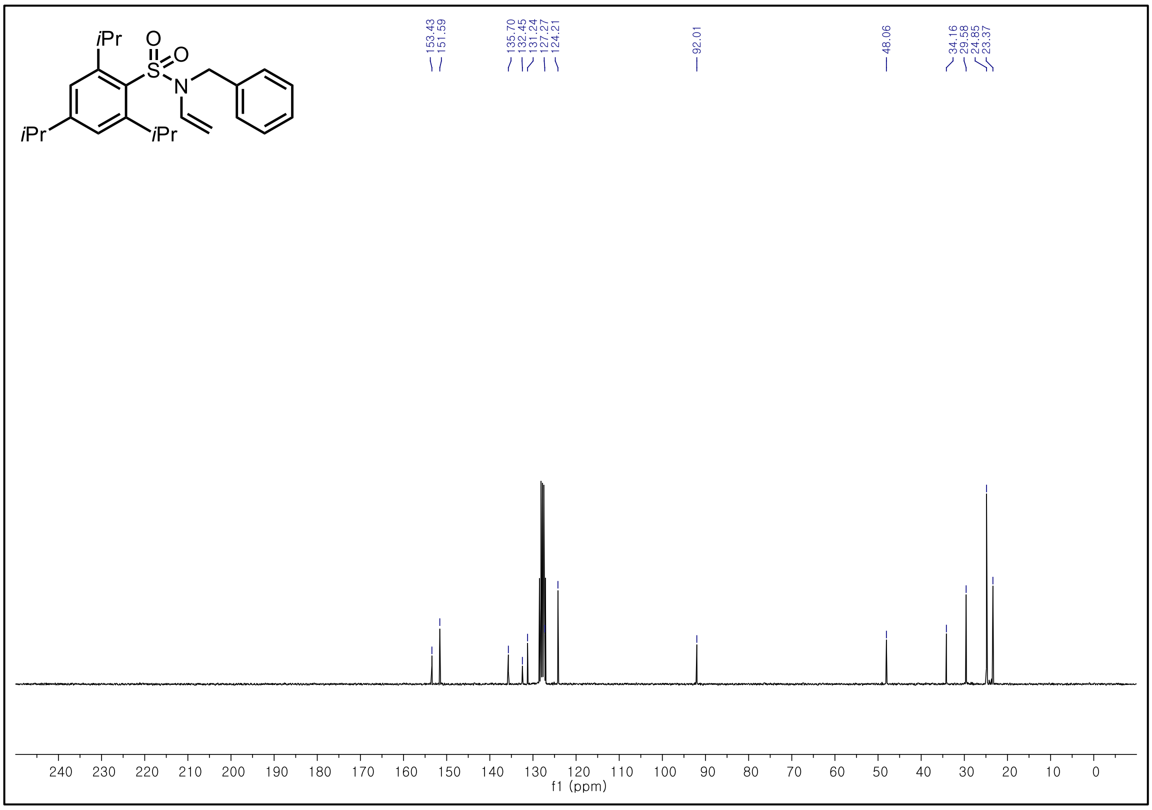

Supplementary Figure 40. ^{1}H NMR (**VS12**) (C_6D_6, 300 MHz), and ^{13}C NMR (**VS12**) (C_6D_6, 75 MHz)

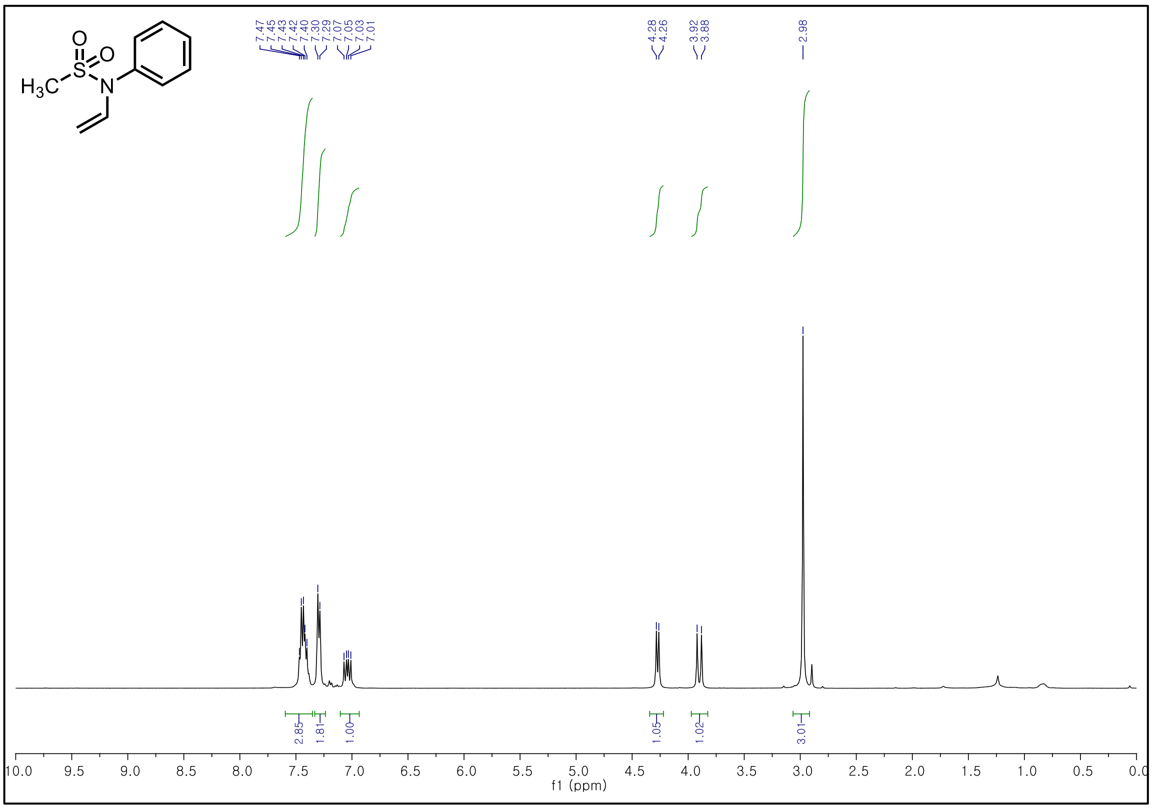

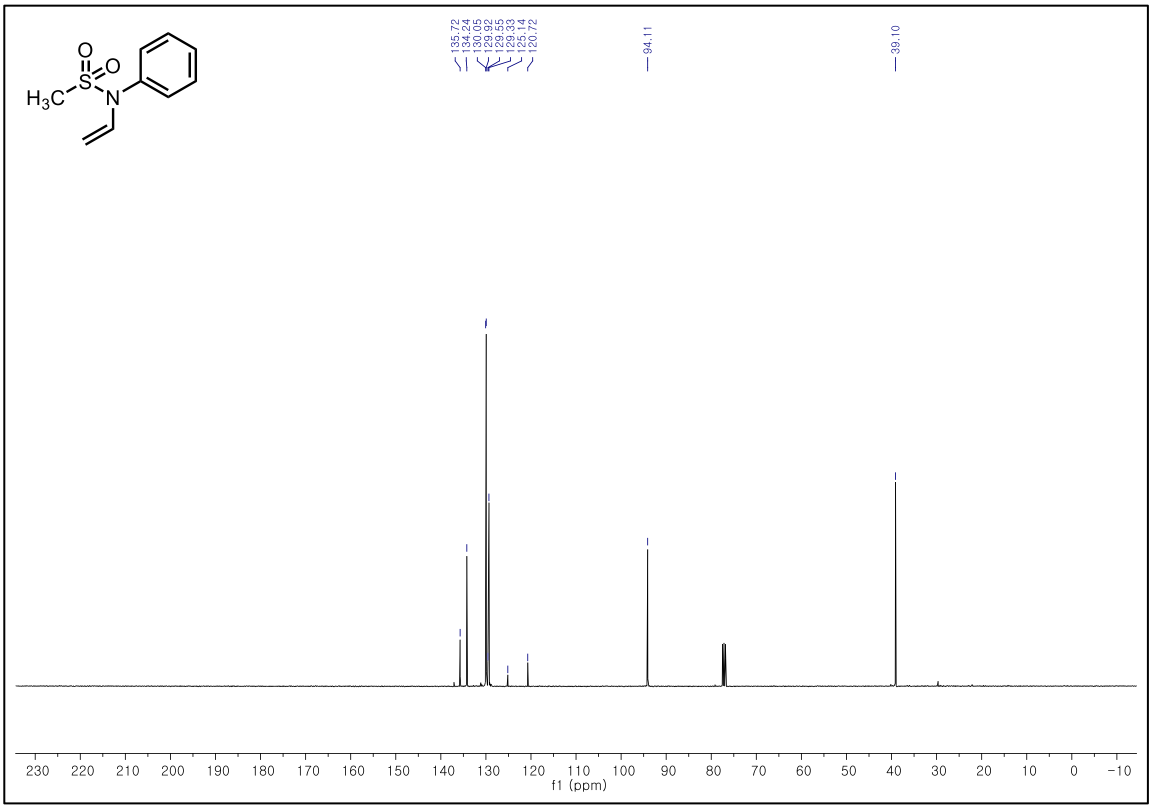

Supplementary Figure 41. ^{1}H NMR (**VS13**) ($CDCl_3$, 400 MHz), and ^{13}C NMR (**VS13**) ($CDCl_3$, 100 MHz)

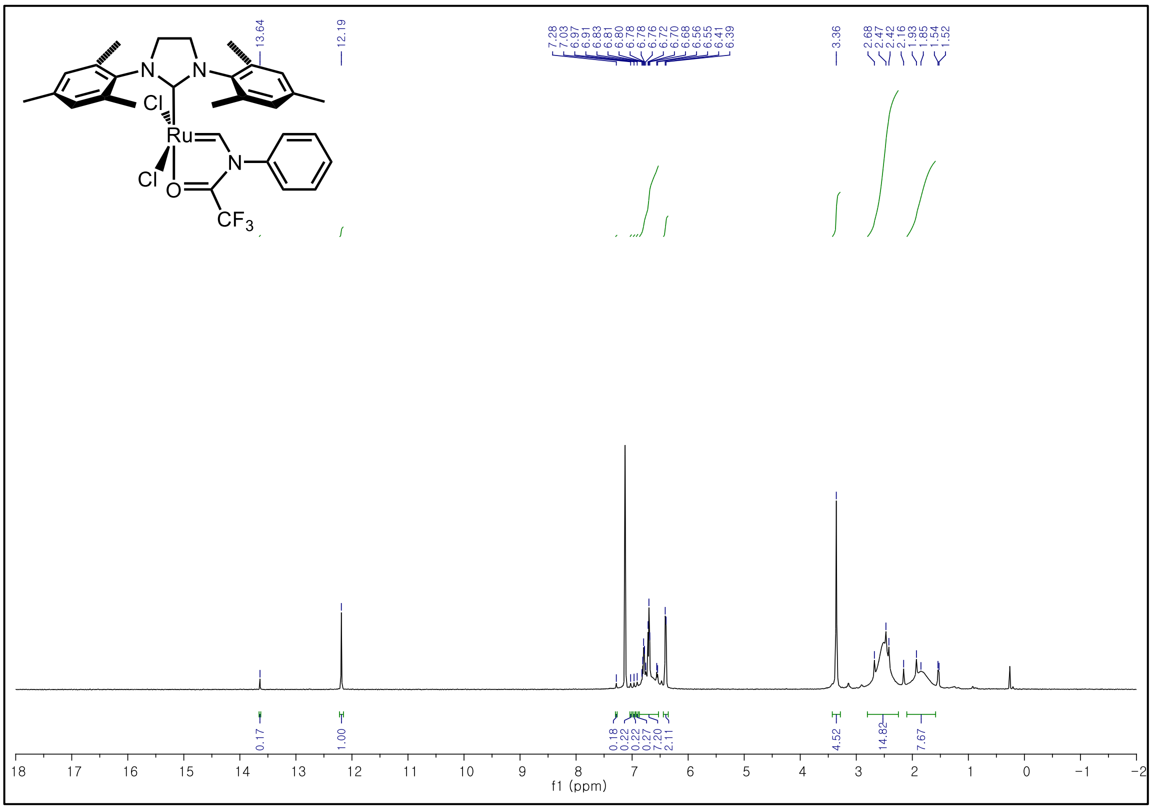

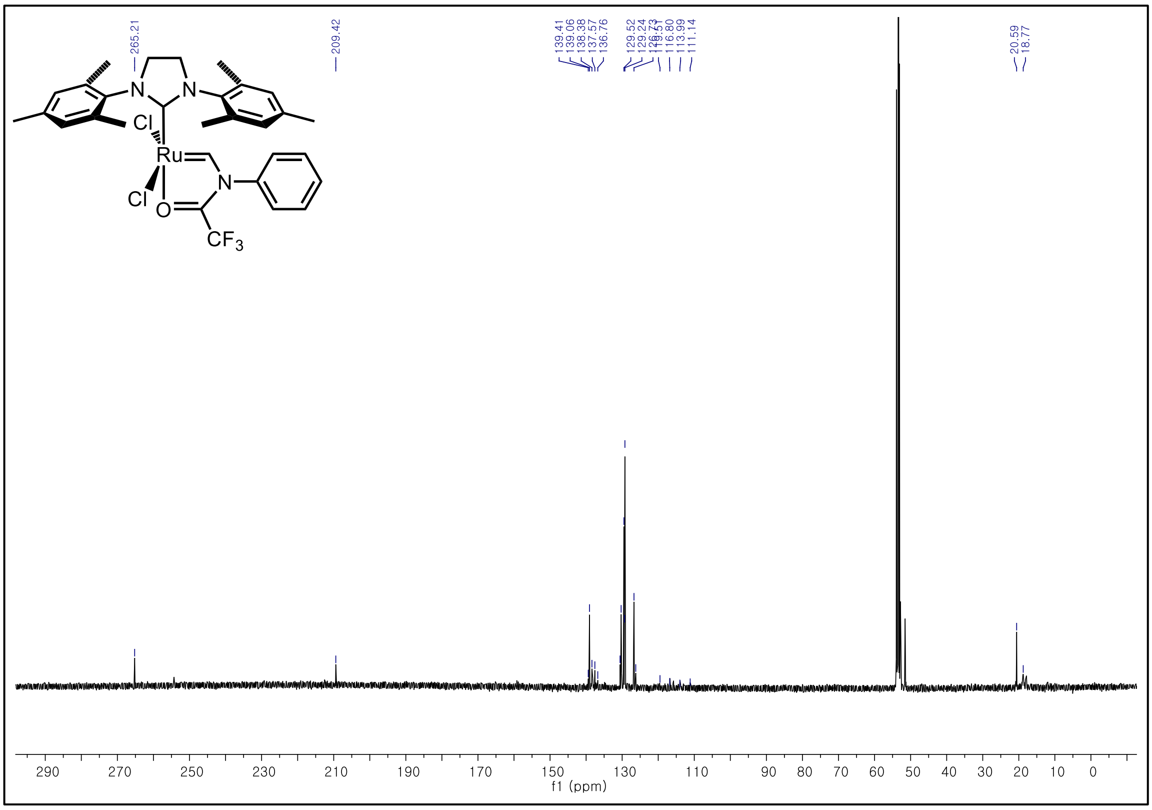

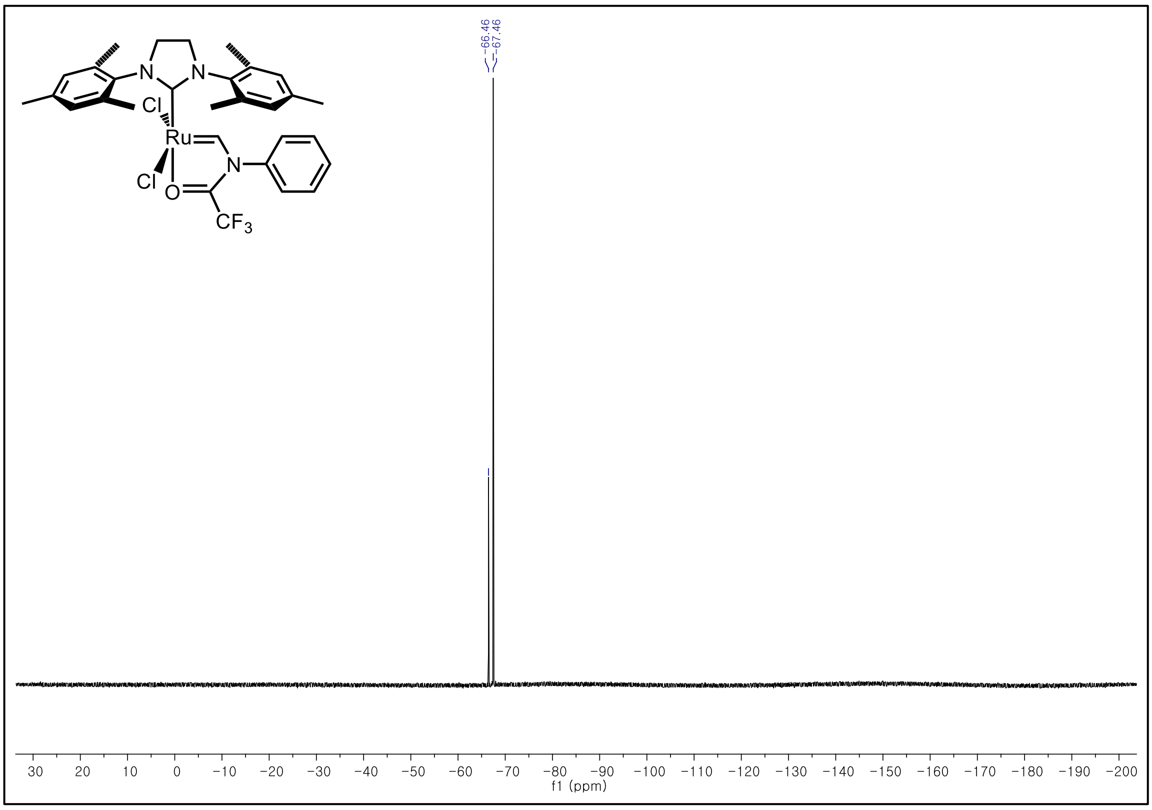

Supplementary Figure 42. ^{1}H NMR (**Ru-amide**) (C_6D_6, 400 MHz), ^{13}C NMR (**Ru-amide**) (CD_2Cl_2, 100 MHz) and ^{19}F NMR (**Ru-amide**) (CD_2Cl_2, 376 MHz)

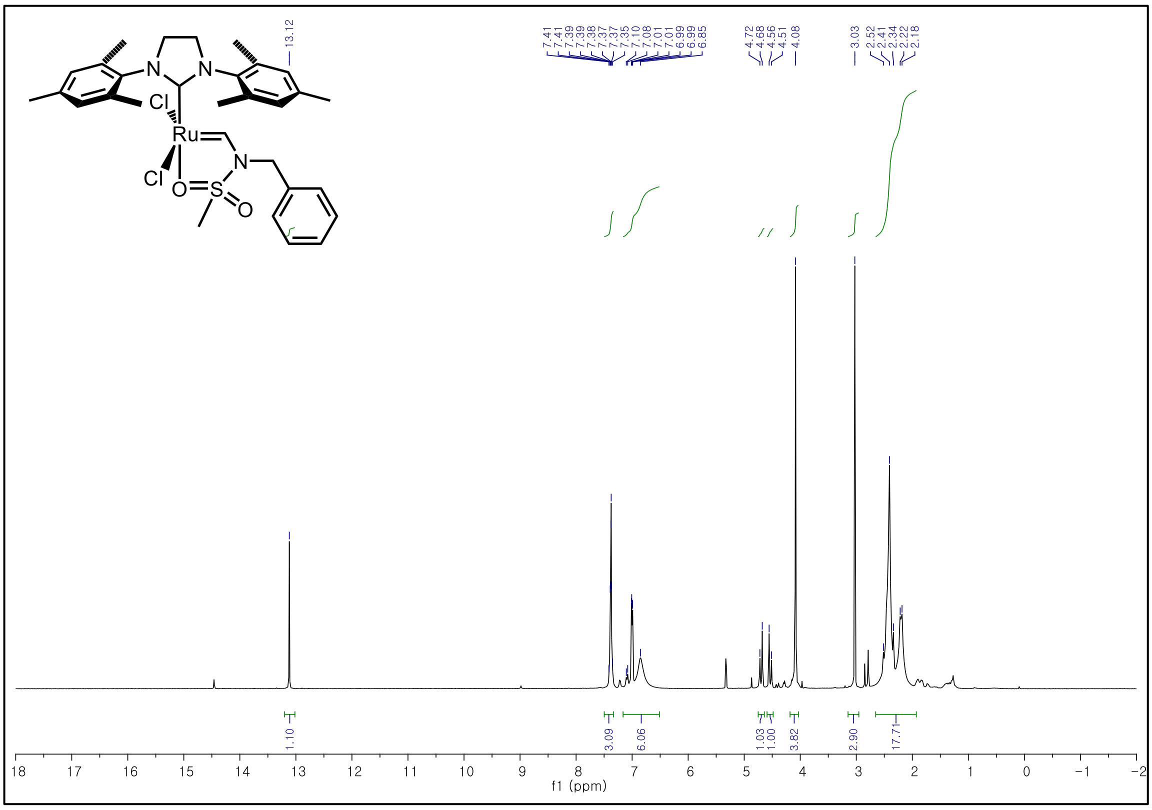

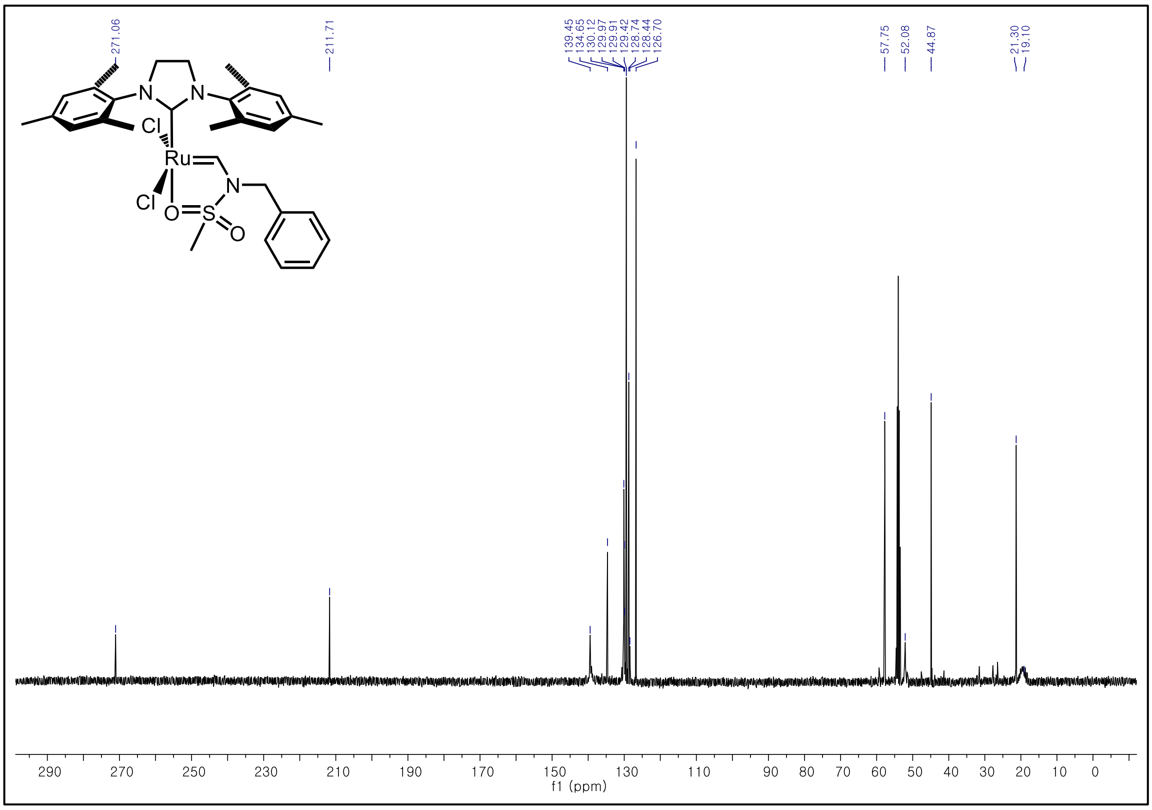

Supplementary Figure 43. ^{1}H NMR (**Ru-1**) (CD_2Cl_2, 400 MHz), and ^{13}C NMR (**Ru-1**) (CD_2Cl_2, 100 MHz)

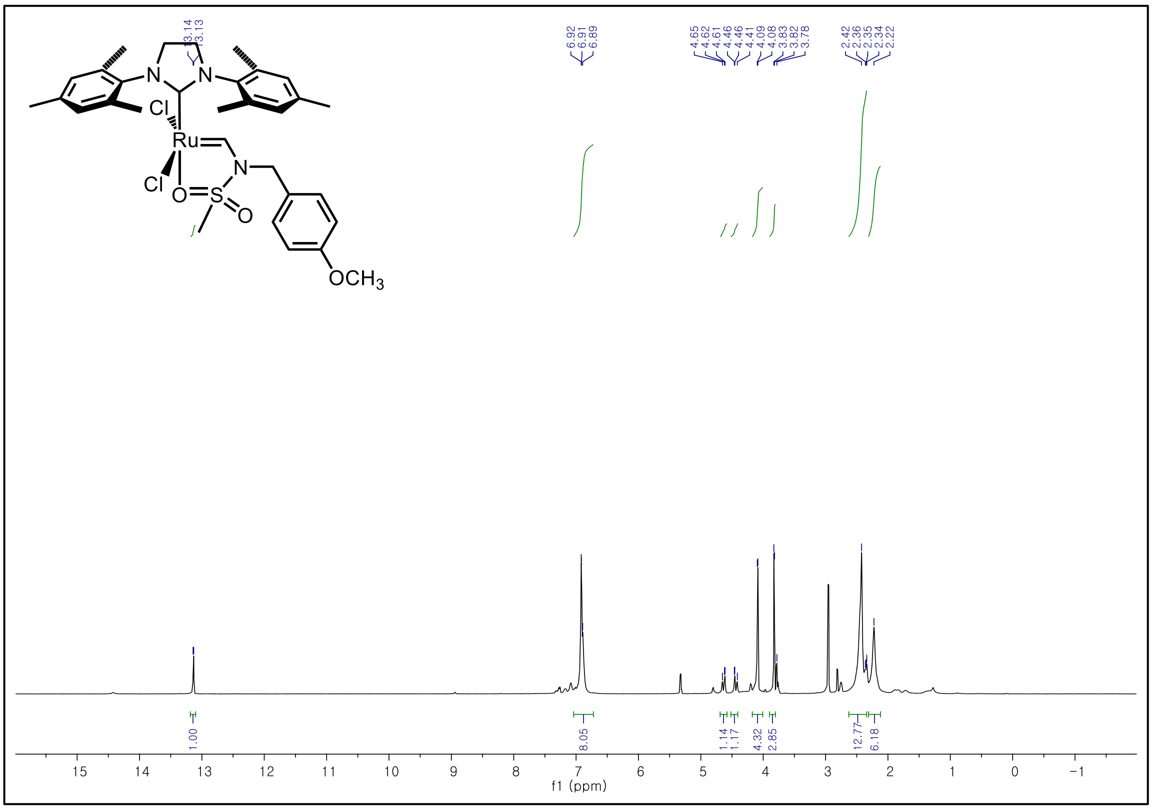

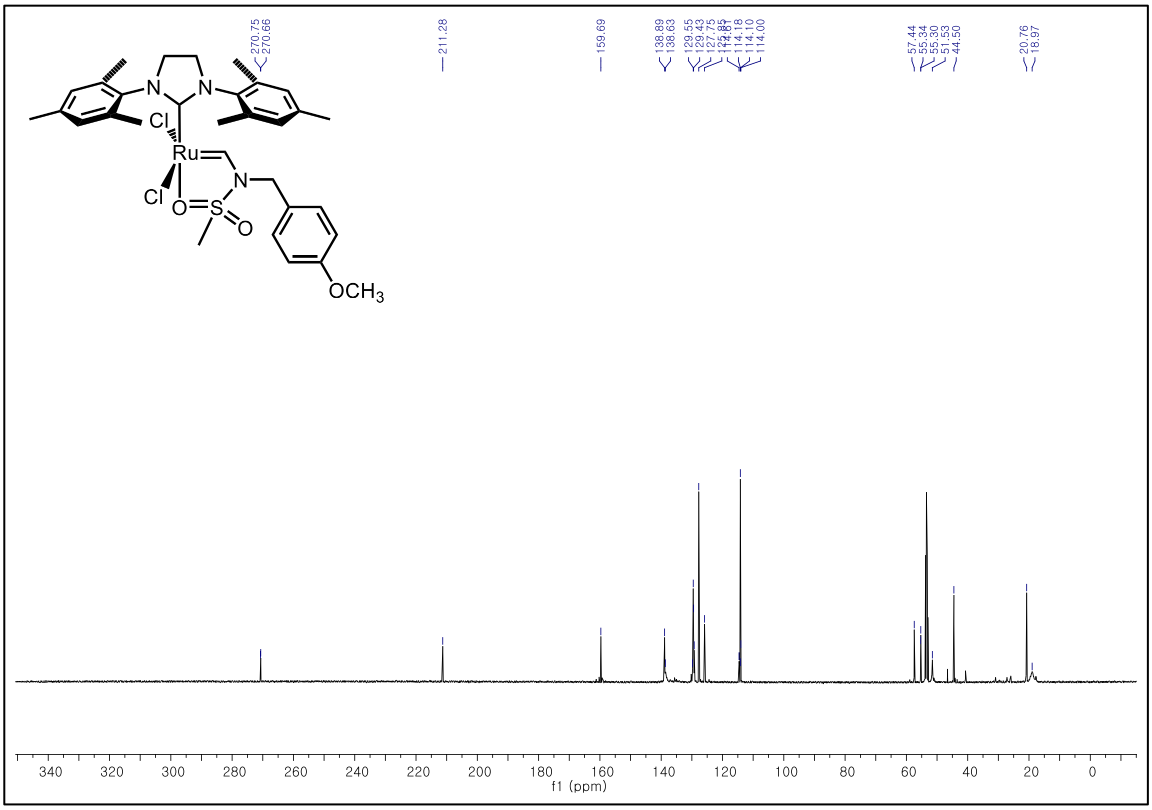

Supplementary Figure 44. ^{1}H NMR (**Ru-2**) (CD_2Cl_2, 400 MHz), and ^{13}C NMR (**Ru-2**) (CD_2Cl_2, 100 MHz)

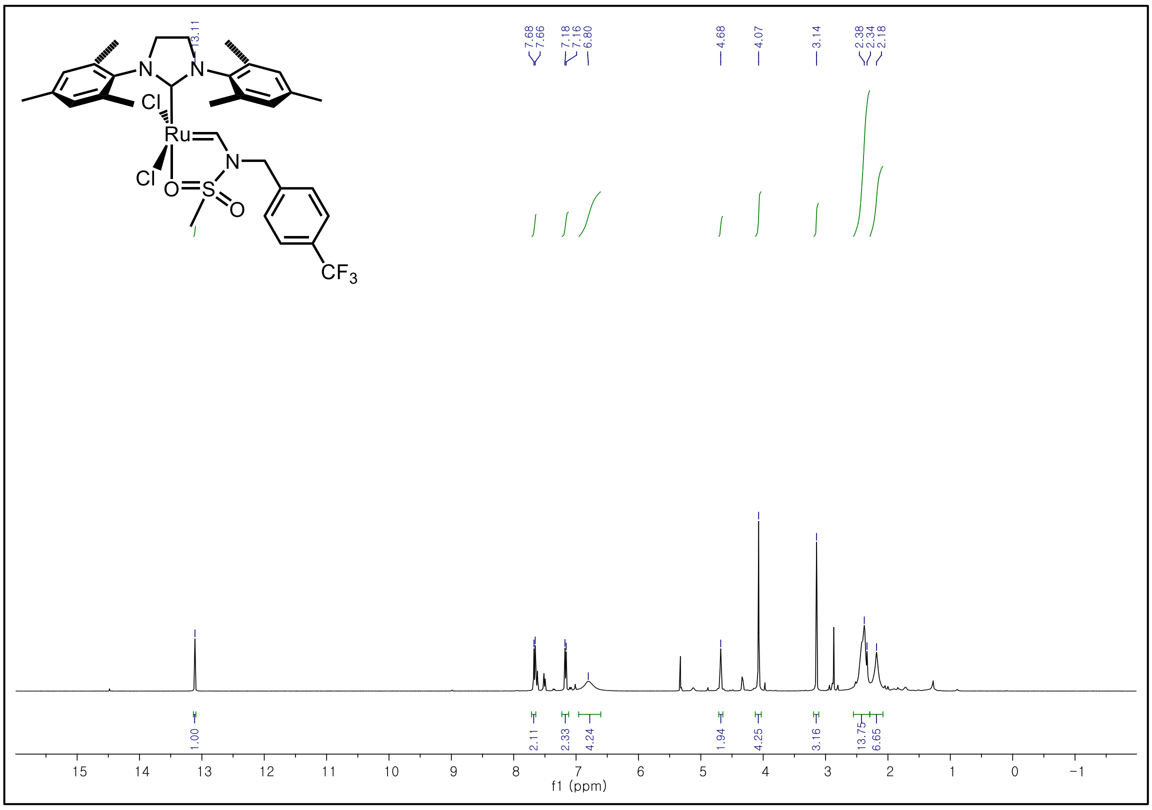
13.11
7.68
7.66
7.18
7.16
6.80
4.68
4.07
3.14
2.38
2.34
2.18
Cl
Ru
N
O
S
CF3
1.00
2.11
2.33
4.24
1.94
4.25
3.16
13.75
6.65
15 14 13 12 11 10 9 8 7 6 5 4 3 2 1 0 −1
f1 (ppm)

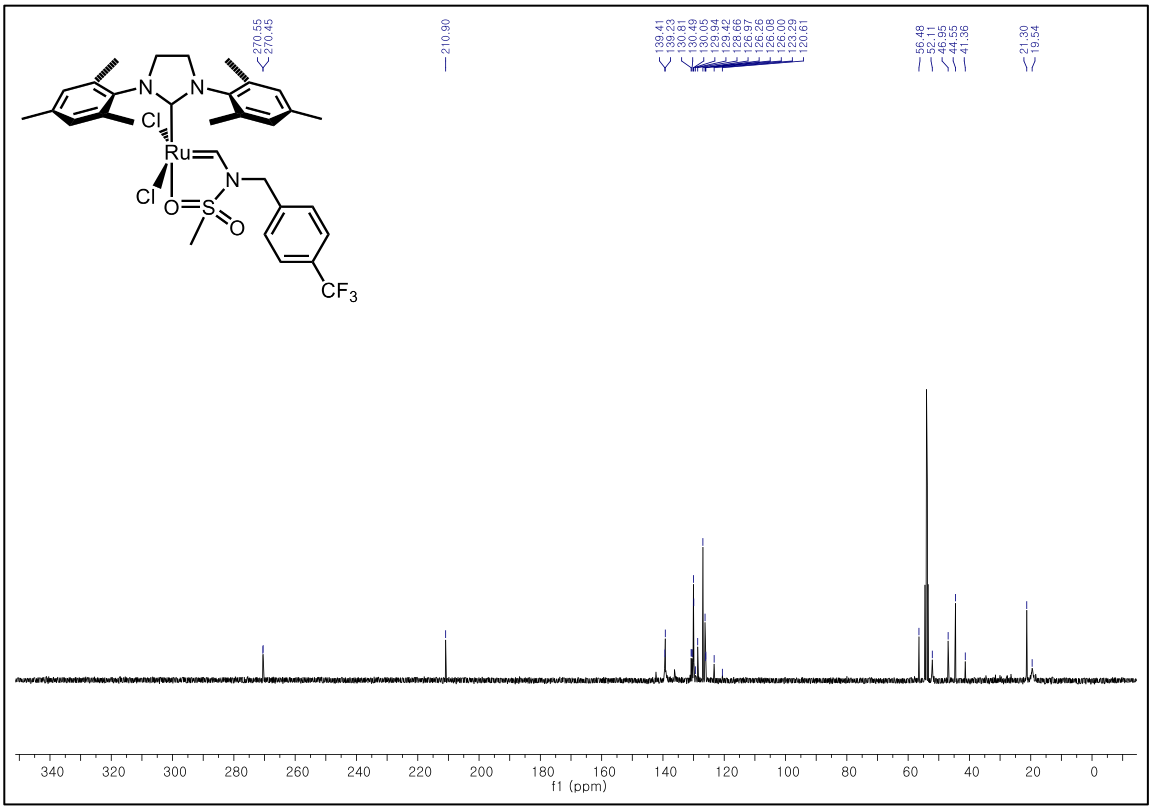
270.55
270.45
210.90
139.41
139.23
130.81
130.49
130.05
129.94
129.42
128.66
126.97
126.26
126.08
126.00
123.29
120.61
56.48
52.11
46.95
44.55
41.36
21.30
19.54
Cl
Ru
N
O
S
CF3
340 320 300 280 260 240 220 200 180 160 140 120 100 80 60 40 20 0
f1 (ppm)

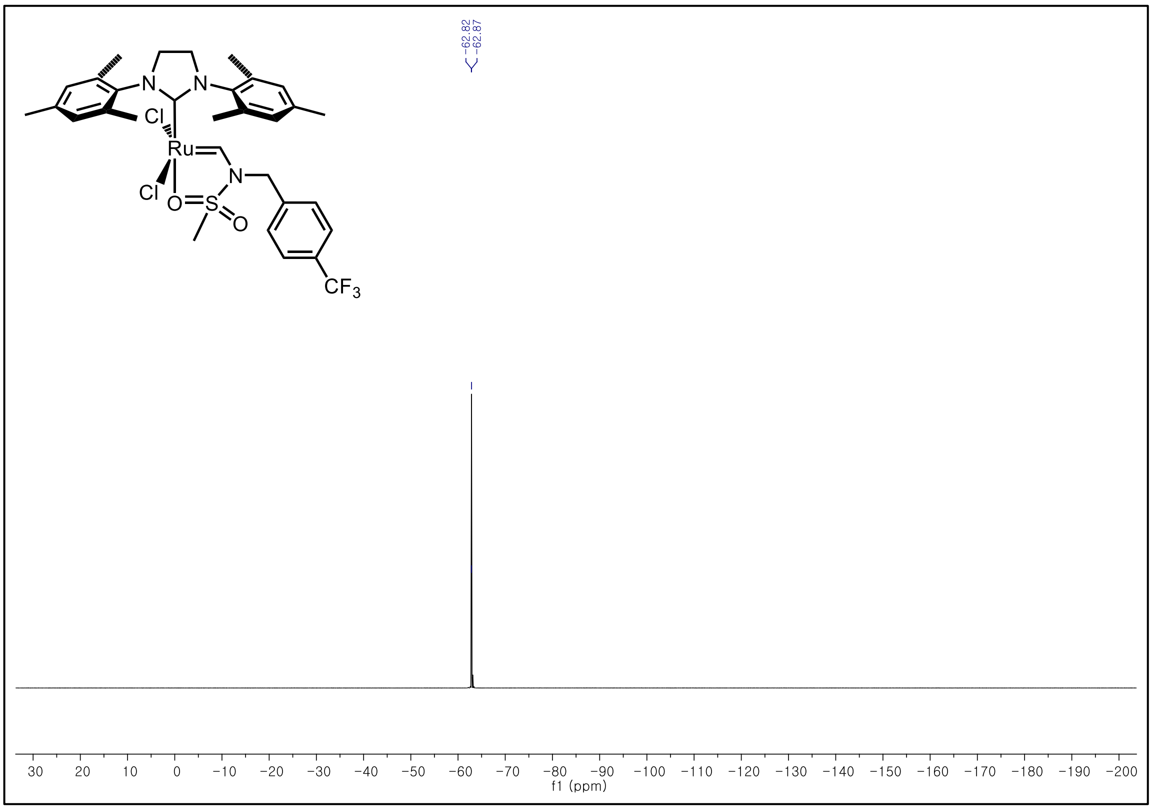

Supplementary Figure 45. ^{1}H NMR (**Ru-3**) (CD_2Cl_2, 300 MHz), ^{13}C NMR (**Ru-3**) (CD_2Cl_2, 125 MHz), and ^{19}F NMR (**Ru-3**) (CD_2Cl_2, 376 MHz)

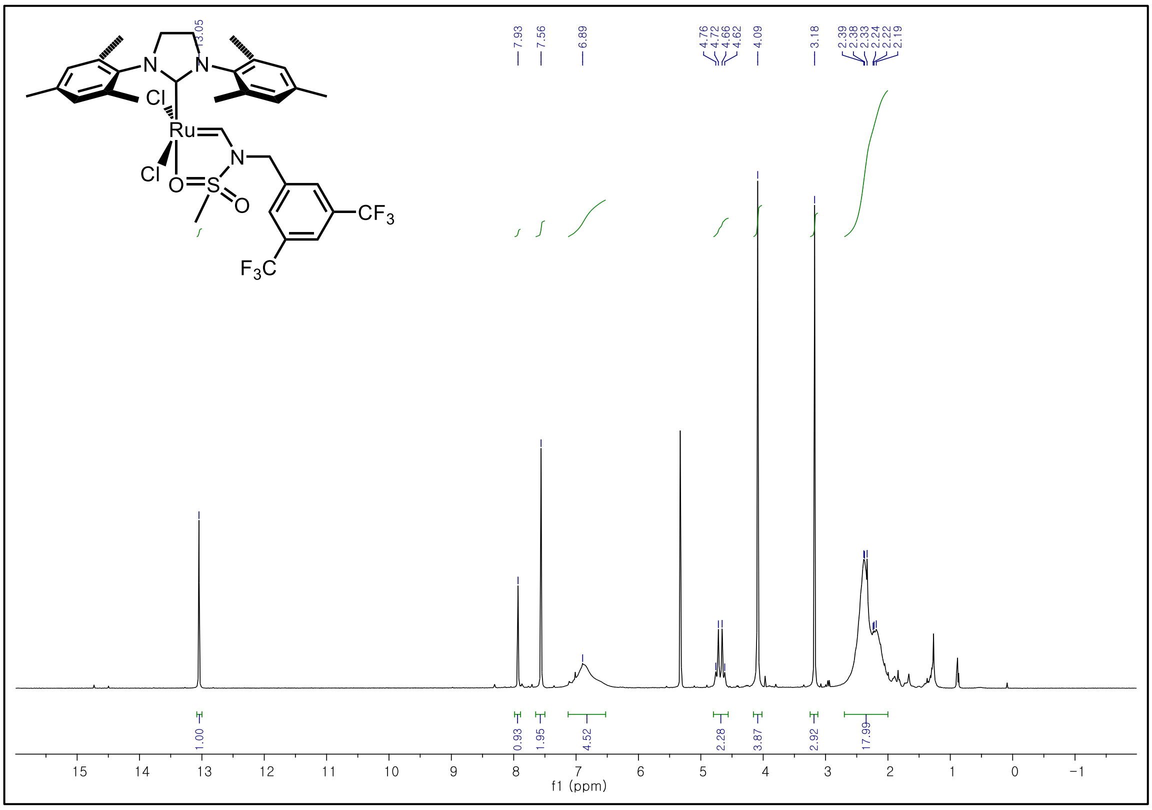
13.05
7.93
7.56
6.89
4.76
4.72
4.66
4.62
4.09
3.18
2.39
2.38
2.33
2.24
2.22
2.19
1.00
0.93
1.95
4.52
2.28
3.87
2.92
17.99
CF3
F3C
f1 (ppm)

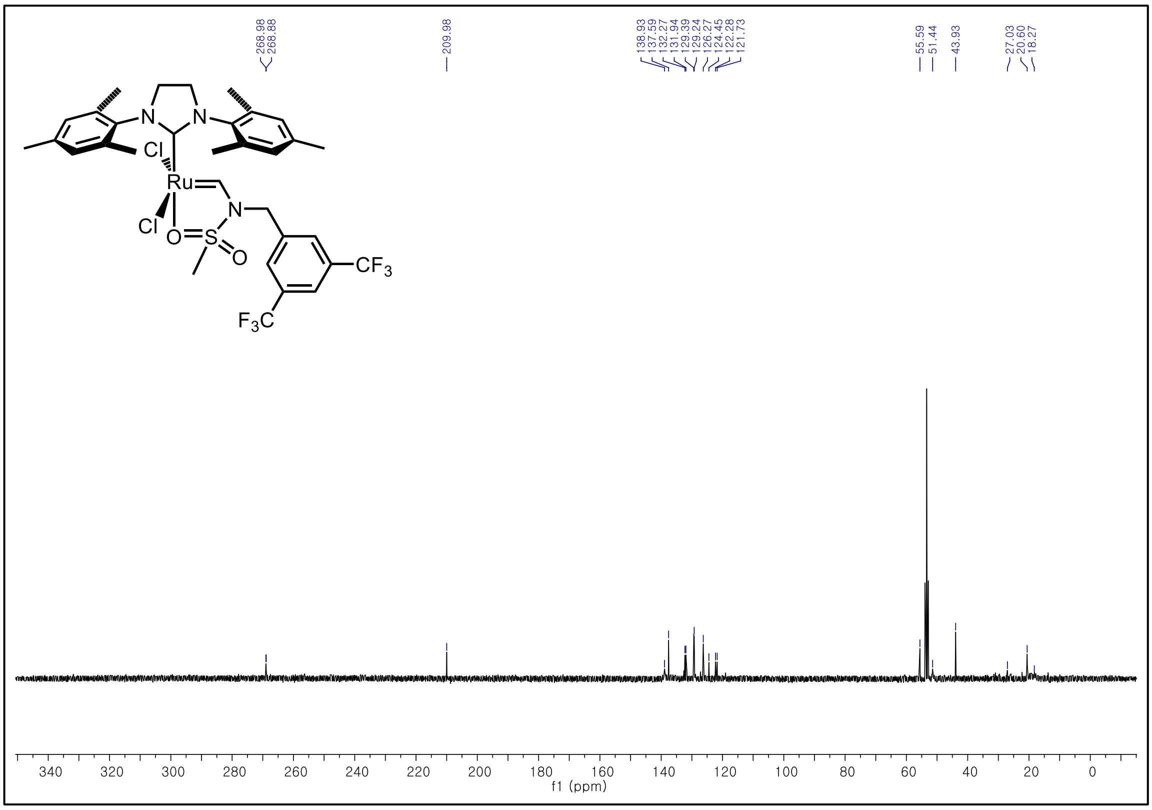
268.98
268.88
209.98
138.93
137.59
132.27
131.94
129.39
129.24
126.27
124.45
122.28
121.73
55.59
51.44
43.93
27.03
20.60
18.27
CF3
F3C
f1 (ppm)

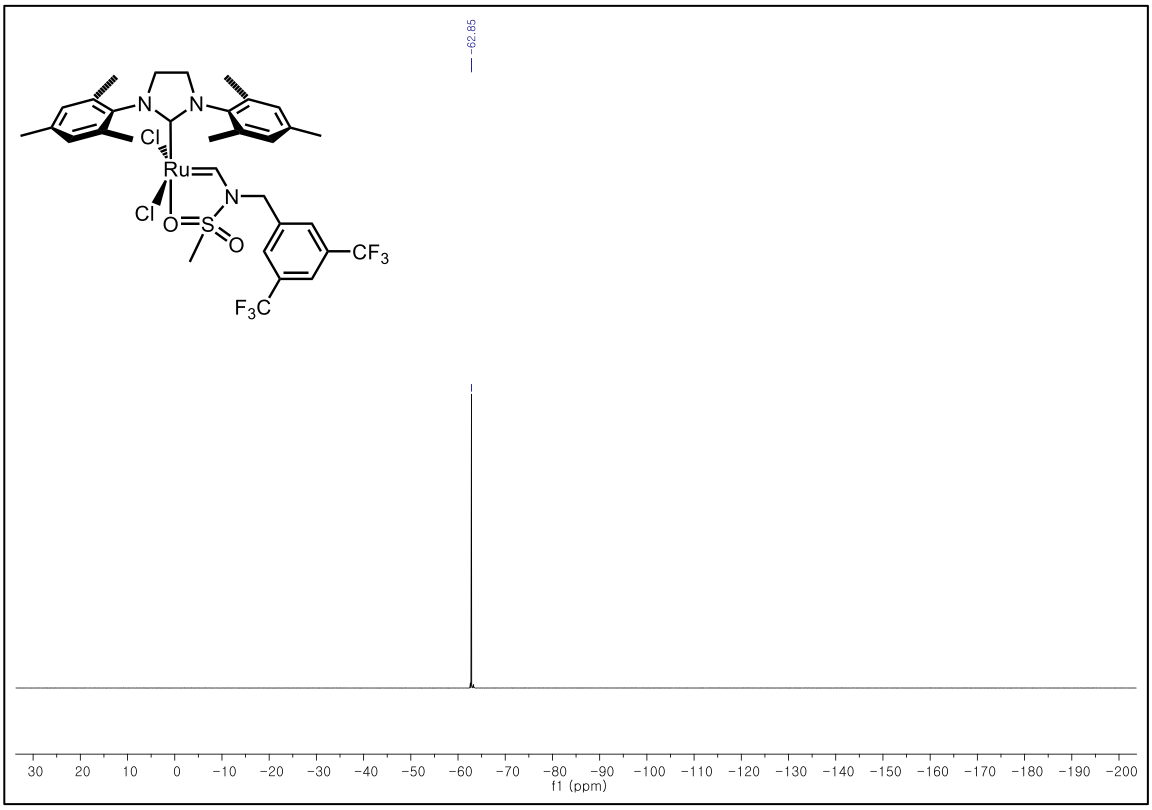

Supplementary Figure 46. ^{1}H NMR (**Ru-4**) (CD_2Cl_2, 400 MHz), ^{13}C NMR (**Ru-4**) (CD_2Cl_2, 125 MHz), and ^{19}F NMR (**Ru-4**) (CD_2Cl_2, 376 MHz)

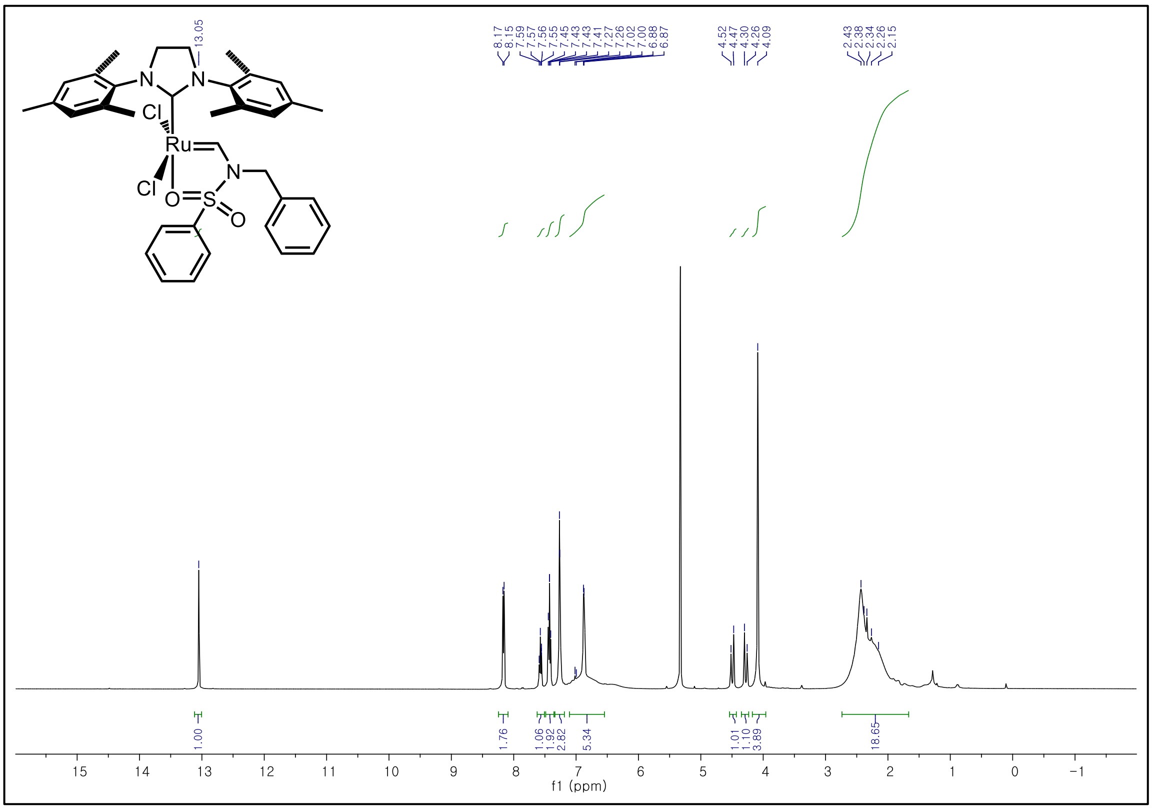

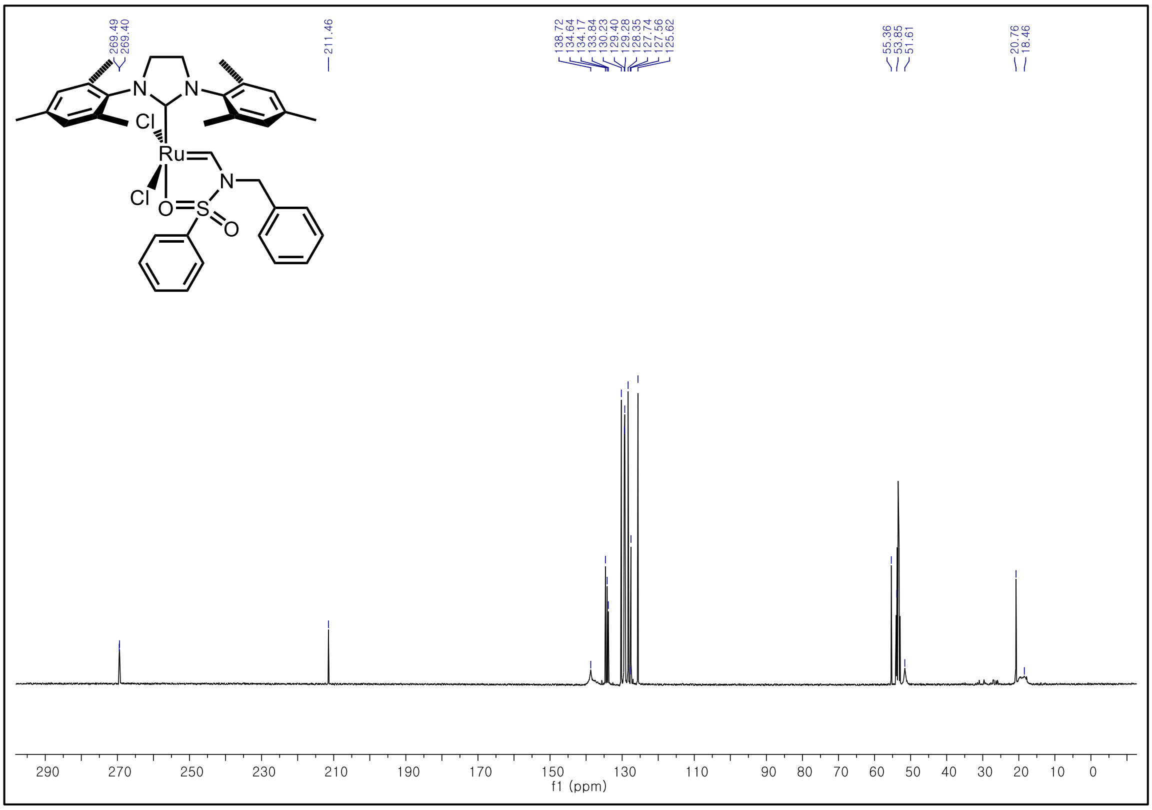

Supplementary Figure 47. ^{1}H NMR (**Ru-5**) (CD_2Cl_2, 400 MHz), and ^{13}C NMR (**Ru-5**) (CD_2Cl_2, 100 MHz)

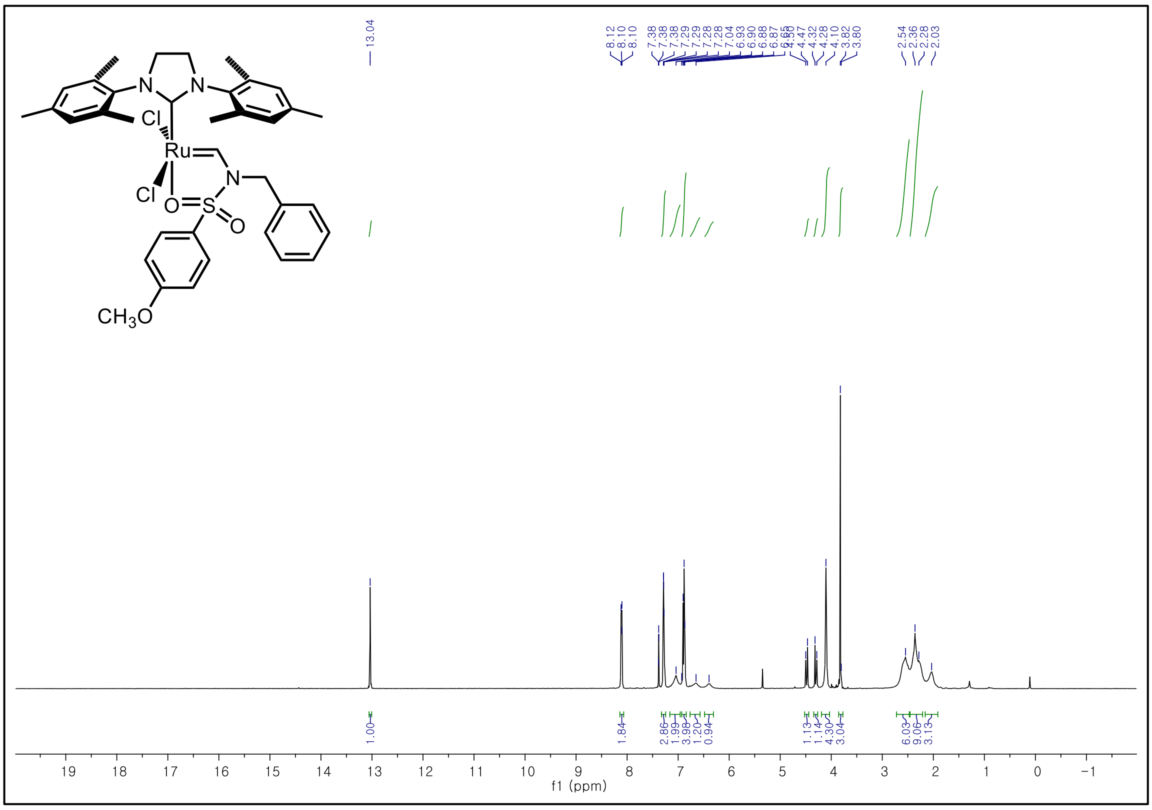

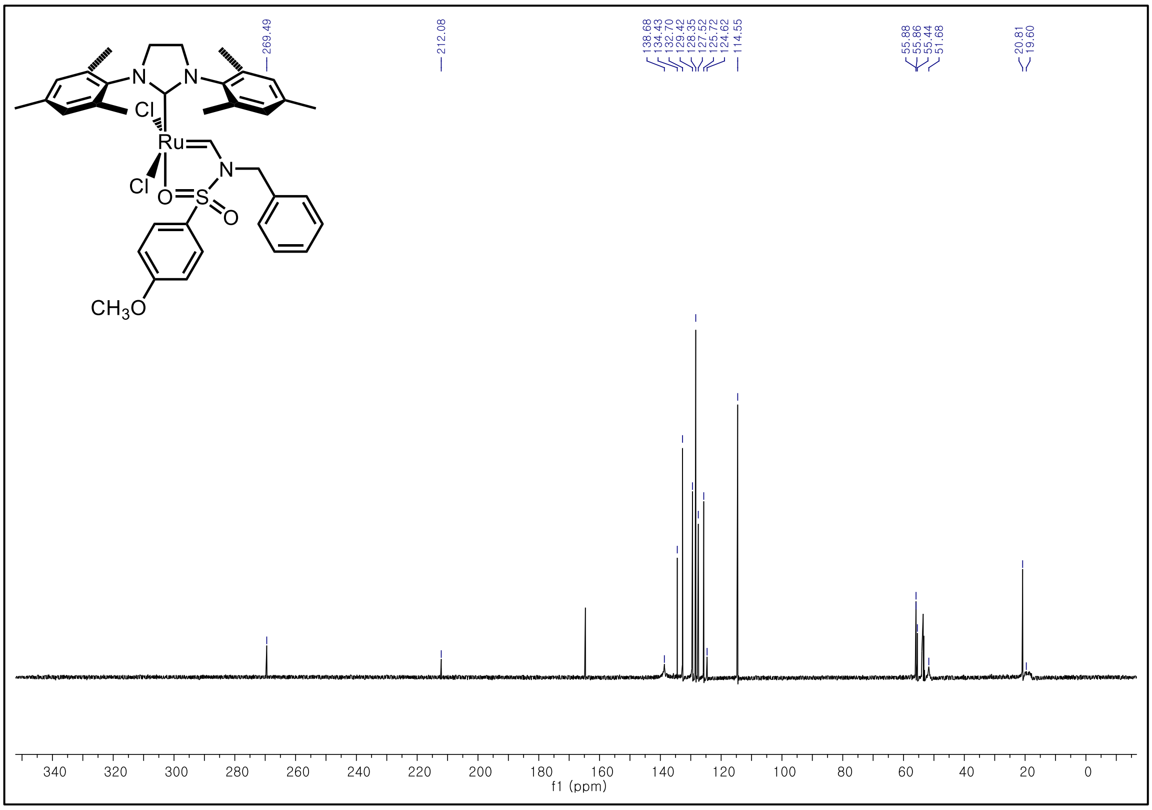

Supplementary Figure 48. ^{1}H NMR (**Ru-6**) (CD_2Cl_2, 500 MHz), and ^{13}C NMR (**Ru-6**) (CD_2Cl_2, 125 MHz)

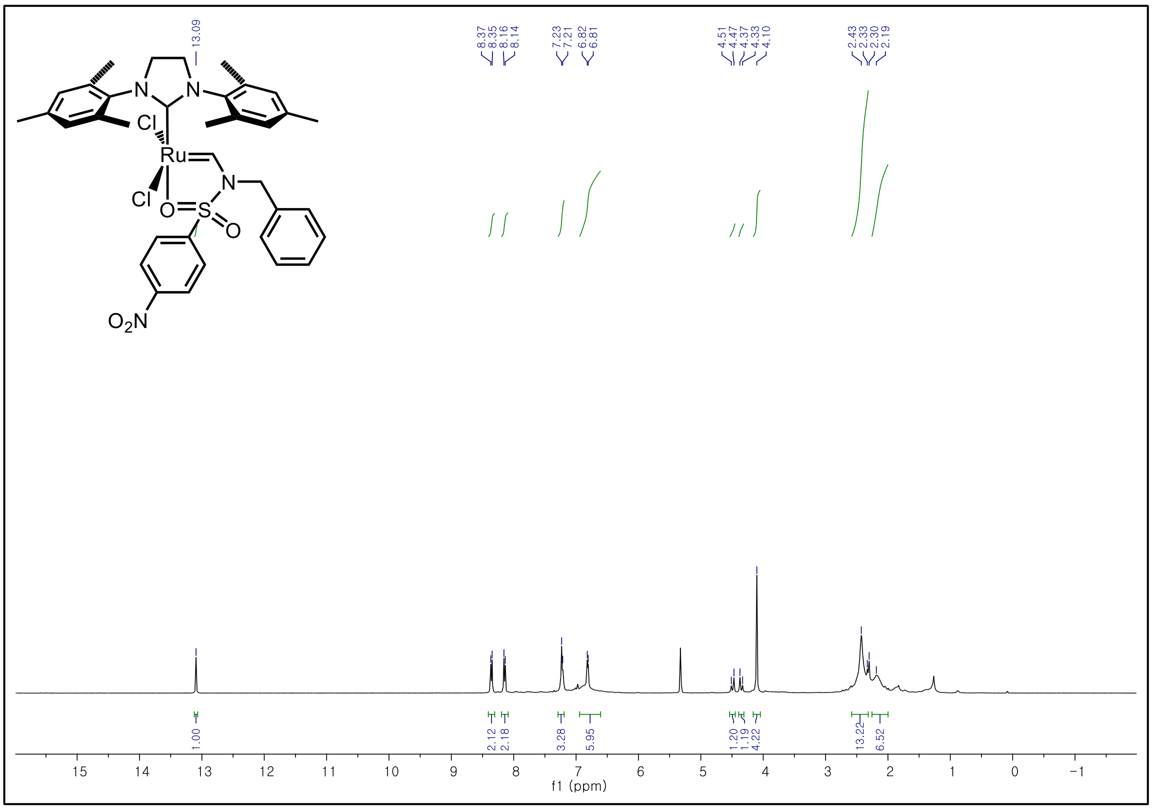

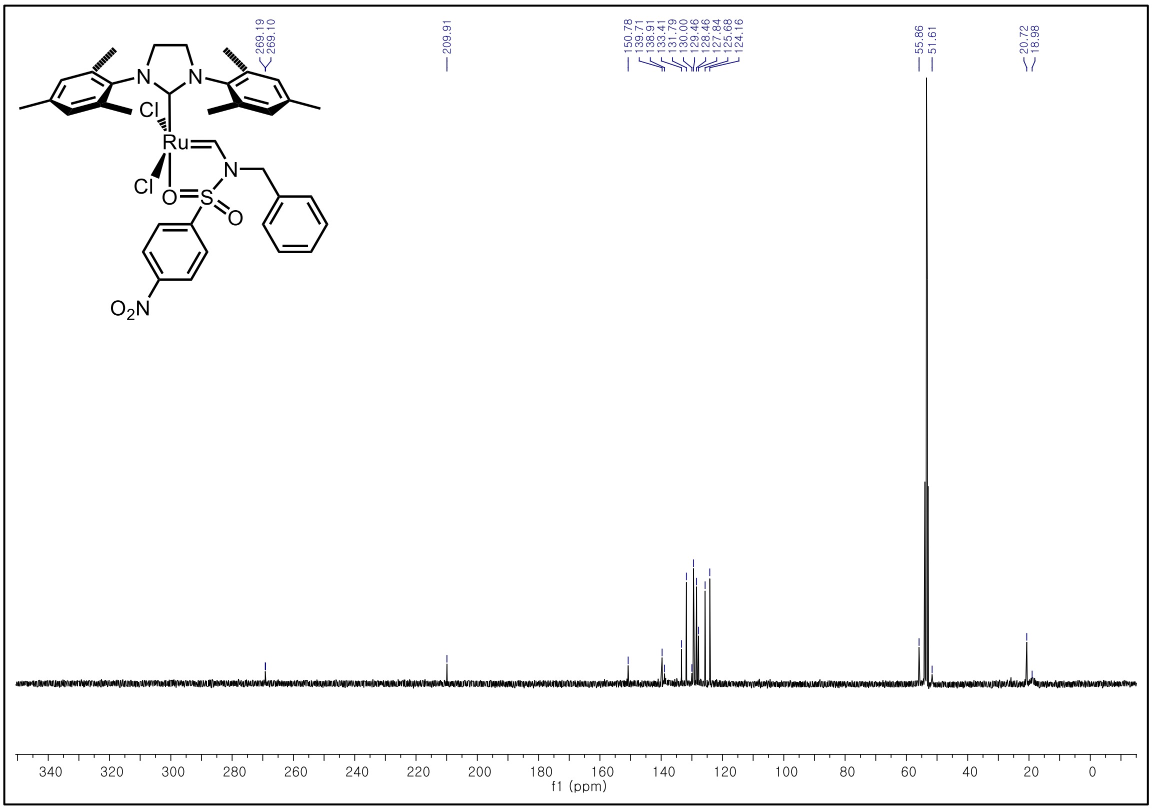

Supplementary Figure 49. ^{1}H NMR (**Ru-7**) (CD_2Cl_2, 400 MHz), and ^{13}C NMR (**Ru-7**) (CD_2Cl_2, 125 MHz)

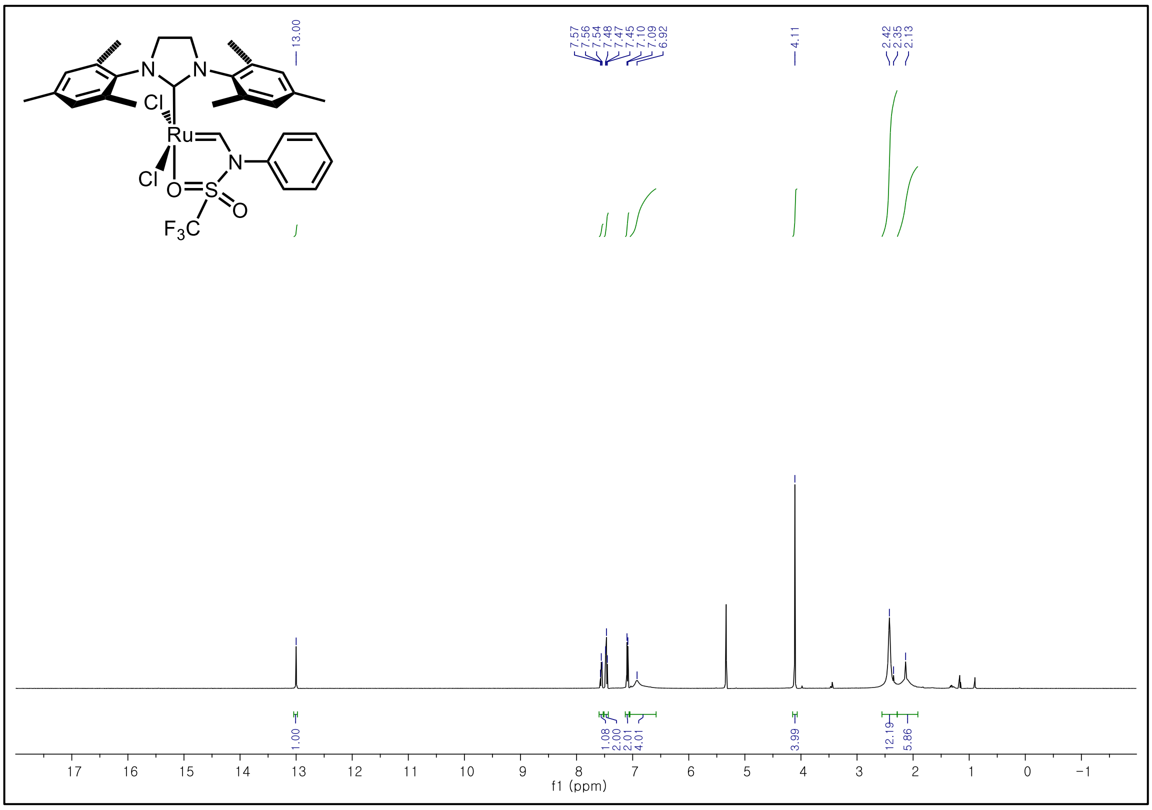
Cl
Ru
N
Cl
O
S
O
F3C
13.00
7.57
7.56
7.54
7.48
7.47
7.45
7.10
7.09
6.92
4.11
2.42
2.35
2.13
1.00
1.08
2.00
2.01
4.01
3.99
12.19
5.86
17 16 15 14 13 12 11 10 9 8 7 6 5 4 3 2 1 0 −1
f1 (ppm)

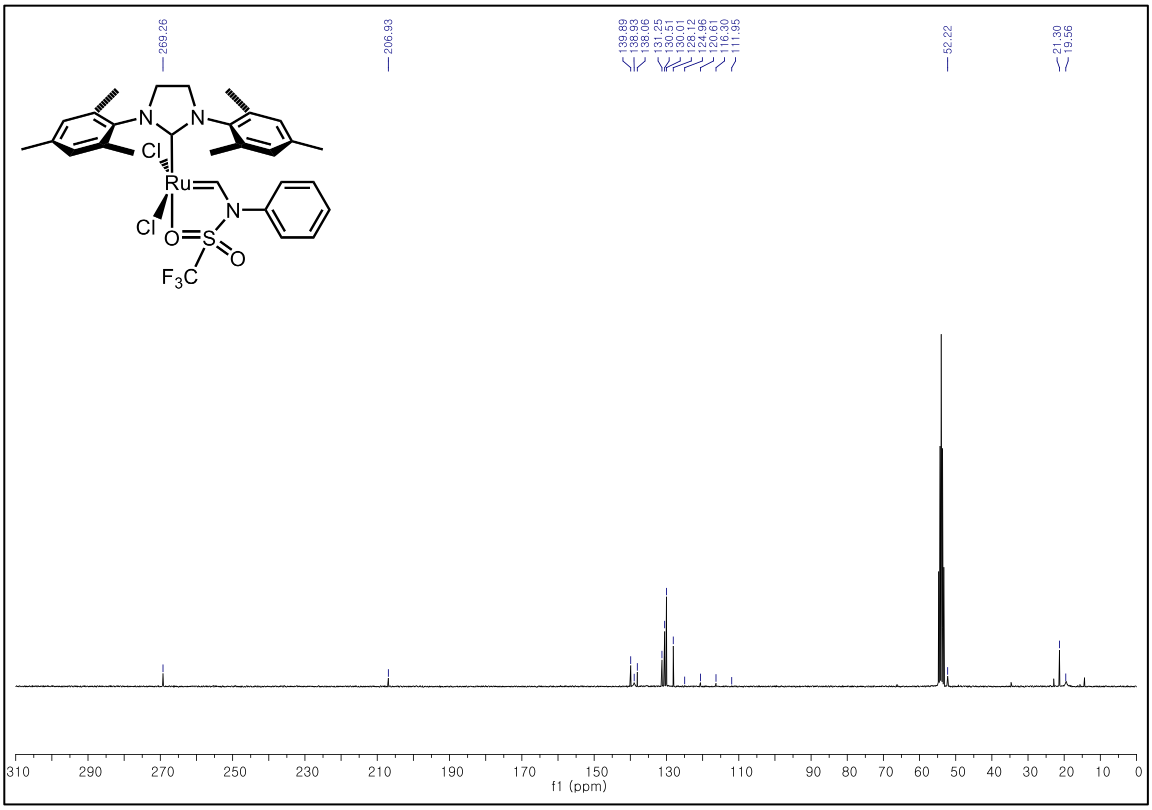
Cl
Ru
N
Cl
O
S
O
F3C
269.26
206.93
139.89
138.93
138.06
131.25
130.51
130.01
128.12
124.96
120.61
116.30
111.95
52.22
21.30
19.56
310 290 270 250 230 210 190 170 150 130 110 90 80 70 60 50 40 30 20 10 0
f1 (ppm)

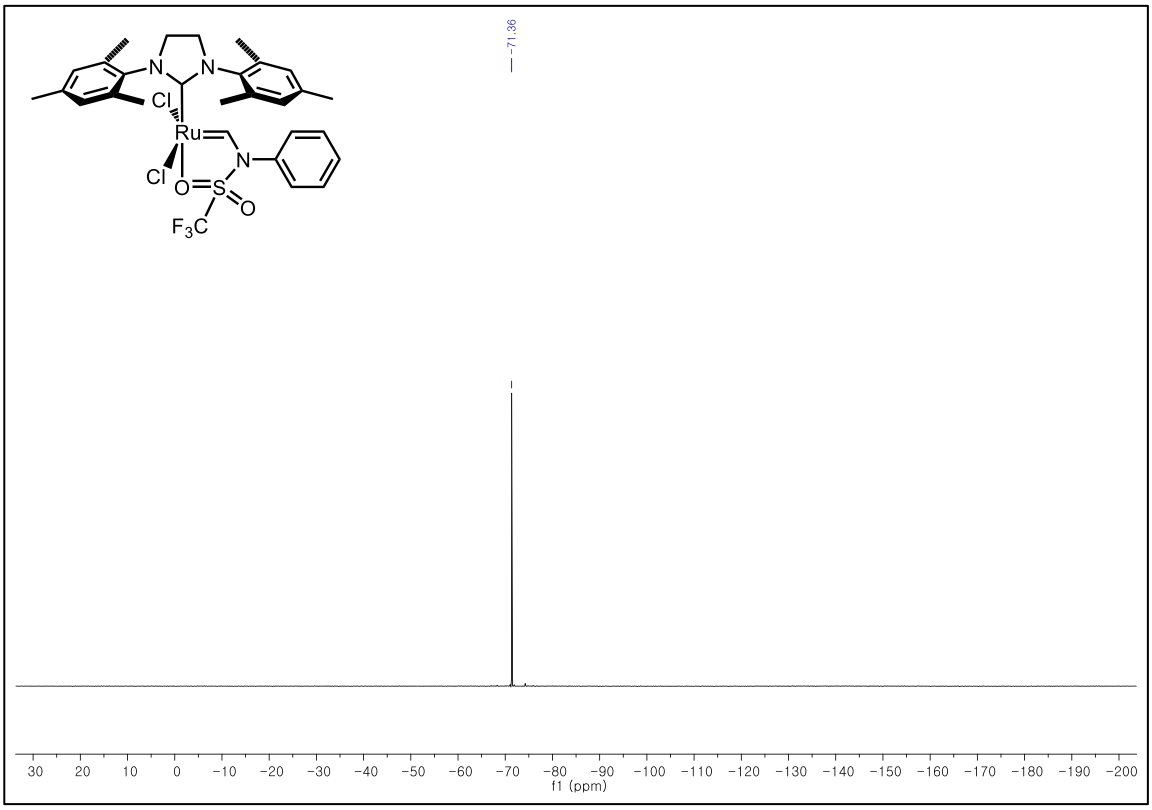

Supplementary Figure 50. ^{1}H NMR (**Ru-8**) (CD_2Cl_2, 400 MHz), ^{13}C NMR (**Ru-8**) (CD_2Cl_2, 75 MHz), and ^{19}F NMR (**Ru-8**) (CD_2Cl_2, 376 MHz)

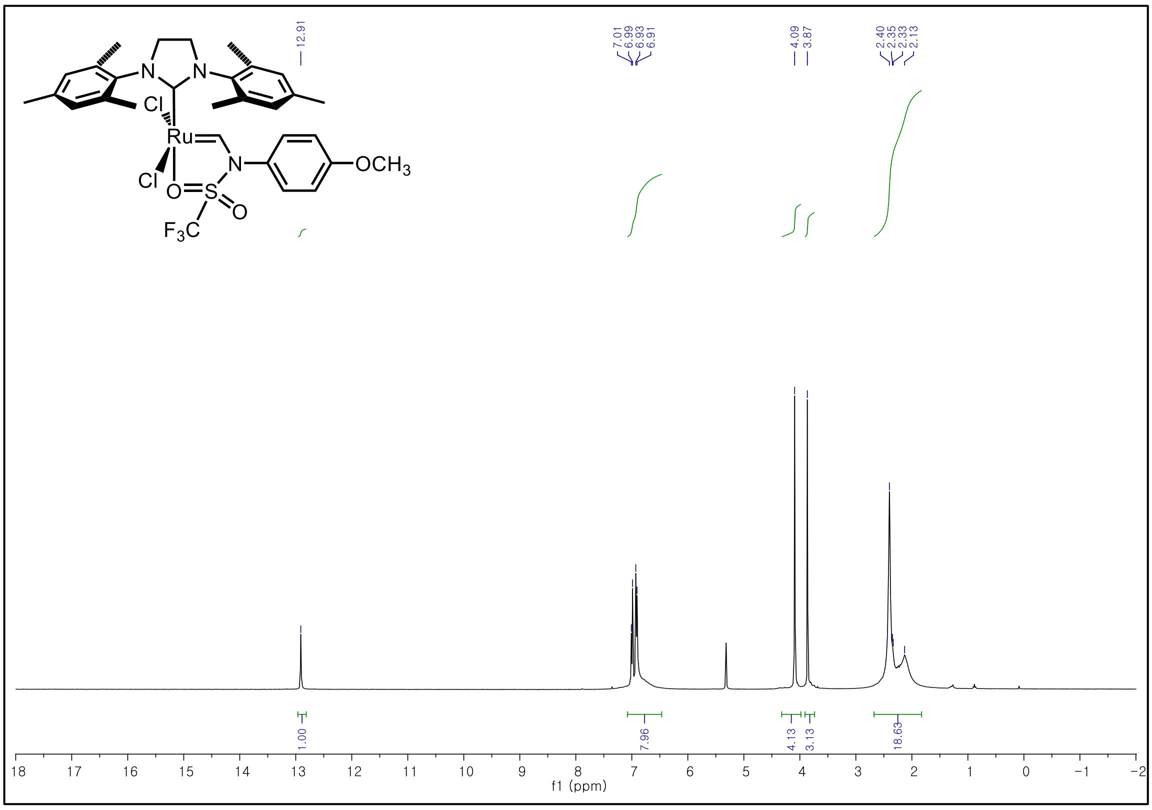
12.91
7.01
6.99
6.93
6.91
4.09
3.87
2.40
2.35
2.33
2.13
OCH3
Cl
Ru
N
O
S
F3C
1.00
7.96
4.13
3.13
18.63
f1 (ppm)

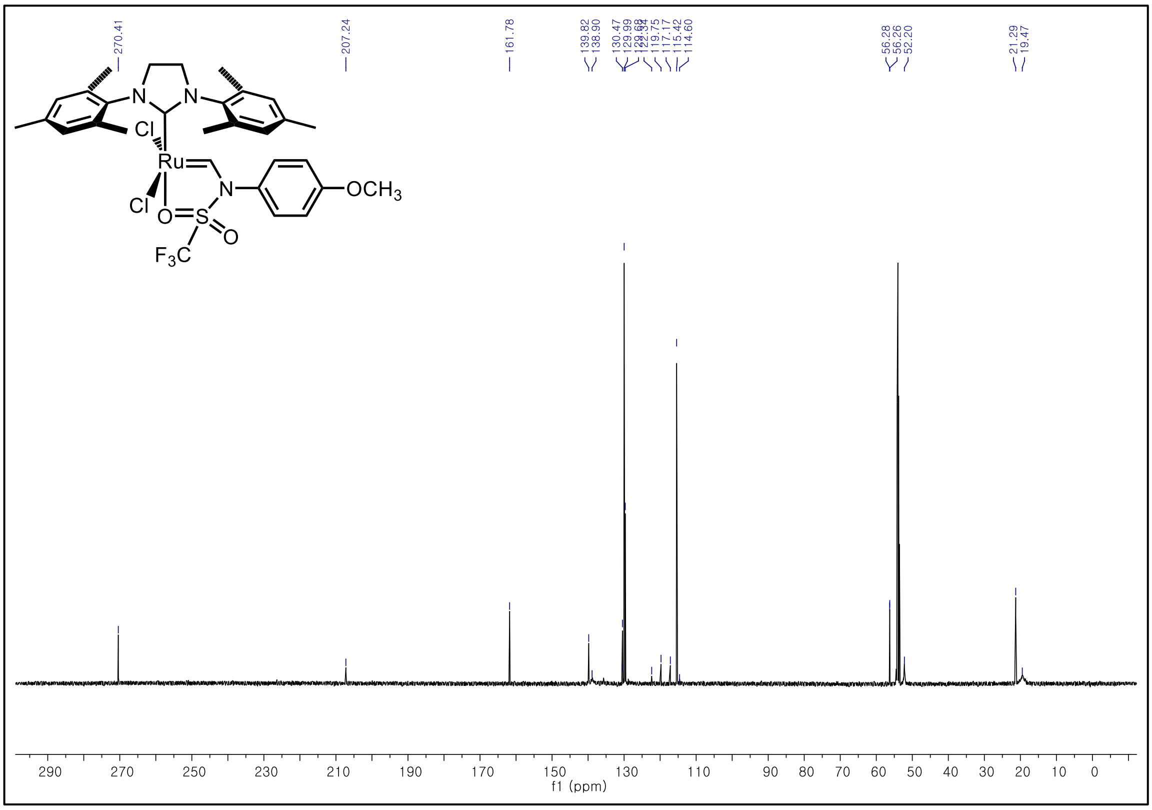
270.41
207.24
161.78
139.82
138.90
130.47
129.99
115.42
114.60
56.28
56.26
52.20
21.29
19.47
OCH3
Cl
Ru
N
O
S
F3C
f1 (ppm)

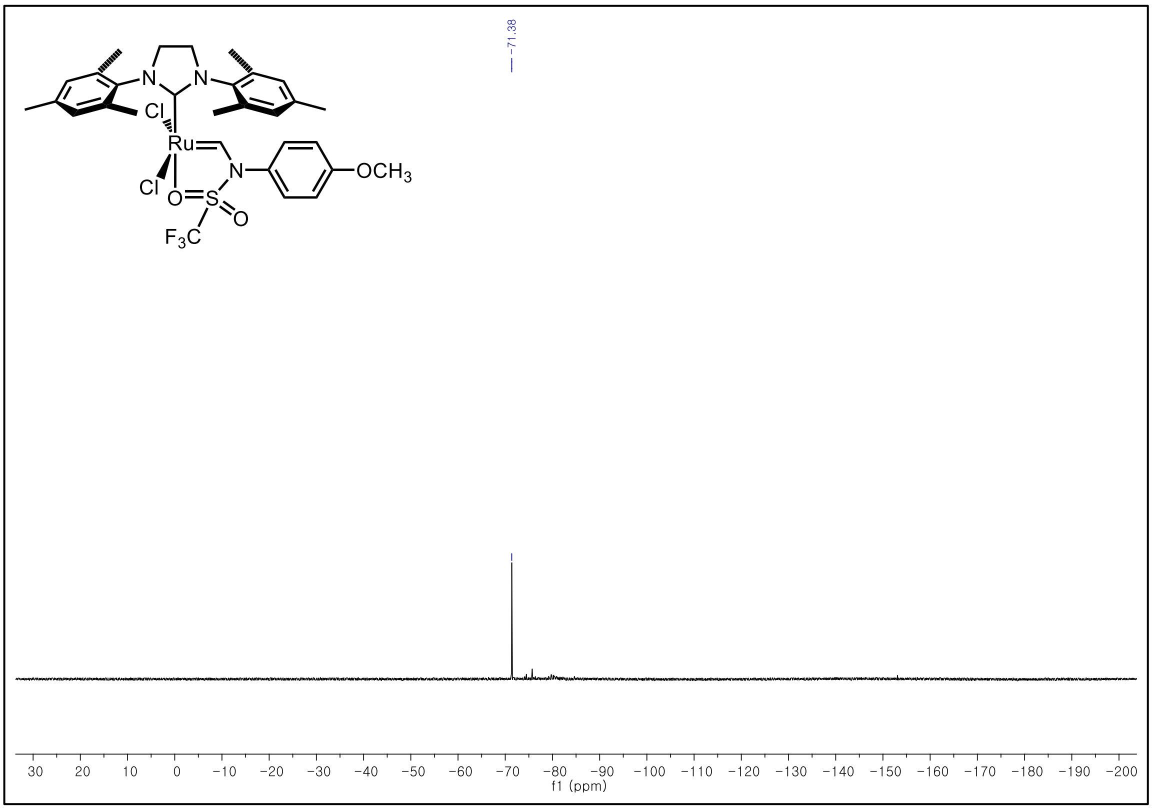

Supplementary Figure 51. ^{1}H NMR (**Ru-9**) (CD_2Cl_2, 500 MHz), ^{13}C NMR (**Ru-9**) (CD_2Cl_2, 75 MHz), and ^{19}F NMR (**Ru-9**) (CD_2Cl_2, 376 MHz)

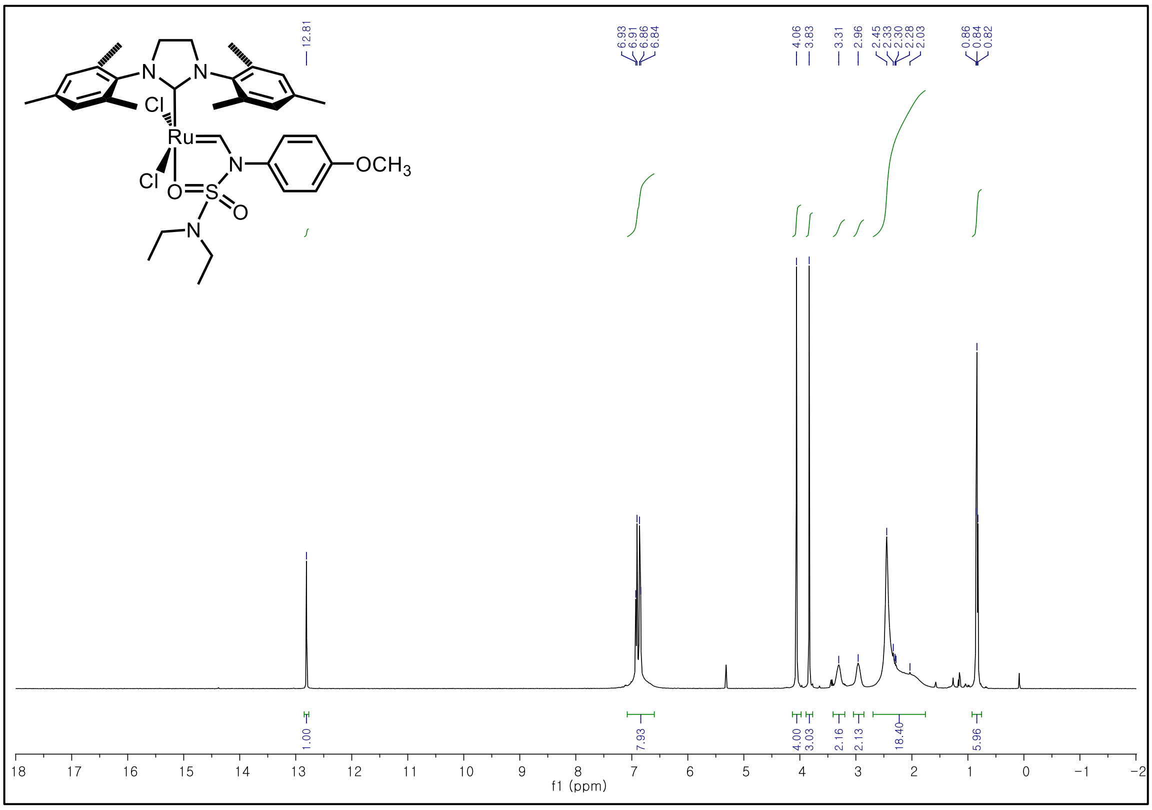

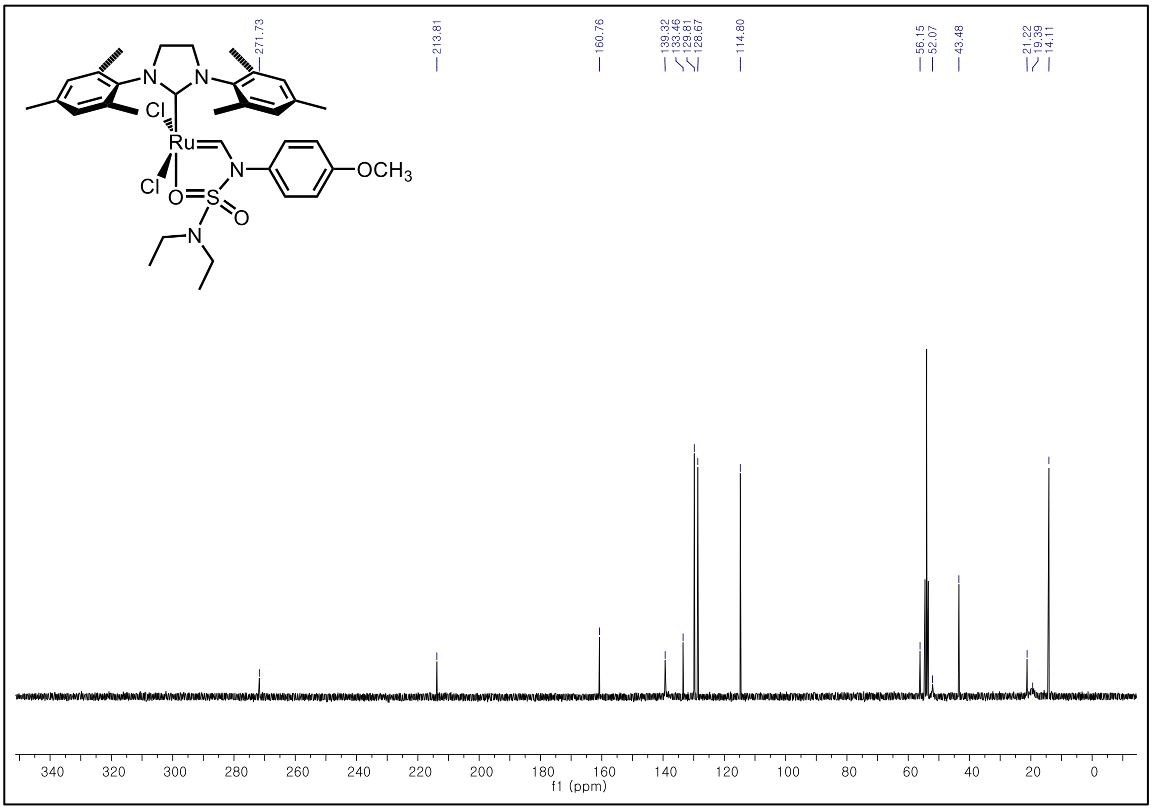

Supplementary Figure 52. ^{1}H NMR (**Ru-10**) (CD_2Cl_2, 400 MHz), and ^{13}C NMR (**Ru-10**) (CD_2Cl_2, 100 MHz)

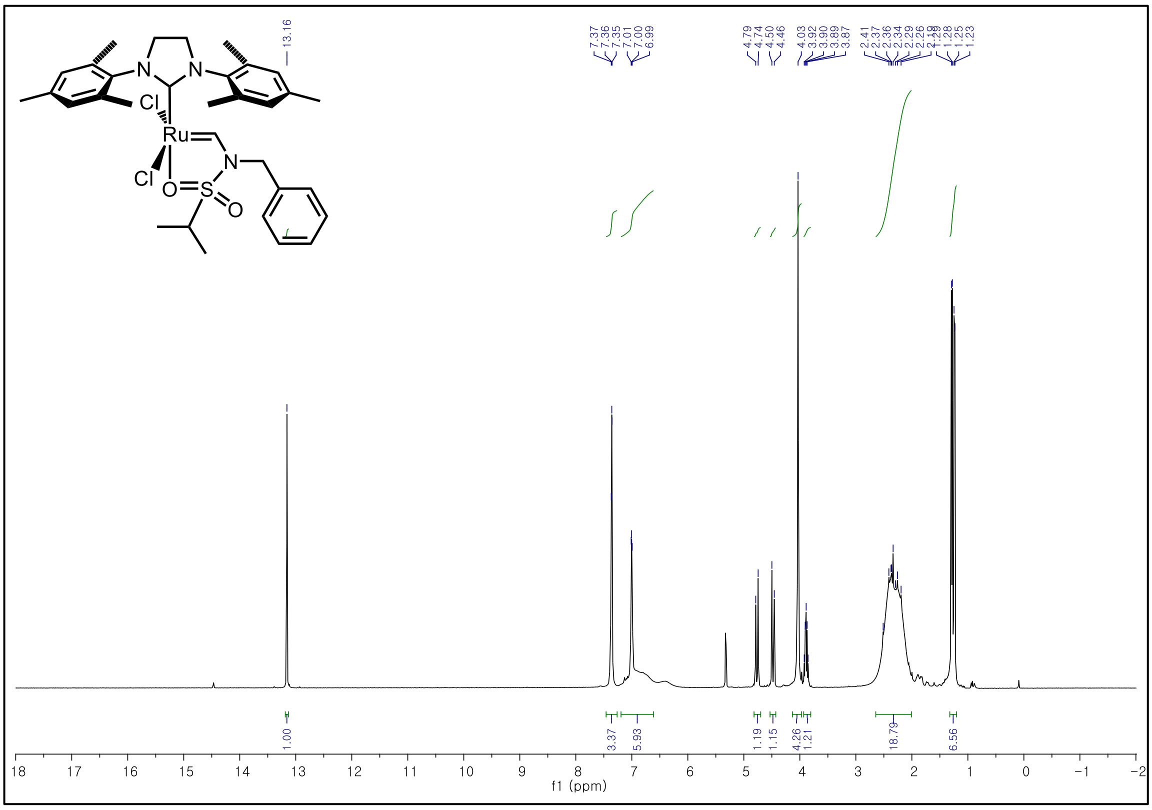

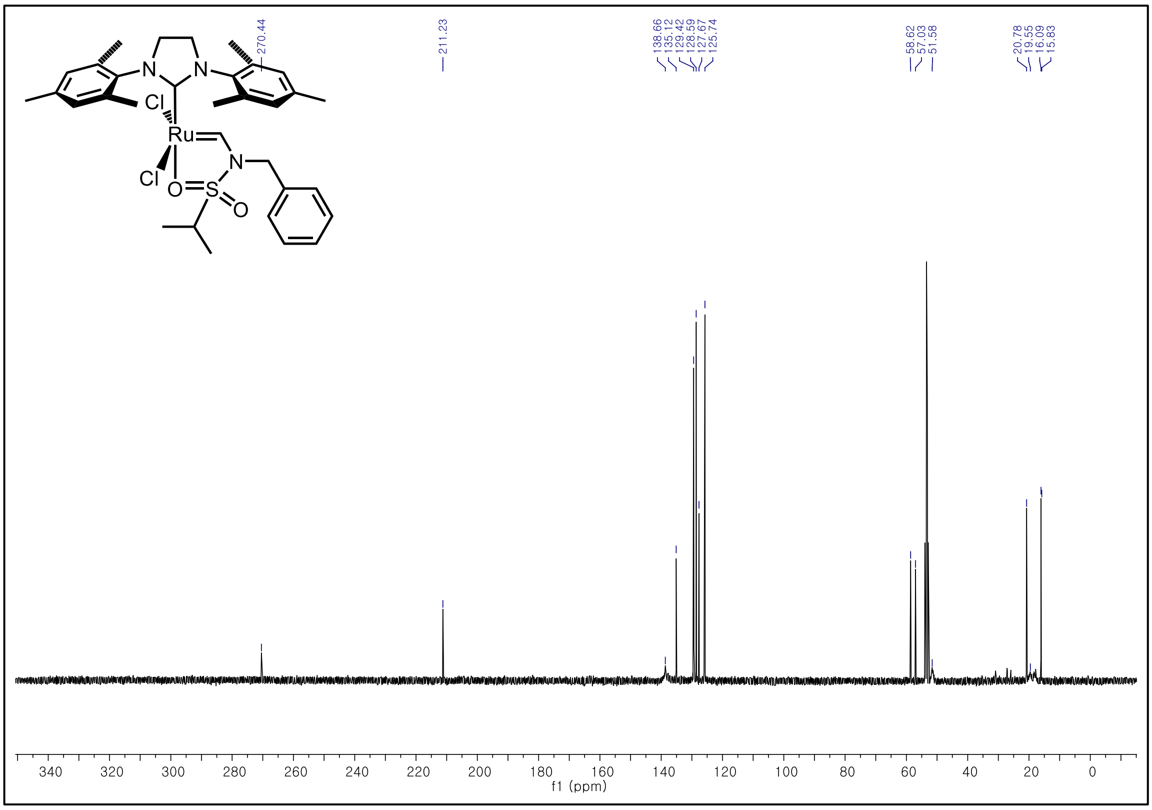

Supplementary Figure 53. ^{1}H NMR (**Ru-11**) (CD_2Cl_2, 400 MHz), and ^{13}C NMR (**Ru-11**) (CD_2Cl_2, 100 MHz)

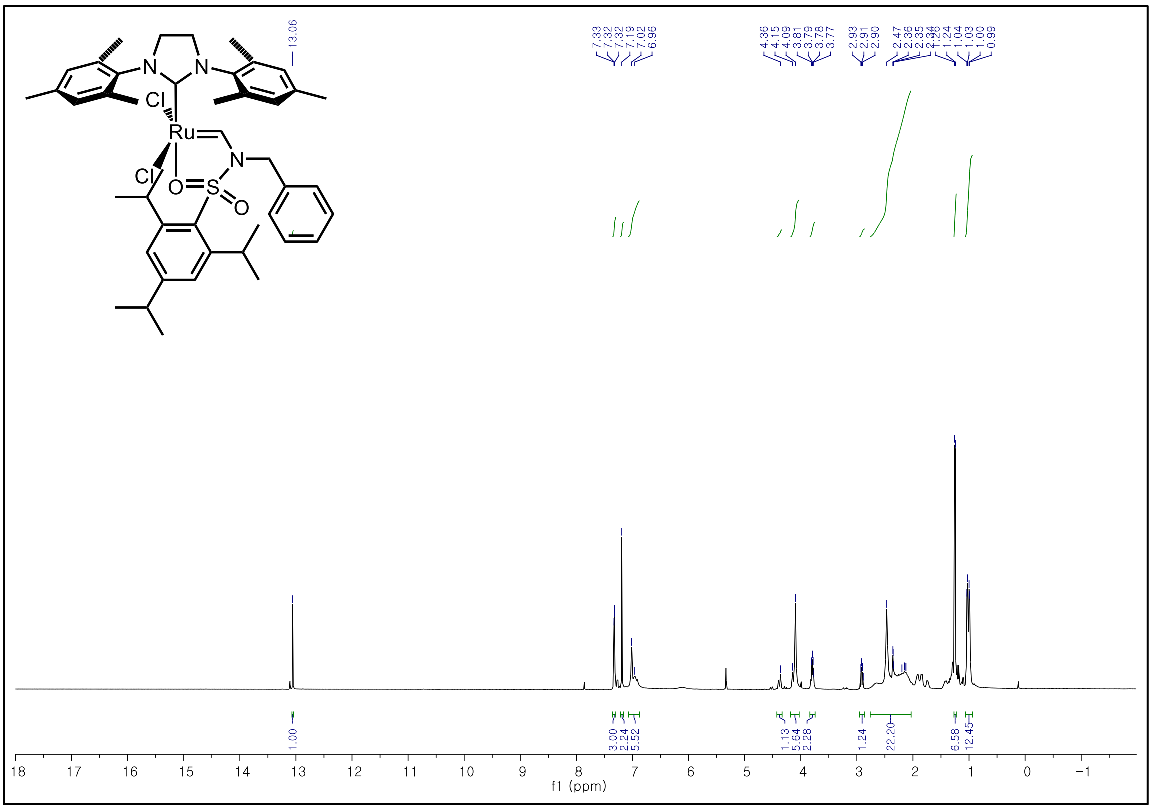

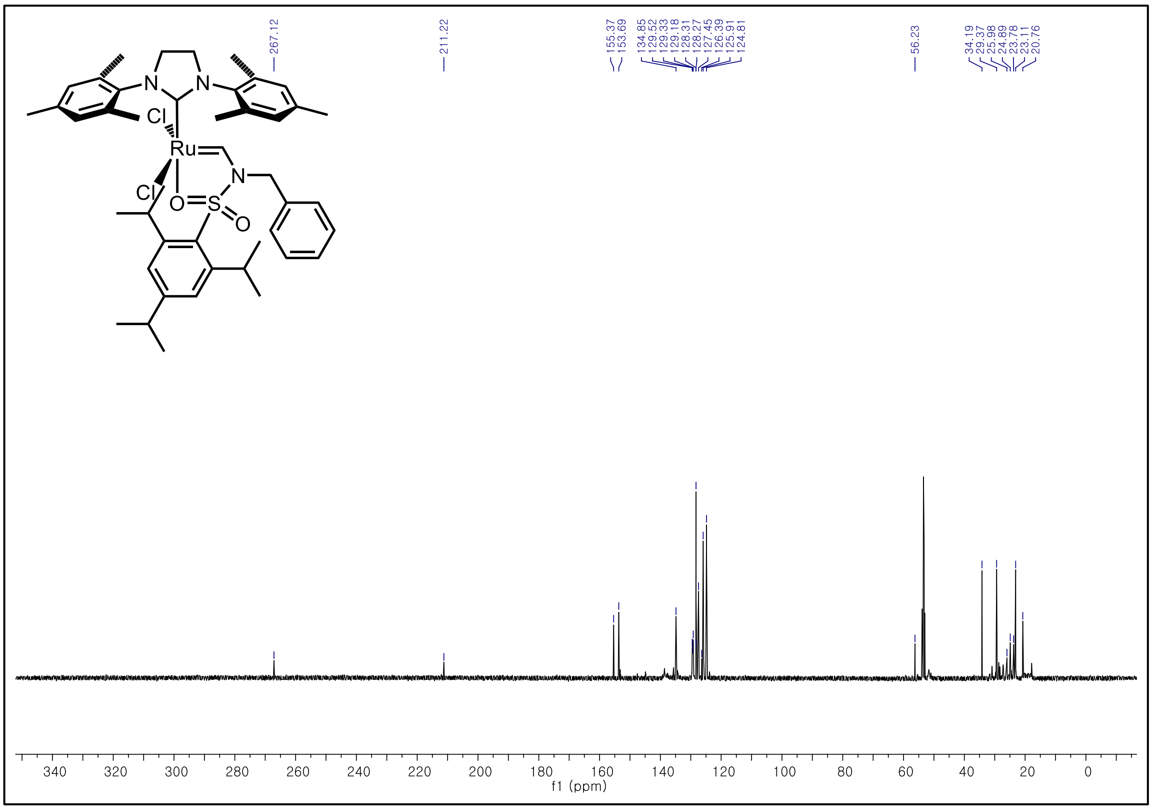

Supplementary Figure 54. ^{1}H NMR (**Ru-12**) (CD_2Cl_2, 500 MHz), and ^{13}C NMR (**Ru-12**) (CD_2Cl_2, 125 MHz)

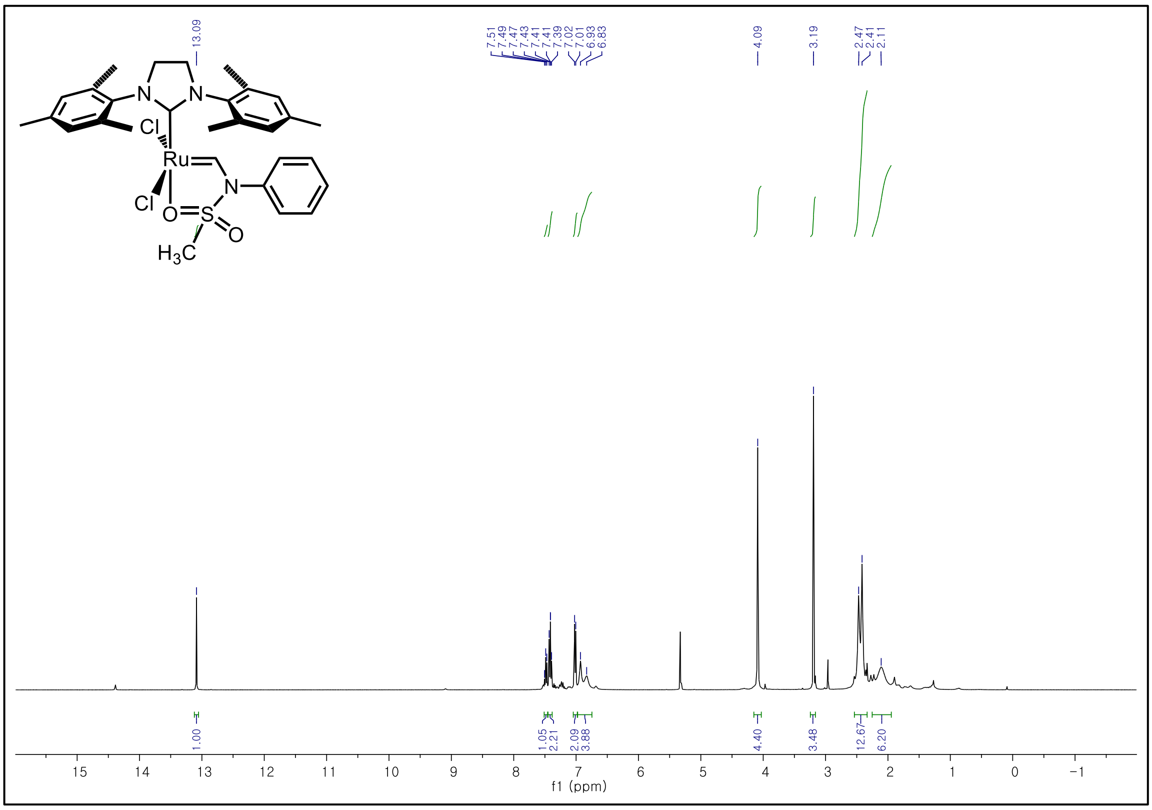

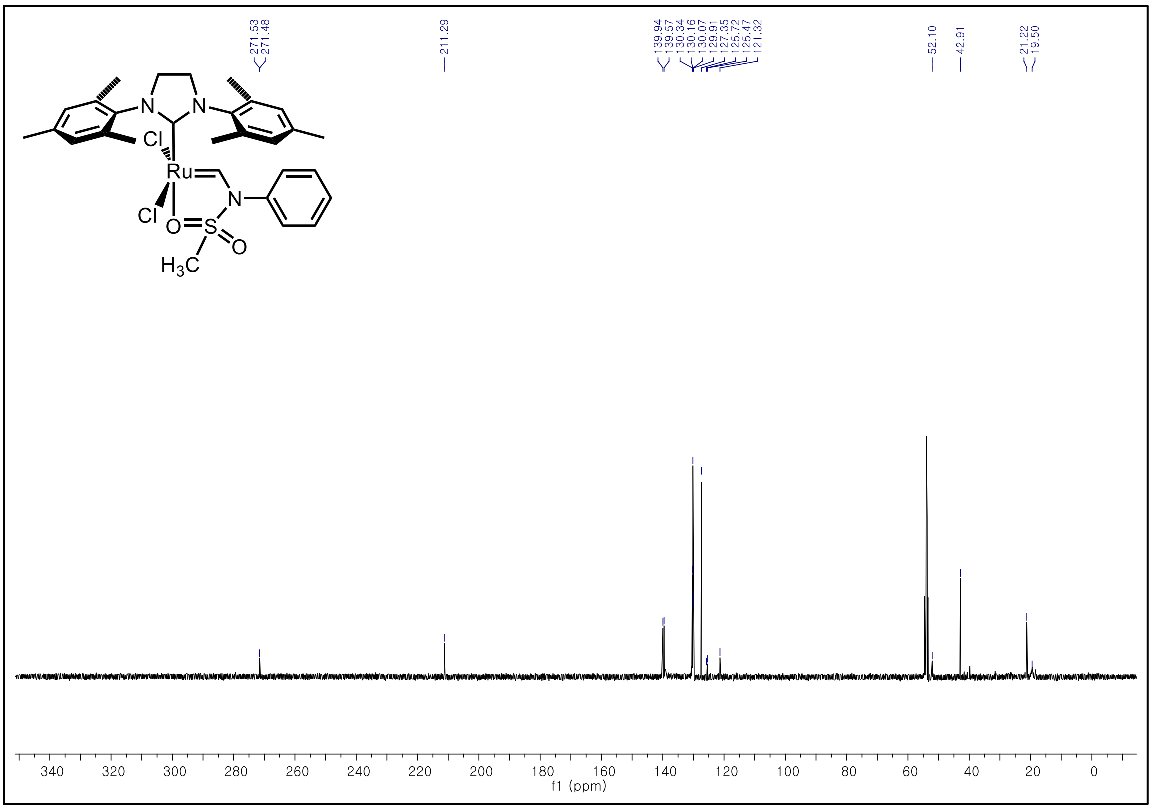

Supplementary Figure 55. ^{1}H NMR (**Ru-13**) (CD_2Cl_2, 500 MHz), and ^{13}C NMR (**Ru-13**) (CD_2Cl_2, 125 MHz)

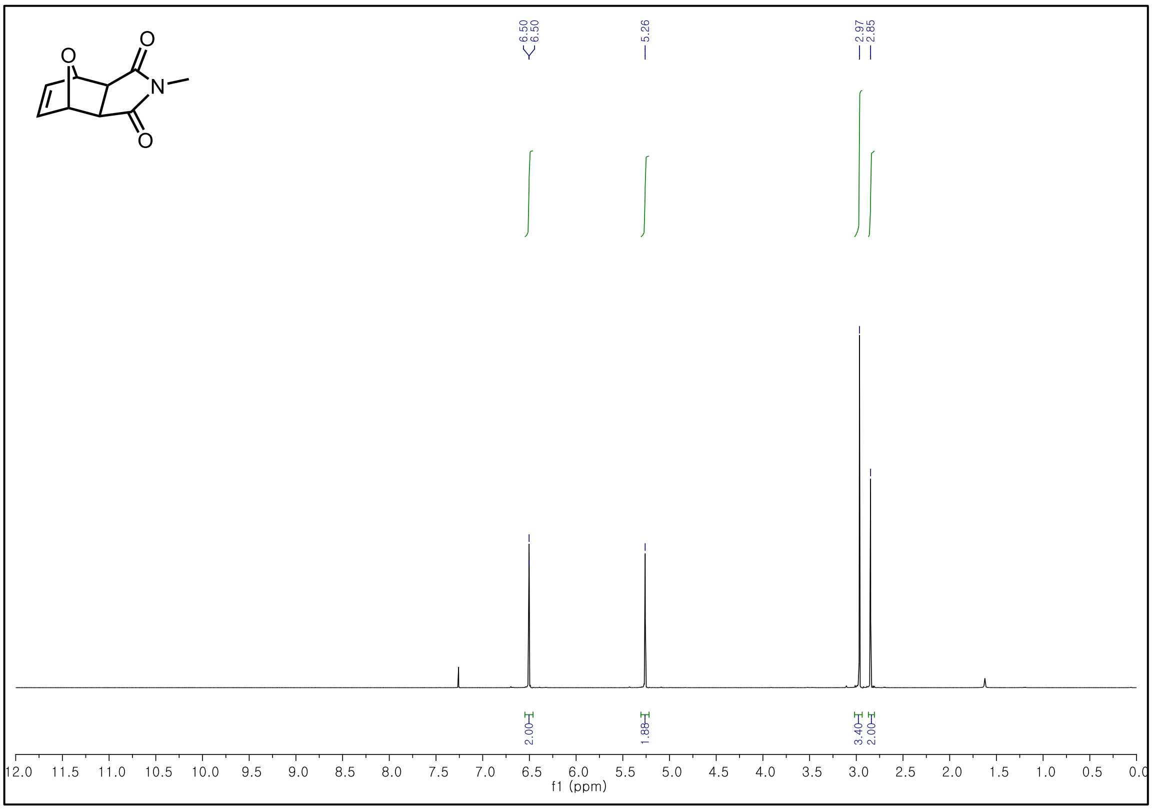

Supplementary Figure 56. ^{1}H NMR (**5**) ($CDCl_3$, 500 MHz)

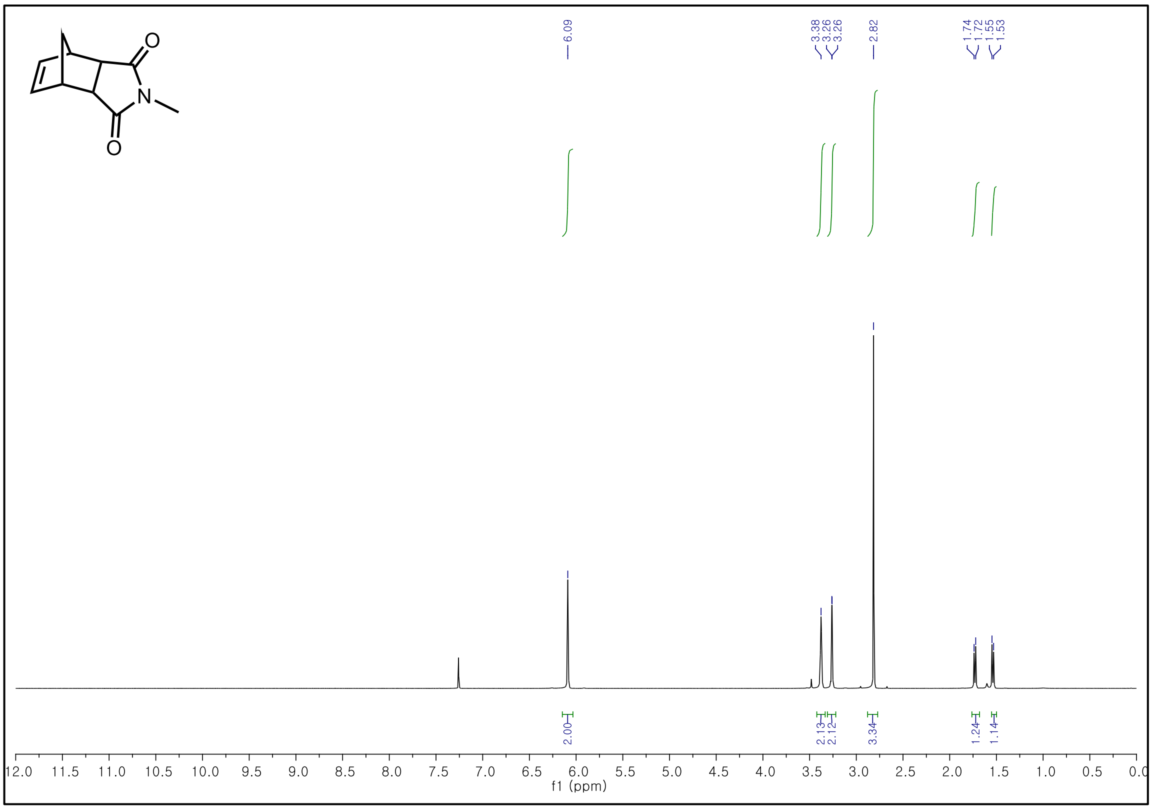

Supplementary Figure 57. ^{1}H NMR (**6**) ($CDCl_3$, 500 MHz)

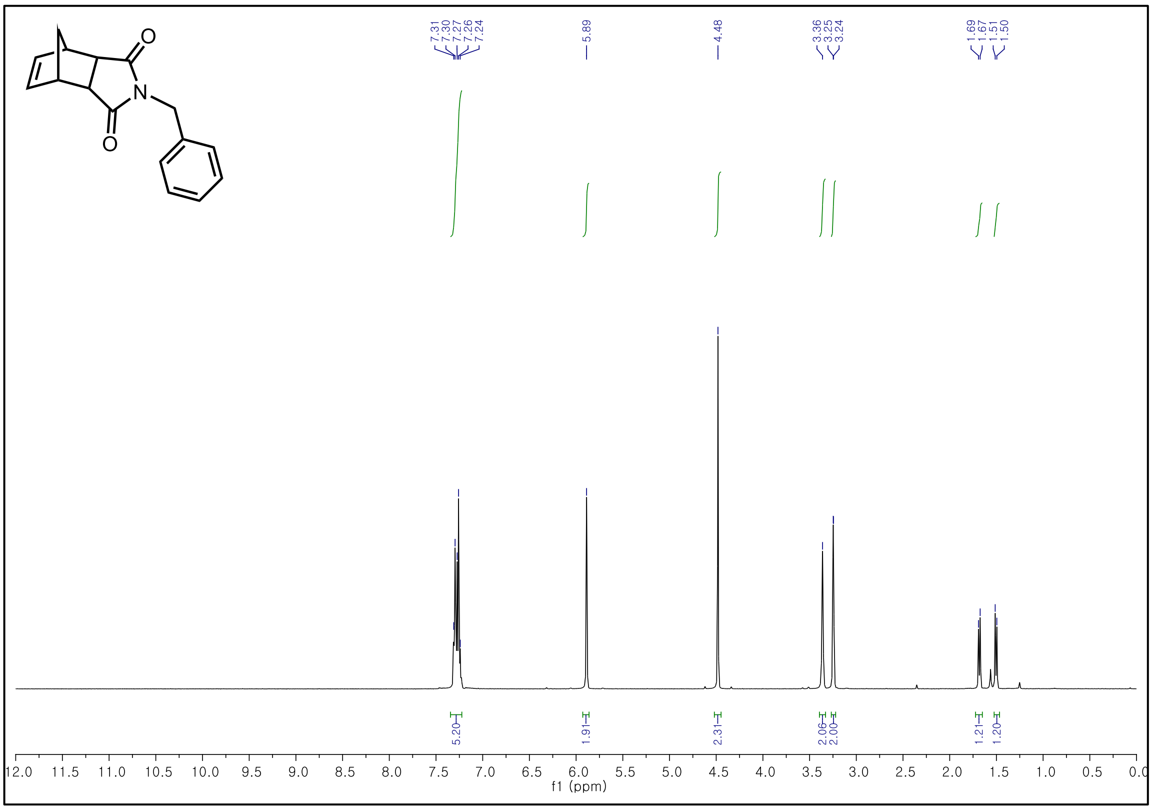

Supplementary Figure 58. ^{1}H NMR (**7**) ($CDCl_3$, 500 MHz)

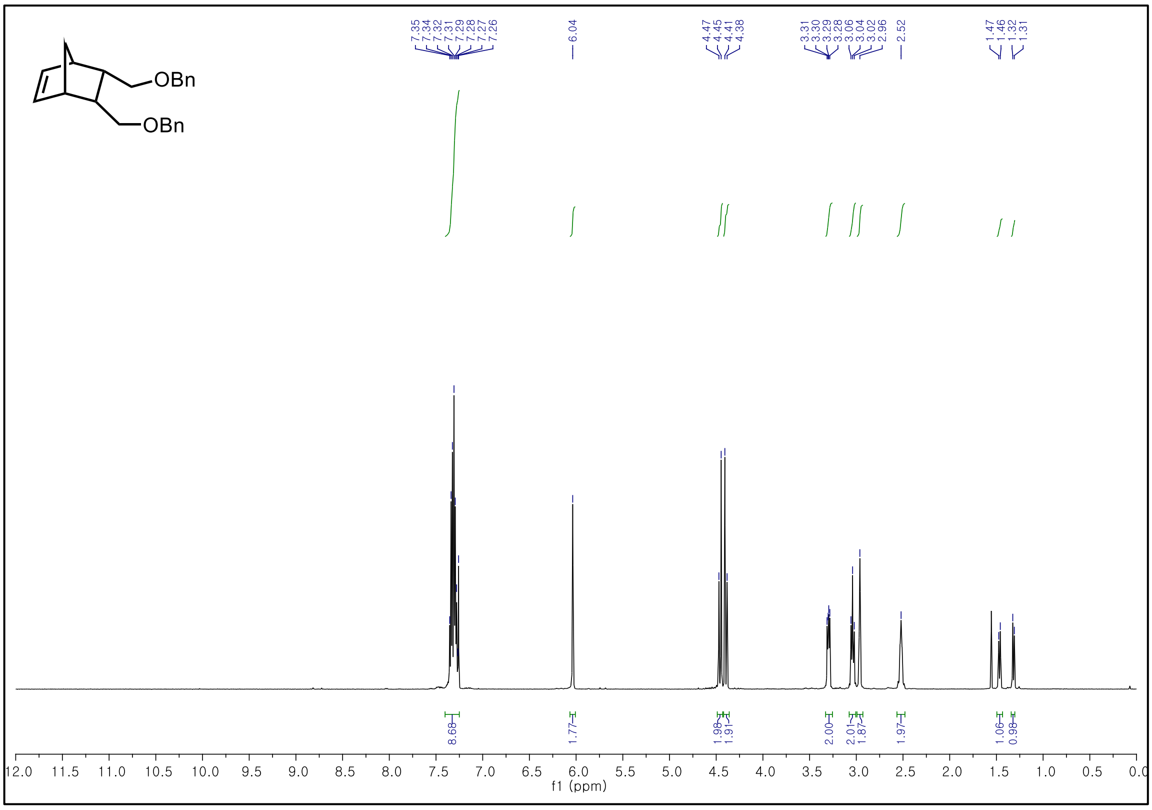

Supplementary Figure 59. ^{1}H NMR (**8**) ($CDCl_3$, 500 MHz)

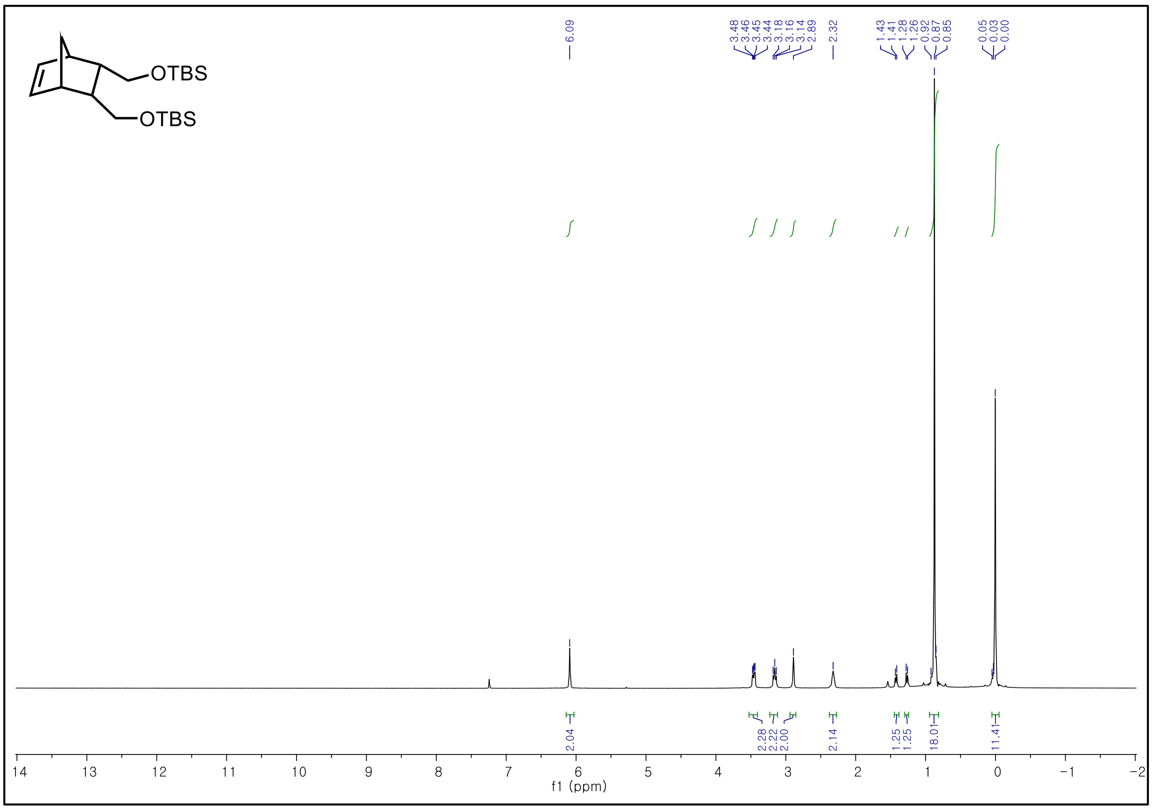

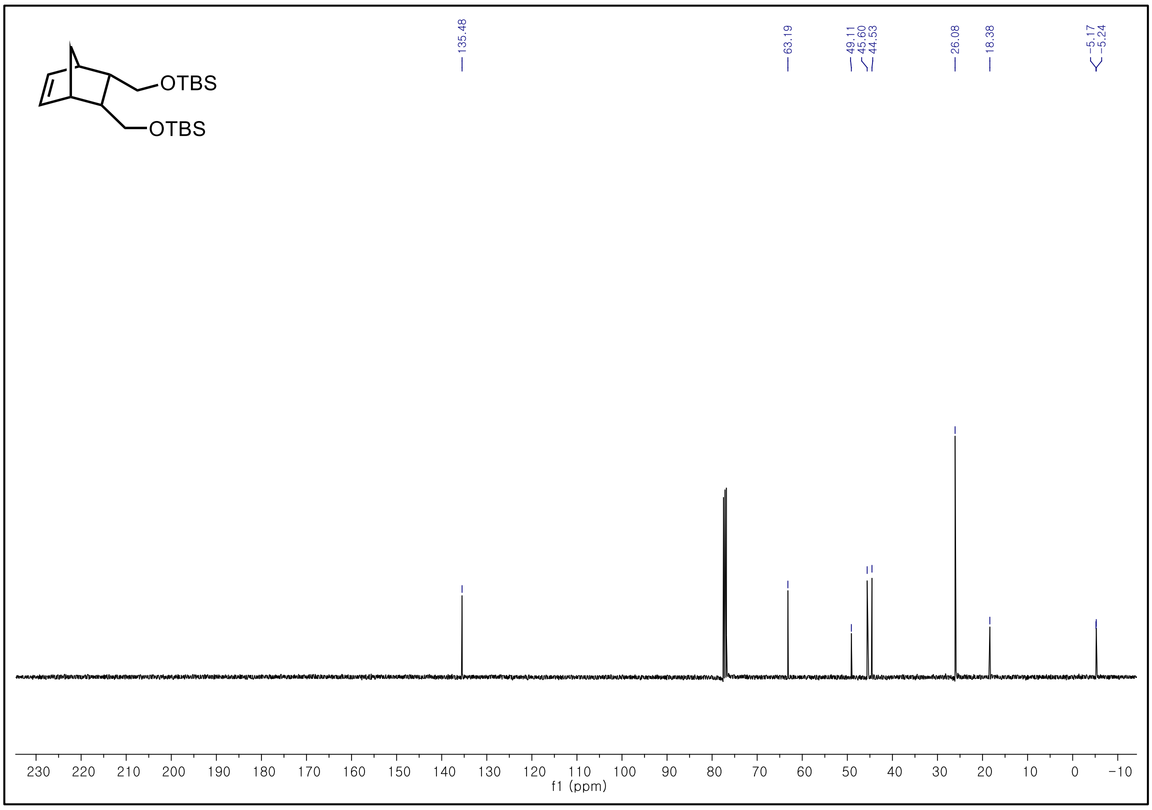

Supplementary Figure 60. ^{1}H NMR (**9**) ($CDCl_3$, 400 MHz) and ^{13}C NMR (**9**) ($CDCl_3$, 100 MHz)

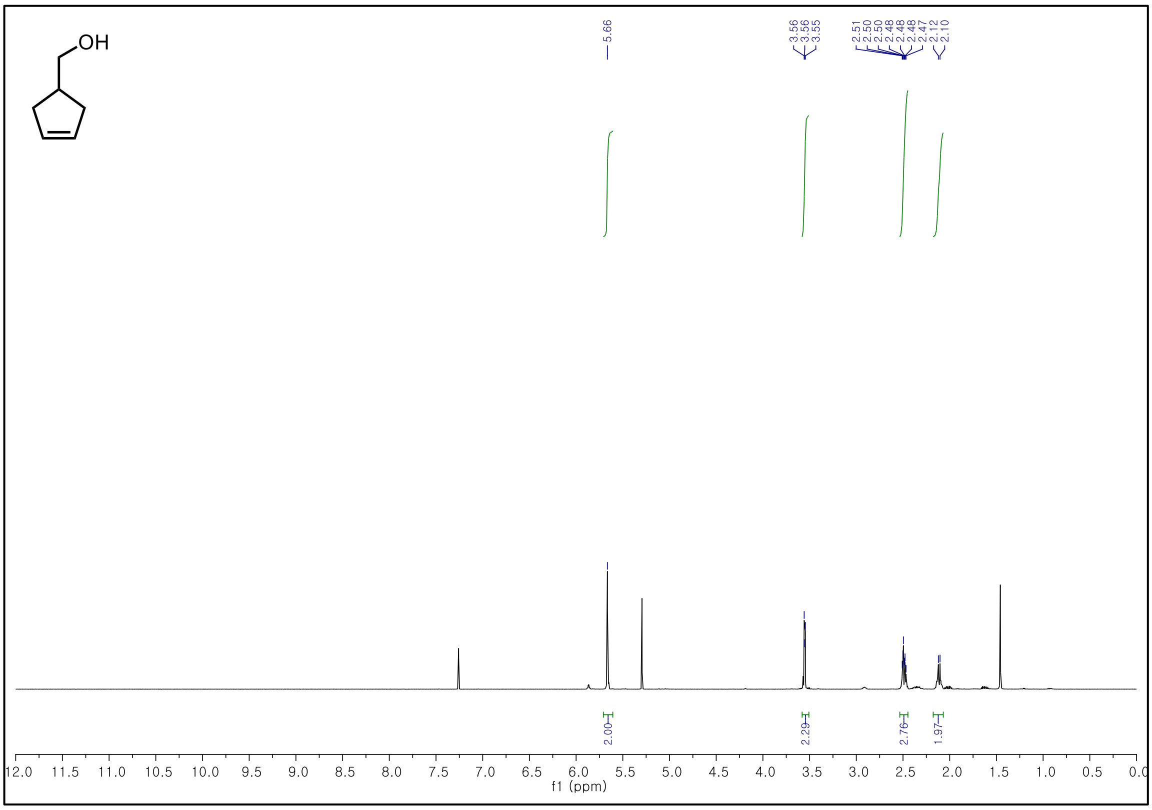

Supplementary Figure 61. ^{1}H NMR (**11**) ($CDCl_3$, 500 MHz)

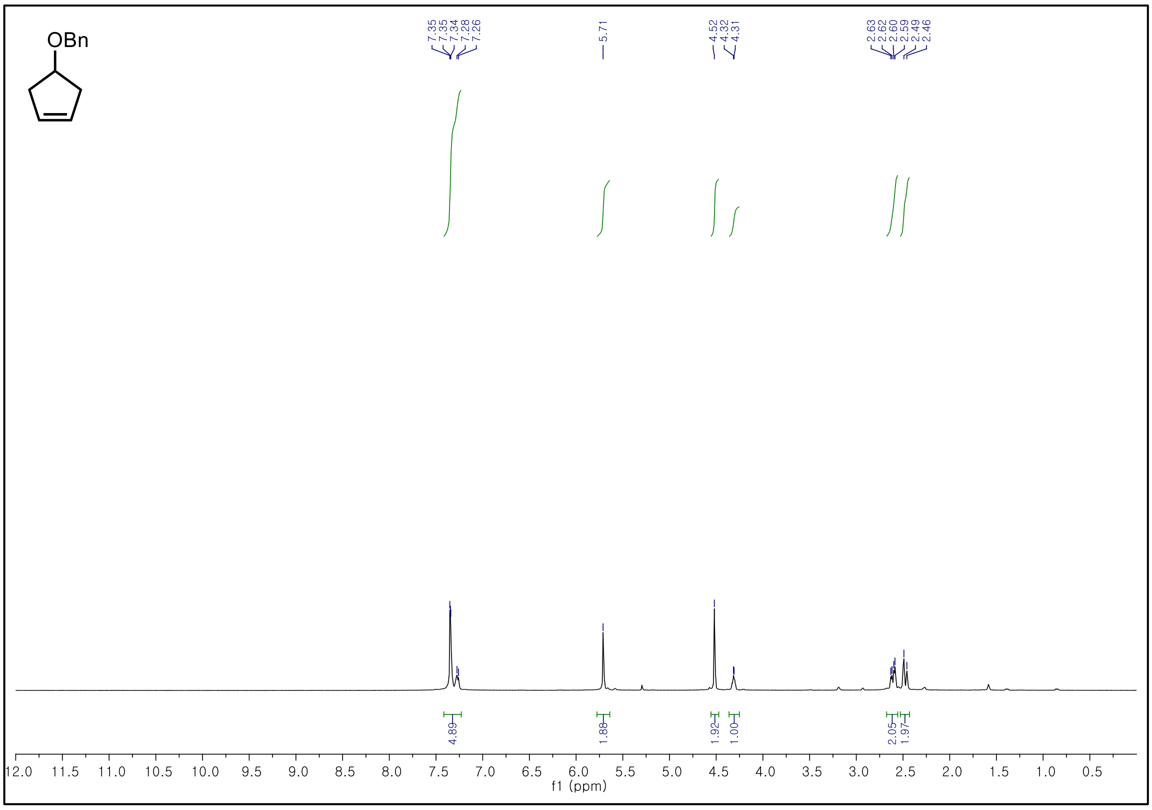

Supplementary Figure 62. ^{1}H NMR (**12**) ($CDCl_3$, 300 MHz)

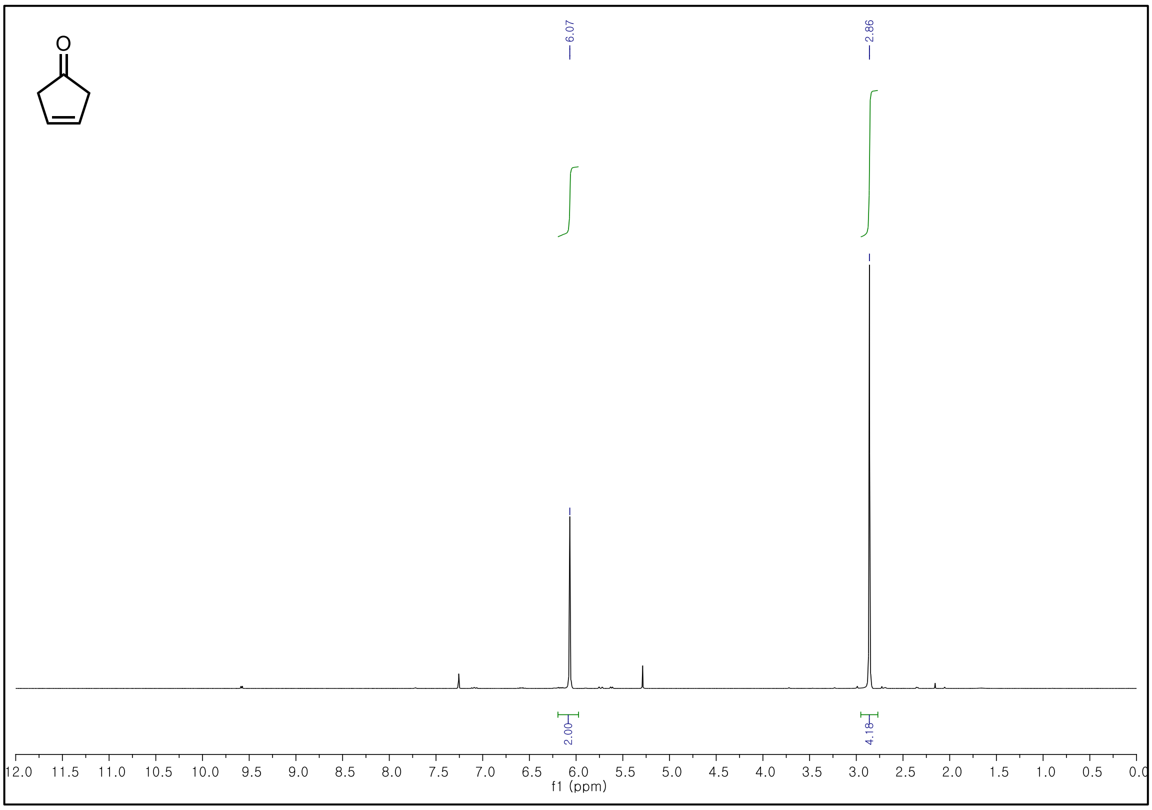

Supplementary Figure 63. ^{1}H NMR (**13**) ($CDCl_3$, 500 MHz)

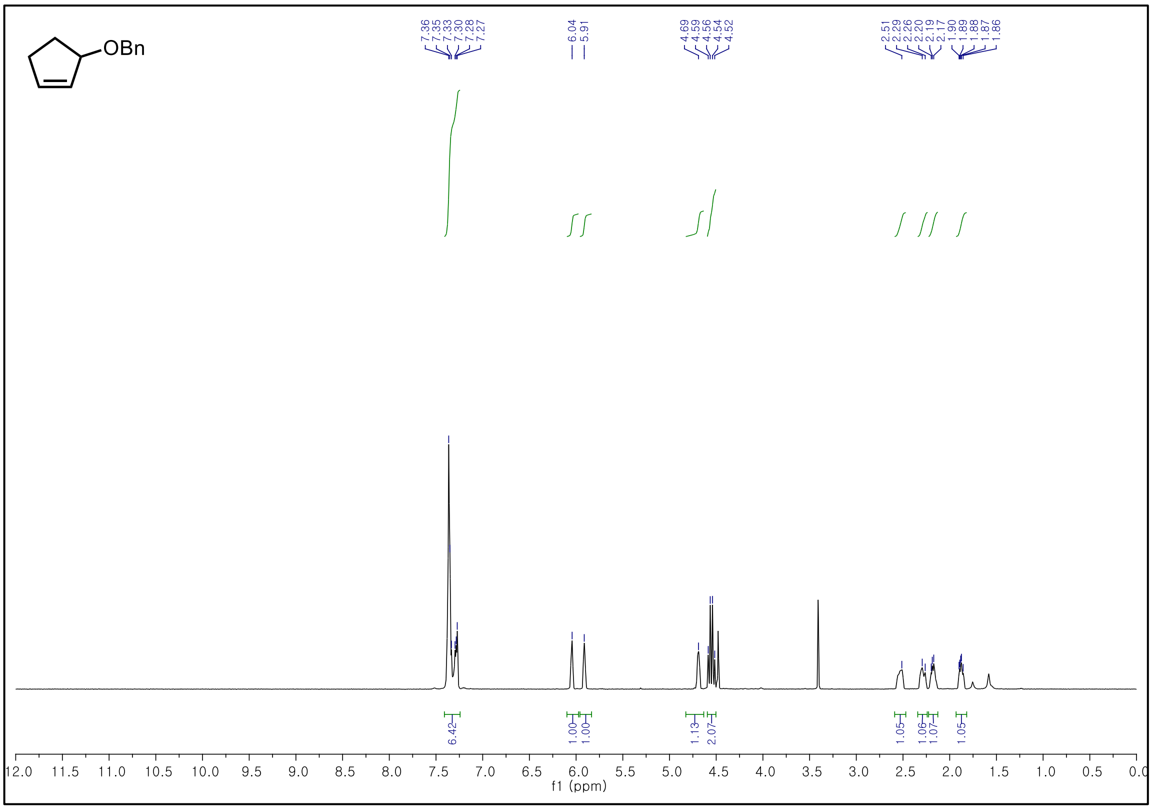

Supplementary Figure 64. ^{1}H NMR (**14**) ($CDCl_3$, 500 MHz)

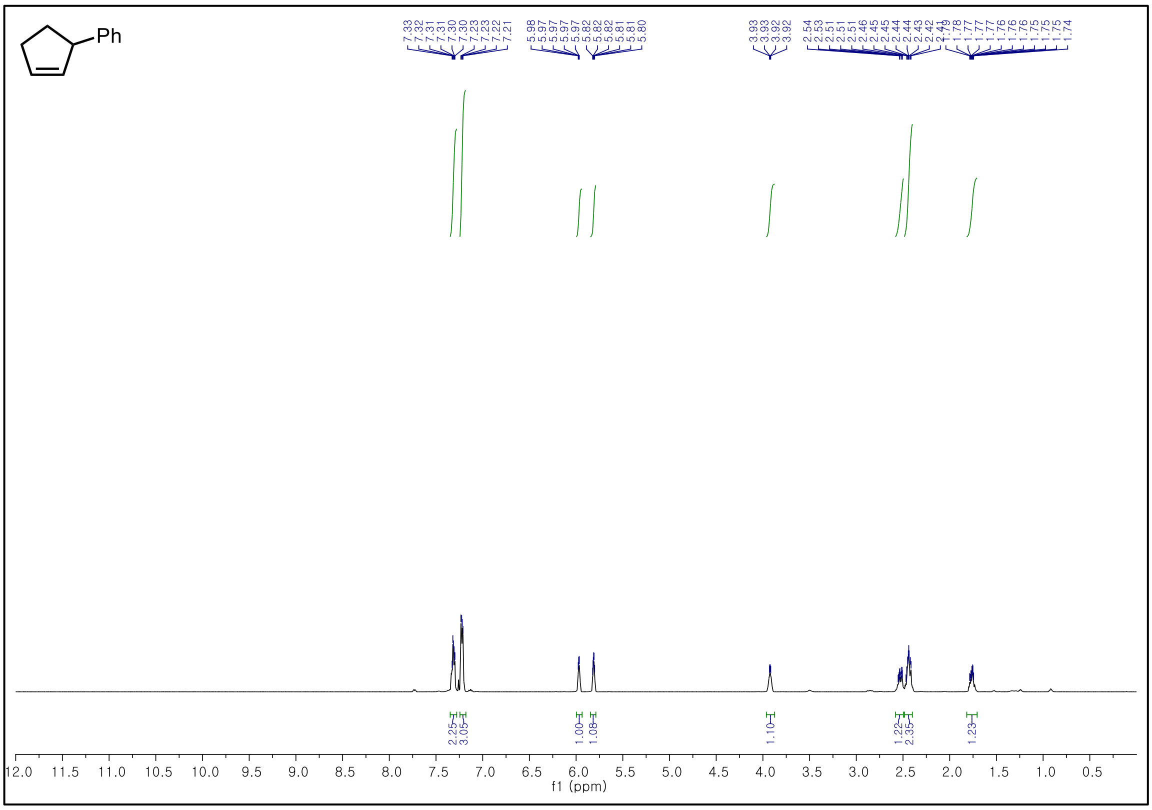

Supplementary Figure 65. ^{1}H NMR (**15**) ($CDCl_3$, 500 MHz)

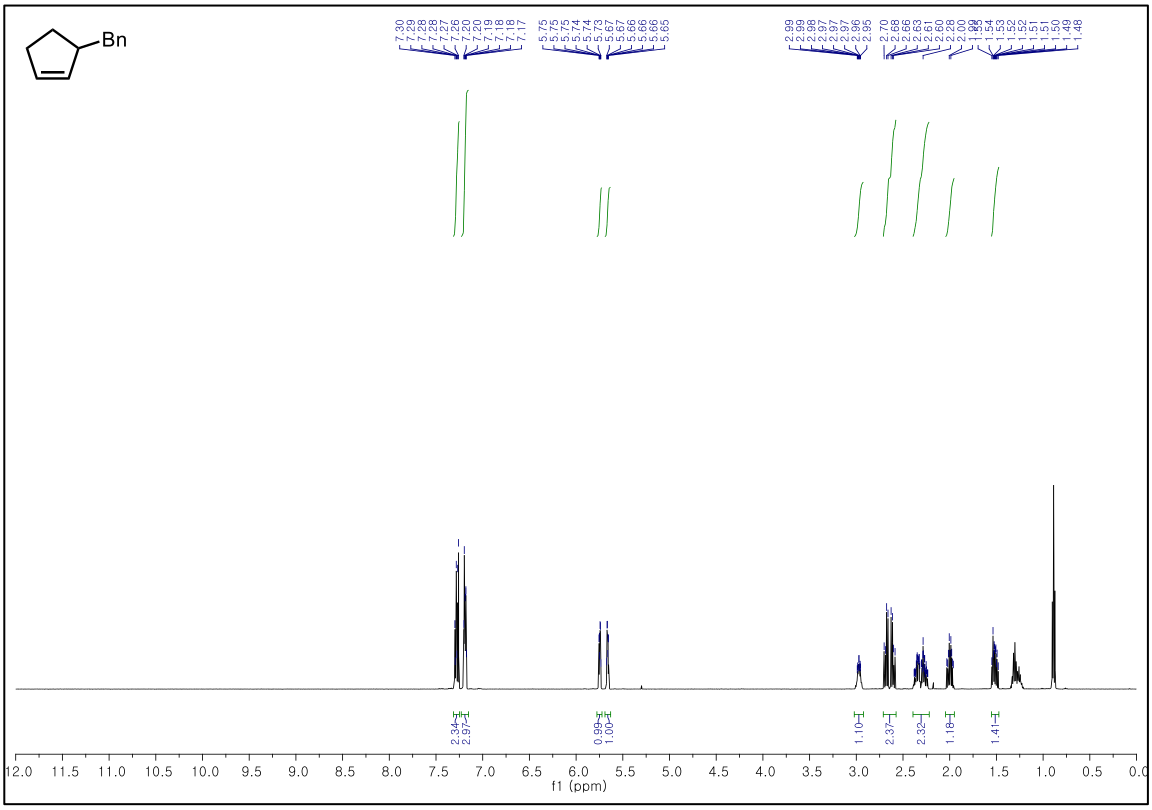

Supplementary Figure 66. ^{1}H NMR (**16**) ($CDCl_3$, 500 MHz)

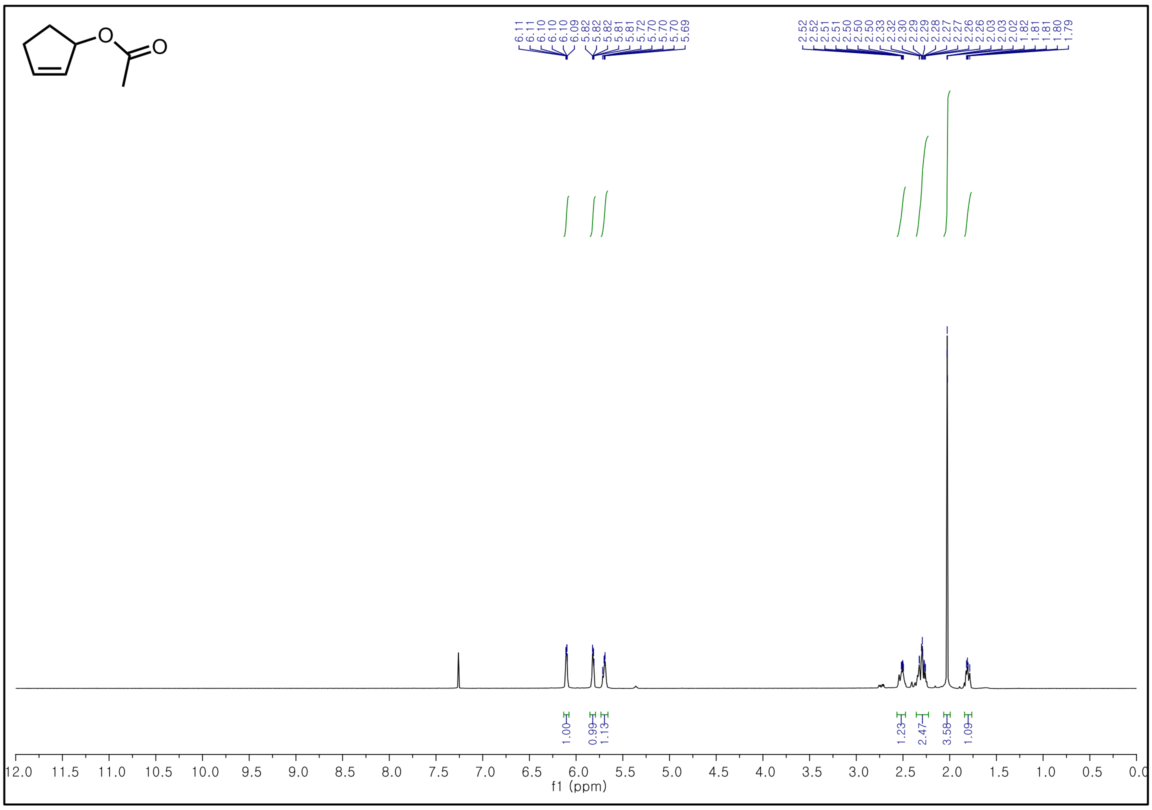

Supplementary Figure 67. ^{1}H NMR (**17**) ($CDCl_3$, 500 MHz)

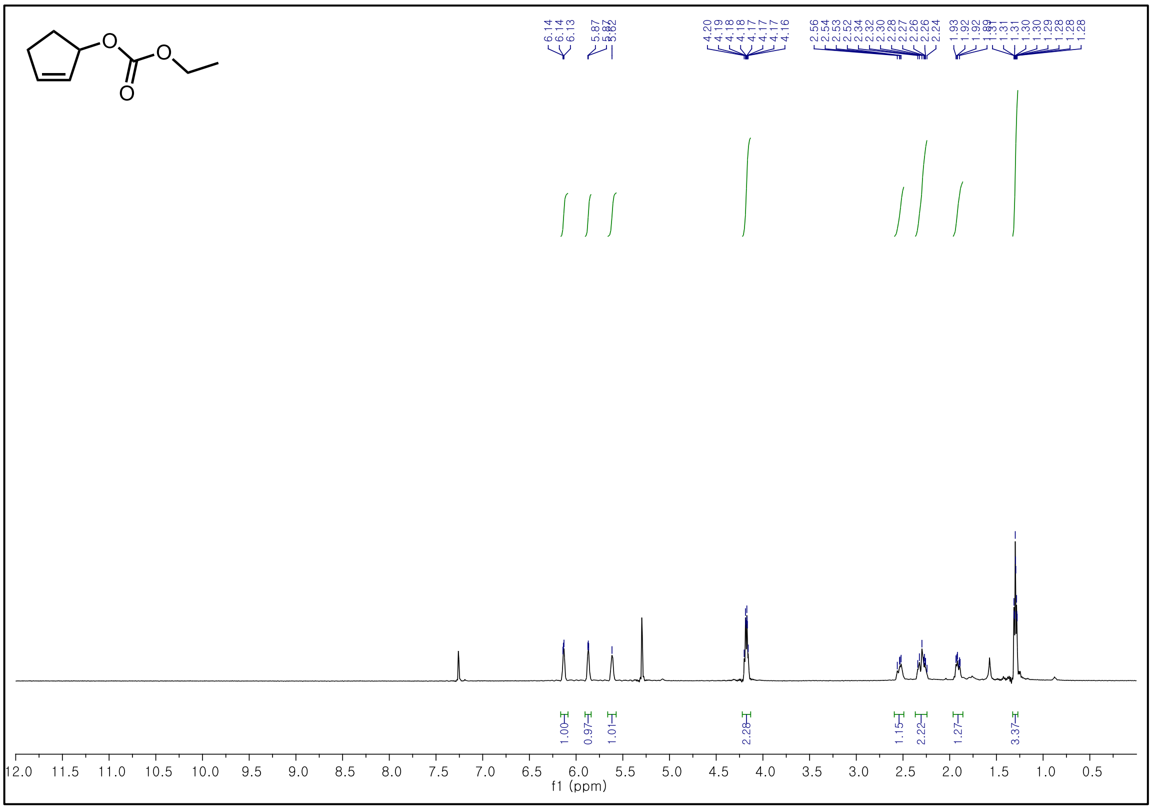

Supplementary Figure 68. ^{1}H NMR (**18**) ($CDCl_3$, 400 MHz)

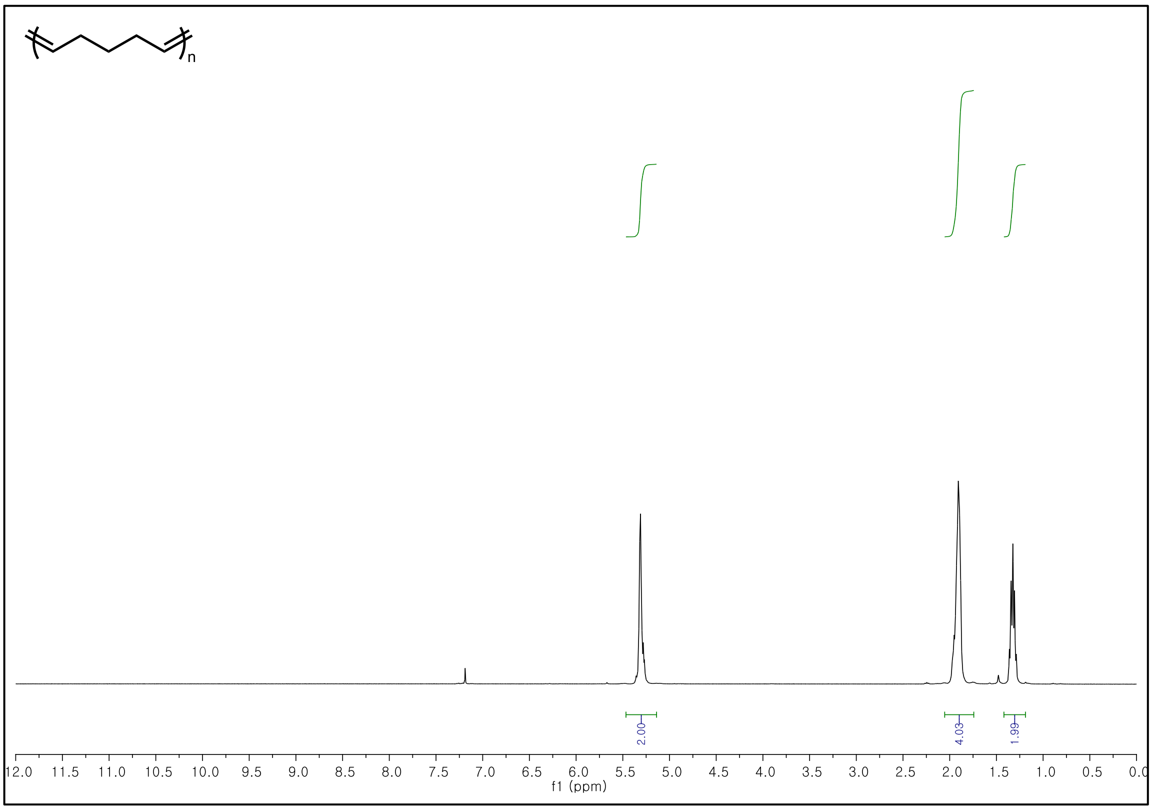

Supplementary Figure 69. ^{1}H NMR (**poly-10**) ($CDCl_3$, 400 MHz)

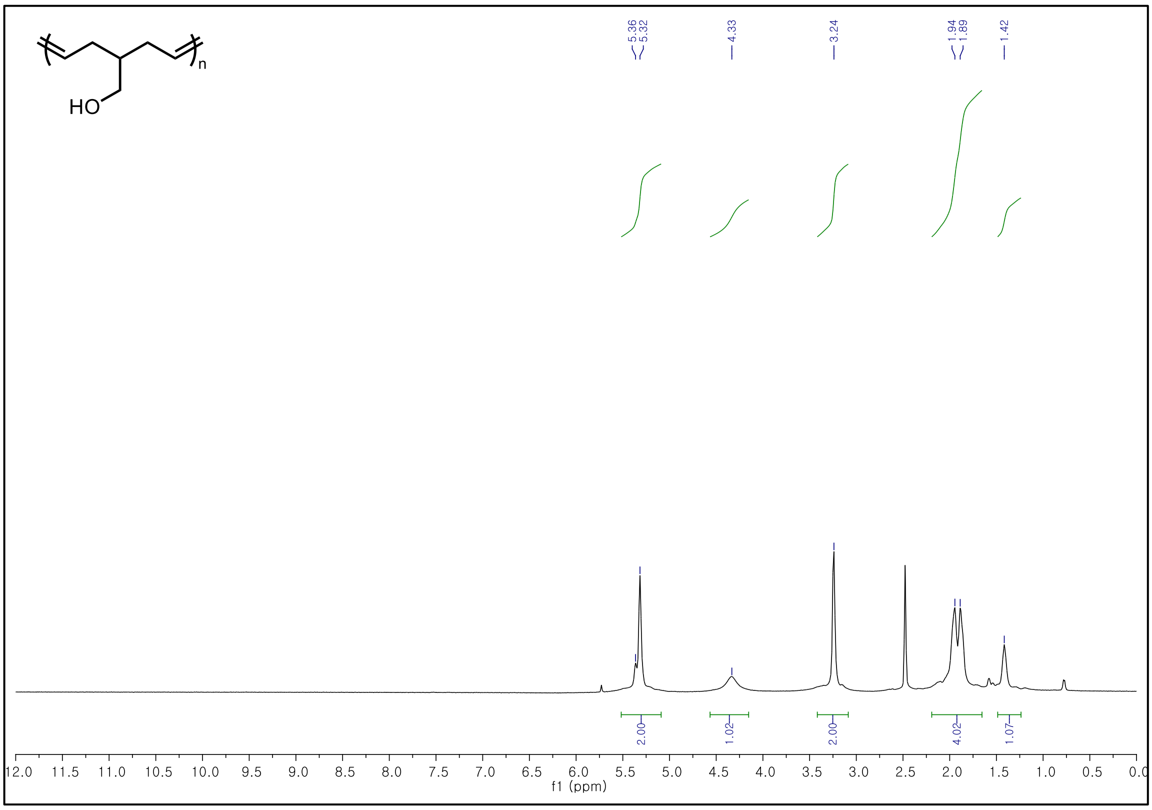

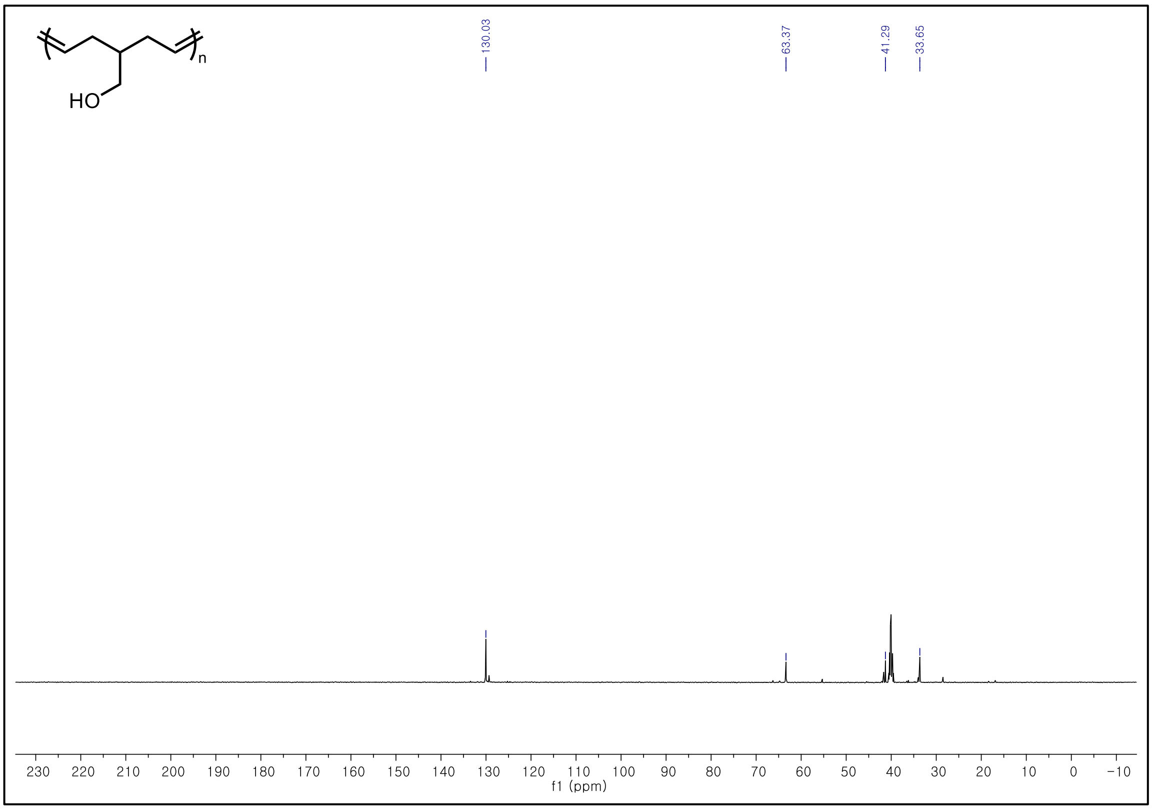

Supplementary Figure 70. ^{1}H NMR (**poly-11**) (DMSO-d_6, 500 MHz) and ^{13}C NMR (**poly-11**) (DMSO-d_6, 125 MHz)

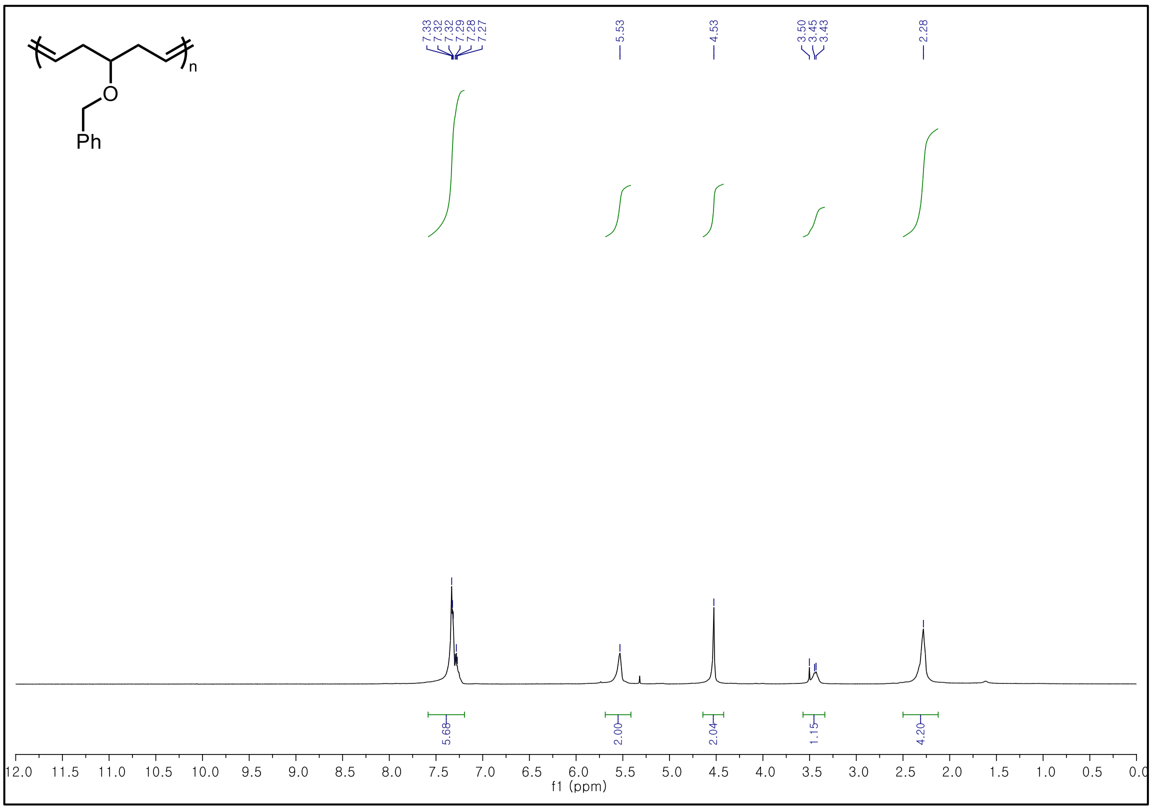

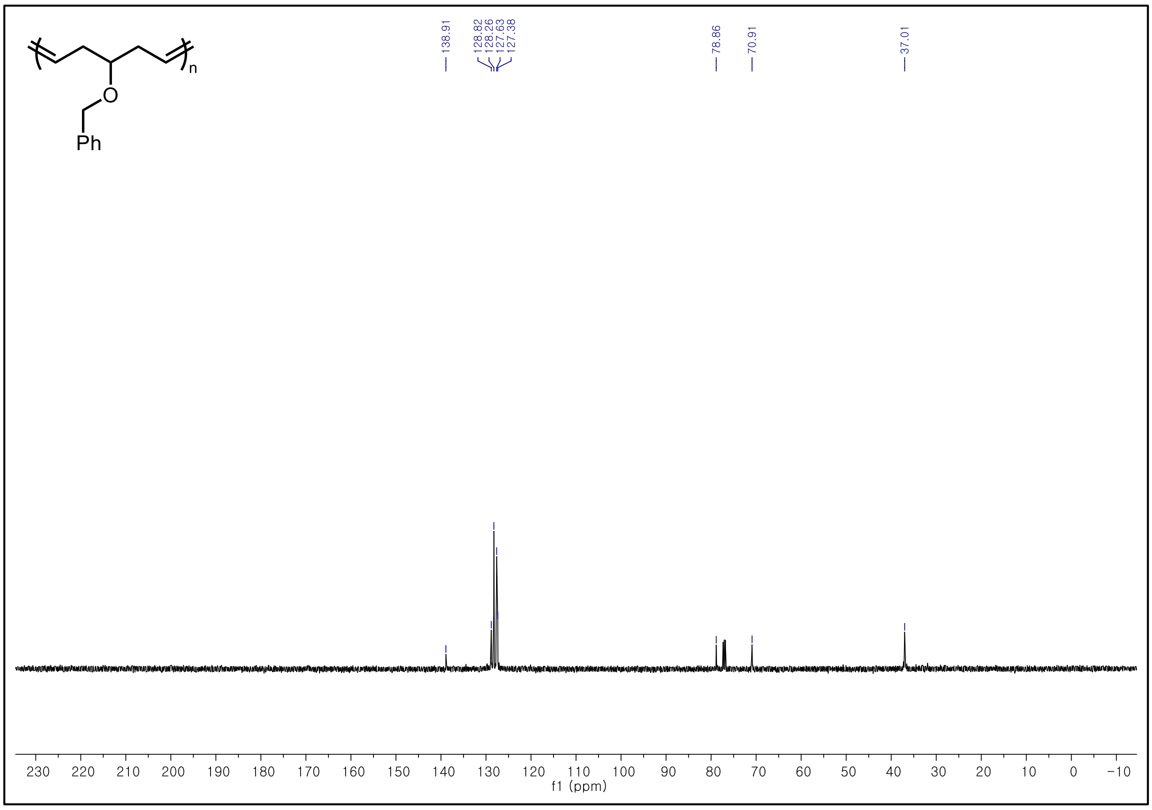

Supplementary Figure 71. ^{1}H NMR (**poly-12**) ($CDCl_3$, 300 MHz) and ^{13}C NMR (**poly-12**) ($CDCl_3$, 125 MHz)

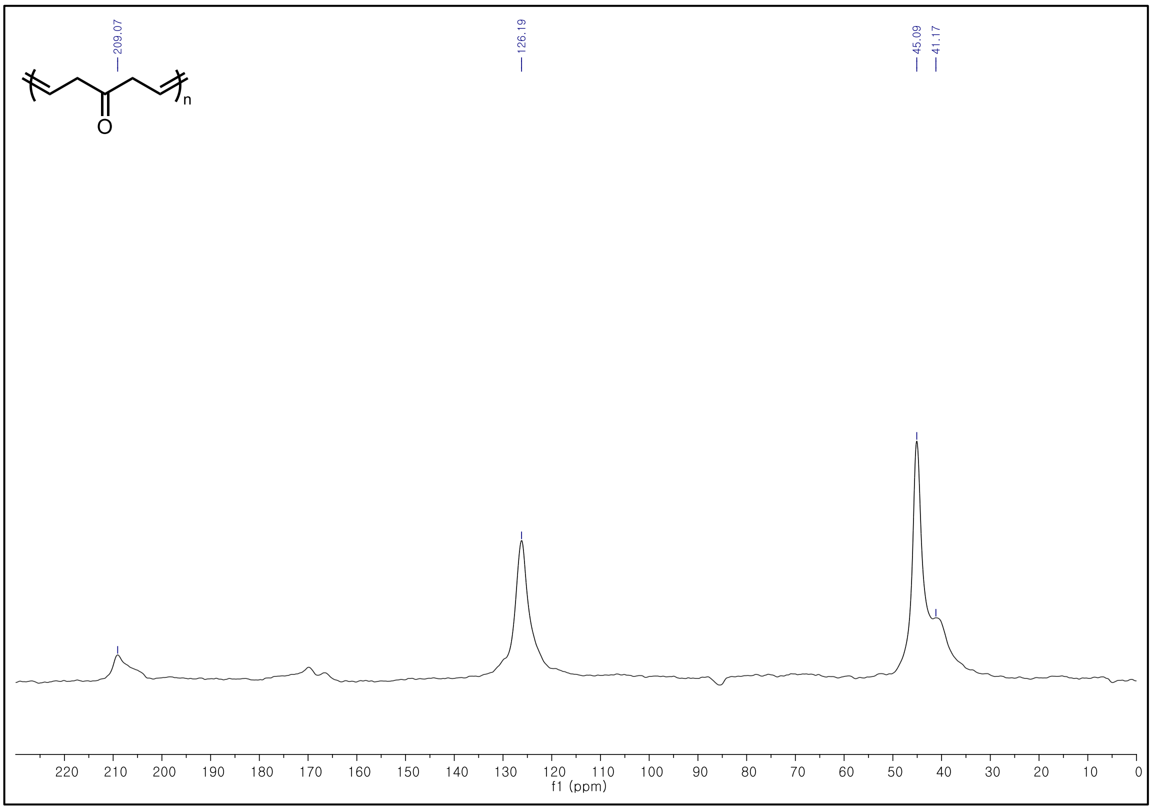

Supplementary Figure 72. ^{13}C NMR (**poly-13**) (CPTOSS 5 kHz, 125 MHz)

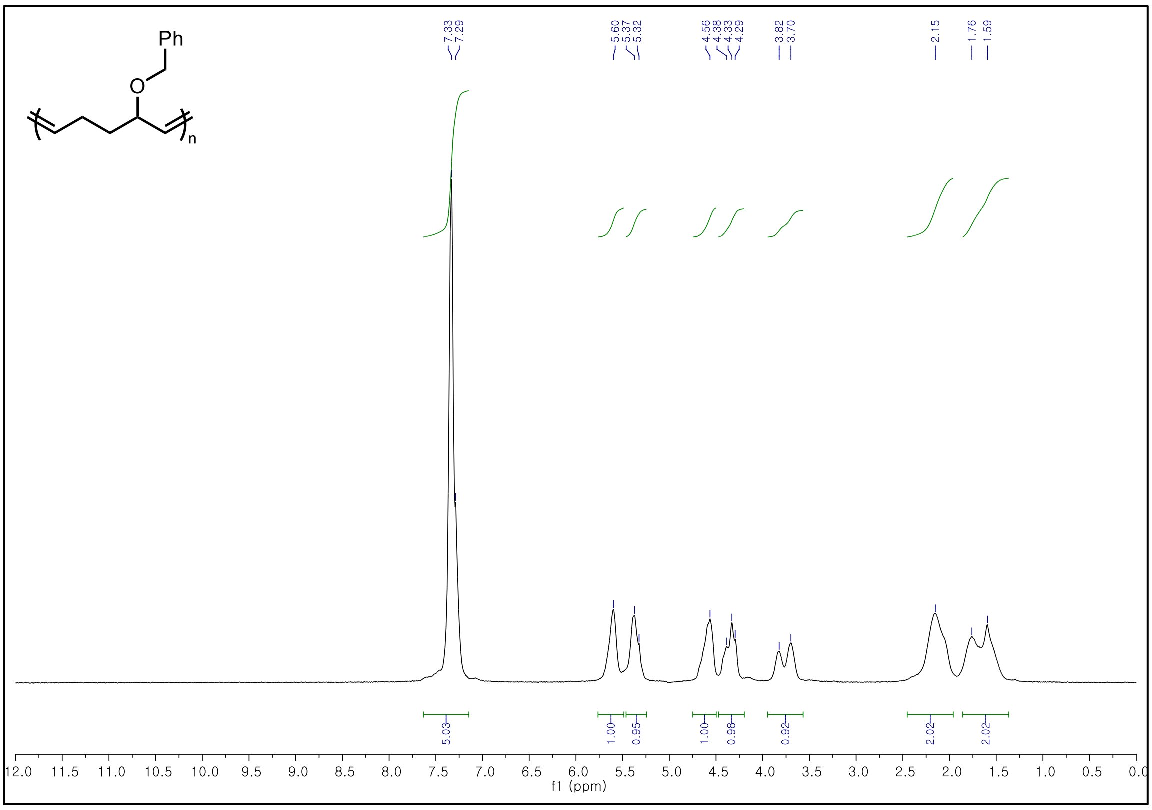

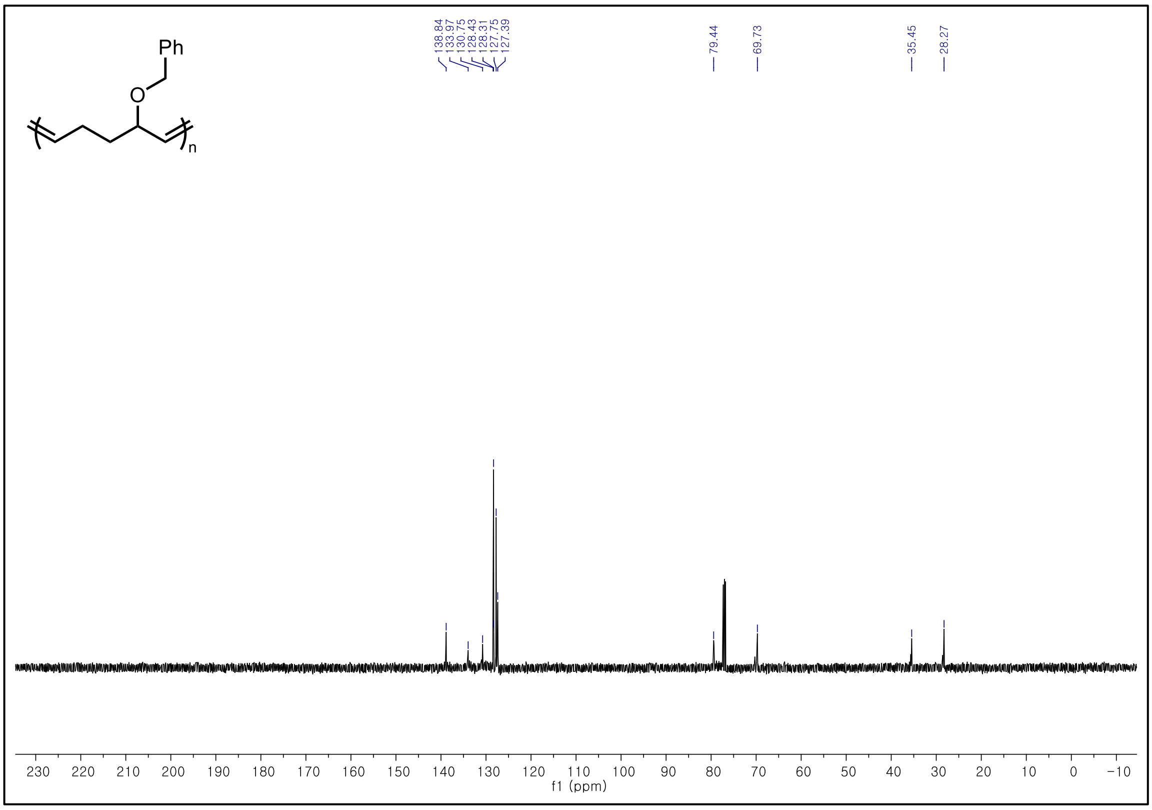

Supplementary Figure 73. ^{1}H NMR (**poly-14**) ($CDCl_3$, 300 MHz) and ^{13}C NMR (**poly-14**) ($CDCl_3$, 125 MHz)

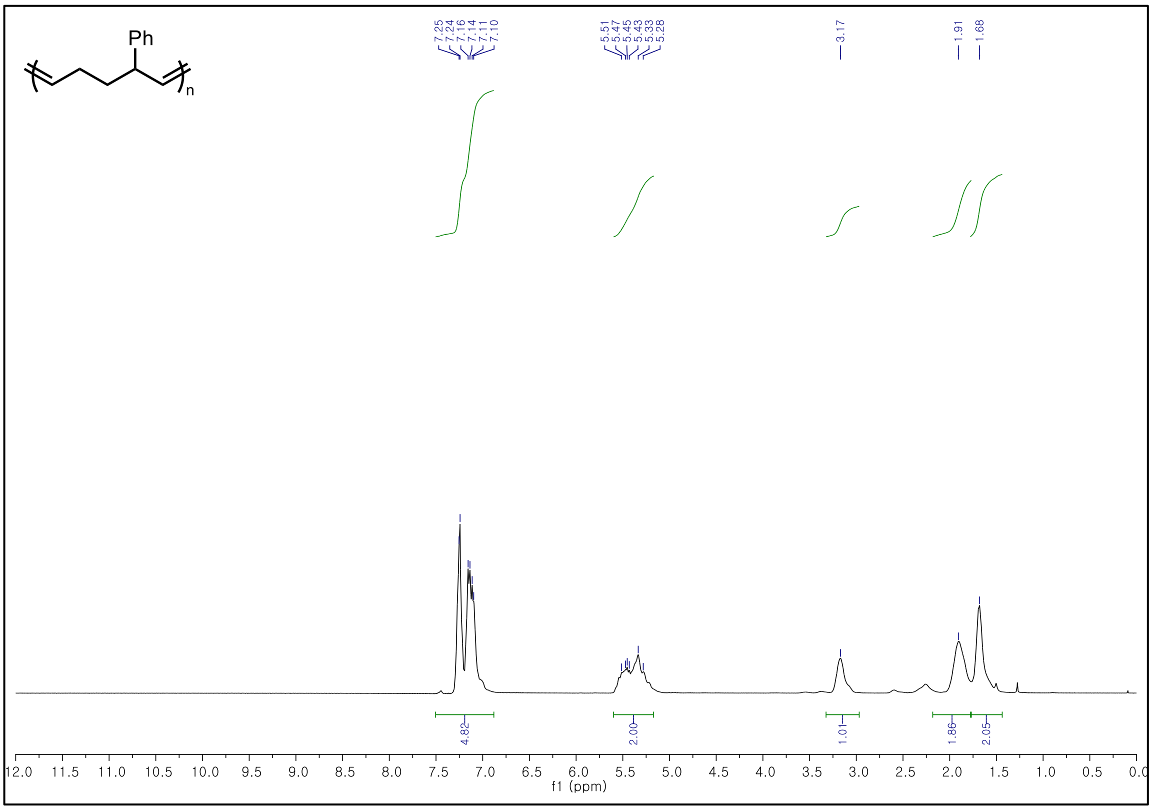

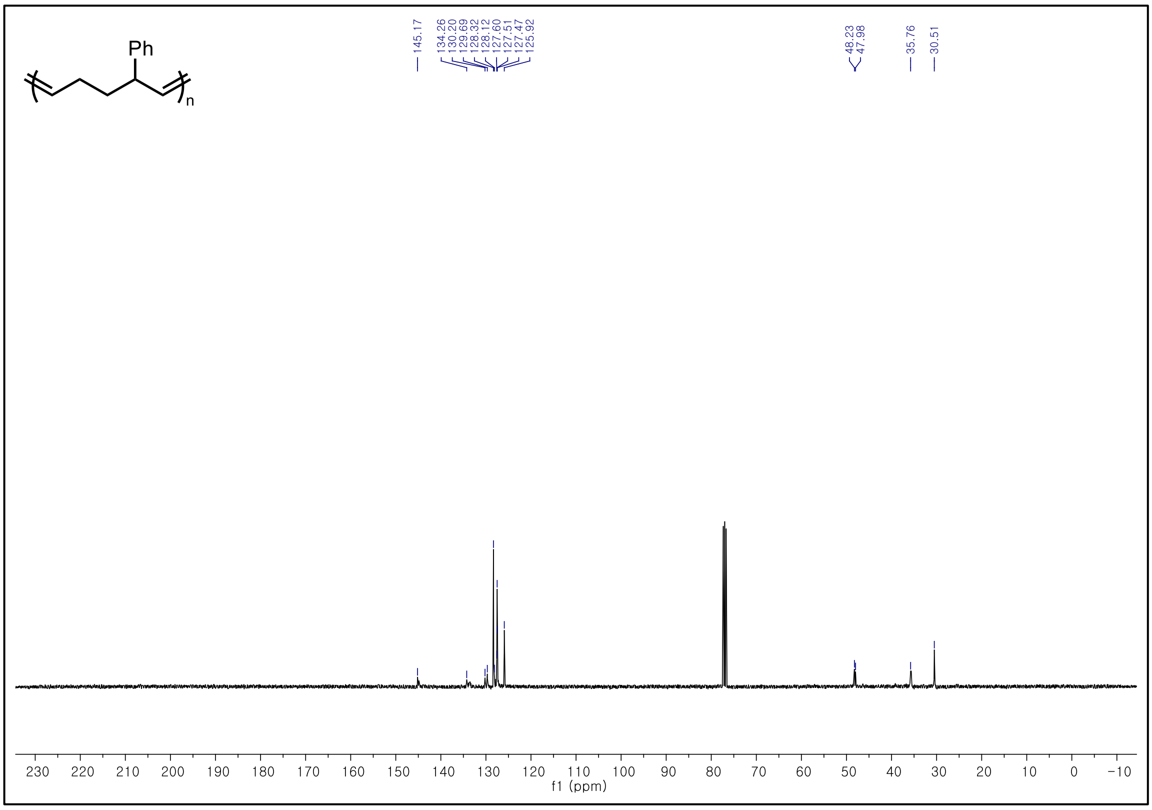

Supplementary Figure 74. ^{1}H NMR (**poly-15**) ($CDCl_3$, 400 MHz) and ^{13}C NMR (**poly-15**) ($CDCl_3$, 100 MHz)

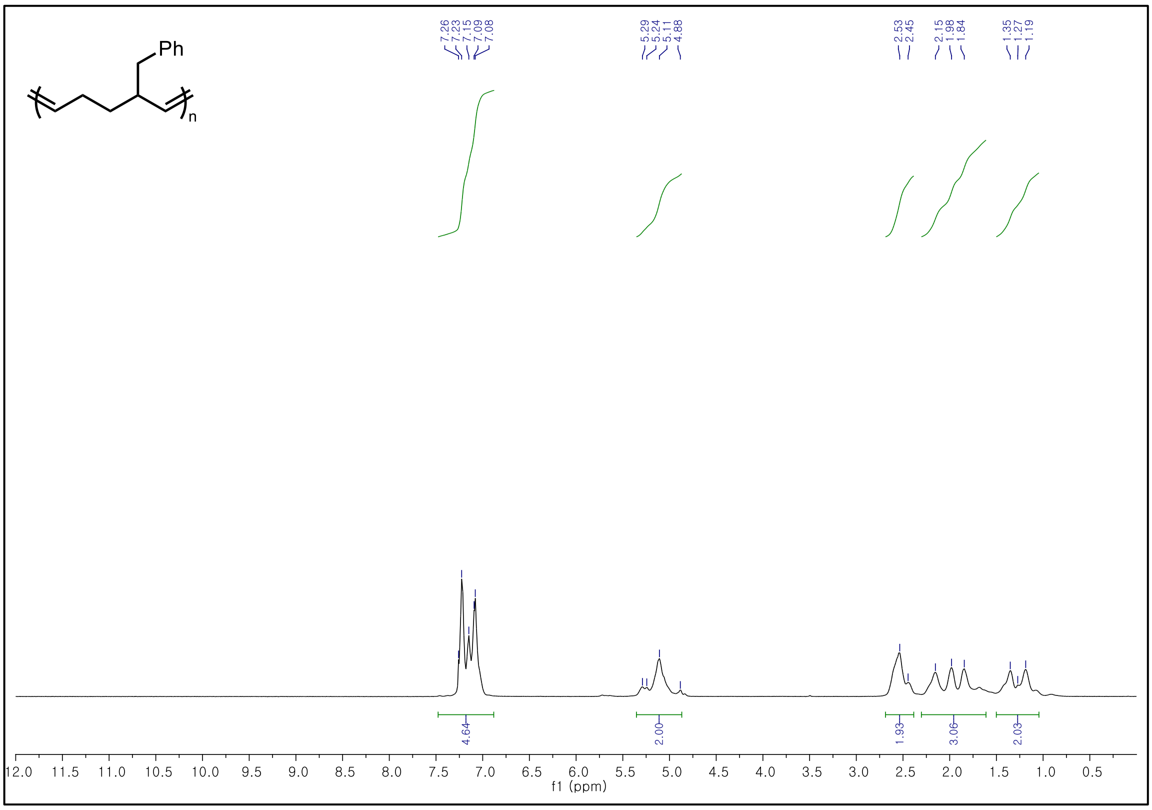

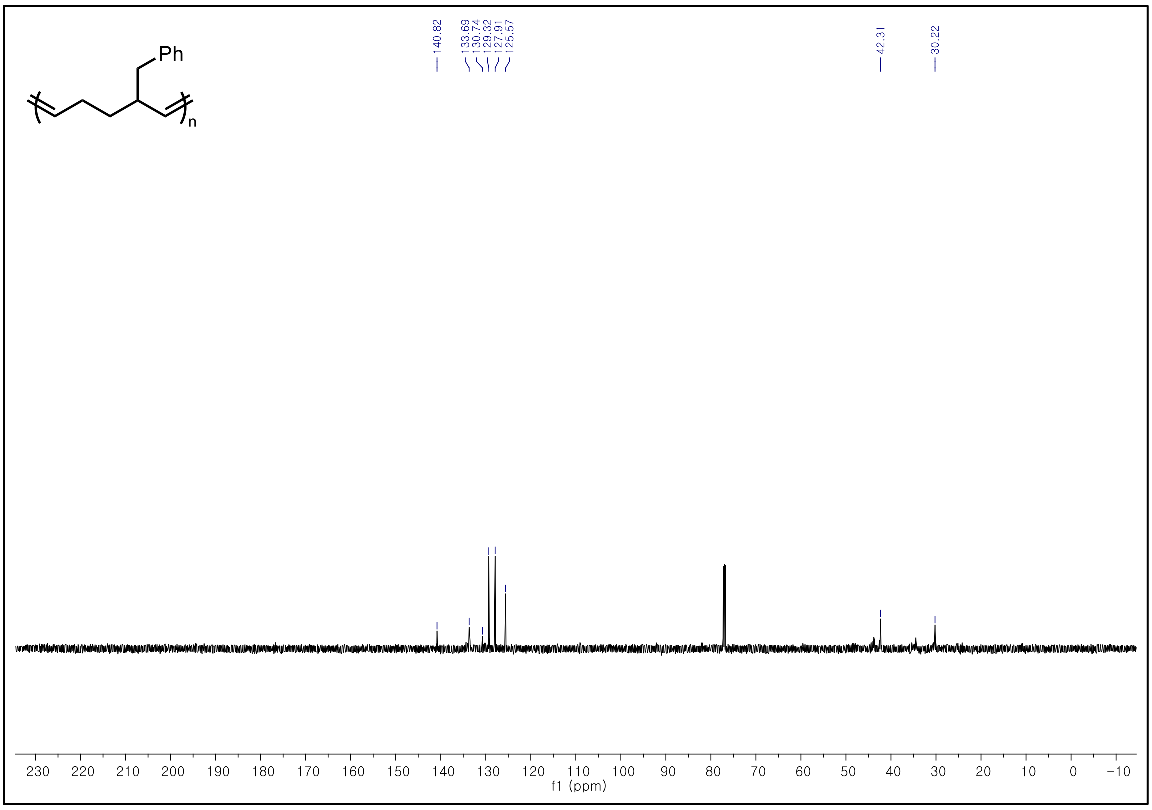

Supplementary Figure 75. ^{1}H NMR (**poly-16**) ($CDCl_3$, 500 MHz) and ^{13}C NMR (**poly-16**) ($CDCl_3$, 125 MHz)

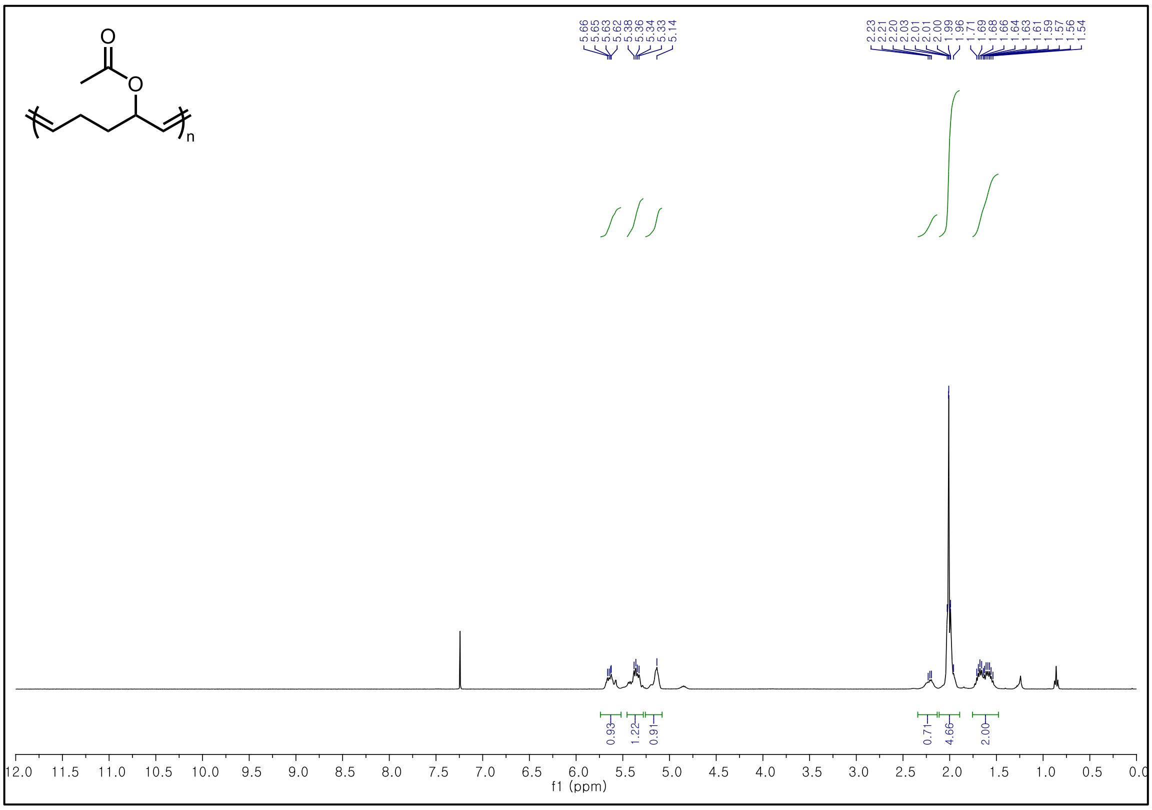

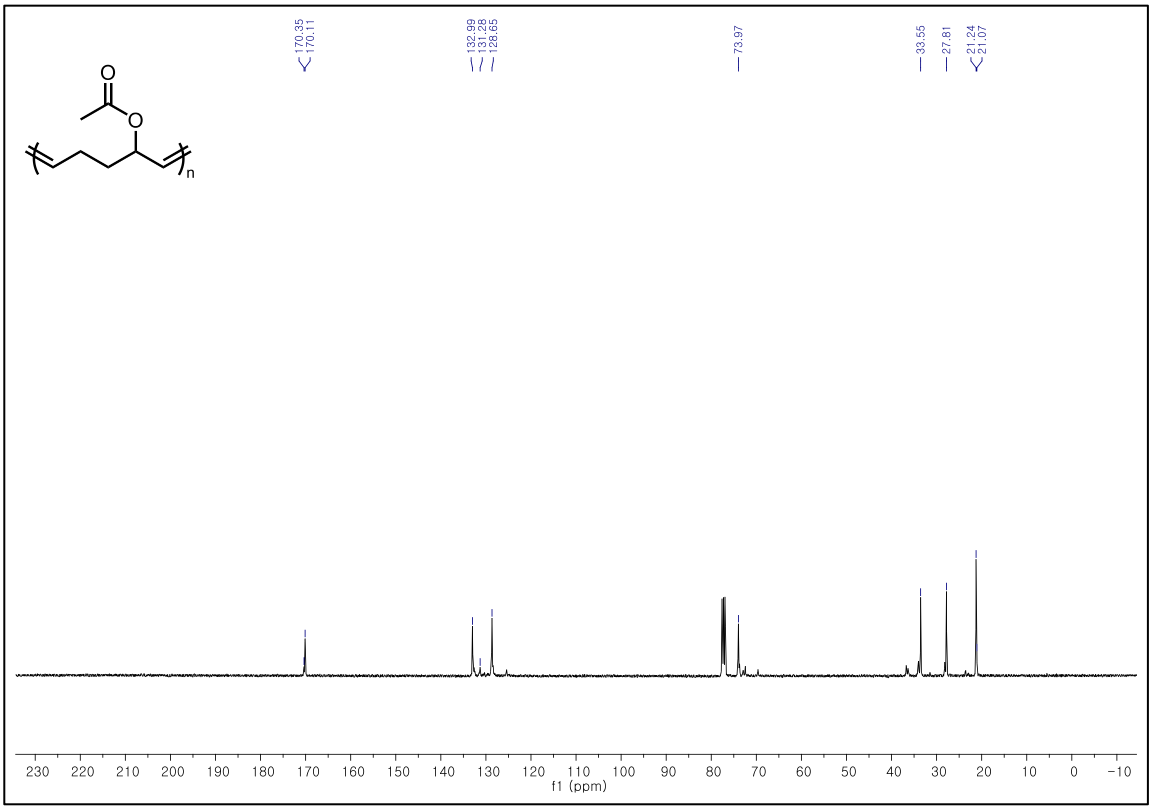

Supplementary Figure 76. ^{1}H NMR (**poly-17**) ($CDCl_3$, 500 MHz) and ^{13}C NMR (**poly-17**) ($CDCl_3$, 100 MHz)

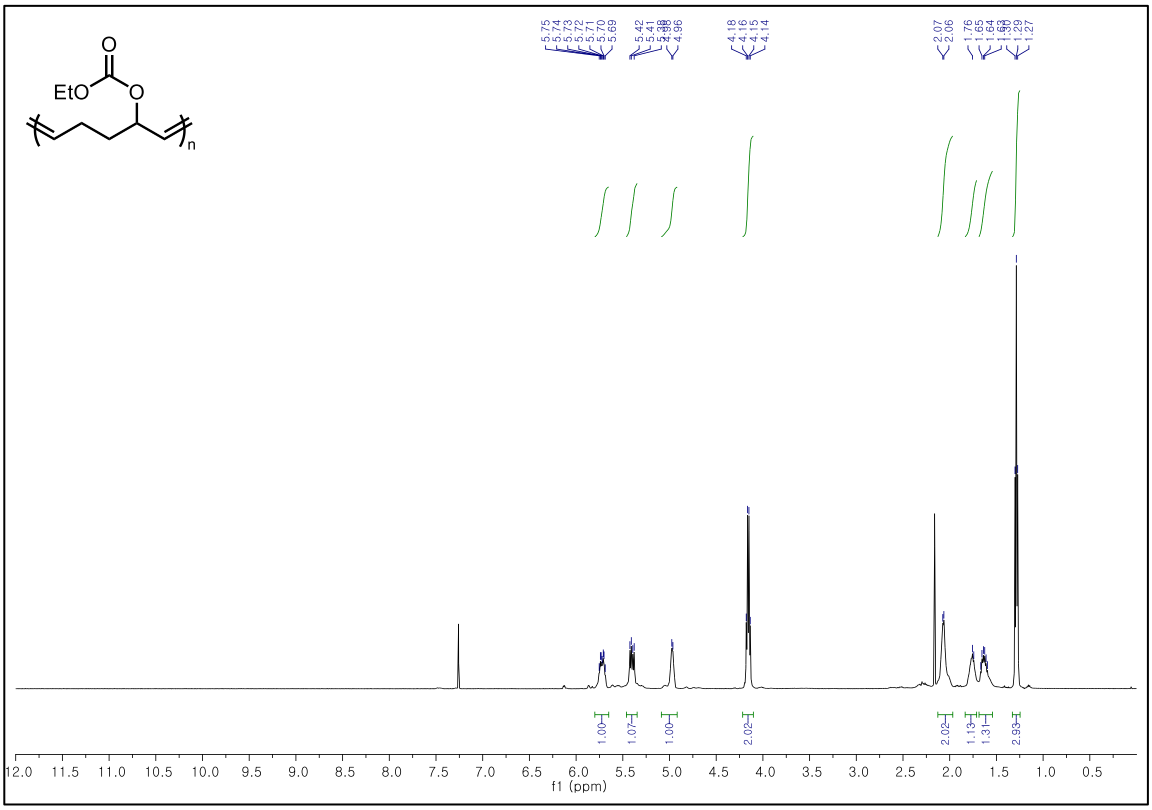

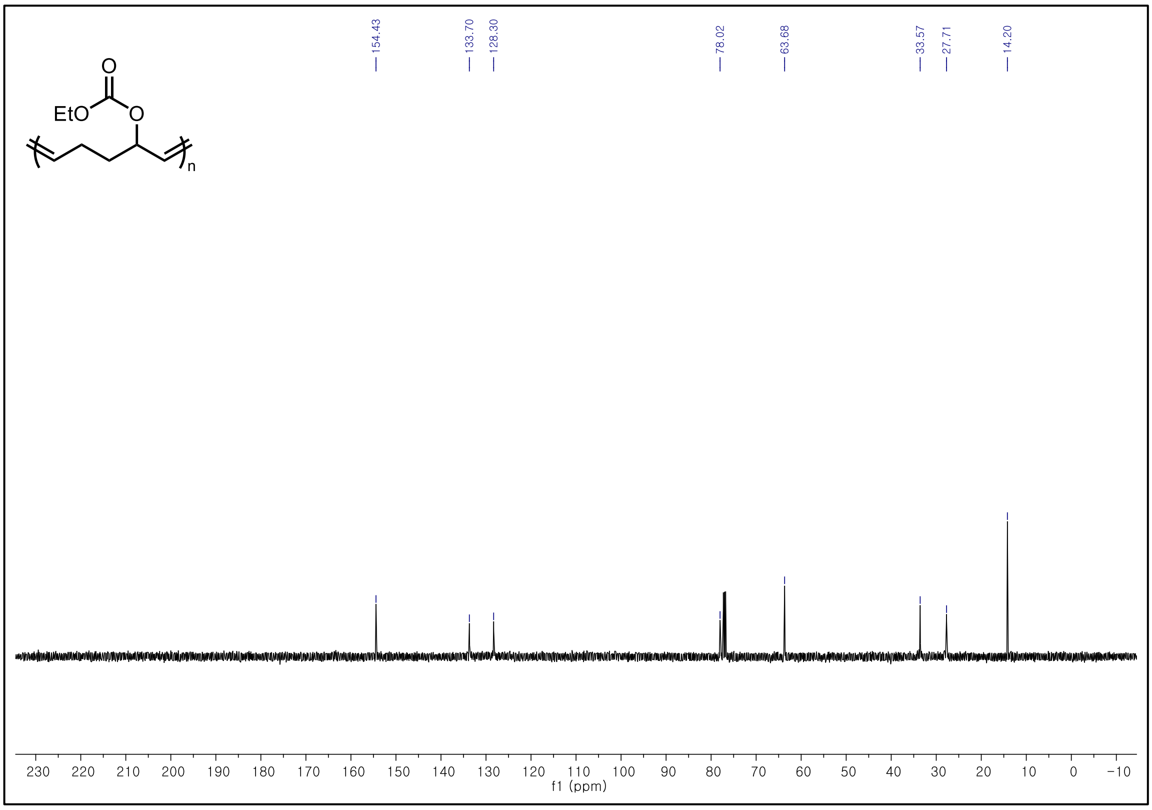

Supplementary Figure 77. ^{1}H NMR (**poly-18**) ($CDCl_3$, 500 MHz) and ^{13}C NMR (**poly-18**) ($CDCl_3$, 100 MHz)

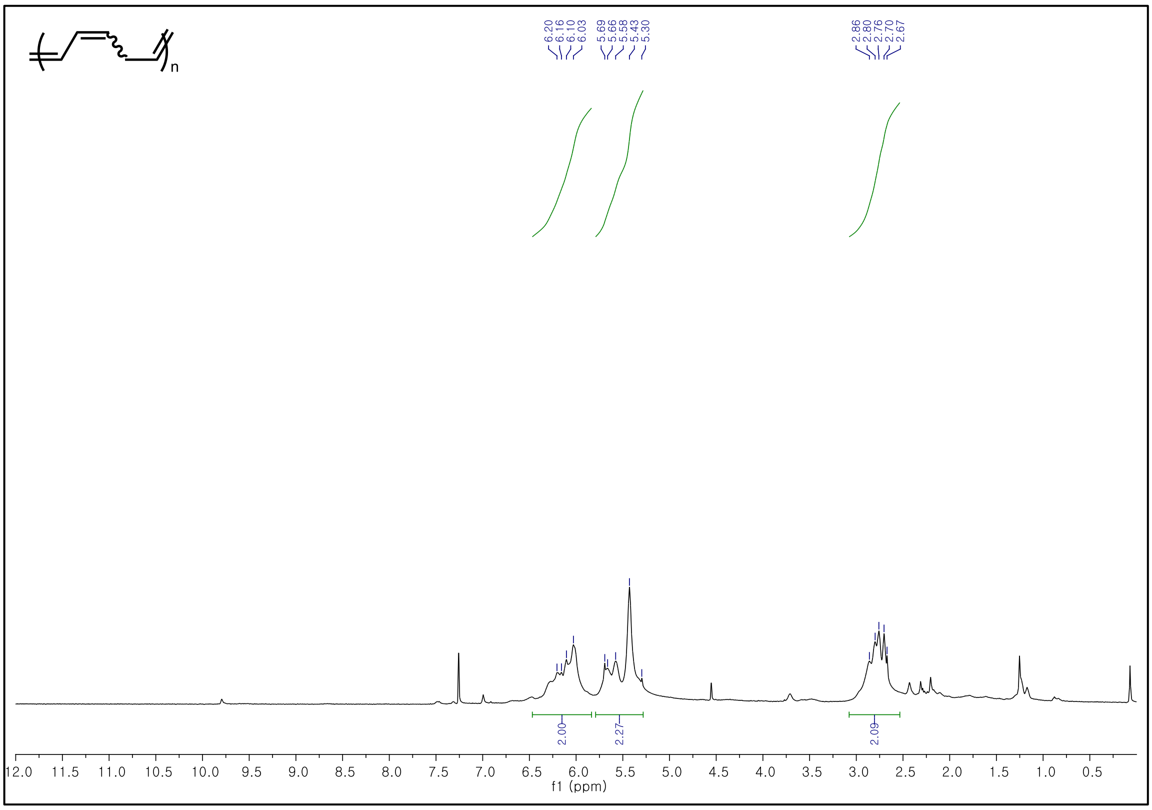

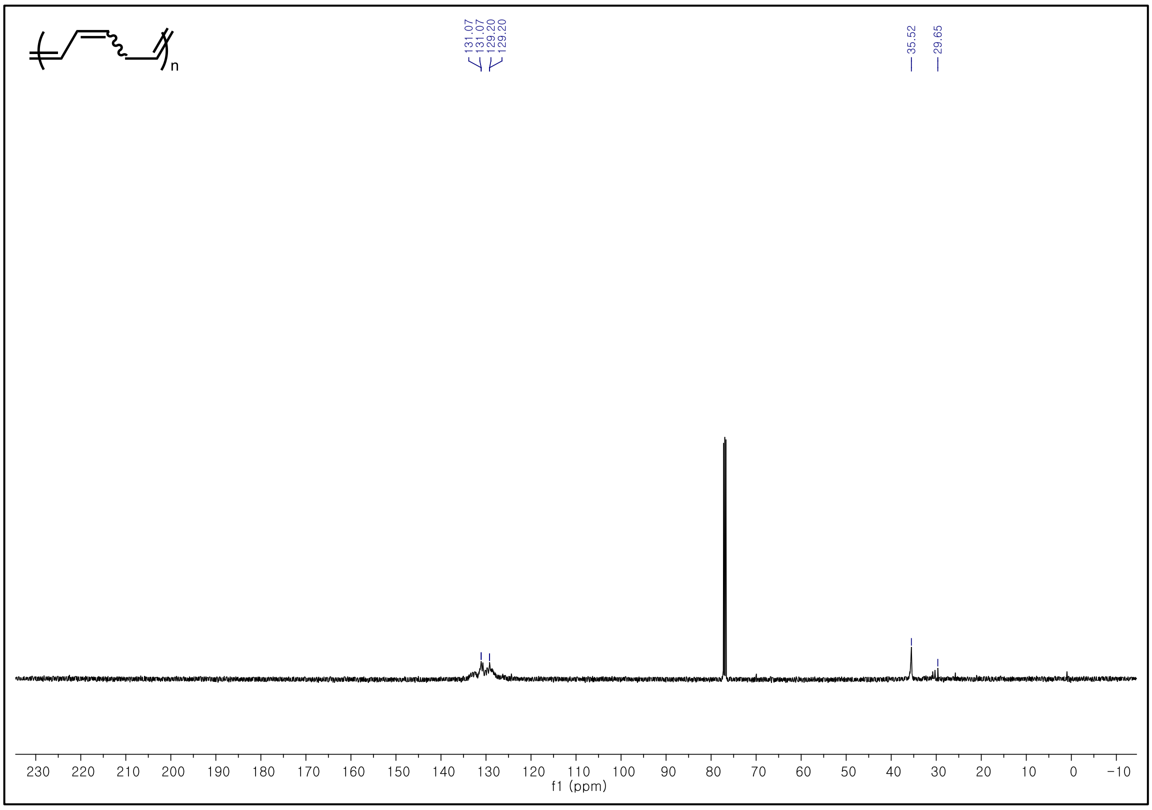

Supplementary Figure 78. ^{1}H NMR (**poly-CPD**, crude mixture) ($CDCl_3$, 500 MHz) and ^{13}C NMR (**poly-CPD**, crude mixture) ($CDCl_3$, 125 MHz)

[*N*-benzylphenylsulfonamido-κ*O*)methylene-κ*C*][1,3-Bis(2,4,6-trimethylphenyl)-2-imidazolidinylidene]dichlororuthenium (Ru-5) (CCDC 1526992)

Complex **Ru-5** was crystallized from Pentane. The atoms are depicted with 50% probability ellipsoids. Details about the analysis of the diffraction data are provided in CIF files provided as Supplementary Information.

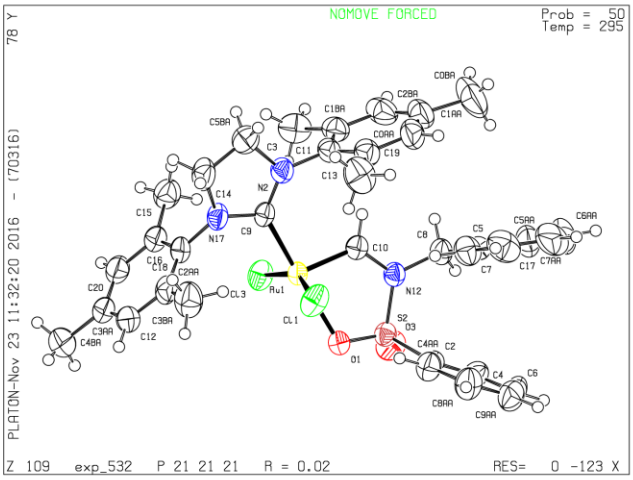

Supplementary Figure 79. X-ray structure of **Ru-5**

[*N*-benzyl-4-methoxyphenylsulfonamido-κ*O*)methylene-κ*C*][1,3-Bis(2,4,6-trimethylphenyl)-2-imidazolidinylidene]dichlororuthenium (Ru-6) (CCDC 1526991)

Complex **Ru-6** was crystallized from Pentane. The atoms are depicted with 50% probability ellipsoids. Details about the analysis of the diffraction data are provided in CIF files provided as Supplementary Information.

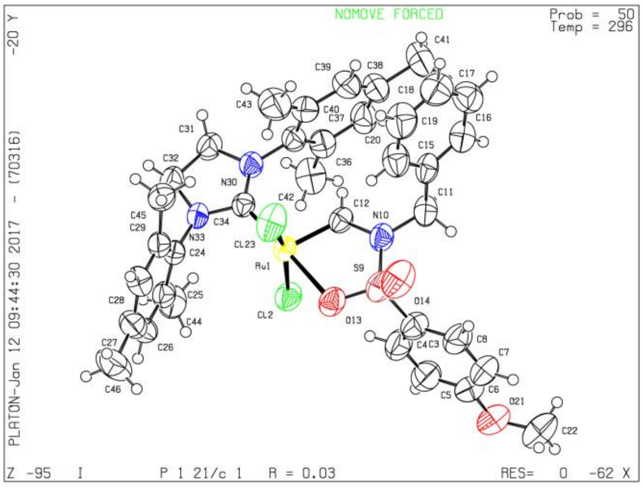

Supplementary Figure 80. X-ray structure of **Ru-6**

[*N*-benzyl-4-nitrophenylsulfonamido-κ*O*)methylene-κ*C*][1,3-Bis(2,4,6-trimethylphenyl)-2-imidazolidinylidene]dichlororuthenium (Ru-7) (CCDC 1526994)

Complex **Ru-7** was crystallized from Pentane. The atoms are depicted with 50% probability ellipsoids. Details about the analysis of the diffraction data are provided in CIF files provided as Supplementary Information.

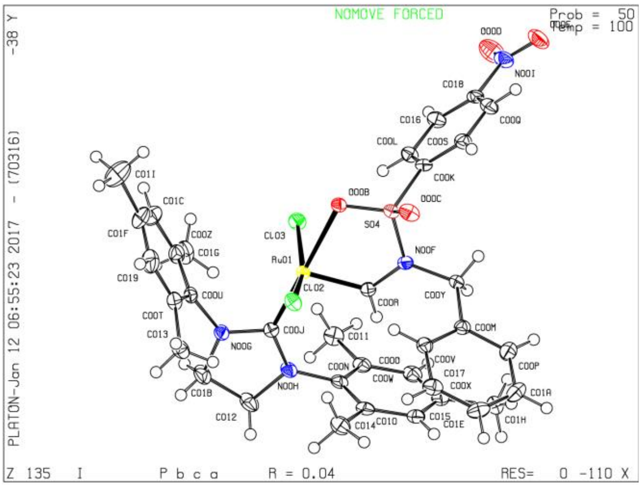

Supplementary Figure 81. X-ray structure of **Ru-7**

[1,3-Bis(2,4,6-trimethylphenyl)-2-imidazolidinylidene]dichloro[1,1,1-trifluoro-*N*-4-methoxyphenylmethylsulfonamido-κ*O*)methylene-κ*C*]ruthenium (Ru-9) (CCDC 1526990)

Complex **Ru-9** was crystallized from Pentane. The atoms are depicted with 50% probability ellipsoids. Details about the analysis of the diffraction data are provided in CIF files provided as Supplementary Information.

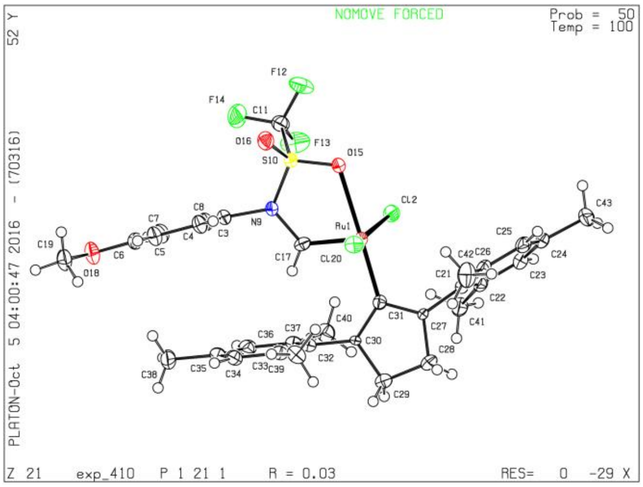

Supplementary Figure 82. X-ray structure of **Ru-9**

Supplementary References

1 Venkateswarlu, V. *et al.* C-H oxygenation and N-trifluoroacylation of arylamines under metal-free conditions: a convenient approach to 2-aminophenols and N-trifluoroacyl-ortho-aminophenols. *Chem. Eur. J.* **20**, 6641-6645, (2014).

2 Levison, J. J. & Robinson, S. D. Transition-metal complexes containing phosphorus ligands. Part III. Convenient syntheses of some triphenylphosphine complexes of the platinum metals. *J. Chem. Soc. A*, 2947-2954, (1970).

3 Kakiuchi, F. *et al.* Catalytic Addition of Aromatic Carbon–Hydrogen Bonds to Olefins with the Aid of Ruthenium Complexes. *Bull. Chem. Soc. Jpn.* **68**, 62-83, (1995).

4 Skrypnik, Y. G., Vasil'eva, N. V., Lyashchuk, S. N., Bezrodnyi, V. P. & Enya, V. I. Synthesis and structure of 2-N,N-dimethyl- and 2-N,N-diethylsulfamoyl-1-(3-indolyl)-1,2-dihydroisoquinolines. *Russ. J. Org. Chem.* **36**, 577-580 (2000).

5 Wheeler, K. W. & Degering, E. F. Preparation and properties of certain derivatives of sulfamide. *J. Am. Chem. Soc.* **66**, 1242-1243, (1944).

6 Zhu, M., Fujita, K. & Yamaguchi, R. Simple and versatile catalytic system for N-alkylation of sulfonamides with various alcohols. *Org. Lett.* **12**, 1336-1339, (2010).

7 Sanchez-Cantalejo, F., Priest, J. D. & Davies, P. W. A gold carbene manifold to prepare fused gamma-lactams by oxidative cyclisation of ynamides. *Chem. Eur. J.* **24**, 17215-17219, (2018).

8 Wang, Z., Li, X., Ji, P. & Cheng, J. P. Absolute pKas of sulfonamides in ionic liquids: comparisons to molecular solvents. *J. Org. Chem.* **81**, 11195-11200, (2016).

9 Harmata, M. *et al.* Expedient synthesis of sulfinamides from sulfonyl chlorides. *J. Org. Chem.* **72**, 683-685, (2007).

10 Chakravarti, R. *et al.* Functionalization of mesoporous carbon with superbasic MgO nanoparticles for the efficient synthesis of sulfinamides. *Chem. Eur. J.* **17**, 6673-6682, (2011).

11 Jiang, L., Job, G. E., Klapars, A. & Buchwald, S. L. Copper-catalyzed coupling of amides and carbamates with vinyl halides. *Org. Lett.* **5**, 3667-3669, (2003).

12 Xu, J., Fu, Y., Xiao, B., Gong, T. & Guo, Q. Room-temperature palladium(II)-catalyzed N-vinylation of sulfonamides and acylamides with vinyl acetate as vinyl source. *Tetrahedron Lett.* **51**, 5476-5479, (2010).

13 Feltenberger, J. B., Hayashi, R., Tang, Y., Babiash, E. S. C. & Hsung, R. P. Enamide-Benzyne-[2 + 2] Cycloaddition: Stereoselective Tandem [2 + 2]−Pericyclic Ring-Opening−Intramolecular N-Tethered [4 + 2] Cycloadditions. *Org. Lett.* **11**, 3666-3669, (2009).

14 Kobayashi, Y. *et al.* A medium fluorous Grubbs–Hoveyda 2nd generation catalyst for phase transfer catalysis of ring closing metathesis reactions. *Tetrahedron Lett.* **56**, 1363-1366, (2015).

15 Neary, W. J. & Kennemur, J. G. Variable temperature ROMP: Leveraging low ring strain thermodynamics to achieve well-defined polypentenamers. *Macromolecules* **50**, 4935-4941, (2017).

16 Benson, S. W. *et al.* Additivity rules for the estimation of thermochemical properties. *Chem. Rev.* **69**, 279-324, (1969).

17 Curtiss, L. A., Raghavachari, K., Redfern, P. C., Rassolov, V. & Pople, J. A. Gaussian-3 (G3) theory for molecules containing first and second-row atoms. *J. Chem. Phys.* **109**, 7764-7776, (1998).

18 Irikura, K. K. & Frurip, D. J. *Computational Thermochemistry*. Vol. 677 (American Chemical Society, 1998).

19 Holmes, J. L. & Aubry, C. Group additivity values for estimating the enthalpy of formation of organic compounds: an update and reappraisal. 1. C, H, and O. *J. Phys. Chem. A* **115**, 10576-10586, (2011).

20 Yin, G., Herdtweck, E. & Bach, T. Enantioselective access to bicyclo[4.2.0]octanes by a sequence of [2+2] photocycloaddition/reduction/fragmentation. *Chem. Eur. J.* **19**, 12639-12643, (2013).

21 Goksu, G., Ocal, N. & Kaufmann, D. E. Reductive Heck reactions of N-methyl-substituted tricyclic imides. *Molecules* **15**, 1302-1308, (2010).

22 Camm, K. D. *et al.* Tandem ROMP-hydrogenation with a third-generation Grubbs catalyst. *J. Am. Chem. Soc.* **129**, 4168-4169, (2007).

23 Liu, W. & RajanBabu, T. V. Reactivity and selectivity in hydrovinylation of strained alkenes. *J. Org. Chem.* **75**, 7636-7643, (2010).

24 Kang, E.-H., Yang, S., Yu, S. Y., Kim, J. & Choi, T.-L. Spontaneous evolution of nanostructures by light-driven growth of micelles obtained from in situ nanoparticlization of conjugated polymers. *J. Polym. Sci. A* **55**, 3058-3066, (2017).

25 Mugemana, C. *et al.* Ring opening metathesis polymerization of cyclopentene using a ruthenium catalyst confined by a branched polymer architecture. *Polym. Chem.* **7**, 2923-2928, (2016).

26 Pryde, D. C. *et al.* A flexible, efficient synthesis of (±)-carbocyclic phosphonic acid nucleoside derivatives. *Synlett*, 0765-0768, (2005).

27 Brookes, P. C., Milne, D. J., Murphy, P. J. & Spolaore, B. Epoxide rearrangements using dilithiated aminoalcohols as chiral bases. *Tetrahedron* **58**, 4675-4680, (2002).

28 Larock, R. C. & Baker, B. E. Palladium-catalyzed intermolecular allylic arylation of cyloalkenes. *Tetrahedron Lett.* **29**, 905-908, (1988).

29 Reile, I. *et al.* Heterogeneous platinum catalytic aerobic oxidation of cyclopentane-1,2-diols to cyclopentane-1,2-diones. *Tetrahedron* **70**, 3608-3613, (2014).

30 Hansson, S., Heumann, A., Rein, T. & Aakermark, B. Preparation of allylic acetates from simple alkenes by palladium(II)-catalyzed acetoxylation. *J. Org. Chem.* **55**, 975-984, (1990).

31 Lee, H. K., Bang, K. T., Hess, A., Grubbs, R. H. & Choi, T. L. Multiple olefin metathesis polymerization that combines all three olefin metathesis transformations: ring-opening, ring-closing, and cross metathesis. *J. Am. Chem. Soc.* **137**, 9262-9265, (2015).

32 Rounds, H. R., Zeller, M. & Uyeda, C. Dinuclear pathways for the activation of strained three-membered rings. *Organometallics* **37**, 545-550, (2018).

33 Gerdil, R., Liu, H. Y. & Bernardinelli, G. Organic reactions in the solid state: Reactions of enclathrated 3,4-epoxyclopentanone (=6-oxobicyclo[3.1.0]hexan-3-one) in tri-o-thymotide and absolute configuration of 4-hydroxy- and 4-chlorocyclopent-2-en-1-one. *Helv. Chim. Acta.* **82**, 418-434 (1999).

34 Tufariello, J. J., Bayer, A. C. & Spadaro, J. J. Synthesis and reactivity of exo- and endo-bicyclo[2.1.0]pentan-5-ol and their derivatives. *J. Am. Chem. Soc.* **101**, 3309-3315, (1979).

35 Hejl, A., Scherman, O. A. & Grubbs, R. H. Ring-opening metathesis polymerization of functionalized Low-strain monomers with ruthenium-based catalysts. *Macromolecules* **38**, 7214-7218, (2005).
